# Widespread Distribution and Expression of Gamma A (UMB), an Uncultured, Diazotrophic, γ-Proteobacterial *nifH* Phylotype

**DOI:** 10.1371/journal.pone.0128912

**Published:** 2015-06-23

**Authors:** Rebecca Langlois, Tobias Großkopf, Matthew Mills, Shigenobu Takeda, Julie LaRoche

**Affiliations:** 1 Department of Oceanography, Dalhousie University, Halifax, NS, Canada; 2 The University of Warwick, Coventry, United Kingdom; 3 Environmental Earth System Science, Stanford University, Stanford, California, United States of America; 4 Faculty of Fisheries, Nagasaki University, Nagasaki, Japan; Royal Netherlands Institute of Sea Research (NIOZ), NETHERLANDS

## Abstract

Marine dinitrogen (N_2_) fixation studies have focused nearly exclusively on cyanobacterial diazotrophs; however γ-proteobacteria are an abundant component of the marine community and have been largely overlooked until recently. Here we present a phylogenetic analysis of all *nifH* γ-proteobacterial sequences available in public databases and qPCR data of a γ-proteobacterial phylotype, Gamma A (UMB), obtained during several research cruises. Our analysis revealed a complex diversity of diazotrophic γ-proteobacteria. One phylotype in particular, Gamma A, was described in several traditional and quantitative PCR studies. Though several γ-proteobacterial *nifH* sequences have been described as laboratory contaminants, Gamma A is part of a large cluster of sequences isolated from marine environments and distantly related to the clade of contaminants. Using a TaqMan probe and primer set, Gamma A *nifH* DNA abundance and expression were analyzed in nearly 1000 samples collected during 15 cruises to the Atlantic and Pacific Oceans. The data showed that Gamma A is an active, cosmopolitan diazotroph found throughout oxygenated, oligotrophic waters reaching maximum abundances of 8 x 10^4^ *nifH* DNA copies l^-1^ and 5 x 10^5^ *nifH* transcript copies l^-1^. Gamma A *nifH* transcript abundances were on average 3 fold higher than *nifH* DNA abundances. The widespread distribution and activity of Gamma A indicate that it has potential to be a globally important N_2_ fixing organism.

## Introduction

Biological dinitrogen (N_2_) fixation is an important source of fixed nitrogen to many oligotrophic oceanic regions carried out by a diverse group of Bacteria and Archaea called diazotrophs. All diazotrophic organisms contain the nitrogenase enzyme, which is the only enzyme known to catalyze the reduction of N_2_ gas to ammonia. Nitrogenase is composed of two subunits, dinitrogenase reductase and dinitrogenase. The former provides the electrons to the active site of N_2_ fixation in the latter subunit. The *nifH* gene, which codes for dinitrogenase reductase, is highly conserved and present in all diazotrophs, making it an ideal candidate for phylogenetic analyses [1].

The presence of oceanic diazotrophs belonging to γ-proteobacteria was first reported by Zehr et al. (1998) [2]. However, subsequent research focused mainly on cyanobacterial diazotrophs, including *Trichodesmium*, the unicellular cyanobacteria groups A and B (UCYN A and UCYN B) and diatom-diazotroph symbionts [3]. A recent high-throughput sequencing analysis of *nifH* PCR amplicons from 12 sites world-wide recovered *nifH* sequences from α, β, δ, and γ-proteobacteria, methanogens, *Clostridia* and cyanobacteria and indicated that *nifH* genes belonging to non-cyanobacterial diazotrophs were diverse and dominant. This suggests that non-cyanobacterial diazotrophs may play a bigger role in global N_2_ fixation than previously thought [4].

Proteobacteria are the dominant microbes in the oceans [5]. Within the proteobacteria, γ-proteobacteria form an abundant and metabolically diverse class in marine systems and are capable of photoautotrophy, chemoautotrophy, chemoheterotrophy and facultative anaeroby [6]. In the Sargasso Sea, γ-proteobacterial sequences were the second most abundant group following α-proteobacterial sequences [7], accounting for 20% of the sequences recovered. In an analysis of over 30 Sorcerer II Global Ocean Samples, γ-proteobacteria 16S rRNA gene sequences were the third most dominant group (13%) of sequences following α-proteobacteria (32%) and unclassified proteobacteria (16%) [8]. Among the γ–proteobacteria, the SAR86 ribotype has been described as the largest group of uncultured microbes [9]. Given the prominence of γ-proteobacteria in the ocean and the common occurrence of diazotrophy among this phylum, there is a large potential for the presence of diazotrophic γ-proteobacteria in marine systems.

Among cultured representatives, the soil bacterium *Azotobacter vinelandii* is arguably the best described γ-proteobacterial diazotroph, and has served as a model organism for the description of the nitrogenase enzyme complex [1]. Other cultured representatives include pathogens such as *Klebsiella pneumonia*, a human pathogen also found in freshwater and *Vibrio splendidus*, an oyster pathogen [10]. The cultivated marine γ–proteobacterial species include *Vibrio natriegens* and *Vibrio diazotrophicus*, found in salt marsh mud and marine and estuarine waters, respectively [11, 12]. Many γ-proteobacterial *nifH* phylotypes described from the open ocean so far have low similarity (<86%) to cultivated diazotrophic γ-proteobacteria [2, 13, 14].

In an early study on *nifH* diversity, Bird et al. (2005) [15] described a group of γ-proteobacterial sequences (Uncultured Marine Bacteria, UMB) that was dominant and actively transcribed the *nifH* gene throughout the water column in the Arabian Sea. Identical and similar sequences have also been described in the Atlantic and Pacific Oceans [2, 14, 16, 17]. These γ-proteobacterial *nifH* sequences accounted for over 6% of the sum of all *nifH* DNA sequences (*Trichodesmium*, UCYN A, B, and C, Cluster III and two γ-proteobacteria phylotypes) detected by qPCR in a study in the Atlantic Ocean and were the most broadly distributed phylotype [18]. This group was also widely distributed in the Pacific Ocean [19]. In the South Pacific Gyre, γ-proteobacteria phylotypes were dominant at all but two stations [13]. Data is accumulating showing that diazotrophic γ-proteobacteria are prevalent and active throughout open ocean systems. Given the paucity of data available presently, one can only speculate about the physiology and N_2_ fixing potential of marine diazotrophic γ-proteobacteria.

In an effort to advance our knowledge about diazotrophic γ-proteobacteria we looked at the diversity and distribution of γ-proteobacterial *nifH* genes, in particular one sequence (Gamma A, AY896371) that has been targeted by quantitative PCR (qPCR) in several studies [16, 18, 20]. We conducted an in-depth phylogenetic analysis of publically available γ-proteobacterial *nifH* genes and the γ-proteobacterial *nifH* phylotypes of marine origin used in qPCR studies to date in order assess the phylogenetic relationship of the Gamma A (UMB) clade to the broader diversity of *nifH* sequences clustering within the γ-proteobacteria. In addition, abundances of Gamma A *nifH* DNA and transcripts (cDNA) were measured during several cruises to the Atlantic and Pacific Oceans. The spatial and temporal distribution of Gamma A was analyzed with respect to available physical and chemical properties of the water column. Results indicate that Gamma A phylotypes and transcripts are found throughout oligotrophic surface waters in both the Pacific and Atlantic Oceans. The wide distribution and active transcription of the *nifH* gene indicate that this group may be important for global N_2_ fixation.

## Materials and Methods

### Sample collection, genetic material isolation and cDNA synthesis

Samples for nucleic acid extraction were collected during 15 cruises to the Atlantic and Pacific Oceans (Table 1). All samples analyzed during this study were collected in international waters and did not require specific permissions. This study did not involve protected or endangered species. One to eight liters of bulk seawater were collected using either a CTD, underway ship pump or trace metal clean tow-fish. Water was filtered onto 0.2 μm Durapore (Millipore) filters using low vacuum (2 kPa) and immediately frozen at -80°C until extraction in the laboratory. Generally, filtrations were stopped either after 2 l of seawater had been filtered or 2 hours had passed. Filtrations usually lasted 45 min. Samples were processed in a darkened, air-conditioned laboratory.

**Table 1:** Summary of cruise information and sample preparation.

Name	Dates	Location	No. of Samples	Water Filtered (l)	Collection Method	Extraction Method
AMT 17	Nov. 2005	N-S Atlantic transect	70	1–2	CTD, Fish	RNA/DNA Mini Kit
COST	Feb. 2005	SW Pacific	41	1–2	CTD	RNA/DNA Mini Kit
D361	Feb.-Mar. 2011	Tropical-Subtropical Atlantic	354	1–2	CTD, Fish	RNA/DNA Mini Kit
KH 04 05	Dec.-Mar. 2005	S-N Pacific transect	45	1–2	CTD	Maxwell DNA
KH 05 02	Aug.-Sept. 2005	S-N Pacific transect	39	1–2	CTD	Maxwell DNA
KT 05 24	Oct. 2005	W Sub-tropical N Pacific	22	1–2	CTD	Maxwell DNA
M55^a^	Oct.-Nov. 2002	W-E Sub-tropical Atlantic transect	61	1–2	CTD, Fish	DNA Plant Mini Kit
M60^a^	Mar.-Apr. 2004	W-E N Atlantic transect	47	1–2	CTD, Fish	DNA Plant Mini Kit
M68/2	Jun.-Jul. 2006	W-E Tropical Atlantic transect	80	1–2	CTD	RNA/DNA Mini Kit
M68/3	Jul.-Aug. 2006	E Sub-tropical N Atlantic	113	1–2	CTD, Fish	RNA/DNA Mini Kit
N Cycle	Mar.-April 2006	SW Pacific	11	1–2	CTD	RNA/DNA Mini Kit
P284^a^	Mar. 2002	E Sub-tropical N Atlantic	12	1–2	CTD	DNA Plant Mini Kit
P332^b^	Feb. 2006	E Sub-tropical N Atlantic	41	1–2	CTD	RNA/DNA Mini Kit
PINTS	Jan.-Feb. 2010	SW Pacific	36	1–2	CTD	RNA/DNA Mini Kit
S152^a^	Dec. 2000	NW Tropical Atlantic	20	1–8	Underway pump	DNA Plant Mini Kit

^a^[18].

^b^[50].

Samples were extracted using one of three methods (see Table 1 description): Qiagen DNA Plant Mini Kit, Qiagen RNA/DNA Mini Kit or automated Maxwell 16 Tissue DNA Kit (Promega). Samples were extracted according to the manufacturers’ instructions after filter preparation. All filters were prepared in the following manner. Filters were first broken up by holding the tube in liquid nitrogen for at least 30 s and then pulverized using either a sterile pipette tip or a plastic pestle. 100 μl of lysozyme (5 mg ml^-1^) was added to the filter pieces. It was then vortexed for 30 s and incubated for 10 min at room temperature. The kit lysis buffer was then added and the tube was vortexed again for 30 s. Samples from the D361 cruise and those extracted with the Plant Kit (Table 1) were then put through a QiaShredder column (Qiagen) before proceeding with the manufacturer’s instructions. Extraction of samples using the other kits directly followed filter preparation using the kit manufacturer’s instructions.

DNA and RNA samples extracted using Qiagen kits were eluted with 40–80 μl of TE buffer or RNAse-free water, respectively. Maxwell extracted samples were eluted in 180–250 μl of the elution buffer provided by the manufacturer. Due to uneven evaporation, the amount of elute remaining at the end of the Maxwell automatic extraction protocol was recorded. Blank extractions (no filters added) were routinely conducted to test extraction reagents for contamination. DNA and RNA concentrations were measured in all samples using the Picogreen and Ribogreen Quantitation Kits (Molecular Probes), respectively. No DNA or RNA was detected in the blank extraction samples. Possible contaminant DNA in RNA samples was removed completely by using the Turbo DNA-*free* kit (Ambion). 6 μl RNA (6–108 ng μl^-1^) was then transcribed to cDNA using Superscript III reverse transcriptase (Invitrogen), according to the manufacturer’s instructions. The nested *nifH* PCR reverse primers nifH2 (5’ ADNGCCATCATYTCNCC 3’) and nifH3 (5’ ATRTTRTTNGCNGCRTA 3’) [21] were used. No reverse-transcription (NRT) control reactions were included to test cDNA for DNA contamination.

### Abundance estimations using qPCR

Gamma A *nifH* abundances were estimated in DNA (992 samples) and cDNA (673 samples) using the TaqMan probe qPCR method described in Langlois et al. (2008); forward primer- 5’-TTATGATGTTCTAGGTGATGTG-3‘, reverse primer- 5‘-AACAATGTAGATTTCCTGAG CCTTATTC-3‘ and probe- 5‘-TTGCAATGCCTATTCG-3‘. A qPCR master mix was made using TaqMan master mix (Applied Biosystems), 5 pmol forward primer, 5 pmol reverse primer, 25 pmol probe, 10 μg BSA and either 1 μl of sample (2–80 ng DNA or 2–27 ng cDNA) or 5 μl of water or plasmid standard. Samples were run either in triplicate or duplicate. Duplicate no template controls (NTCs, water) and 7-point plasmid standards were run on every plate. Samples were run on ABI Real-Time PCR cyclers using the default program with 45 cycles and raw data was analyzed using software from the manufacturer (Applied Biosystems).

No amplification was seen in NTCs, blank extraction samples, NRTs or RNA samples, indicating that all reagents and samples were clean and that there was no DNA contaminating the cDNA. The average slope and intercept of the standard curves were -3.38 +/- 0.08 and 38.15 +/- 0.44, respectively. The average primer efficiency (E = 10^-1/slope^-1) was 99.6% (range 94.9–104.2%). The qPCR cycler detection limit was 1 copy reaction^-1^. After accounting for elution and filtration volumes, the actual detection limit ranged from 20 to 80 copies l^-1^. Samples where detection was not observed in all replicates were considered not quantifiable and removed from the data set (four samples in total).

In order to compare *nifH* transcript abundances between areas of high and low *nifH* DNA abundances, cDNA abundances were normalized to corresponding DNA abundances (cDNA:DNA ratios). All samples where *nifH* abundances were greater than 80 copies l^-1^ in both the DNA and cDNA samples were used to calculate cDNA:DNA *nifH* ratios.

### Sequence analysis

The Gamma A target nucleotide sequence (AY896371) in our qPCR assay was compared against the NCBI data bank using a Blastn search with the default maximum number of target sequences changed to 20000. Sample collection information and references were downloaded for all identical and highly similar (>99%) sequences (90 sequences). Sequences that were targets in qPCR studies were downloaded individually and compared using BioEdit Sequence Alignment Editor (v. 7.0.5.3). The 20000 Blastn search result sequences were downloaded and sequences that did not correctly translate into an uninterrupted NifH protein were removed. All non-γ-proteobacterial sequences were also removed, leaving 6240 sequences (S1 Fig, S1 Table). Two comparisons were made using the Blastn search results. First all sequences annotated as ‘uncultured’ or ‘unidentified’, except for sequences used as targets in qPCR studies of marine origin, were removed, allowing the comparison of key sequences in the context of cultured organisms. Then the ‘uncultured’ and ‘unidentified’ sequences (regardless of origin) were added back to the alignment. The following pipeline was used to construct all phylogenetic trees. Sequence identities were compared and operational taxonomic units (OTUs) at 100%, 99%, and 97% identical were formed using h-cd-hit-est [22]. Unless stated otherwise, the OTUs presented are at 97% (> 97% sequence similarity). Sequences were then aligned using the Multiple Sequence Comparison by Log Expectation tool (www.ebi.ac.uk). Alignments were checked to ensure that the correct reading frame was preserved. Two phylogenetic trees of each alignment were compared using FigTree (v1.4.1). Sequences were imported into JalView [23] and a neighbor-joining tree was constructed. RAxML (Randomized Accelerated Maximum Likelihood) Black Box was used to construct a maximum-likelihood tree [24]. Conserved properties between the two tree construction methods were identified in the phylogenetic trees by black branches and bootstrap values.

### Statistical analysis

Gamma A *nifH* abundances were analyzed in the context of a suite of environmental parameters including nutrients, salinity, temperature and oxygen collected concurrently. Surface *nifH* phylotype distribution and nutrient plots were constructed using Ocean Data View (v 4.3.6). A Principle Components Analysis (PCA) was conducted using only samples (344) for which all metadata (temperature, salinity, oxygen, silicate, nitrate and phosphate) were collected using PRIMER 6 (v 6.1.12). Before the PCA, draftsman’s plots of all variables were made to evaluate the magnitude and skew of the variables and to identify variable pairs with high correlations. After transformation, variable values were within three orders of magnitude of each other and were distributed evenly. Nitrate concentrations were highly correlated with phosphate (R^2^ = 0.98) and oxygen (R^2^ = 0.90) and were not used in the PCA. The N:P ratio did not correlate highly with the other variables and was used instead of the nitrate and phosphate concentrations. Variables were normalized ((x-μ)*σ ^-1^, where x is the sample variable, μ is the mean, and σ is the standard deviation) before proceeding with the PCA. A resemblance matrix using the Euclidean distance measure was calculated and a hierarchical agglomerative cluster analysis was performed to investigate grouping of samples. Samples were also assigned to categories based on sample collection depth (surface- <20 m, mid- 20–100 m, or deep- >100 m) and type (coastal- <1000 km to continental margin or open- >1000 km to continental margin). An Analysis of Similarities (ANOSIM) test was performed to determine the statistical significance of the categories at a significance level of 0.1%. The environmental variables were further analyzed by PCA cluster using MiniTab17 software. ANOVA (with Tukey post-hoc) and t-tests were run and considered statistically significant at P < 0.05.

## Results and Discussion

### Phylogenetic analysis of γ-proteobacterial *nifH*

**Fig 1:**
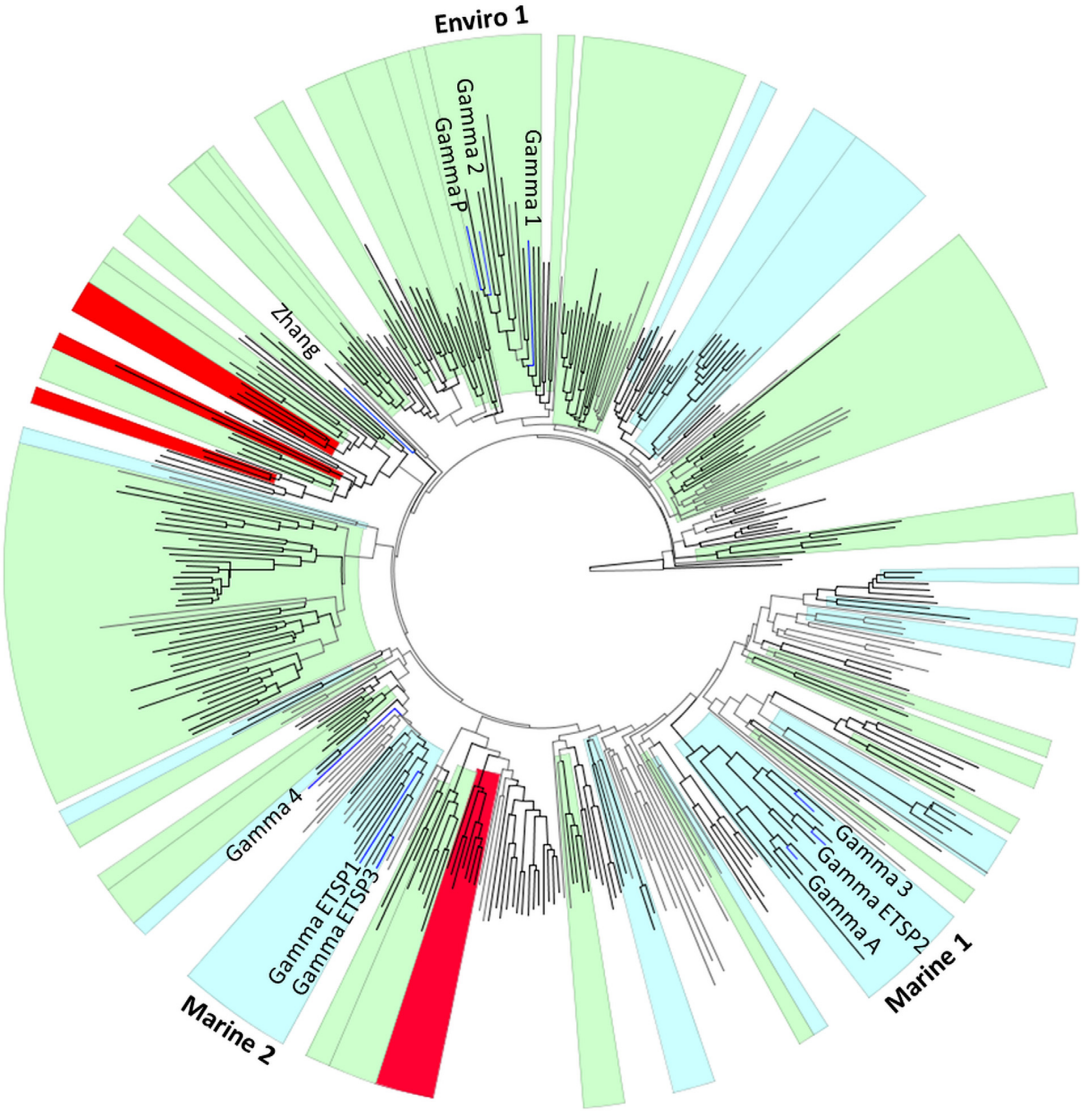
Maximum likelihood tree of γ-proteobacterial *nifH* OTUs. To improve visibility only OTUs ≥3 sequences are shown (400 of 1492 OTUs) and sequences names are not displayed (see S4 Fig for the sequence names). Clades of uncultured sequences from marine environments are shaded blue and from a variety of environments are shaded green. Clades containing several qPCR target sequences are labeled. Clades encompassing sequences isolated from PCR reagents are colored red. Probes used in qPCR studies are labeled and include Gamma 1, 2, 4, P, ETSP1, and ETSP3, and Zhang (S2 Fig). Tree was constructed using neighbor joining and RAxML methods. Significant branching (agreement between both algorithms and bootstrap >60) is colored black.

Several *nifH* diversity studies have reported γ-proteobacterial sequences; however each research group used different terminology, which makes it difficult to compare data across studies [13, 19, 25]. In addition different sets of sequences were used to construct phylogenetic trees, leading to conflicting conclusions about the phylogenetic grouping of phylotypes (for example Gamma 1 and Gamma 2 as α-proteobacteria in [25] versus γ-proteobacteria in [13]). In order to compare our data with published work, we conducted a large-scale phylogenetic analysis of γ-proteobacterial *nifH* sequences and identified similarities and differences in the various target sequences used to identify marine γ-proteobacteria. A search of the public databases revealed a large number of diverse γ–proteobacterial *nifH* sequences (6235 sequences), encompassing both cultured (90 unique OTUs) and uncultured representatives (1402 unique OTUs) (Fig 1). The uncultured γ–proteobacterial sequences were recovered from a variety of marine, aquatic, and terrestrial environments including soils, invertebrate guts, hot springs, hydrothermal vents, estuaries, and open oceans. Environmental (uncultured) sequences greatly outnumbered sequences from cultures forming 35 distinct clades, which often consisted entirely of environmental sequences distantly related to cultured organisms (Fig 1). This analysis shows that γ–proteobacteria are vastly underrepresented in culture collections and have been barely touched upon in environmental studies.

**Fig 2:**
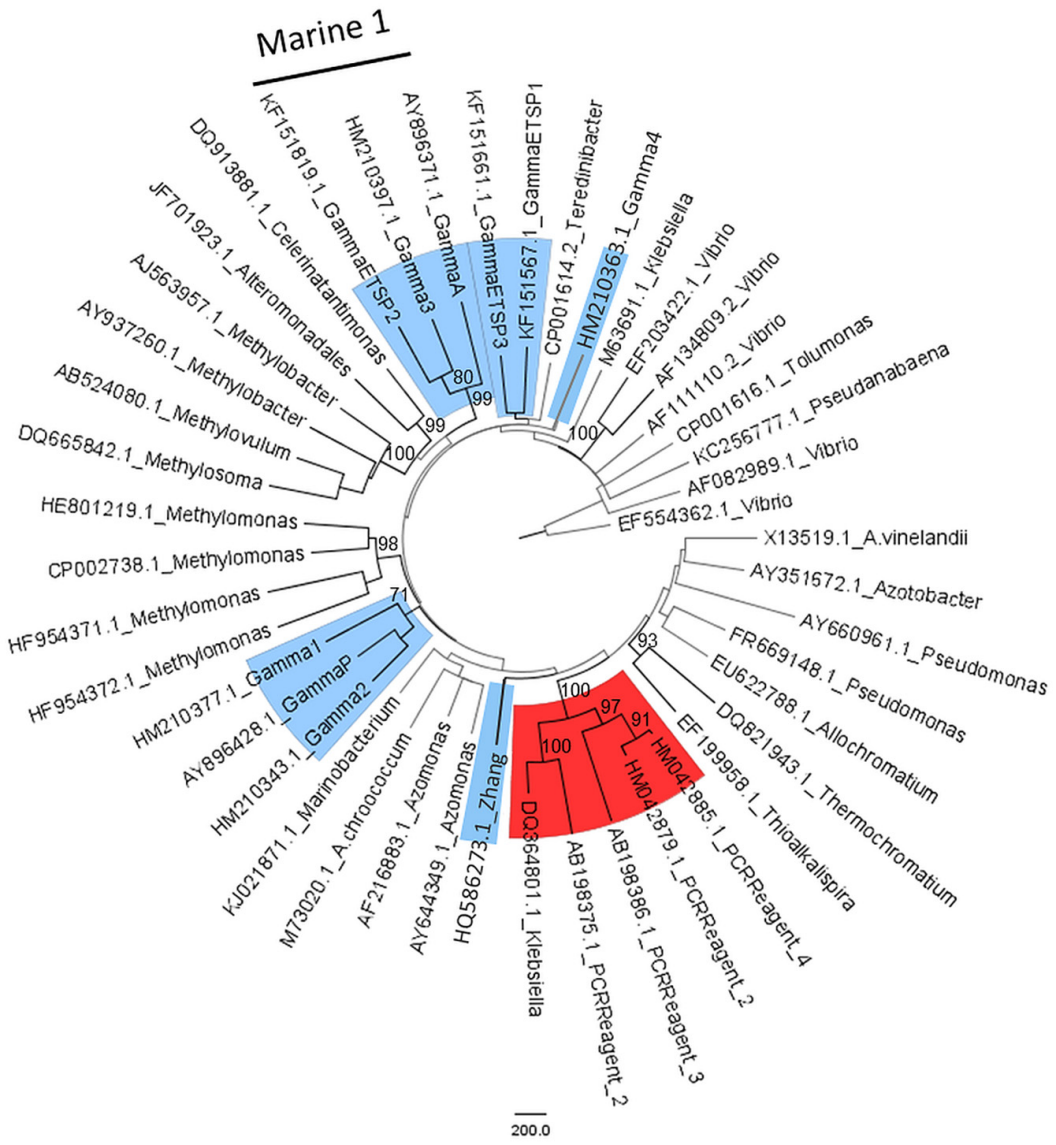
Maximum-likelihood tree of the nearest cultured neighbors to the γ-proteobacterial *nifH* OTUs targeted in marine qPCR studies. Sequences that have been used as targets for qPCR studies are colored blue and labeled as in the original publications: Gamma 1–4 from Halm et al. (2011) [13], Gamma A and P from Langlois et al. (2008) [18], ETSP1-3 from Turk-Kubo et al. 2014 [25], and Zhang et al. (2011) [26]. Tree was constructed using neighbor joining and RAxML methods. Significant branching (agreement between both algorithms and bootstrap >70) is colored black and bootstrap values are shown.

At least ten different uncultured γ–proteobacterial *nifH* sequences belonging to six separate clades have been used as targets in qPCR studies of marine environments [13, 18, 25–27]. These unique target sequences are all distantly related (<84% similarity) to clades containing sequences demonstrated to be contaminants in PCR reagents (Figs 1 and 2). The Gamma 4 (P8 in [28]) and Zhang sequences [13, 26] had the least similarity (<84 and 83%, respectively) to all other target sequences and formed two distantly-related, separate clades with environmental sequences from sediments, soil and estuaries. The remaining target sequences fell into one of three clades containing large numbers of environmental sequences. Clade Enviro 1 included Gamma 2, P and 1, as well as sequences from the open ocean, sediments, hot springs and seagrasses (Fig 1 and S4 Fig). The Gamma 2 and P sequences [13, 18] had 91% similarity. Gamma 1 had a higher similarity to (86% similar) to cultivated γ–proteobacteria than to Gamma 2 and P [13, 18] (85 and 83% similarity, respectively) (Figs 1 and 2). GammaETSP1 and GammaETSP3 grouped together (87% similarity) [25] with other sequences from marine environments in Clade Marine 2 (Fig 1). These sequences had a 90% similarity to *Teredinibacter*, a γ-proteobacterial symbiont found in the gills of shipworms [29]. Gamma A, 3, and ETSP2 clustered together in another clade of uncultured sequences from the open ocean [13, 18, 25], distantly related to cultivated organisms (<80% similarity, Figs 1 and 2). Bird et al. (2005) [15] called the Gamma A phylotype, unidentified marine bacteria (UMB). This phylotype is part of a much larger, well-conserved clade of uncultured marine bacteria, which we refer to as Marine 1.

**Fig 3:**
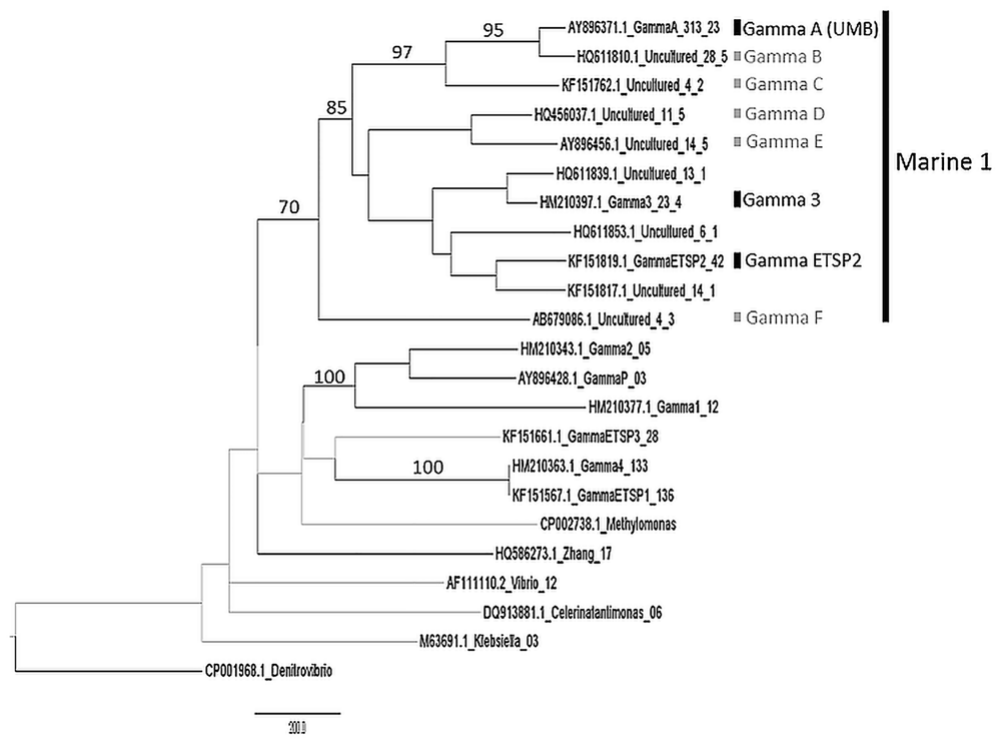
Maximum-likelihood tree of Marine 1 *nifH* OTUs. For visualization, only OTUs of ≥3 sequences are shown. Maximum likelihood and neighbor joining algorithms were compared. Bootstrap values above 70 are shown and significant branches (bootstrap >70 and agreement between both algorithms) are shown in black. Sequences are identified by accession number, first word in title, the number of sequences in the OTU, and then the number of studies where the sequence was found. For comparison purposes, sequences used as probes and the closest related organisms are included. The qPCR phylotype targets in the Marine 1 clade are labeled (black), as are potential phylotype targets (grey).

Using the *nifH* sequences analyzed in this study, the Marine 1 clade was comprised of 11 OTUs representing 474 sequences (Fig 3). Within this clade, Gamma A was by far the OTU with the largest number of representatives, containing 313 sequences originating from 23 independent studies. For comparison the next largest OTU, Gamma B represented by HQ611810.1, was comprised of 28 sequences from 5 studies. In addition to Gamma A, 3, and ETSP2, potentially five more *nifH* phylotypes in the Marine 1 clade would be good candidates for further study (Fig 3). These new phylotype targets were described in multiple studies; however they may not be as common as Gamma A based on the number of sequences forming the OTU.

Abundances of the Gamma A *nifH* phylotype were measured using TaqMan probes in several studies in the marine environment (e.g. [16, 18, 30]). Although three different primer-probe sets have been used so far to target Gamma A, sequence comparisons indicate that all probe and primer sets will target all Gamma A sequences while discriminating the other nine γ–proteobacterial *nifH* phylotypes (S2 Fig, Table 2). The Gamma A sequence (AY896371) was identical to 13 other sequences in our database. When the sequence identity was lowered to 99%, which corresponds to two mismatches in the *nifH* amplicon, the OTU contained 194 sequences, all recovered from the open ocean. The Gamma A OTU includes all three Gamma A primer-probe type sequences and five of the original ten UMB sequences described by Bird et al. (2005) [15]. Interestingly, the first reported and most commonly occurring Gamma A sequence in the NCBI database, AF059623 [2], has never been directly used to design a probe in either a qPCR or microarray study, but is targeted by the Gamma A qPCR probes and primers.

**Table 2:** Comparison of Gamma A primer and probe sequences to the other γ–proteobacterial *nifH* sequences used as targets in qPCR studies.

		Number of mismatches to Gamma A (AY896371.1)			
Sequence Type	Target Sequence	Forward	Reverse	Probe	Reference
Gamma A	AF059623.1	0	0	0	[2]
Gamma A	AY706889.1	1	0	0	[20]
Gamma A	EU052413.1	0	1	0	[16]
Gamma ETSP2	KF151819.1	6	5	2	[25]
Gamma 3	HM210397.1	4	4	4	[13]
Gamma ETSP3	KF151661.1	9	3	5	[25]
Gamma ETSP1	KF151567.1	7	3	4	[25]
Gamma 4	HM210363.1	5	5	1	[13]
Gamma P	AY896428.1	5	6	8	[18]
Gamma 1	HM210377.1	5	3	6	[13]
Gamma 2	HM210343.1	8	4	4	[13]
Zhang	HQ586273.1	3	3	6	[11]

### Distribution of Gamma A *nifH* phylotypes and transcripts

**Fig 4:**
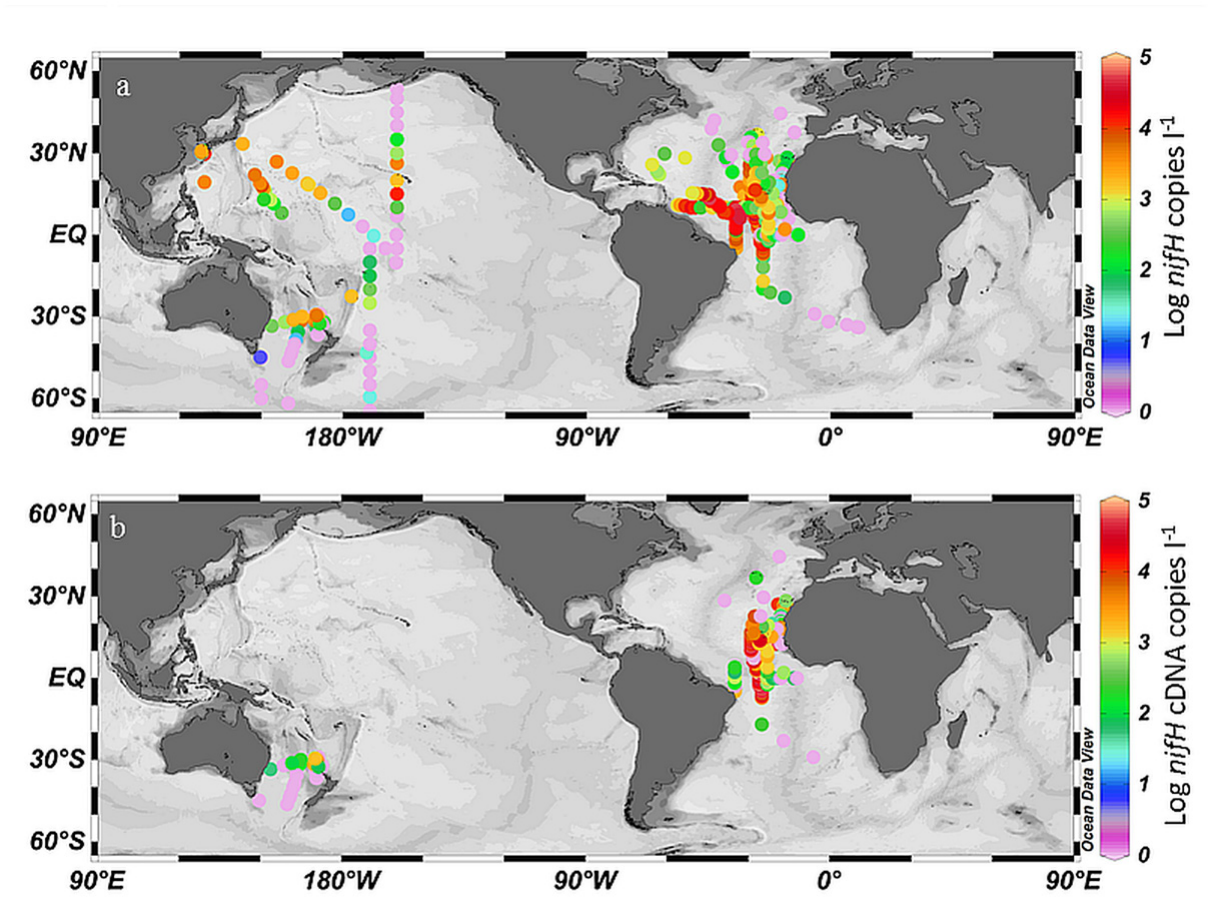
Surface abundances of Gamma A *nifH* DNA (a) and cDNA (b) estimated by TaqMan qPCR. Abundances are presented as log (*nifH* copies l^-1^) where red colors indicate high abundances and purple near detection limit abundances.

Abundances of Gamma A *nifH* phylotypes were estimated in 992 samples using TaqMan qPCR (Fig 4a). The samples were collected at 494 stations from 15 cruises between 2000 and 2010 (Table 1). Depths from the surface to 500 m were sampled, but over half (536 samples) were collected at depths shallower than 20 m. Gamma A *nifH* DNA was detected and quantifiable in 67% of the samples and abundances ranged from the detection limit to 8 x 10^4^ *nifH* copies l^-1^. The highest Gamma A *nifH* abundances were detected in the tropical Atlantic Ocean between 28°N and 10°S, but were also elevated in the sub-tropical North Pacific Ocean between 10–20°N. It should be noted that only 194 samples were collected in the Pacific Ocean compared to 798 samples in the Atlantic Ocean. Gamma A *nifH* transcript abundance was estimated in parallel in 673 samples and was detected in 64% of the samples (Fig 4b). Gamma A transcript abundances were correlated with DNA abundances, but were usually higher and ranged from the detection limit to 4.7 x 10^5^ *nifH* copies l^-1^. The highest transcript abundances were detected in the tropical Atlantic Ocean between 20°N and 10°S.

In addition to our study sites, Gamma A sequences (sequences with similarity greater than 98% to Gamma A) have also been reported in a variety of marine ecosystems including the oligotrophic Mediterranean [31], Red [32], and Arabian Seas [15], the edge of the Mekong River Plume [17], South China Sea [16], Indian Ocean [27], South Pacific [19], and Southern California Bight [33]. Gamma A was found at all stations throughout the upper water column in the Arabian Sea [15] and at low abundances in the Red Sea [32]. In the Indian and South Pacific Oceans, South China Sea during inter-monsoon, and oceanic stations in the Mekong River Plume, abundances were similar to those reported in the present study [16, 17, 19, 27]. Gamma A appears to be a cosmopolitan diazotroph more commonly found in tropical and sub-tropical, oligotrophic regions.

**Fig 5:**
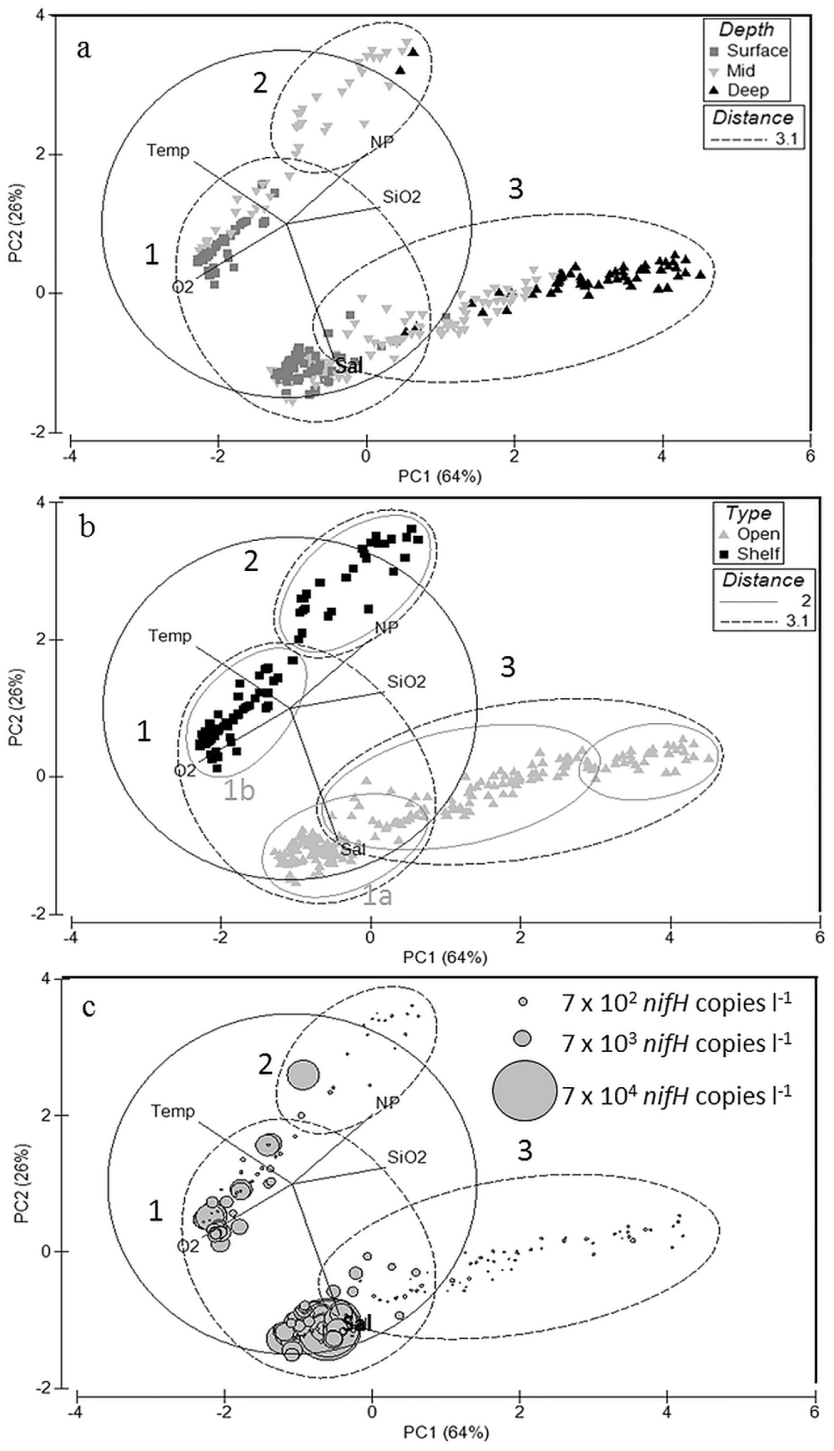
Principal components analysis of environmental parameters for samples where Gamma A *nifH* abundances were estimated. The cluster analysis distance of 3.1 is shown in panels ‘a-c’ and 2 in ‘b’. Samples are identified by collection depth (surface- <20 m, mid- 20–100 m, deep- >100 m) in ‘a’ and distance to the continental margin (coastal- <1000 km or open- >1000 km) in ‘b’. The Gamma A abundances (*nifH* copies l^-1^) are displayed in ‘c’.

A multivariate analysis of the samples supports the conclusion that the Gamma A phylotype has a global distribution throughout tropical to sub-tropical surface oceans. A PCA of the environmental parameters showed that the samples grouped significantly with respect to depth and distance from the continental margin (ANOSIM, P < 0.01; Fig 5a and 5b). A resemblance matrix of the data was constructed to further compare similarities between the samples and a hierarchical cluster analysis revealed three clusters (SIMPROF, Fig 5). Gamma A abundances were significantly higher in Cluster 1. The average dissolved nutrient concentration of Cluster 1 samples was significantly lower than Cluster 2 and 3 samples (ANOVA, P < 0.01, S2 Table). In contrast, the average Cluster 1 oxygen concentration (194+/- 1.6 μM) was nearly double that of Cluster 2 and 3 (100+/-9.7 and 109+/-4.5 μM). Cluster 1 samples were collected at depths shallower than 100 m (average 26 +/- 1.6) and contained the highest concentrations of Gamma A *nifH* DNA with an average of 3400 +/- 500 *nifH* copies l^-1^. At a shorter cluster distance of 2, cluster 1 separates into two clusters, one containing open ocean samples (1a) and the second containing shelf samples (1b, Fig 5b). Gamma A abundances were significantly higher in open ocean samples (9000 +/- 800 *nifH* copies l^-1^) than in samples collected closer to the coast (2700 +/- 300 *nifH* copies l^-1^; T-test, P = 0.005).

In addition to dissolved nutrient and oxygen concentrations, Cluster 2 and 3 differed from Cluster 1 with respect to depth, temperature, and salinity. Cluster 2 (coastal shelf) and 3 (open ocean) samples were collected at average depths of 66 +/- 5.3 and 177 +/-14 m, respectively. Samples in Cluster 2 had the highest average temperature (36 +/- 0.06°C), but the lowest average salinity (18 +/- 0.4 psu), indicating a freshwater influence. Cluster 3 samples had the highest average salinity (36 +/- 0.04 psu), but the lowest average temperature (16 +/- 0.4°C) of the three clusters. The environmental conditions in Cluster 2 and 3 samples were perhaps not as favorable to Gamma A as in Cluster 1; Gamma A abundance greater than 10^2^ *nifH* copies l^-1^ was detected in only one sample in clusters 2 and 3 (Fig 5c). Average abundances of samples in clusters 2 and 3 were only 580 +/- 500 and 100 +/- 30 *nifH* copies l^-1^, respectively. Although not all types of marine environments have been tested for the presence of Gamma A, this data set shows the highest Gamma A abundances were detected in warm, well-oxygenated, oligotrophic waters, indicating a preference for tropical or sub-tropical oceans.

### Environmental Niche for Gamma A

**Fig 6:**
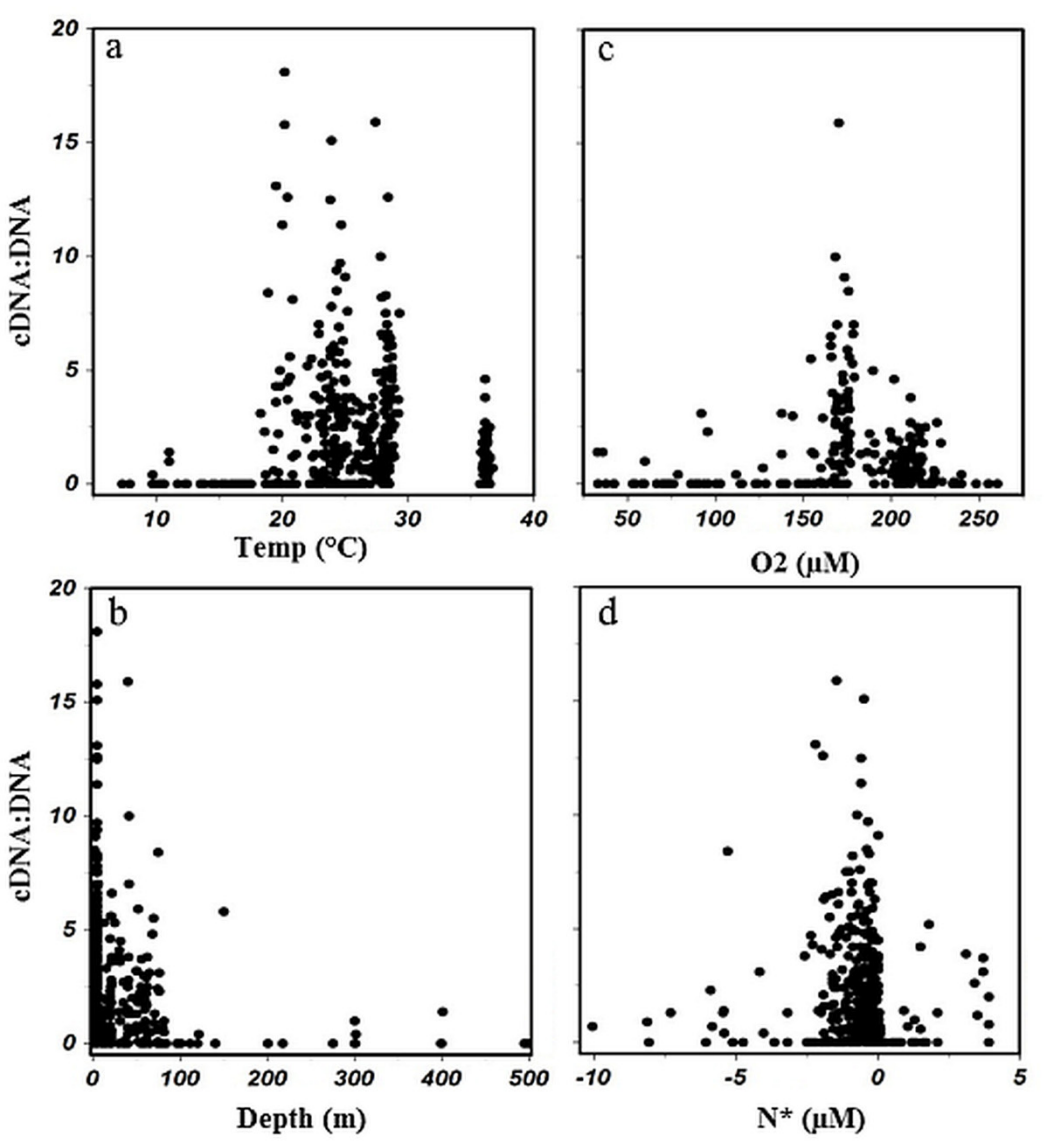
Activity ratio (cDNA:DNA) where Gamma A *nifH* cDNA and DNA were detected by TaqMan qPCR as a function of measured environmental parameters. The cDNA:DNA ratio is graphed versus temperature (°C, a), depth (m, b), O_2_ concentration (μM, c) and N* (μM, d). The cDNA:DNA ratio was calculated only for samples in which both Gamma A DNA and cDNA abundances were >80 *nifH* copies l^-1^. Please note that not all parameters were available for all samples.

Patterns of *nifH* transcripts normalized to *nifH* DNA abundances were observed when compared with environmental parameters (Fig 6 and S3 Fig). Gamma A *nifH* cDNA:DNA was highest in waters with temperatures ranging from 20–30°C (Fig 6a) and at depths of 100 m and shallower (Fig 6b, S2 and S3 Tables). In the surface and upper water column samples, nutrient concentrations were low to undetectable (S2 Fig). For samples where nitrate and phosphate concentrations were measurable, N* was calculated (NO3 - 16PO4 [34]). Elevated Gamma A *nifH* cDNA:DNA was observed at N* concentrations from -5 to 5 μM, however the highest cDNA:DNA ratios (>5) were from samples where N* concentrations were between -2.5 to 0 μM (Fig 6d). It should be noted, though, that a large number of samples, particularly cDNA samples, were collected in the tropical oceans (S4 Table). Despite this, research conducted in geographic areas separate from our study support these conclusions. Moisander et al. (2008) also observed Gamma A only in the upper, well-lit and oxygenated water column [16]. In the Indian Ocean, Shiozaki et al. (2014) found the highest abundances of Gamma A in surface ocean waters [27]. Gamma A abundances in the South Pacific correlated positively with temperature and oxygen and negatively with nutrients and depth [19]. Several studies have looked at *nifH* diversity in oxygen-minimum and-depleted zones and reported γ-proteobacterial sequences such as Gamma 4, ETSP1, or ETSP3 but no sequences similar to Gamma A or any other Marine 1 sequences were present in those clone libraries [35–37]. No Gamma A was found in clone libraries from samples collected world-wide at depths of 500–5900m [38]. The Gamma A *nifH* phylotype abundance and expression are associated with warm, oligotrophic, oxygenated surface waters that have an N deficit (i.e. the tropical and sub-tropical oligotrophic oceans).

Nitrogenase activity is sensitive to oxygen and thus the presence of the Gamma A phylotype and expression of *nifH* in oxygenated waters is intriguing. Strictly heterotrophic bacteria do not rely on sunlight for energy; however their presence in the oxygenated surface waters may reflect other metabolic preferences, such as an ample supply of organic carbon. In addition, Gamma A could be capable of photoheterotrophy, a metabolic pathway that uses the energy from sunlight to activate a proton gradient which then augments the organism’s ability to take in organic carbon [39, 40]. Photoheterotrophy may be used to offset the energy requirements for N_2_ fixation [41]and would explain why this diazotroph is found in well-lit, oxygenated waters. An alternative explanation for the observed higher abundances of Gamma A in the upper water column may be the availability of labile dissolved organic carbon (DOC) that can fuel heterotrophy. DOC is produced in tropical-subtropical oceans by autotrophs and can accumulate in surface waters, where the highest concentrations are measured, due to vertical stratification [42].

Like many questions concerning the physiology of Gamma A, how this diazotroph reconciles the incompatible high oxygen levels with anaerobic N_2_ fixation still remains to be elucidated. Diazotrophs living in oxygenated environments have developed many ways to protect the nitrogenase enzyme, ranging from specialized cell structures, called heterocysts and diazocytes, to temporal separation of oxygen producing processes (such as photosynthesis) and nitrogenase activity [43]. Gamma A may reconcile biological N_2_ fixation and inhabiting an aerobic environment by living in association with other organism(s), as exemplified by *Candidatus Atelocyanobacterium thalassa* or *Richelia sp*. [44, 45], where the host could provide a carbon source and possibly protect the symbiont from harmful oxygen concentrations in return for fixed nitrogen. If this strategy is used, the light requirements of a putative photosynthetic host may explain the observed depth distribution of Gamma A. Alternatively, Gamma A could avoid high oxygen concentrations by living in association with particles, which have been shown to provide diazotrophs with low oxygen environments and high organic carbon [46, 47].

Though we have reported a large geographic coverage of Gamma A DNA and cDNA abundances, *nifH* abundance data cannot be converted into N_2_ fixation rates. Studies that have measured both γ-proteobacterial phylotype abundances and N_2_ fixation rates have reached opposing conclusions about the N_2_ fixing potential of heterotrophic diazotrophs. Halm et al. (2011) attributed much of the N_2_ fixation they measured to the dominant γ-proteobacterial phylotypes they detected [13]. Conversely, Turk-Kubo et al. (2014) concluded that the abundances of γ-proteobacteria that they detected were too low to account for the N_2_ fixation rates they measured [25]. The Gamma A abundances detected in our study were high enough to produce measurable N_2_ fixation rates, according to the calculations in Turk-Kubo et al. (2014) [25]. However, a more thorough assessment of their importance in the ocean will require cell specific N_2_ fixation rates for open ocean γ-proteobacterial diazotrophs. Presently, it is unclear how much of the global N_2_ fixation can be attributed to heterotrophic diazotrophs.

## Conclusions

Heterotrophic bacteria are prevalent throughout oligotrophic oceans [48] and γ–proteobacteria are an abundant constituent of this community [8, 49]. Following cyanobacterial *nifH* phylotypes, γ-proteobacterial phylotypes were the next most abundant diazotrophic phylotype in the Atlantic Ocean [18]. Although non-cyanobacterial *nifH* genes can be more abundant and appear to have a larger distribution than cyanobacterial *nifH* genes [4, 25], they have received very little attention until recently [41]. This study presents extensive data on the abundance and expression of Gamma A, a putatively heterotrophic diazotroph. Our work and that of others [19, 27] indicate that the Gamma A phylotype is a globally distributed and active diazotroph based on the wide distribution of Gamma A *nifH* DNA and cDNA throughout the tropical, oligotrophic surface oceans. It is not yet clear how much of an impact this diazotroph has on local and global nitrogen- and carbon-cycles, but it is clear that this organism warrants more attention.

## Supporting Information

S1 Alignment Alignment of *nifH* sequences used in Fig 1. (PDF)

S2 Alignment Alignment of *nifH* sequences used in Fig 2. (PDF)

S3 Alignment Alignment of *nifH* sequences used in Fig 3. (PDF)

S1 Fig Phylogenetic tree of *nifH* from cultured organisms in relation with *nifH* OTU’s from environmental sequences used as qPCR target phylotypes and *nifH* contaminants of PCR Reagents. The tree was constructed using the neighbor joining method. Clades have been collapsed taxonomically by class according to the given color scheme. The number of sequences comprising each clade is shown. Clades that are not collapsed contained sequences originating from multiple taxonomic classes. The clades containing the qPCR phylotype targets are labeled. Clades containing sequences originating from PCR reagents are colored red. Sequences from all clades identified as γ-proteobacteria were used in further analyses.
(TIF)

S2 Fig Dot plot alignment of γ–proteobacterial *nifH* amplicons used as targets in qPCR studies. The full Gamma A amplicon is shown at the top. The amplicons used in other studies follow, identified by either the first author’s name or probe name and sequence accession number in brackets. The first six sequences (bold font) are in the Marine 1 clade. The Gamma A primers (light grey bars) and probes (dark grey bars) used in qPCR studies are shown.
(TIF)

S3 Fig Plot of Gamma A cDNA:DNA versus NO_3_ (μM, a), PO_4_ (μM, b), and SiO_2_ (μM, c) concentrations. Note that the x-axis scale changes in each panel.
(TIF)

S4 Fig Linear maximum likelihood tree of γ-proteobacterial *nifH* OTUs. This tree is a reproduction of Fig 1, including the accession numbers, first word in title, the number of sequences in the OTU, and isolation environmental source (if available).
(PDF)

S1 Table List of accession numbers for sequences identified as γ-proteobacteria. (PDF)

S2 Table Descriptive statistics summary of PCA clusters. The sample size of each cluster is given (n). The calculated mean, and standard error (SE), and range of values measured for each parameter are given. Means in bold font are statistically different (ANOVA, P < 0.01).
(PDF)

S3 Table Detection Frequency of Gamma A in qPCR Analysis. The number of samples where Gamma A abundances (*nifH* copies l^-1^) were detected per each depth range (m) are shown. The number of samples per depth range (n) is given and the detection frequency (%) was calculated.
(PDF)

S4 Table Sampling frequency arranged by latitudinal bands. The number of DNA and cDNA samples collected in each latitudinal band (°N) is provided.
(PDF)

S5 Table Designation of sequences in the Marine 1 clade into OTUs at 100% similarity. Representative sequences in Fig 3 are in bold font.
(PDF)

S6 Table Designation of sequences in the Marine 1 clade into OTUs at 99% similarity. Representative sequences in Fig 3 are in bold font.
(PDF)

S7 Table Designation of sequences in the Marine 1 clade into OTUs at 97% similarity. Representative sequences in Fig 3 are in bold font.
(PDF)
