# Supplementary material for: Widespread Distribution and Expression of Gamma A (UMB), an Uncultured, Diazotrophic, γ-Proteobacterial nifH Phylotype
Source: PLoS One. 2015 Jun 23;10(6):e0128912. doi: 10.1371/journal.pone.0128912 (PMC4477881; doi:10.1371/journal.pone.0128912)
Supplement: S1 Alignment — (PDF) [file pone.0128912.s001.pdf]

>EU594060.1_Uncultured_marine sponge_5

TCCACCCGTCTGATGCTTCACAGTAAAGCTCAAACCACCATTCTTCAC------CTG---
GCAGCTGAGCGCGGTGCGGTAGAAGACCTGGAA---
CTCGAAGAAGTGCTCCTGACTGGCTACAAAAACGTTAAGTGCGTTGAATCCGGTGGTCCTGAGCCCGGCGTGGGC
TGCGCTGGACGGGGCATCATCACC---GCCATTAACTTCCTGGAAGAGGAAGGTGCTTAC------GAAGATCTA---
GAT---------TTTGTTTCTTACGACGTATTAGGCGACGTTGTTTGCGGTGGTTTTGCCATGCCTATCCGGGAAGGT---
AAAGCCCAGGAAATCTACATCGTTACCTCT

>GU593984.1_Uncultured_freshwater_21

TCCACTCGCCTCATGCTTCACAGCAAAGCCCAAACCACTATCTTGCAC------TTA---
GCTGCCGAGCGTGGTGCAGTGGAAGATCTCGAA---
CTGGAAGAAGTATTACTCACGGGCTATCGCGGCGTGAAGTGCGTAGAGTCCGGTGGTCCTGAACCCGGTGTAGGT
TGCGCCGGTCGTGGAATTATCACT---GCCATTAACTTCCTGGAAGAAGAAGGGGCTTAC------GAAGATCTC---GAT-
--------TTCGTCTCCTACGATGTTTTAGGGGACGTGGTATGCGGTGGCTTTGCCATGCCTATTCGGGAAGGT---
AATGCTCAGGAAATCTACATTGT-------

>AF227927.1_Phormidium

TCCACCCGCCTGATGCTGCACAGCAAAGCTCAAACCTCTGTACTGCAG------CTG---
GCCGCTGAACTTGGTGCTGTTGAAGATGTTGAA---
CTTGACCAGGTGCTGCAAATCGGCTATCGCGGCGTTAAGTGCGTTGAGTCGGGCGGTCCTGAGCCTGGCGTAGGC
TGTGCCGGTCGCGGCATCATCACC---GCCATCAACTTCCTGGAAGAAGAAGGCGCTTAC------GAAGACCTC---GAT-
--------TTCGTTTCCTATGACGTATTGGGCGACGTAGTTTGCGGCGGTTTCGCCATGCCCATTCGGGAAGGC---
AAAGCCCAGGAAATCTACATCGTTTGCTC-

>JF896962.1_Uncultured_marine mat_3

--GTTGAAGATGTCGAA---
CTTGAGCAAGTGCTGCAAATTGGCTATCGTGGCGTTAAGTGTGTAGAGTCCGGTGGCCCAGAACCCGGTGTGGGC
TGTGCGGGTCGGGGTATTATTACC---GCCATCAACTTCCTAGAAGAAGAAGGCGCTTAC---ACC---GATCTA---GAT-
--------TTCGTTTCCTACGATGTGCTAGGCGATGTGGTTTGCGGTGGTTTTGCAATGCCCATTCGGGAAGGC---
AAAGCTCAGGAAATCTACATTGT-------

>GQ426268.1_Uncultured_hot spring_3

---ACCCGCCTGATTCTGCATCAAAAGTGCCAGGACACCATCCTGTCG------CTG---
GCAGCTGAGGCCGGTTCCGTGGAAGATCTCGAG---
CTGGAAGATGTAATGAAAGTCGGTTTCAAAGACATTCGTTGCGTTGAATCTGGTGGTCCAGAGCCTGGTGTAGGTT
GCGCCGGTCGCGGTGTGATTACT---TCCATTAACTTCCTGGAAGAAGAAGGTGCCTAC------GAAGGTATC---GAT---
------TACGTCTCCTACGATGTATTGGGTGACGTGGTGTGTGGCGGTTTCGCAATGCCGATTCGCGAGAAC---
AAGGCGCAGGAAATCTACATCGTT------

>EF174676.1_Uncultured_marine_4

TCCACGCGCCTGATCCTGAACTCCAAAGCACAGGACACCGTGCTGCAC------CTG---
GCCGCTGAGATGGGCTCGGTTGAGGATCTGGAA---
CTCGAAGATGTTCTGAAGATTGGTTACTCGGACATCAAATGCGTTGAATCCGGTGGTCCGGAGCCAGGGGTTGGC
TGCGCGGGCCGTGGTGTTATCACC---TCGATCAACTTCCTCGAAGAAAACGGTGCCTAT------GACGATGTC---GAT-
--------TACGTCTCCTATGACGTTCTTGGCGACGTTGTGTGCGGTGGCTTTGCGATGCCGATCCGCGAAAAC---
AAGGCGCAGGAAATCTACATCGTT------

>HM801361.1_Uncultured_marine_5

TCAACACGTTTGATTCTGCACGCAAAAGCACAGGACACCATCCTGTCA------TTG---
GCTGCTGAAGCTGGTTCCGTGGAAGATCTGGAA---
CTCGAAGACGTGATGAAAGTTGGCTATCGTGACATCCGTTGCGTAGAGTCAGGTGGCCCAGAGCCAGGCGTTGGC
TGTGCTGGTCGCGGTGTGATCACA---TCAATCAACTTCCTCGAAGAAAACGGCGCATAC------GAAGGCGTT---GAT-
--------TATGTTTCTTACGACGTATTGGGTGACGTGGTTTGCGGCGGTTTCGCTATGCCAATCCGTGAGAAC---
AAAGCGCAAGGAATTTACATCGTT------

>AY159592.1_Uncultured_root_25

TCCACCCGGCTGATCCTGAACGCCAAAGCGCAGGACACGGTTCTGCAT------CTG---
GCAGCGCAGGAAGGTTCGGTGGAAGACCTTGAG---
CTCGAGGACGTGCTCAAGGCCGGCTACAAAGGCATCAAGTGCGTGGAGTCCGGCGGTCCGGAACCGGGCGTCGG
CTGCGCCGGGCGCGGCGTCATCACC---TCGATCAATTTCCTTGAAGAGAACGGTGCATAT------GACGATGTC---
GAC---------TACGTCTCCTATGACGTGCTCGGCGATGTGGTGTGCGGTGGCTTTGCGATGCCGATCCGTGAGAAC---
AAGGCCCAGGAGATCTACATCGT-------

>AB198366.1_PCRreagent_7

TCCACGCGCCTCATCCTGCACGCCAAGGCTCAGGACACCATCCTCAGC------CTC---
GCCGCCGAGCAGGGCAGCGTCGAGGACCTCGAA---
CTCGAAGACGTAATGAAGATCGGCTACCAAAACATCCGTTGTGTGGAATCCGGCGGTCCGGAGCCGGGCGTCGGC
TGCGCTGGCCGCGGCGTCATCACC---TCGATCAACTTCCTCGAGGAAAACGGCGCCTAC------GAGGACATC---
GAC---------TACGTCTCCTACGACGTGCTGGGC--

>HE803322.1_Uncultured_culture_4

TCAACTCGTCTCATCCTGCACGCCAAAGCGCAGGACACCATCTTGAGC------CTC---
GCGGCCGCCCAGGGCAGCGTCGAGGATCTTGAG---
CTCGAAGACGTGATGAAGATCGGCTATGCCGGAATCAAATGCGTCGAGTCCGGAGGTCCGGAGCCCGGCGTTGGT
TGTGCGGGTCGCGGCGTCATCACC---TCGATCAACTTCCTCGAGGAAAACGGCGCTTAC------GAAGACATC---GAC-
--------TACGTTTCCTATGACGTGCTCGGTGACGTCGTGTGCGGCGGTTTCGCGATGCCGATACGTGAAAAT---
AAGGCGCAGGAAATCTACATCGT-------

>HQ335640.1_Uncultured_soil_3

TCTACCCGCCTCATCCTGCATGCAAAGGCACAGGACACCGTGCTGTCG------CTG---
GCAGCAGAAGCTGGCTCGGTGGAAGACCTCGAG---
ATTGAAGACGTGCTCAAGGTCGGCTATCGCGACATCCGCTGCGTTGAGTCTGGCGGTCCTGAGCCCGGTGTGGGT
TGTGCCGGTCGCGGCGTGATCACA---TCGATCAACTTCCTCGAGGAGAACGGCGCTTAC------GACGGCGTG---
GAC---------TATGTTTCCTATGACGTTTTGGGCGACGTGGTGTGCGGCGGATTTGCGATGCCGATTCGCGAGAAC---
AAGGCTCAGGAAATCTACATCGT-------

>HQ335648.1_Uncultured_soil_2

TCTACCCGCCTGATCCTGCATGCAAAAGCACAGGACACCGTGCTGTCG------CTG---
GCAGCAGAAGCCGGCTCGGTAGAAGACCTCGAG---
ATTGAAGATGTGCTCAAGGTCGGCTATCGCGACATCCGCTGCGTTGAGTCCGGCGGGCCGGAGCCCGGTGTAGGT
TGTGCCGGTCGCGGCGTCATCACT---TCGATCAACTTCCTCGAAGAGAACGGCGCTTAC------GACGGTGTG---GAC-
--------TATGTTTCCTATGACGTACTGGGCGACGTGGTATGCGGCGGATTCGCGATGCCGATTCGCGAAAAC---
AAGGCACAGGAAATCTACATCGT-------

>AF484669.1_Methylococcus_2

TCCACCCGTCTGATCCTGCACGCAAAGGCCCAGGACACCATCCTGTCC------CTG---
GCCGCCGATGCCGGCAGCGTCGAGGACCTGGAG---
CTGGAAGACGTCATGAAGGTCGGCTTCCGCGACATCCGTTGCGTGGAATCCGGCGGTCCGGAACCGGGCGTCGGC
TGCGCCGGCCGGGGTGTGATCACC---TCGATCAACTTCCTGGAAGAGGAAGGCGCTTAT------GACGGCATC---
GAC---------TACGTCTCCTACGACGTGCTCGGCGACGTGGTCTGCGGCGGTTTCGCCATGCCCATCCGTGAGAAC---
AAGGCACAGGAAATCTACATCGT-------

>EU331502.1_Uncultured_soil_4

TCCACCCGGCTGATCCTGCACGCAAAAGCCCAGGACACCATCCTGTCG------CTG---
GCCGCTGCGGCCGGCACGGTGGAAGACCTCGAG---
CTCGATGAAGTGATGAAGATCGGCTACCGCGACATCCGCTGCGTGGAGTCCGGCGGCCCCGAGCCGGGCGTCGG
CTGCGCCGGTCGCGGTGTGATCACC---TCGATCAACTTCCTGGAAGAGGAAGGCGCCTAC------GAGGACCTC---
GAC---------TACGTGTCCTACGACGTGCTCGGGGATGTGGTGTGCGGCGGCTTCGCCATGCCCATCCGCGAGAAC---
AAGGCGCAGGAAATCTACATCGT-------

>HQ586513.1_Uncultured_South China Sea_2

TCCACCCGCCTGATCCTGAACTCCAAGGCGCAGGACACGGTGCTGCAC------CTC---
GCGGCCGAGATGGGCTCCGTCGAGGATCTCGAG---
CTGGAAGACGTGCTGAAGACCGGCTTCAAGGACATCAAGTGCGTCGAGTCCGGCGGTCCCGAGCCGGGCGTCGG
CTGCGCCGGCCGCGGCGTCATCACC---GCCATCAACTTCCTCGAGGAGAACGGCGCCTAC------GATGACGTA---
GAT---------TACGTGTCCTACGACGTTCTGGGCGACGTGGTCTGTGGCGGCTTCGCCATGCCGATCCGCGAGAAC---
AAGGCACAGGAAATCTACATCGT-------

>DQ776446.1_Uncultured_terrestrial rhizosphere_2

TCCACACGTCTCATCCTGCATGCCAAGGCCCAGGACACCATCCTGTCG------CTG---
GCCGCTGAAGCCGGTTCCGTCGAGGATCTGGAA---
CTGGAAGACGTCATGAAGGTGGGATATCGCGACATCCGTTGCGTTGAATCCGGTGGTCCAGAGCCAGGTGTTGGT
TGTGCTAGTCGTGGCGTGATTACT---TCGATCAACTTCCTCGAAGAAAACGGCGCTTAC------GACGATATG---GAC-
--------TACGTTTCCTACGACGTTCTGGGTGACGTGGTGTGCGGCGGCTTTGCAATGCCGATCCGTGAAAAC---
AAGGCACAGGAAATCTACATCGT-------

>HQ335876.1_Uncultured_soil_11

TCGACTCGTCTGATTCTGCATGCAAAGGCACAGGACACCGTTCTGTCG------CTG---
GCCGCAGAAGCCGGTTCCGTGGAGGATCTGGAA---
CTGGAAGACGTGATGAAGATCGGTTATCGCGACATCCGTTGCGTTGAGTCCGGTGGTCCTGAGCCAGGTGTGGGT
TGCGCTGGCCGTGGTGTGATTACT---TCGATCAACTTCCTCGAAGAGAACGGCGCATAC------GACGGCGCT---
GAC---------TACGTCTCCTACGACGTGCTGGGCGACGTGGTGTGCGGCGGCTTCGCCATGCCGATTCGCGAAAAC---
AAGGCGCAGGAAATCTACATCGT-------

>AY225105.1_PCRreagent

TCGACCCGCCTGATCCTGCACGCCAAGGCCCAGGACACCATCCTGTCG------CTG---
GCGGCCGAAGCCGGCTCGGTGGAGGACCTGGAG---
CTCGAGGACGTGATGAAGATCGGCTACGAGGACATCCGTTGCGTCGAATCCGGTGGCCCGGAGCCCGGAATGGG
CTGCGCCGGCCGCGGCGTGATCACC---TCGATCAACTTCCTTGAAGAAAACGGCGCCTAC------GACGGTGTT---
GAC---------TACGTCTCTTACGACGTGCTGGGCGACGTGGTGTGCGGCGGCTTCGCCATGCCCATCCGCGAAAAC---
AAGGCGCAAGAGATCTACATCGTCATGTCC

>KF872848.1_Uncultured_root_4

TCGACCCGCCTGATCCTGCACGCCAAGGCGCAGGACACCATCCTCTCG------CTG---
GCTGCGGAAGCGGGCTCGGTGGAGGATCTGGAG---
CTTGAAGACGTCATGAAGATCGGCTATCGCGACATCCGCTGCGTCGAGTCCGGCGGTCCCGAGCCAGGGGTTGGC
TGCGCGGGCCGTGGTGTGATCACC---TCGATCAACTTCCTGGAAGAAAACGGCGCTTAC------GACGGCGTG---
GAC---------TATGTGTCCTACGACGTGCTGGGCGACGTGGTGTGCGGCGGCTTTGCCATGCCCATCCGCGAAAAC---
AAGGCGCAGGAAATCTACATCGT-------

>AY225106.1_PCRreagent_8

TCGACCCGCCTGATCTTGCACGCGAAGGCTCAGGACACCATCTTGTCG------CTG---
GCCGCTGAAGCTGGTTCGGTGGAGGACCTCGAA---
CTGGAAGACGTGATGAAGGTCGGGTACCGCGACATCCGTTGCGTGGAATCCGGCGGCCCTGAGCCTGGGGTTGG
CTGCGCCGGCCGCGGCGTGATCACT---TCGATCAACTTCCTGGAAGAAAACGGCGCCTAC------GAAGGCGTG---
GAC---------TATGTGTCCTACGACGTGCTGGGCGACGTGGTGTGCGGTGGCTTTGCCATGCCCATCCGTGAGAAC---
AAGGCACAGGAAATCTACATCGTCATGTCC

>AB198390.1_PCRreagent_34

TCCACCCGCCTGATCCTGCACGCAAAGGCTCAGGACACCATCCTGTCG------CTG---
GCCGCTGAAGCCGGTTCGGTGGAAGACCTCGAG---
ATCGATGATGTGATGAAGGTGGGCTATCGCGACATCCGTTGCGTGGAGTCCGGTGGTCCTGAGCCCGGCGTGGGC
TGTGCCGGCCGTGGCGTGATCACC---TCGATCAACTTCCTGGAAGAAAACGGTGCCTAC------AAAGGCGTG---
GAC---------TATGTGTCCTACGACGTGCTGGGCGACGTGGTGTGCGGTGGCTTTGCCATGCCCATCCGTGAGAAT---
AAAGCCCAGGAAATCTACATCGT-------

>AF378720.1_Methylosinus_2

TCGACCCGTCTGATCCTGCACGCCAAGGCTCAGGACACCATCCTCTCC------CTG---
GCCGCCGAGGCCAGTTCGGTCGAGGATCTCGAG---
CTCGAAGACGTCATGAAGGTCGGCTTCGAAGACATTCGTTGCGTCGAGTCCGGTGGTCCGGAGCCGGGAGTTGGC
TGCGCCGGCCGTGGCGTTATCACC---TCGATCAACTTCCTCGAGGAGCAGGGCGCTTAC------GACGGCGTC---
GAC---------TACGTTTCTTATGACGTGCTCGGCGACGTGGTCTGCGGCGGCTTTGCGATGCCGATCCGTGAGAAC---
AAGGCTCAGGAGATCTACATCGT-------

>KF847333.1_Uncultured_soil_4

TCGACCCGCCTGATCCTGCACGCCAAGGCCCAGGACACCGTGCTGTCG------CTG---
GCTGCCGAAGCCGGCTCGGTCGAGGATCTGGAA---
CTCGAGGACGTGCTCAAGGTCGGCTACCGCGACATCCGCTGCGTCGAGTCCGGCGGTCCGGAACCCGGCGTCGGC
TGTGCCGGTCGCGGCGTCATCACC---TCGATCAACTTCCTCGAAGAGAACGGGGCCTAC------GACGGCGTC---
GAT---------TACGTCTCCTACGACGTTCTTGGCGACGTGGTTTGTGGCGGCTTCGCCATGCCGATCCGCGAGAAC---
AAGGCGCAGGAAATCTACATCGT-------

>HQ335967.1_Uncultured_soil_3

TCGACCCGTCTTATTCTGCACGCCAAGGCGCAGGACACTATCCTGTCG------CTC---
GCCGCGAATGCGGGTTCGGTCGAGGATCTCGAA---
ATCGAAGAAGTGATGAAGGTCGGTTATCGGGACATCAAGTGCGTTGAGTCGGGTGGTCCGGAGCCGGGCGTCGG
CTGCGCCGGTCGTGGCGTTATCACC---TCGATCAACTTCCTGGAAGAGAACGGCGCATAC------GAGGACATC---
GAC---------TACGTGTCCTACGACGTGCTGGGTGACGTCGTGTGCGGCGGTTTCGCGATGCCGATCCGCGAGAAC---
AAGGCGCAGGAAATCTACATCGT-------

>HQ336019.1_Uncultured_soil_4

TCGACCCGTCTTATCTTGCACGCCAAGGCGCAGGACACCATCCTGTCG------CTC---
GCGGCGAATGCGGGTTCGGTCGAAGACCTCGAA---
ATCGAAGAAGTGATGAAGGTCGGTTATCGGGACATCAAGTGCGTTGAGTCGGGTGGTCCGGAGCCGGGCGTCGG
TTGCGCTGGCCGCGGCGTCATCACC---TCGATCAACTTCCTGGAAGAGAACGGCGCCTAC------GAGGACATC---
GAT---------TACGTGTCCTACGACGTGCTGGGCGACGTGGTTTGCGGCGGTTTCGCGATGCCGATCCGTGAGAAC---
AAGGCGCAGGAAATCTACATCGTT------

>AY225107.1_PCRreagent

TCGACCCGTCTTATCCTTCACTCGAAGGCCCAGGACACCATCCTCAGC------CTC---
GCCGCTGCCGCTGGTTCCGTTGAGGACCTCGAA---
ATCGAAGACGTCATGAAGGTCGGTTACCTCGACATCCGTTGCGTCGAGTCGGGTGGTCCGGAGCCGGGCGTTGGC
TGCGCGGGTCGTGGTGTTATCACC---TCGATCAACTTCCTCGAGGAAAACGGCGCTTAC------GAAGACGTT---GAC-
--------TACGTTTCCTACGACGTTCTCGGCGACGTGGTCTGCGGCGGTTTCGCCATGCCGATCCGTGAGAAC---
AAGGCTCAGGAAATTTACATCGTCATGTCC

>AJ716247.1_Uncultured_mine spoils_2

TCGACCCGTCTTATCCTTCACTCGAAGGCTCAAGACACCATCCTCAGC------CTC---
GCCGCTGCCGCTGGTTCGGTCGAAGACCTTGAA---
ATCGAAGACGTCATGAAGGTCGGCTACCTCGACATCAAGTGCGTCGAGTCGGGCGGTCCGGAGCCAGGCGTTGGT
TGCGCCGGCCGCGGCGTTATCACC---TCGATCAACTTCCTTGAGGAAAACGGCGCTTAC------GAAGACACC---GAT-
--------TACGTTTCCTATGACGTTCTCGGCGACGTGGTCTGCGGCGGTTTCGCCATGCCGATCCGTGAGAAC---
AAGGCTCAGGAAATCTACATCGT-------

>HF559626.1_Uncultured_wood_3

--GCTGGTTCGGTCGAAGACCTCGAG---
ATCGAAGACGTCATGAAAATCGGCTATCTCGACATCAAGTGCGTCGAGTCCGGTGGTCCGGAGCCGGGCGTTGGT
TGCGCCGGTCGCGGTGTTATCACC---TCGATCAACTTCCTTGAAGAGAACGGCGCTTAC------GAAGACGTT---GAT-
--------TACGTCTCCTACGACGTGCTCGGCGACGTTGTCTGCGGCGGCTTTGCTATGCCGATCCGTGAGAAC---
AAGGCTCAGGAAATCTACATCGT-------

>AM110721.1_Methylocapsa_fungus

--GCTGGTTCGGTCGAAGACCTTGAA---
ATCGAAGACGTCATGAAGATTGGCTACCTCGACATCAAGTGCGTCGAGTCGGGCGGTCCGGAGCCGGGCGTCGGC
TGCGCCGGTCGCGGTGTCATCACC---TCGATCAACTTCCTTGAAGAGAACGGCGCTTAC------GAAGACGTT---GAT-
--------TACGTTTCCTATGACGTGCTCGGCGACGTTGTTTGCGGCGGTTTCGCTATGCCGATCCGTGAGAAC---
AAGGCTCAGGAAATCTACATCGT-------

>AJ716267.1_Uncultured_mine spoils_2

TCGACCCGCCTCATCCTGCACGCCAAGGCCCAAGACACCATTCTGAGC------CTC---
GCCGCTGCCGCTGGTTCGGTCGAAGACCTCGAA---
ATCGAAGACGTCATGAAGGTCGGCTACCTCGACATCAAGTGCGTTGAGTCGGGCGGTCCGGAGCCGGGCGTCGG
CTGCGCTGGTCGTGGCGTTATCACC---TCGATCAACTTCCTTGAGGAGAACGGCGCTTAC------GAAGACGTC---
GAT---------TACGTCTCCTATGACGTGCTCGGCGACGTCGTCTGCGGCGGTTTCGCCATGCCGATCCGTGAGAAC---
AAGGCTCAGGAAATCTACATCGTT------

>HF559607.1_Uncultured_wood_12

--GCTGGTTCGGTCGAAGATCTCGAG---
ATCGAAGACGTGATGAAGGTCGGCTACCTCGACATCAAGTGCGTCGAGTCGGGCGGTCCGGAGCCGGGCGTCGG

CTGCGCCGGTCGTGGCGTTATCACC---TCGATCAACTTCCTCGAGGAAAATGGCGCTTAC------GAAGACGTT---GAT---------TACGTCTCCTATGACGTGCTCGGCGACGTGGTCTGCGGCGGTTTCGCCATGCCGATCCGTGAGAAC---AAGGCTCAGGAAATCTACATCGT-------

>KF800054.1_Rhodoblastus

TCCACCCGCCTGATCCTGCACGCCAAGGCGCAGGACACCATCCTGTCG------CTG---GCCGCCGAAGCCGGTTCGGTGGAAGATCTCGAA---CTCGAAGACGTGCTGAAGGTCGGCTTCGGCGACATCAAGTGCGTTGAGTCCGGCGGTCCGGAGCCCGGCGTGGGCTGCGCCGGCCGCGGCGTCATCACC---GCCATCAACTTCCTCGAAGAAAACGGCGCCTAT------GACGACGTG---GAC---------TATGTGTCCTACGACGTGCTCGGCGACGTGGTCTGCGGCGGCTTCGCCATGCCGATCCGCGAGAAC---AAGGCCCAGGAAATCTACATCGT-------

>AF315429.1_Uncultured_soil_2

TCCACCCGTCTGATCCTGCACGCCAAGGCTCAGGACACCATCCTGTCG------CTG---GCCGCGGCCGCCGGTTCGGTCGAAGACCTCGAA---CTCGAAGACGTGATGAAGGTCGGCTATCGCGACATCCGTTGCGTTGAGTCCGGCGGTCCGGAGCCGGGCGTTGGTTGCGCTGGTCGTGGCGTTATCACC---TCGATCAACTTCCTCGAAGAGAACGGCGCCTAC------GAAGACATC---GAC---------TATGTCTCCTATGACGTTCTCGGCGACGTGGTCTGCGGCGGCTTCGCCATGCCGATCCGCGAGAAC---AAGGCTCAGGAAATCTACATCGT-------

>FR822664.1_Uncultured_soil_2

TCCACCCGCCTAATCCTGCACGCCAAGGCCCAGGACACCATCCTGTCG------CTC---GCCGCCGAAGCCGGCTCGGTCGAGGATCTCGAA---CTCGAAGATGTGATGAAAATCGGCTATCGCGACATCAAGTGCGTTGAGTCCGGCGGTCCGGAGCCCGGCGTTGGTTGCGCGGGTCGCGGCGTTATCACC---TCGATCAACTTCCTCGAAGAAAACGGCGCCTAC------GAAGACATC---GAC---------TATGTGTCTTATGACGTGCTCGGCGACGTGGTGTGTGGCGGTTTCGCCATGCCGATCCGCGAGAAT---AAGGCTCAGGAAATCTACATCGT-------

>EU912967.1_Uncultured_soil_4

TCCACCCGTTTGATTCTTCACGCAAAAGCACAGTCAACTGTTATGGAC------CTG---GTGCGGGAAAGAGGGACGGTTGAGGATCTGGAG---CTGGATGATGTGCTTAAGGTTGGTTACGGTGAGGTCAAGTGCGTGGAATCGGGTGGCCCAGAGCCGGGCGTCGGCTGTGCAGGCCGTGGTGTTATTACC---GCCATCAACTTCCTTGAAGAGAATGGCGCCTAC---ACGGACGACCTC---GAC---------TTCGTGTTCTATGATGTTCTCGGTGACGTTGTTTGTGGTGGTTTTGCCATGCCGATCCGGGAAGGC---AAGGCTGAAGAGATTTATATCGTCTGTTC-

>GU193438.1_Uncultured_marine mat_13

TCAACCCGTCTGATCCTCCACGCCAAAGCCCAGGAAACCGTCATGGAC------AAG---GTCCGTGAACTGGGTACCGTTGAGGATCTGGAA---CTGGAGGATGTGCTCAAGGTCGGCTACGGCGGCGTGAAATGTGTCGAATCAGGCGGTCCAGAACCTGGGGTTGG

CTGTGCCGGTCGCGGTGTTATCACT---GCCATCAACTTCTGTGAAGAAGAAGGCGCCTAC---ACAGATGATCTC---
GAT---------TTTGTTTTTTATGATGTTCTTGGTGATGTTGTCTGCGGCGGGTTCGCCATGCCGATTCGTGAGAAC---
AAAGCCCAGGAGATCTACATCGT-------

>AY221759.1_Uncultured_saline lake_3

---GGCACCGTTGAGGATCTGGAA---
CTGGATGATGTGCTCCGGATTGGCTATGGCGGAGTCAAGTGCGTTGAGTCCGGCGGTCCGGAACCGGGGGTCGG
CTGTGCCGGTCGCGGCGTTATCACC---GCCATCAACTTCTGTGAAGAAGAAGGCGCTTAC---ACCCCCGACCTC---
GAC---------TTTGTTTTTTATGATGTTCTTGGTGACGTCGTCTGTGGCGGGTTCGCCATGCCGATTCACGAGAAC---
AAGGCCCAGGAAATCTACATCGTT------

>JF897105.1_Uncultured_marine mat_118

TCCACCCGTCTGATCCTTCATGCCAAAGCGCAGGAAACGGTGATGGAC------AAG---
GTCCGGGAACTCGGCACCGTCGAAGATCTGGAA---
CTTGAGGATGTGCTCAGGGTTGGCTACGGCGGCATCAAGTGCGTTGAATCAGGCGGTCCTGAGCCCGGTGTCGGC
TGTGCCGGCCGCGGTGTTATTACC---GCTATCAACTTCTGTGAAGAGGAAGGTGCCTAT---ACCCCTGACCTG---
GAC---------TTCGTTTTTTATGATGTTCTCGGTGACGTCGTCTGCGGCGGTTTTGCCATGCCCATCCGCGAGAAC---
AAGGCCCAGGAGATCTACATCGTCTGCTC-

>GU192716.1_Uncultured_marine mat_22

TCCACCCGTCTGATCCTCCATGCCAAAGCCCAGGAAACGGTTATGGAC------AAG---
GCCCGGGAACTCGGCACCGTCGAGGATCTGGAG---
TTGTCCGATGTTCTCAGGGTCGGTTATGGCGGCATCAAGTGCGTTGAGTCAGGCGGTCCCGAGCCCGGTGTCGGCT
GTGCCGGCCGCGGCGTTATCACC---GCAATCAATTTCTGTGAGGAAGAAGGTGCCTAC---ACCCCCGACCTG---
GAC---------TTTGTTTTTTATGATGTTCTCGGTGACGTCGTCTGCGGCGGCTTTGCCATGCCGATCCGCGAGAAC---
AAGGCCCAGGAGATCTACATCGT-------

>GU192729.1_Uncultured_marine mat_2

---GGCACCGTCGAGGACCTGGAA---
CTTGAGGATGTCCTCAGGGTCGGCTACGGTGGCATCAAGTGCGTTGAATCAGGCGGTCCTGAACCCGGCGTCGGC
TGTGCCGGTCGGGGCGTTATTACC---GCTATCAATTTCTGCGAAGAAGAAGGCGCTTAC---ACCCCTGACCTG---
GAC---------TTCGTTTTTTATGATGTTCTCGGCGACGTCGTCTGCGGCGGCTTTGCCATGCCGATCCGTGAGAAT---
AAAGCCCAGGAGATCTACATCGT-------

>GU193897.1_Uncultured_marine mat_9

TCCACCCGCCTGATCCTCCATGCCAAAGCGCAGGAAACGGTAATGGAC------AAG---
GTCCGGGAACTCGGCACCGTCGAGGACCTGGAG---
CTTGAGGATGTTCTCAGGGTCGGCTATGGCGGCATCAAGTGCGTTGAGTCAGGCGGTCCTGAACCCGGTGTCGGC
TGTGCCGGCCGCGGCGTTATTACC---GCGATCAATTTCTGTGAAGAAGAAGGTGCTTAC---ACCCCCGATCTG---

GAC---------TTCGTTTTTATGATGTTCTCGGTGACGTCGTCTGCGGCGGCTTTGCCATGCCGATCCGTGAGAAC---
AAAGCCCAGGAAATCTACATCGT-------

>DQ520451.1_Uncultured_soil_2

TCAACACGTTTGATCCTGCATGCTAAAGCCCAGAATACCGTAATAGAT------CTG---
GTACGTGAGCTGGGAACCGTTGAGGATCTGGAG---
TCGGAAGATGTCCTCAAGGTCGGTTATGGCGATATCAAATGCGTTGAATCCGGTGGTCCTGAGCCGGGTGTCGGC
TGTGCCGGTCGCGGTGTTATTACA---GCGATCAACTTTCTGGAAGAAAACGGCGCCTAC---ACTGATGATCTG---
GAT---------TTTGTCTTCTACGATGTTCTCGGTGACGTTGTCTGCGGCGGATTCGCCATGCCGATCCGCGAAGGC---
AAGGCTGAAGAGGTCTACATCGTCTGCTC-

>HQ660863.1_Uncultured_marine_4

--GGTACCGTTGAGGATTTAGAG---
TTAGAAGATGTCATGAAGCGCGGTTATGGCGAAGTGATGTGCGTTGAATCTGGTGGCCCTGAGCCGGGTGTTGGT
TGTGCTGGGCGTGGTGTTATTACC---GCTATCAACTTCTTGGAGGAAGAGGGCGCATAC---ACCGACGATCTT---
GAT---------TTTGTTTTTTACGATGTTTTAGGTGACGTTGTCTGTGGTGGTTTCGCCATGCCGATTCGTGAAAAC---
AAAGCGCAAGAGATCTACATCGTT------

>AY181004.1_Uncultured_root_6

TCCACCCGTCTGATTCTCCACGCTAAAGCGCAGTCAACGGTTATGGAC------CTG---
GTCCGAGAACTGGGAACCGTTGAGGACCTGGAG---
CTTGAAGATGTTCTTAAAGTCGGCTATGGCGATGTGAAGTGCGTCGAGTCGGGTGGTCCCGAGCCGGGTGTTGGC
TGTGCAGGCCGTGGTGTTATTACC---GCCATTAACTTCCTGGAAGAAAACGGCGCTTAT---ACCCCTGATCTT---
GAT---------TTCGTATTTTACGACGTACTTGGTGACGTTGTTTGCGGTGGTTTTGCGATGCCGATCCGTGAAGGC---
AAGGCGGAAGAGATTTACATCGTCTGCTC-

>DQ776493.1_Uncultured_terrestrial rhizosphere_4

TCCACCCGTCTGATCCTGCACGCCAAGGCCCAGAATACAGTAATGGAC------CTG---
GTGCGGGAGCGCGGCACCGTCGAGGACCTGGAG---
CTGGAAGACGTCATGAAGGTTGGCTATGGCGATGTCAAATGTGTCGAATCAGGTGGTCCTGAGCCTGGTGTTGGT
TGTGCCGGCCGCGGTGTCATCACC---GCCATCAACTTTCTGGAAGGGAACGGCGCCTAC---ACTCCTGACCTC---
GAC---------TTCGTATTCTACGATGTTCTGGGTGACGTCGTCTGCGGCGGGTTTGCCATGCCGATTCGCGAAGGA---
AAGGCAGAAGAGATCTATATCGTCTGCTC-

>DQ776311.1_Uncultured_terrestrial rhizosphere_4

TCAACCCGTCTGATCCTGCACGCCAAGGCGCAGAATACGGTTATGGAC------CTG---
GTGCGCGAGCGCGGCACCGTCGAGGATCTGGAG---
CTGGAAGAGGTCATGAAGATCGGCTACGGCGACATCAAATGCGTCGAGTCCGGCGGGCCTGAGCCGGGCGTTGG
CTGTGCCGGTCGCGGAGTTATCACC---GCCATCAACTTCCTGGAAGAAAACGGCGCGTAC---ACCCCGGACCTC---

GAT---------TTCGTATTCTACGATGTTCTGGGTGACGTCGTCTGCGGCGGGTTCGCCATGCCGATCCGCGAGGGC---AAGGCTGAAGAGATCTATATCGT-------

>KF848125.1_Uncultured_soil_2

TCAACCCGTCTGATCCTGCACGCCAAGGCGCAGAATACGGTTATGGAC------CTG---GTGCGCGAGCGTGGCACCGTCGAGGATTTGGAG---CTGGAAGAGGTCATGAAGATCGGCTACGGCGACATCAAATGCGTCGAGTCCGGCGGACCTGAGCCGGGCGTTGGCTGTGCCGGTCGCGGTGTCATCACC---GCCATCAACTTCCTGGAAGAAAACGGCGCGTAC---ACCCCTGACCTC---GAC---------TTCGTATTCTATGATGTTCTGGGTGACGTGGTCTGCGGCGGGTTCGCCATGCCGATCCGCGAGGGC---AAGGCTGAAGAGATTTATATCGT-------

>KF847997.1_Uncultured_soil_5

TCAACCCGTCTGATCCTGCACGCCAAGGCGCAGAATACTGTTATGGAC------CTG---GTGCGCGAGCGCGGCACCGTCGAGGACCTGGAG---CTTGAAGAGGTCATGAAGATCGGCTACGGCGACATCAAATGCGTCGAGTCCGGCGGACCTGAACCGGGTGTCGGTTGTGCCGGTCGCGGTGTCATCACC---GCTATCAACTTCCTTGAAGAAAACGGCGCGTAC---ACCCCTGACCTC---GAC---------TTCGTGTTCTATGATGTTCTGGGTGACGTTGTCTGCGGCGGGTTTGCCATGCCGATCCGCGAGGGC---AAGGCTGAAGAGATTTATATCGTT------

>EU305261.1_Uncultured_glacier_4

TCAACCCGTCTGATCCTGCACGCCAAGGCGCAGAATACGGTTATGGAC------CTG---GTGCGCGAGCGCGGTACCGTTGAGGACCTGGAG---CTGGAAGAGGTCATGAAGATCGGCTACGGCGACATCAAATGCGTCGAGTCCGGCGGACCTGAACCGGGTGTCGGCTGTGCCGGTCGTGGTGTCATCACC---GCCATCAACTTCCTTGAAGAAAACGGCGCGTAC---ACCCCTGACCTC---GAC---------TTCGTGTTCTATGATGTTCTGGGTGACGTCGTCTGCGGCGGGTTCGCCATGCCGATCCGCGAGGGG---AAGGCTGAAGAAATTTATATCGT-------

>KF847897.1_Uncultured_soil_2

TCAACCCGCCTGATTCTGCACGCCAAGGCACAGAATACGGTTATGGAC------CTG---GTGCGCGAGCGCGGCACCGTCGAGGATCTTGAG---CTGGAAGAGGTCATGAAGATCGGCTACGGCGACATCAAATGCGTCGAGTCCGGCGGACCTGAGCCGGGTGTCGGCTGTGCCGGTCGCGGTGTCATCACT---GCCATCAACTTCCTGGAAGAAAACGGCGCGTAC---ACACCAGACCTT---GAT---------TTCGTATTCTACGATGTTCTGGGTGACGTTGTCTGCGGCGGGTTCGCCATGCCGATCCGCGAGGGG---AAGGCCGAAGAGATCTATATCGT-------

>KF847954.1_Uncultured_soil_4

TCAACGCGTCTGATTCTGCACGCCAAGGCGCAGAATACGGTTATGGAC------CTG---GTGCGCGAGCGCGGCACCGTCGAGGATCTGGAG---CTGGAAGAGGTCATGAAGATCGGCTACGGCGACATCAAATGCGTCGAGTCCGGCGGACCTGAACCGGGTGTCGGCTGTGCCGGTCGCGGCGTCATCACC---GCCATCAACTTCCTGGAAGAAAACGGCGCGTAC---ACCCCGGACCTC---

GAC---------TTCGTGTTTTATGATGTTCTGGGTGACGTTGTCTGCGGCGGGTTCGCCATGCCGATCCGCGAGGGG---
AAGGCTGAAGAGATTTATATCGT-------

>KF847888.1_Uncultured_soil_2

TCCACCCGTCTGATTCTGCACGCCAAGGCTCAGAATACGGTTATGGAC------CTG---
GTGCGCGAGCGCGGCACCGTCGAGGACCTGGAG---
CTGGAAGAGGTCATGAAGATCGGCTACGGCGACATCAAATGCGTCGAGTCCGGCGGACCTGAACCAGGTGTCGG
CTGTGCCGGTCGCGGTGTCATCACA---GCCATCAACTTCCTTGAAGAAAACGGCGCGTAC---ACACCAGACCTC---
GAT---------TTCGTATTCTATGATGTTCTGGGTGACGTTGTCTGCGGCGGGTTCGCCATGCCGATCCGCGAGGGC---
AAGGCTGAAGAAATTTATATCGTT------

>AY819562.1_Uncultured_soil_5

TCAACCCGTCTGATTCTGCACGCCAAGGCGCAGAATACGGTTATGGAC------CTG---
GTGCGCGAGCGCGGCACCGTCGAGGATCTGGAG---
CTGGAAGAGGTCATGAAGATCGGCTACGGCGACATCAAATGCGTCGAGTCCGGCGGACCTGAACCGGGTGTCGG
CTGTGCCGGTCGCGGTGTCATCACC---GCCATCAACTTCCTTGAAGAGAACGGCGCGTAC---ACACCAGACCTC---
GAC---------TTCGTATTTTACGATGTTCTGGGTGACGTTGTCTGCGGCGGGTTCGCCATGCCGATCCGCGAGGGC---
AAGGCCGAAGAGATCTATATCGT-------

>DQ520356.1_Uncultured_soil_2

TCAACCCGTCTGATTCTGCACGCCAAGGCGCAGAATACGGTTATGGAC------CTG---
GTGCGCGAGCGCGGCACTGTCGAGGATTTGGAG---
CTGGAAGAGGTCATGAAGATCGGCTACGGCGACATCAAATGCGTCGAGTCCGGCGGACCTGAACCGGGTGTCGG
CTGTGCCGGCCGCGGTGTCATCACC---GCCATCAACTTCCTTGAAGAAAACGGCGCGTAC---ACACCAGACCTC---
GAC---------TTTGTATTCTATGATGTTCTGGGTGACGTTGTCTGCGGCGGGTTCGCCATGCCGATCCGCGAGGGC---
AAGGCTGAAGAAATTTATATCGT-------

>FJ381622.1_Uncultured_soil_4

TCCACCCGTCTGATTCTTCACGCCAAGGCCCAGTCCACGGTTATGGAT------CTG---
GTCCGTGAGCTCGGAACGGTTGAGGACCTTGAG---
CTTGATGACGTACTGAAGGTCGGCTATGGCGACGTCAAGTGCGTGGAGTCGGGTGGTTCCGAGCCGGGTGTCGGC
TGTGCCGGCCGCGGCGTCATCACC---GCCATCAACTTCCTGGAAGAGAACGGCGCCTAC---ACCCCTGATCTC---
GAC---------TTCGTCTTCTACGACGTTCTCGGCGACGTTGTCTGTGGCGGGTTCGCCATGCCGATCCGCGAGAAC---
AAGGCGGAAGAGATTTACATCGTCTGCTC-

>KF847297.1_Uncultured_soil_14

TCCACCCGCCTGATCCTGCACGCCAAGGCGCAGTCCACCGTCATGGAC------CTG---
GTGCGCGAGCTCGGTACGGTAGAGGATCTGGAG---
CTTGAGGACGTCCTTAAGGTCGGTTACGGCGACGTGAAATGCGTGGAGTCCGGCGGTCCCGAGCCGGGGGTTGG
CTGTGCAGGGCGCGGCGTCATCACC---GCCATCAACTTCCTCGAGGAAAACGGCGCCTAC---ACCCCGGACCTC---

GAC---------TTCGTCTTCTACGACGTTCTGGGCGACGTGGTCTGCGGCGGGTTCGCCATGCCGATCCGCGAGAAC---
AAGGCGGAAGAGATCTACATCGTCTGCTC-

>EU052656.1_Uncultured_South China Sea_4

TCGACCCGCCTGATCCTGCATGCCAAGGCTCAGGCCACAGTCATGGAC------AAG---
GTTCGTGAACCCGGCACCGTCGAGGATCTGGAA---
CTGGAAGATGTCATGAAGGTCGGCTACCACGATGTTAAATGTGTTGAGTCCGGTGGCCCCGAGCCAGGTGTTGGT
TGTGCTGGTCGTGGAGTCATAACC---GCGATCAACTTCCTCGAAGAAGAAGGCGCCTAC---ACTCCCGACCTC---
GAC---------TTTGTCTTCTATGATGTTCTCGGCGACGTGGTCTGTGGCGGTTTCGTCATGCCGATCTGCGAGAAC---
AAGGCTCAGGAGATTTACATCGT-------

>FJ686509.1_Uncultured_marine sediment_4

TCCACTCGCCTGATCCTGCACGCCAAGGCTCAGACAACAGTAATGGAC------AAA---
GTCCGCGAGCTTGGCACTGTTGAGGACCTGGAG---
CTGGAAGATGTTCTGAAGCGTGGCTATGGCGAGGTCATGTGCGTTGAGTCGGGTGGCCCGGAACCGGGTGTCGG
TTGTGCAGGTCGTGGTGTTATCACT---GCGATCAACTTCCTCGAGGAAGAAGGCGCTTAC---ACTCCTGATCTC---
GAT---------TACGTCTTCTATGACGTTCTCGGCGACGTTGTCTGCGGCGGCTTCGCTATGCCTATCCGCGAGAAC---
AAGGCTCAGGAAATCTACATTGT-------

>GU193556.1_Uncultured_marine mat_4

---GGCACCGTTGAGGACTTGGAG---
TTGGAAGATGTTCTTAAGTGGGGATACGGCGATGTTAAATGCGTTGAGTCGGGCGGTCCTGAGCCAGGTGTTGGT
TGTGCCGGTCGTGGTGTTATCACC---GCCATCAACTTCCTGGAAGTAGAAGGTGCTTAC---ACTGACGACCTC---
GAC---------TTCGTTTTCTATGACGTTCTCGGTGACGTTGTCTGCGGTGGTTTCGCTATGCCGATCCGCGAGAAC---
AAAGCGGAAGAGATCTACATTGTT------

>HQ611429.1_Uncultured_marine_3

TCGACCCGTCTGATTCTGCACGCCAAAGCACAGGAAACTGTTATGGAC------AAG---
GTCCGCGAGCTCGGTACCGTCGAGGATCTGGAG---
CTCGAAGATGTGATGAAGGTTGGCTACGGCGACGTCAAATGCGTCGAGTCGGGTGGTCCAGAGCCGGGCGTCGG
TTGCGCCGGTCGTGGTGTTATCACC---GCTATCAACTTCCTGGAAGAAGAAGGCGCCTAC---ACCGACGACCTC---
GAT---------TTCGTCTTCTACGACGTCCTCGGTGACGTTGTCTGCGGTGGCTTTGCCATGCCGATCCGCGAGAAC---
AAAGCCGAAGAGATCTACATCGT-------

>EU052526.1_Uncultured_South China Sea_4

TCCACCCGTCTGATTCTGCATGCAAAAGCTCAGGAAACAGTTATGGAC------AAG---
GTCCGGGAGCTCGGTACCGTTGAGGACCTGGAG---
CTTGAGGATGTACTGAAGTGGGGTTACGACGACGTCAAGTGCGTTGAATCAGGTGGTCCGGAGCCGGGTGTCGGT
TGCGCCGGTCGTGGTGTTATCACA---GCCATCAACTTTCTCGAGGAAGAGGGTGCATAC---ACTGATGACCTC---

GAC---------TTCGTTTTCTACGACGTTCTAGGTGATGTTGTCTGCGGTGGTTTTGCTATGCCGATCCGAGAAAAC---AAGGCCGAAGAAATATATATTGT-------

>HQ223497.1_Uncultured_marine sediment_2

TCCACCCGCCTGATCCTGCATGCAAAAGCTCAGGAAACAGTTATGGAC------AAG---GTCCGGGAGCTCGGTACTGTTGAGGACCTGGAG---CTGGAAGATGTGCTGAAGTGGGGCTACGGCGACGTCAAGTGCGTTGAATCAGGTGGCCCGGAGCCGGGTGTCGGCTGCGCCGGTCGTGGTGTTATAACA---GCGATCAACTTTCTCGAGGAAGAGGGTGCATAC---ACTGATGACCTC---GAC---------TTCGTCTTCTACGACGTTCTCGGTGATGTTGTTTGCGGGGGCTTTGCAATGCC---

>KF861045.1_Uncultured_soil_5

--GGCACCGTCGAGGACCTCGAA---CTGGAAGACGTGATGAAGTACGGCTACGGCGACGTCAAGTGCGTCGAGTCGGGTGGCCCGGAGCCGGGTGTCGGCTGCGCCGGTCGTGGAGTTATTACT---GCTATCAACTTCCTCGAGGAAGAGGGAGCTTAC---ACTGACGATCTC---GAT---------TTCGTCTTCTATGACGTTCTCGGTGACGTTGTCTGTGGCGGATTCGCCATGCCGATCCGCGAGAAC---AAGGCGGAAGAGATCTATATCGT-------

>KF861115.1_Uncultured_soil_4

--GGCACTGTTGAGGATCTTGAA---TTAGATGATGTGTTGAAGTGGGGTTACGGCGACGTCAAGTGCGTTGAGTCCGGTGGCCCTGAGCCGGGTGTCGGTTGCGCCGGTCGTGGTGTTATTACT---GCTATCAACTTCCTCGAGGAAGAGGGCGCTTAC---ACAGACGACCTC---GAT---------TTCGTTTTCTACGACGTTCTCGGTGACGTTGTCTGCGGTGGATTCGCTATGCCGATCCGCGAGAAC---AAGGCAGAAGAGATCTACATCGTT------

>HM063760.1_Uncultured_marine sediment_7

TCCACTCGCCTGATCCTGCACGCAAAAGCCCAGGAAACTGTCATGGAC------AAA---GTTCGTGAGCTCGGAACTGTCGAGGACCTGGAG---CTGGAAGATGTATTGAAGTGGGGATACGGCGACGTCAAGTGCGTTGAGTCCGGTGGTCCTGAGCCGGGTGTTGGTTGTGCCGGTCGTGGTGTTATCACA---GCGATCAACTTCCTCGAGGAAGAGGGCGCTTAC---ACGGACGACCTC---GAT---------TTCGTCTTCTACGACGTTCTCGGCGACGTTGTCTGTGGTGGATTCGCGATGCCGATCCGCGAGAAC---AAGGCAGAAGAAATCTATATCGTT------

>DQ098247.1_Uncultured_marine_2

TCCACCCGTCTGATCCTACACGCGAAAATGCAGGAGACGGTCATGGAC------AAG---GTCCGTGAGCTCGGCACTGTCGAGGACCTGGAG---CTGGAAGATGTATTGAAGTGGGGCTACGGCGACGTCAAGTGCGTTGAGTCCGGTGGTCCTGAGCCTGGTGTTGGTTGTGCGGGCCGTGGCGTTATCACA---GCCATCAACTTCCTCGAGGAAGAGGGCGCTTAC---ACAGACGACCTC---GAC---------TTCGTCTTCTACGACGTTCTCGGTGACGTTGCCTGCGGTGGCTTCGCAATGCCGATCCGTGAGAAC---AAGGCTGAAGAGATTTATATCGTT------

>EU052415.1_Uncultured_South China Sea_4

TCTACCCGCCTTATCCTGCACGCGAAAGCCCAGGAAACAGTTATGGAC------AAA---
GTCCGTGAGCTCGGCACTGTCGAGGACCTGGAG---
CTGGAAGATGTATTGAAGTGGGGTTACGGCGACGTCAAGTGCGTTGAGTCCGGTGGCCCTGAGCCTGGTGTTGGT
TGTGCAGGCCGTGGTGTTATCACA---GCCATCAACTTCCTCGAGGAAGAGGGCGCTTAC---ACAGATGACCTC---
GAT---------TTCGTATTCTACGACGTTCTCGGTGACGTTGTCTGCGGTGGCTTCGCAATGCCGATCCGTGAGAAC---
AAGGCTGAAGAAATCTACATCGTT------

>EU052524.1_Uncultured_South China Sea_4

TCCACCCGCCTGATCCTTCACGCGAAAATGCAGGAGACGGTCATGGAC------AAG---
GTTCGCGAGCTGGGTACTGTTGAGGATCTCGAG---
TTGGAAGATGTATTGAAGTGGGGCTACGGTGACGTCAAGTGTGTTGAATCTGGCGGTCCTGAGCCCGGTGTTGGT
TGTGCTGGTCGTGGTGTTATTACA---GCGATCAACTTCCTTGAGGAAGAGGGTGCTTAC---ACTGATGATCTC---
GAC---------TTCGTCTTCTACGACGTTCTCGGTGACGTCGTCTGTGGCGGATTCGCTATGCCGATCCGTGAGAAC---
AAGGCGGAAGAGATTTACATCGTT------

>HM219757.1_Uncultured_marine mudflat_2

TCCACCCGTCTGATCCTTCACGCGAAAATGCAGGAGACGGTCATGGAC------AAG---
GTCCGTGAGCTGGGTACTGTTGAGGACCTCGAG---
TTGGAAGATGTGTTGAAGTGGGGCTACGGCGACGTCAAGTGTGTTGAGTCCGGTGGTCCTGAGCCGGGTGTCGGC
TGCGCTGGTCGTGGTGTTATTACA---GCGATCAACTTCCTCGAGGAAGAAGGCGCTTAC---ACTGATGACCTC---
GAC---------TTCGTCTTCTATGATGTTCTGGGTGACGTTGTTTGCGGTGGTTTTGCTATGCCGATCCGCGAAAAC---
AAGGCCGAAGAGATCTACATCGTT------

>FJ756633.1_Uncultured_marine_4

TCCACCCGCCTGATCCTTCACGCGAAAATGCAGGAGACAGTCATGGAC------AAG---
GTCCGTGAGCTGGGCACTGTTGAGGACCTCGAA---
TTAGAAGATGTGTTGAAGTGGGGTTACGGCGACGTTAAATGTGTTGAGTCCGGTGGTCCTGAGCCGGGCGTTGGT
TGTGCCGGACGTGGTGTTATTACC---GCCATCAACTTCCTCGAGGAAGAGGGCGCGTAC---ACTGATGACCTG---
GAC---------TTCGTCTTTTATGACGTGCTTGGTGATGTTGTCTGCGGTGGTTTCGCTATGCCGATCCGCGAGAAT---
AAGGCCGAAGAGATTTACATCGTT------

>HM219676.1_Uncultured_marine mudflat_2

TCCACCCGCCTGATCCTCCACGCGAAAACGCAGGAGACAGTCATGGAC------AAG---
GTCCGTGAACTGGGCACTGTAGAGGATCACGAA---
CTGGAATATGTGTTGAAGTGGGGGTACGGGGACGTGAAATGCGTTGAGTCCGGTGGTCCTGAGCCGGGTGTCGG
CTGCGCCGGTCGTGGTGTTATTACC---GCCATCAACTTTCTCGAGGAAGAGGGCGCCTAC---ACTGATGACCTC---
GAC---------TTCGTCTTTTATGACGTCCTTGGTGACGTTGTCTGCGGTGGTTTCGCTATGCCAATCCGTGAGAAC---
AAGGCCGAAGAAATTTACATCGTT------

>DQ098253.1_Uncultured_marine_7

TCAACTCGCCTGATTCTGCACGCTAAAGCCCAGGACACAGTTATGGAC------AAG---
GTTCGCGAGCTTGGCACCGTTGAGGACCTTGAG---
TTGGAAGATGTCTGTAGGCGTGGTTACGGCGACGTCATGTGCGTTGAGTCGGGCGGTCCAGAGCCTGGAGTCGGT
TGTGCCGGACGAGGTGTTATCACC---GCGATCAACTTCCTCGAGGAAGAGGGCGCTTAC---ACCCCGGATCTT---
GAT---------TATGTTTTTACGACGTCCTCGGTGACGTCGTCTGCGGCGGCTTCGCCATGCCAATCCGCGAGAAC---
AAGGCTCAGGAGATCTACATTGTT------

>DQ098260.1_Uncultured_marine_2

TCCACTCGACTGATCCTGCACGCCAAGGCCCAGGACACAGTTATGGAC------AAG---
GTCCGCGAGCTTGGCACCGTAGAGGATCTGGAG---
CTGGAGGATGTCTGTAAACGCGGCTACGGCGAAGTCATGTGCGTTGAGTCGGGTGGCCCGGAGCCGGGCGTCGG
CTGCGCCGGCCGTGGAGTTATCACC---GCTATCAACTTCCTTGAGGAAGAAGGTGCTTAC---ACCCCCGACCTC---
GAT---------TATGTTTTCTACGATGTTCTTGGTGATGTTGTATGTGGTGGCTTCGCCATGCCGATCCGAGAAAAC---
AAGGCTCAGGAGATCTACATCGTT------

>GU193881.1_Uncultured_marine mat_2

TCGACCCGTCTGATCCTGCATGCCAAGGCTCAGGACACAGTCATGGAC------AAG---
GTCCGTGAACTCGGTACCGTCGAGGATCTGGAG---
CTGGAGGATGTTTGTAAGCGCGGTTACGGCGATGTCATGTGTGTTGAGTCTGGTGGTCCTGAGCCGGGTGTTGGT
TGTGCCGGTCGTGGTGTTATCACC---GCCATCAACTTTTTGGAAGAAGAGGGGGCTTAC---ACTCCGGATCTC---
GAT---------TATGTTTTCTACGATGTTCTCGGTGACGTTGTTTGTGGCGGTTTCGCTATGCCGATTCGTGAGAAC---
AAGGCCCAGGAGATTTACATCGTT------

>GU193073.1_Uncultured_marine mat_5

TCCACCCGTCTCATCCTGCACGCCAAGGCCCAGACCACGGTTATGGAC------AAG---
GTTCGTGAGCTCGGCACCGTTGAGGACCTGGAG---
CTGGAGGATGTGCTCAAGAAAGGCTACGGCGATGTTATGTGCGTCGAGTCTGGTGGTCCCGAGCCGGGCGTCGGT
TGTGCCGGTCGTGGGGTTATCACC---GCCATCAACTTTCTCGAGGAAGAAGGCGCCTAC---ACTCCGGACCTC---
GAC---------TATGTCTTCTATGATGTTCTCGGTGACGTTGTCTGCGGCGGTTTCGCCATGCCGATCCGTGAAAAT---
AAAGCCCAGGAGATCTACATTGTT------

>DQ077981.1_Uncultured_marine sediment_7

TCTACCCGCCTGATCCTGCATGCCAAAGCCCAGGACACGGTTATGGAC------AAG---
GTTCGTGAACTCGGCACCGTTGAGGATCTGGAA---
CTGGAAGATGTCTGCAAACGCGGCTACGGCGATGTTATGTGTGTCGAGTCGGGCGGTCCCGAGCCAGGCGTTGGT
TGTGCCGGCCGTGGTGTTATTACC---GCCATCAACTTTTTGGAAGAAGAAGGCGCCTAC---ACCCCTGACCTC---
GAC---------TACGTCTTCTATGATGTTCTCGGTGATGTTGTCTGTGGCGGCTTTGCTATGCCGATCCGTGAGAAC---
AAAGCCCAGGAGATCTACATTGTTGTCTCT

>DQ098192.1_Uncultured_marine_42

--GGCACCGTTGAGGATCTGGAA---
CTGGAAGATGTCTGTAAACGCGGCTACGGCGATGTCATGTGCGTCGAGTCGGCGGTCCTGAGCCAGGCGTCGGT
TGTGCCGGGCGTGGTGTTATTACC---GCCATCAACTTTTTGGAAGAAGAAGGCGCCTAC---ACCCCTGACCTC---
GAC---------TACGTTTTCTATGATGTTCTCGGTGATGTTGTCTGCGGCGGCTTTGCTATGCCGATCCGAGAGAAC---
AAAGCCCAGGAGATCTACATCGTTGTCTCT

>EU594031.1_Uncultured_marine sponge_21

---ACACGTCTAATTCTTCATTCAAAGGCTCAGGAGACTATTATGCAT------CTG---
GCCGCCGATGCAGGCAGTGTGGAAGACCTAGAG---
CTGGAAGATGTCCCCAGAGTGGGTTATGGCGATGTCAAGTGTGTCGAATCCGGTGGTCCCGAGCCGGGTGTGGGT
TGTGCCGGGCGCGGAGTGATTACC---GCGATTAACTTCTTAGAGGAAGAAGGAGCCTAC---GAAGAGGATCTG---
AAC---------TTTGTGTTTTATGATGTCTTGGGAGACGTGGTTTGCGGTGGTTTCGCTATGCCTATCCGTGAAGGT---
AAGGCTCAAGAAATCTACATCGTTACCTCT

>AB679086.1_Uncultured_marine_4

TCCACGCGTCTTATTCTGCATTCAAAAGCGCAAAATACTATCATGGAA------ATG---
GCTGCAGAGGCTGGCACGGTAGAGGATCTAGAG---
CTCGAAGAAGTGCTGAAGGTCGGTTTTGGTGATATTAAATGTGTCGAATCAGGCGGTCCAGAGCCTGGCGTTGGT
TGTGCTGGCCGCGGTGTTATTACG---GCAATTAACTTTCTTGAAGAGGAAGGTGCTTAC---GAAGAAGACTTA---
GAT---------TTCGTTTTCTATGATGTATTGGGAGACGTCGTTTGCGGCGGCTTCGCGATGCCCATCCGTGAAAAC---
AAAGCCCAAGAAATATACATCGTTTGCTC-

>HM210395.1_Uncultured_marine_4

TCTACACGACTTACTCTGCACTCAAAAGCGCAAAACACTATAATGGAG------ATG---
GCTGCGCAGGCTGGCACGGTAGAAGATCTCGAG---
CTTGAAGAAGTGCTTAAAGTTGGTTATGGCGACATTAAGTGCGTTGAATCAGGTGGACCAGAGCCAGGTGTTGGC
TGTGCCGGTCGTGGTGTTATTACC---GCGATTAACTTCCTTGAAGAGGAAGGTGCATAT---TCAGACGACCTA---
GAT---------TTCGTATTTTATGATGTCCTTGGGGACGTGGTCTGTGGTGGATTTGCAATGCCTATTCTTGAGAAT---
AAGGCTCAAGAAATTTACATTGTTTGTTCT

>DQ118232.1_Uncultured_marine_4

TCTACACGTCTTATTCTGCACTCAAAAGCGCAAAACACAATAATGGAG------ATG---
GCTGCACAGGCTGGTACGGTAGAGGATCTTGAG---
CTTGAAGAAGTGCTTAAGGTTGGCTTTGGCGATATTAAGTCCGTGGAGTCAGGTGGTCCTGAGCCCTGGAGTTGG
CTGTGCCTGGTCGTGGTGTTATTACCGCCAATTAATTTCCTTGAAGAGGAAGGTGCATAC---ACAGACGTCTTA---
GAT---------TTCGTTTTCTATGATGTTCTAGGTGATGTGGTTTGTGGCGGATTTGCAATCCCTATTGGTGAGAAT---
AAGCCTCAGGAAATCTACATTGTTTGCTCT

>DQ118235.1_Uncultured_marine_30

TCTACACGTCTTATTCTGCACTCAAAAGCGCAAAACACAATAATGGAG------ATG---
GCTGCACTGGCTGGTACGGTAGAGGATCTTGAG---
CTTGAAGAAGTGCTTAAGGTTGGCTTTGGCGATATTAAGTGCGTGGAGTCAGGTGGTCCTGAGCCCGGAGTTGGC
TGTGCTGGTCGTGGTGTTATTACC---GCGATTAATTTCCTTGAAGAGGAAGGTGCATAC---ACAGACGACTTA---
GAT---------TTCGTTTTCTATGATGTCCTAGGTGATGTGGTTTGTGGCGGATTTGCAATGCCTATTCGTGAGAAT---
AAGGCTCAGGAAATCTCCATTGTTTGCTCT

>AY896371.1_GammaA_marine_309

TCCACACGTCTTATTCTGCACTCAAAAGCGCAAAACACAATAATGGAG------ATG---
GCTGCACAGGCTGGCACGGTAGAGGATCTTGAG---
CTTGAAGAAGTGCTTAAGGTTGGCTTTGGCGACATCAAGTGCGTGGAGTCAGGTGGTCCTGAGCCTGGAGTTGGC
TGTGCCGGTCGTGGTGTTATTACC---GCGATTAACTTTCTTGAAGAGGAAGGTGCATAC---TCAGACGACTTA---
GAT---------TTCGTTTTTTATGATGTTCTAGGTGATGTGGTTTGTGGCGGATTTGCAATGCCTATTCGTGAGAAT---
AAGGCTCAGGAAATCTACATTGTTTGCTCT

>AF016617.1_Unidentified_marine_4

TCCACACGTCTTATTCTGCACTCAAAAGCGCAAAACACAATAATGGAG------ATG---
GCTGCACAGGCTGGCACGGTAGAGGATCTTGAG---
CTTGGAGAAGTGCTTAAGGTTGGTTTTGGCGACATTAAGTGCGTGGAGTCAGGTGGTCCAGAGCCTGGAGTTGGC
TGTGCTGGTCGTGGTGTTATTACC---GCGATTAATTTCCTTGAAGAGGAAGGTGCATAT---TCAGACGACTTA---
GAT---------TTCGCTTTTTATGATGCTCTAGGTGATGTGGTTTGTGGCGGATTTGCAATGCCTATTCGTGAGAAT---
AAGGCTCAGGAAATCTACATTGTTTGCTCT

>AY896456.1_Uncultured_marine_13

TCCACCCGTCTTATCTTGCATTCTAAGGCTCAAAATACCATCATGGAA------ATG---
GCTGCTCAGGCAGGTACGGTAGAGGATCTTGAG---
CTTGATGACGTGCTTAAAGTTGGATATGGCAACATTAAGTGTGTAGAATCAGGTGGTCCCGAGCCCGGTGTTGGGT
GTGCTGGTCGTGGTGTTATCACT---GCGATTAATTTCCTCGAAGAGGAAGGCGCGTAT---GAAGACGATCTG---
GAC---------TTTGTTTTTTACGACGTTCTTGGGGACGTGGTTTGTGGCGGTTTTGCCATGCCAATTCGTGAAAAC---
AAAGCGCAGGAAATCTACATTGTTTGCTCT

>DQ831856.1_Uncultured_marine_12

TCCACTCGTCTTATCCTGCATTCCAAAGCTCAAAACACTATCATGGAA------ATG---
GCTGCTCAGGCAGGCACGGTAGAGGATCTGGAG---
CTAGAAGACGTGCTTAAAGTTGGTTATGGTGACATTAAGTGTGTAGAATCAGGTGGTCCCGAGCCCGGTGTTGGG
TGTGCTGGTCGTGGTGTTATCACT---GCGATTAATTTCCTCGAAGAGGAAGGCGCGTAT---GAAGACGATCTG---
GAC---------TTTGTTTTTTACGACGTTCTTGGGGACGTGGTTTGTGGCGGTTTTGCCATGCCAATTCGTGAAAAC---
AAAGCGCAGGAAATCTACATTGTTTGCTCT

>KF151483.1_Uncultured_marine_12

TCTACACGCCTTATTTTGCATTCCAAAGCTCAAAACACAATCATGGAA------ATG---
GCTGCTCAGGCAGGCACGGTTGAGGATCTCGAG---
CTAGAAGATGTTCTAAAGGTTGGATACGGCGACATTAAGTGTGTAGAAGCAGGCGGTCCAGAGCCCGGCGTTGGA
TGTGCCGGTCGCGGTGTTATTACT---GCAATTAATTTCCTTGAAGAAGAGGGTGCGTAT---GAAGACGATTTG---
GAC---------TTTGTTTTCTATGACGTTCTTGGCGACGTGGTCTGCGGCGGTTTTGCTATGCCAATTCGCGAAAAT---
AAGGCTCAAGAAATTTACATTGTCTGCTCT

>KF151819.1_GammaETSP2_marine_40

TCTACACGCCTTATTTTGCATTCCAAAGCTCAAAACACAATCATGGAA------ATG---
GCTGCTCAGGCAGGCACAGTTGAGGATCTCGAG---
CTCGATGACGTGCTTAAAGTTGGTTATGGTAACATTAAGTGTGTAGAAGCAGGCGGTCCAGAGCCCGGCGTTGGA
TGTGCCGGTCGCGGTGTTATTACT---GCGATTAATTTCCTTGAAGAAGAGGGTGCGTAT---GAGGACGATCTA---
GAC---------TTTGTTTTCTATGACGTTCTTGGCGACGTGGTCTGCGGCGGTTTTGCTATGCCAATTCGCGAAAAT---
AAGGCTCAAGAAATTTACATTGTCTGCTCT

>KF151493.1_Uncultured_marine_4

TCTACACGCCTTATTTTGCANTNNNNAGCTCAAAACACAATCATGGAA------ATG---
GCTGCTCAGGCAGGCACGGTTGAGGATCTCGAG---
CTCGATGACGTGCTTAAGGTTGGTTATGGTAACATTAAGTGTGTAGAAGCAGGCGGTCCAGAGCCCGGCGTTGGA
TGTGCCGGTCGCGGTGTTATTACT---GCAATTAATTTCCTTGAAGAAGAGGGTGCGTAT---GAAGAAGATCTA---
GAC---------TTTGTTTTCTATGACGTTCTTGGCGACGTGGTCTGCGGCGGTTTTGCTATGCCAATTCGCGAAAAT---
AAGGCTCAAGAAATTTACATTGTCTGCTCT

>HQ611835.1_Uncultured_marine_5

TCTACACGTCTCATCTTGCACTCCAAGGCTCAAAACACCATCATGGTG------ATG---
GCTGCTCAGGCAGGCACGGTTGAGGATTTAGAG---
CTGGAAGACGTTCTGAAGGTTGGATATGGCGACATTAAGTGTGTAGAAGCAGGCGGTCCAGAGCCCGGCGTTGGT
TGTGCCGGCCGCGGCGTTATCACT---GCGATTAACTTTCTTGAGGAAGAGGGTGCATAT---GAAGACGATCTA---
GAC---------TTTGTTTTCTATGATGTCCTTGGCGACGTGGTCTGCGGCGGTTTTGCTATGCCAATTCGTGAGAAC---
AAAGCTCAAGAGATCTACATTGTTTGCTCT

>HM210397.1_Gamma3_marine_21

TCCACGCGTCTTATCTTGCACTCCAAAGCCCAAAACACAATCATGGAA------ATG---
GCTGCCCAAGCAGGTACGGTTGAGGATTTAGAG---
CTTGATGACGTACTTAAGGTTGGCTACGGCGATATTAAGTGTGTAGAATCAGGCGGTCCAGAGCCCGGCGTTGGTT
GTGCCGGTCGCGGTGTTATTACC---GCGATTAACTTCCTTGACGAAGAAGGCGCATAT---GAAGACGATCTA---
GAC---------TTTGTTTTCTATGATGTTCTGGGCGACGTGGTCTGCGGCGGCTTTGCTATGCCAATTCGTGAAAAC---
AAAGCTCAGGAGATCTACATTGTTTGCTCT

>HQ611590.1_Uncultured_marine_13

TCTACGCGTCTTATCTTGCATTCCAAGGCCCAAAACACAATCATGGAA------ATG---
GCTGCCCAGGCAGGTACGGTTGAGGATTTAGAG---
CTTGATGACGTACTTAAGGTTGGCTACGGCGATATTAAGTGTGTAGAAGCGGGCGGTCCAGAGCCCGGCGTCGGG
TGTGCCGGTCGCGGTGTTATTACT---GCGATTAACTTTCTTGAAGAAGAGGGTGCATAT---GAAGACGATCTA---
GAC---------TTTGTTTTCTATGATGTTCTGGGCGACGTGGTCTGCGGCGGCTTTGCTATGCCAATTCGTGAAAAC---
AAAGCTCAAGAGATCTACATTGTTTGCTCT

>HF559538.1_Uncultured_wood_7

---ACCCGTCTGATCCTGCACGCCAAAGCGCAAAATACCATTATGGAA------CTG---
GCCGCGGAAGTCGGCTCGGTAGAAGATCTGGAG---
CTGGAAGATGTGCTTCAGATCGGCTATGCGGGTATCCGCTGCGCGGAATCAGGCGGCCCTGAGCCGGGTGTGGGC
TGCGCGGGACGCGGCGTTATCACC---GCCATTAACTTCCTGGAGGAAGAGGGCGCGTAT---GAAGATGACCTG---
GAC---------TTCGTATTCTATGACGTGCTTGGCGACGTGGCTTGCGGCGGGTTCGCCATGCCAATCCGCGAAAAT---
AAGGCGCAGGAAATCTATATCGTCTG----

>HF559482.1_Uncultured_wood_18

---ACCCGGCTGATTCTTCATGCCAAAGCACAAAACACCATTATGGAA------CTG---
GCCGCGGAAGTCGGCTCGGTGGAAGATCTGGAG---
CTGGAAGACGTGTTGCAGGTGGGTTATGCCGGCATCCGCTGCGCCGAATCAGGCGGTCCGGAGCCGGGCGTGGG
CTGCGCCGGCCGCGGCGTCATTACC---GCCATCAACTTCCTGGAAGAAGAGGGCGCTTAC---GAAGAGGATCTG---
GAC---------TTCGTGTTCTATGACGTACTGGGGGATGTGGTGTGCGGCGGGTTCGCCATGCCGATCCGTGAAAAC---
AAGGCGCAGGAAATCTATATCGTCTG----

>HF559531.1_Uncultured_wood_5

---ACCCGGCTGATCCTTCATGCCAAAGCACAAAATACCATTATGGAG------CTG---
GCCGCCGAAGTCGGCTCGGTTGAAGACCTGGAG---
CTGGAAGATGTGTTGCAGGTAGGGTATGCCGGCATCCGCTGCGCGGAATCAGGCGGCCCGGAGCCGGGTGTGGG
CTGTGCCGGCCGCGGTGTTATCACC---GCCATTAACTTCCTGGAGGAAGAGGGCGCGTAC---GAAGCAGATCTG---
GAC---------TTTGTTTTTTATGACGTATTGGGCGACGTGGTCTGCGGCGGGTTCGCCATGCCGATTCGTGAAAAC---
AAGGCGCAGGAAATCTATATCGTCTG----

>HF559969.1_Uncultured_wood_2

---ACCCGTCTAATCCTGCATGCCAAAGCACAAAATACCATTATGGAG------CTG---
GCCGCCGAAGTCGGCTCGGTTGAAGATCTGGAG---
CTGGAGGATGTATTGCAGGTGGGCTATGCCGGCATCCGTTGCGCGGAATCAGGCGGCCCGGAACCGGGTGTGGG
CTGTGCCGGCCGCGGTGTTATCACC---GCCATTAACTTCCTGGAGGAAGAGGGCGCGTAC---GAAGCAGATCTG---
GAC---------TTTGTTTTTTACGACGTATTGGGCGACGTGGTGTGCGGCGGGTTCGCCATGCCCATTCGTGAAAAC---
AAGGCGCAGGAAATCTATATCGTTTG----

>HF559962.1_Uncultured_wood_3

-----------------------AAAGCGCAAAACACCATTATGGAA------CTG---
GCCGCGGAAGTCGGCTCAGTGGAAGATCTGGAG---
CTGGAAGACGTGCTGCAGGTCGGTTATGCCGGCATCCGCTGCGCGGAATCAGGCGGCCCTGAGCCGGGTGTAGG
CTGTGCCGGACGCGGCGTCATTACC---GCCATCAACTTCCTGGAAGAAGAGGGCGCGTAC---GAAGCAGATCTG---
GAC---------TTTGTTTTCTATGACGTATTGGGCGACGTGGTTTGCGGCGGGTTCGCCATGCCGATTCGTGAAAAC---
AAGGCACAGGAAATCTATATCGTCTG----

>HF559964.1_Uncultured_wood_3

-----------------------AAAGCGCAAAACACCATTATGGAA------CTG---
GCCGCTGAAGTCGGCTCAGTGGAAGACCTGGAG---
CTGGAAGACGTCTTGCAGGTCGGTTATGCCGGCATCCGCTGCGCGGAATCAGGCGGCCCTGGGCCGGGTGTGGG
CTGTGCCGGACGCGGCGTGATTACC---GCCATTAACTTCCTGGAGGAGGAGGGCGCGTAC---GAAGCAGATCTG---
GAT---------TTTGTCTTTTATGACGTATTGGGCGACGTGGTATGCGGCGGGTTCGCCATGCCGATTCGTGAAAAT---
AAGGCGCAGGAAATCTATATCGTCTG----

>EF208162.1_Uncultured_soil_2

TCCACTCGCCTTATCCTGCATGCAAAAGCACAGAACACCATTTTGGAA------ATG---
GCGGCTGAAGTGGGCTCTGTAGAAGACCTTGAG---
CTGGAAGATGTTCTGCAAATTGGTTACGGCAACGTCCGTTGTGCTGAATCAGGTGGCCCGGAGCCGGGTGTGGGT
TGTGCTGGTCGCGGCGTTATTACA---GCTATCAACTTCCTGGAAGAGGAAGGCGCTTAT---GAAGACGACCTG---
GAC---------TTTGTGTTTTATGACGTACTGGGCGACGTGGTATGCGGCGGTTTCGCCATGCCAATTCGCGAAAAC---
AAAGCACAGGAAATCTACATTGTTTGTTCT

>DQ431161.1_Raoultella

TCCACGCGTCTTATTCTTCACGCGAAAGCGCAGAACACCATTATGGAA------ATG---
GCGGCGGAAGTCGGTTCTGTCGAAGACCTGGAG---
TTAGAGGACGTGTTGCAAATCGGCTTCGGTGATGTCCGTTGCGCTGAGTCCGGCGGTCCGGAGCCAGGCGTGGGC
TGTGCAGGTCGTGGTGTTATTACC---GCCATCAACTTCCTCGAAGAAGAGGGCGCGTAT---GTCCCGGACCTC---
GAC---------TTTGTGTTCTACGACGTGCTCGGCGACGTTGTGTGCGGCGGGTTCGCCATGCCTATCCGTGAAAAT---
AAAGCGCAGGAAATCTACATCGTCTGCTC-

>AY159597.1_Uncultured_root_2

TCAACCCGTTTGATTCTGCACGCCAAAGCGCAGAACACCATTATGGAG------ATG---
GCTGCGGAGGTCGGTTCGGTTGAAGATCTCGAA---
TTGGAAGATGTGCTGCAAATCGGTTACGGCAACGTACGTTGTGCAGAATCAGGTGGCCCAGAGCCGGGCGTTGGC
TGTGCTGGCCGCGGCGTTATCACC---GCCATCAACTTCCTTGAAGAGGAAGGTGCCTAT---GTCGACGACCTC---
GAC---------TTCGTGTTCTATGACGTGCTGGGGGACGTGGTATGCGGCGGCTTCGCGATGCCGATCCGTGAAAAC---
AAGGCGCAGGAAATCTACATCGTGTGCTC-

>CP003406.1_Rahnella_6

TCCACCCGCCTTATTCTGCATGCGAAAGCACAGAACACCATCATGGAA------ATG---
GCCGCTGAAGTGGGTTCCGTGGAAGATCTGGAG---
CTGGAAGATGTGATGCAAATCGGCTATGGCGGCGTGCGCTGTGCGGAATCAGGTGGCCCTGAGCCTGGTGTGGG
TTGTGCCGGACGCGGGGTGATAACC---GCCATCAACTTCCTCGAAGAAGAAGGCGCATAT---GTACCGGATCTG---
GAT---------TTTGTGTTTTACGACGTATTGGGCGACGTGGTATGCGGCGGTTTCGCGATGCCGATCCGCGAAAAT---
AAAGCGCAGGAGATCTACATCGTGTGCTC-

>AY242355.1_Klebsiella_PCRreagent_49

TCCACCCGTCTGATCCTCCACGCTAAAGCCCAGAACACCATCATGGAG------ATG---
GCGGCGGAAGTGGGCTCGGTCGAGGATCTGGAG---
CTCGAAGACGTTCTGCAAATCGGCTATGGCGATGTCCGTTGCGCCGAATCCGGCGGCCCGGAGCCAGGCGTCGGC
TGCGCCGGACGCGGGGTGATCACC---GCCATCAACTTCCTCGAGGAAGAAGGCGCCTAT---GAAGAAGATTTG---
GAT---------TTCGTCTTCTATGACGTCCTCGGCGACGTGGTCTGCGGCGGCTTCGCCATGCCGATCCGCGAAAAC---
AAAGCCCAGGAGATCTACATCGTCTGCTC-

>DQ425619.1_Uncultured_stem_15

TCTACCCGCCTGATCCTGCACGCCAAGGCACAGAACACCATTATGGAG------ATG---
GCCGCGGAAGTGGGCTCGGTGGAGGACCTCGAG---
CTCGAAGATGTCCTGCAAATCGGCTACGGCGACGTGCGCTGCGCGGAATCTGGCGGCCCGGAGCCAGGTGTCGGC
TGCGCGGGGCGCGGGGTGATTACC---GCAATCAACTTCCTCGAGGAGGAAGGCGCCTAC---GAGGACGATCTC---
GAC---------TTCGTGTTCTATGACGTGCTCGGGGACGTGGTCTGCGGCGGCTTCGCGATGCCGATCCGTGAAAAC---
AAGGCCCAGGAAATCTACATCGT-------

>GU196851.1_Uncultured_marine dinoflagellate_5

TCCACCCGCCTGATCCTGCACGCTAAAGCACAGAACACCATTATGGAG------ATG---
GCTGCGGAAGTGGGCTCGGTGGAGGACCTCGAG---
CTCGAAGATGTCCTGCAAATCGGCTACGGCGACGTGCGCTGTGCAGAATCAGGCGGCCCGGAGCCAGGCGTCGGC
TGCGCGGGTCGCGGGGTGATTACC---GCAATCAACTTCCTCGAGGAGGAAGGTGCCTAC---GAGGACGATTTC---
GAC---------TTCGTGTTCTATGACGTGCTCGGGGACGTGGTCTGCGGCGGCTTCGCGATGCCAATCCGTGAAAAT---
AAGGCCCAGGAGATCTATATCGTCTGCTC-

>HQ204257.1_Enterobacter

TCCACCCGCCTGATCCTGCACGCTAAAGCACAGAACACCATCATGGAG------ATG---
GCAGCGGAGGTCGGTTCGGTCGAGGACCTCGAA---
CTTGAAGACGTTCTGCAAATTGGCTACGGCGACGTGCGCTGCGCCGAATCCGGCGGCCCGGAGCCAGGTGTCGGC
TGTGCAGGGCGCGGCGTTATCACG---GCGATCAACTTTCTTGAAGAAGAAGGCGCCTAC---GAAGACGATCTC---
GAC---------TTCGTCTTCTATGACGTGCTGGGCGACGTGGTCTGCGGCGGTTTCGCCATGCCGATCCGCGAAAAC---
AAAGCCCAGGAGATCTACATCGTCTGCTCT

>FJ593867.1_Klebsiella_PCRreagent_29

TCCACCCGCCTGATCCTGCACGCTAAAGCACAGAACACCATTATGGAG------ATG---
GCAGCGGAAGTCGGCTCGGTCGAGGATCTCGAG---
CTCGAAGACGTGCTGCAAATTGGCTACGGCGACGTGCGCTGCGCGGAATCCGGCGGCCCGGAGCCAGGCGTCGG
CTGCGCGGGGCGCGGCGTGATCACG---GCGATCAACTTTCTTGAAGAAGAAGGCGCCTAC---GAGGACGATCTC---
GAT---------TTTGTCTTCTATGACGTGCTCGGCGACGTGGTCTGCGGCGGCTTCGCCATGCCAATCCGCGAAAAC---
AAAGCCCAGGAGATCTACATCGTCTGCTC-

>DQ425446.1_Uncultured_stem_8

---ACCCGCCTGATCCTGCACGCCAAGGCACAGAACACCATTATGGAG------ATG---
GCCGCGGAAGTCGGCTCGGTCGAGGACCTCGAA---
CTCGAAGACGTTCTGCAAATTGGCTACGGCGACGTGCGCTGCGCGGAATCCGGCGGCCCGGAGCCAGGCGTCGG
CTGTGTGGGACGCGGCGTGATCACG---GCGATCAACTTTCTTGAAGAAGAAGGCGCCTAC---GAGGACGATCTC---
GAT---------TTCGTGTTCTATGACGTGCTCGGCGACGTGGTCTGCGGCGGCTTCGCCATGCCGATCCGCGAGAAC---
AAGGCCCAGGAAATCTACATCGTT------

>HM042879.1_PCRreagent_15

TCCACCCGCCTGATCCTGCACGCCAAAGCACAGAACACCATTATGGAG------ATG---
GCCGCGGAAGTCGGCTCGGTCGAGGACCTCGAA---
CTCGAAGACGTTCTGCAAATTGGCTACGGCGACGTGCGCTGCGCGGAATCCGGCGACCCGGAGCCAGGCGTCGGC
TGTGCGGGACGCGGCGTGATCACG---GCGATCAACTTTCTTGAAGAAGAAGGCGCCTAC---AAGGACGATCTC---
GAT---------TTCGTGTCCTATGACGTGCTCGGCGACGTGGTCTGCGGTGGCTTCGCCATGCCGATCCGCGAAAAC---
AAAGCCCAGGAGATCTACATCGTCTGCTCC

>FJ593770.1_Pantoea_2

TCCACCCGTTTGATCCTGCATGCCAAAGCTCAAAACACCATTATGGAA------ATG---
GCCGCAGAAGTCGGTTCCGTGGAAGACCTGGAG---
CTGGAAGATGTGCTGCAAATTGGTTATGGCGACGTGCGCTGTGCCGAATCCGGTGGCCCGGAGCCAGGCGTTGGC
TGTGCCGGTCGAGGCGTGATCACG---GCTATCAATTTCCTTGAAGAAGAAGGTGCCTAC---GTGCCCGATCTA---
GAC---------TTCGTTTTCTATGACGTGTTGGGGGACGTGGTGTGCGGTGGTTTTGCCATGCCTATCCGTGAAAAC---
AAAGCGCAGGAGATCTACATCGTCTGCTC-

>EU938523.1_Erwinia_5

TCCACCCGTTTGATTCTGCATGCCAAAGCGCAGAACACCATTATGGAG------ATG---
GCGGCGGAAGTGGGTTCCGTGGAAGACCTCGAG---
CTGGAAGATGTGCTGCAAATCGGTTATGGCGACGTGCGCTGCGCCGAATCTGGCGGTCCGGAACCCGGCGTTGGC
TGTGCTGGACGCGGCGCCATTACC---GCCATCAACTTCCTCGAAGAAGAAGGAGCCTAC---GTACCCGATCTC---
GAT---------TTTGTTTTCTATGACGTACTGGGTGACGTGGTGTGCGGTGGCCTTGCCATGCCCATCCGTGAAAAC---
AAAGCTCAGGAGATCTACATCGTCTGTTC-

>DQ426002.1_Uncultured_stem_8

---ACCCGCCTGATCCTGCACGCCAAGGCGCAGAACACCATCATGGAG------ATG---
GCGGCGGAAGTGGGTTCCGTGGAAGACCTGGAG---
CTGGAAGATGTGCTGCAAATCGGTTACGGCAACGTGCGCTGTGCTGAGTCTGGCGGTCCGGAGCCAGGCGTAGGC
TGTGCAGGACGCGGCGTGATTACC---GCCATCAACTTTCTCGAAGAGGAAGGTGCCTAC---GTACCCGATCTC---
GAT---------TTTGTCTTCTATGACGTACTGGGTGACGTGGTATGCGGTGGCTTTGCCATGCCCATCCGTGAAAAC---
AAGGCCCAGGAAATCTACATCGT-------

>FJ593777.1_Pantoea_4

TCCACCCGTTTGATCCTGCACGCCAAAGCGCAGAACACCATTATGGAG------ATG---
GCGGCGGAAGTGGGTTCCGTGGAAGACCTGGAG---
CTGGAAGATGTGCTGCAAATCGGGTACGGCAACGTGCGCTGTGCTGAGTCCGGCGGCCCGGAGCCAGGCGTTGG
CTGTGCAGGACGCGGCGTGATTACC---GCCATCAACTTCCTCGAAGAAGAAGGTGCCTAC---GTACCCGATCTC---
GAT---------TTTGTCTTCTATGACGTGTTGGGTGACGTGGTATGCGGTGGTTTTGCCATGCCCATCCGTGAAAAC---
AAAGCCCAGGAGATCTACATCGTCTGTTC-

>KF872883.1_Uncultured_root_4

TCCACGCGATTGATCCTGCATGCGAAAGCGCAGAACACCATTATGGAG------ATG---
GCTGCCGAAGTCGGCTCCGTGGAAGATCTGGAA---
TTAGAAGACGTGCTGCAAATCGGTTACGGCGGCGTACGCTGCGCGGAATCCGGCGGCCCGGAGCCAGGCGTTGG
CTGTGCGGGTCGTGGGGTAATCACC---GCGATTAACTTCCTGGAAGAAGAAGGCGCTTAC---GTTCCGGATCTG---
GAT---------TTTGTTTTTACGATGTGCTGGGCGACGTGGTATGCGGTGGTTTCGCCATGCCGATTCGTGAAAAC---
AAAGCGCAGGAGATCTACATCGTCTGCTC-

>CP005991.1_Enterobacter_2

TCCACGCGTTTGATCCTGCATGCGAAAGCGCAGAACACCATTATGGAG------ATG---
GCTGCTGAAGTTGGCTCCGTGGAAGACCTGGAA---
TTAGAAGACGTGCTGCAAATCGGTTACGGCGGCGTGCGCTGCGCAGAATCCGGCGGTCCGGAGCCAGGCGTGGG
TTGTGCCGGTCGTGGGGTGATCACC---GCGATTAACTTCCTCGAAGAAGAAGGCGCTTAC---GTGCCGGATCTC---
GAT---------TTTGTTTTCTACGACGTGCTGGGCGATGTGGTATGCGGTGGTTTCGCCATGCCGATTCGTGAAAAC---
AAAGCGCAGGAGATCTACATCGTTTGCTCT

>CP007215.2_Enterobacter_root

TCCACGCGTTTGATCCTGCATGCGAAAGCGCAGAACACCATTATGGAG------ATG---
GCCGCCGAAGTCGGCTCCGTCGAAGACCTGGAA---
TTAGAAGACGTACTGCAAATCGGTTACGGCGGCGTGCGCTGCGCGGAATCCGGTGGGCCGGAGCCAGGTGTGGG
CTGTGCCGGTCGTGGCGTGATCACC---GCGATTAACTTCCTCGAAGAAGAAGGCGCTTAC---GTTCCGGATCTG---
GAT---------TTTGTTTTCTACGACGTGCTGGGCGACGTGGTATGCGGTGGTTTCGCCATGCCGATTCGTGAAAAC---
AAAGCGCAGGAGATCTACATCGTTTGCTC-

>HQ204232.1_Enterobacter

TCCACCCGTCTGATCCTGCACGCGAAAGCGCAGAACACCATTATGGAG------ATG---GCCGCCGAAGTGGGTTCTGTGGAAGACCTCGAA---CTGGAAGACGTGCTGCAAATCGGTTACGGCGGCGTGCGCTGTGCGGAATCCGGCGGCCCGGAACCAGGCGTGGGCTGTGCAGGACGTGGTGTTATCACC---GCCATCAACTTCCTTGAAGAAGAAGGCGCCTAT---GTCAGCGACCTC---GAC---------TTTGTCTTCTATGACGTCCTCGGCGACGTGGTTTGCGGCGGGTTCGCCATGCCAATTCGTGAAAAC---AAAGCGCAAGAGATCTATATCGTCTGCTC-

>KF872862.1_Uncultured_root

TCCACCCGTCTGATCCTGCATGCGAAAGCGCAGAACACCATTATGGAG------ATG---GCCGCCGAAGTGGGTTCAGTGGAAGACCTTGAA---CTGGAAGATGTGCTGCAAATCGGTTACGGCGGCGTGCGTTGTGCAGAATCCGGCGGCCCGGAGCCAGGCGTGGGTTGTGCAGGCCGCGGCGTTATTACC---GCCATTAACTTCCTTGAAGAAGAAGGCGCCTAT---GTCAGCGACCTC---GAC---------TTTGTCTTCTATGACGTCCTCGGTGACGTGGTCTGCGGCGGGTTCGCCATGCCGATTCGTGAAAAC---AAAGCGCAAGAGATCTATATCGTCTGCTC-

>KF872854.1_Uncultured_root_4

TCCACCCGTCTGATCCTGCATGCGAAAGCGCAGAACACTATTATGGAG------ATG---GCGGCTGAAGTCGGTTCCGTGGAAGACCTGGAG---CTGGAGGATGTGCTGCAAATCGGTTACGGCGACGTGCGCTGTGCAGAATCCGGCGGCCCGGAGCCAGGTGTTGGCTGTGCAGGTCGTGGGGTCATTACC---GCTATTAACTTCCTTGAAGAAGAAGGCGCCTAT---GTTCCCGATCTC---GAT---------TTCGTCTTTTACGACGTGCTGGGCGACGTGGTGTGCGGGGGTTTCGCCATGCCAATCCGCGAAAAT---AAAGCGCAGGAGATCTACATCGTCTGCTC-

>AJ716254.1_Uncultured_mine spoils_4

TCCACCCGTCTGATCCTGCATGCGAAAGCGCAGAACACCATTATGGAG------ATG---GCGGCTGAAGTCGGCTCCGTGGAAGACCTGGAA---CTGGAAGATGTGCTGCAAATTGGTTACGGCGATGTGCGTTGCGCAGAATCCGGCGGCCCGGAACCCGGCGTTGGCTGTGCCGGTCGCGGAGTCATTACC---GCCATCAACTTCCTGGAAGAAGAAGGCGCTTAT---GTTCCCGACCTC---GAT---------TTCGTCTTTTATGACGTGCTGGGCGACGTGGTGTGCGGGGGGTTCGCCATGCCAATTCGCGAAAAC---AAGGCGCAGGAGATCTACATCGTCTGCTC-

>HQ204230.1_Enterobacter_4

TCCACCCGTCTGATCCTGCACGCGAAAGCGCAGAACACCATTATGGAG------ATG---GCGGCTGAAGTCGGCTCCGTGGAAGATCTGGAA---CTGGAAGATGTGCTGCAAATCGGTTACGGCGACGTCCGCTGTGCTGAATCCGGCGGCCCGGAACCAGGCGTTGGCTGTGCCGGTCGCGGGGTGATTACC---GCCATCAACTTCCTGGAAGAAGAAGGCGCCTAT---GTTCCCGACCTC---GAT---------TTCGTCTTTTATGACGTGCTGGGCGACGTGGTGTGCGGTGGGTTCGCCATGCCGATTCGCGAAAAC---AAAGCGCAAGAGATCTACATCGTCTGCTC-

>JN698220.1_Enterobacter_root

---ACCCGTCTGATCCTGCATGCGAAAGCACAGAACACCATTATGGAG------ATG---GCGGCTGAAGTCGGCTCCGTGGAAGACCTGGAA---CTGGAAGATGTGCTGCAAATCGGCTACGGCGACGTGCGCTGCGCAGAATCCGGCGGCCCGGAGCCAGGCGTTGGCTGTGCTGGTCGCGGGGTGATTACC---GCCATCAACTTCCTGGAAGAAGAAGGCGCCTAT---GTTCCCGACCTC---GAT---------TTCGTCTTTTACGACGTGCTGGGCGACGTGGTGTGCGGGGGGTTCGCCATGCCGATTCGCGAAAAC---AAAGCCCAGGAAATCTACATCGT-------

>GQ441477.1_Uncultured_marine mat_3

------------ATTTTACATTCTAAAGCGCAAACTACGGTTATGCAT------TTA---GCCGCAGAAGCGGGTACAGTAGAAGATTTAGAA---TTAGATGATGTATTATCAGTGGGTTACGGTGGTATTCGTTGCGTTGAATCAGGCGGTCCTGAACCCGGTGTTGGTTGCGCAGGACGCGGCGTTATTACC---GCAATTAACTTCTTGGAAGAAGAAGGTGCTTAC---GAAGAAGATTTA---GAT---------TTCGTATTTTATGATGTATTAGGTGACGTGGTCTGCGGTGGCTTTGCAATGCCTATTCGTGAAAAT---AAAGCCCAAGAAATTTACATTGT-------

>GU192739.1_Uncultured_marine mat_14

------------ATCCTTCACTCTAAAGCTCAAACCACTGTTATGCAC------TTA---GCAGCAGAAGCTGGTACGGTGGAAGATTTAGAA---TTAGAAGATGTATTATCTGTAGGTTATGGTGGGATTAAATGCGTTGAATCCGGTGGCCCAGAGCCTGGTGTTGGTTGTGCTGGACGTGGAGTTATTACT---GCGATTAATTTCCTAGAAGAAGAAGGTGCTTAC---GAAGAAGATTTA---GAT---------TTCGTATTCTACGATGTACTAGGGGACGTAGTATGCGGTGGCTTTGCTATGCCTATTCGCGAAAAT---AAAGCCCAAGAAATTTATATCGT-------

>GQ464084.1_Uncultured_fungus_11

TCTACTCGTTTGATCCTGCACTCCAAAATGCAGGTAACAGTAATGCAT------TTA---GCAGCAGAACATGGTTCTGTCGATGATCTGGAA---TTGGAAGACGTATTACAGGTCGGCTTCGGCGATGTGAAATGCGTTGAGTCAGGTGGCCCGGAGCCAGGAGTGGGTTGTGCCGGTCGTGGCGTTATCACT---GCGATTAACTTCCTGGAAGAAGAAGGTGCTTAC---AGTGATGATCTC---GAC---------TTCGTATTTTATGACGTATTAGGTGACGTTGTTTGCGGTGGTTTCGCGATGCCAATTCGTGAAAAC---AAAGCGCAAGAGATCTACATCGTTTGCTCT

>CP001968.1_Denitrovibrio

TCAACAAGACTTATACTTCACTCTAAAGCACAATCAACTATAATGGAG------CTC---GCTGCTGAAGCAGGCTCAGTTGAAGACCTTGAA---CTTGATGACGTTCTCAAAGCCGGTTATCTTGATATACGCTGCGTAGAGGCAGGCGGTCCGGAACCCGGTGTCGGCTGTGCTGGTCGTGGTGTTATTACC---GCTATCAACTTCCTTGAGGAAGAGGGTGCATAC---GAAGAAGACCTC---GAT---------TTCGTTTCATATGACGTTCTCGGTGACGTTGTTTGCGGTGGTTTCGCAATGCCTATTCGTGAGGGT---AAAGCTCAGGAGATATACATTGTT-------

>AF013025.1_Microcoleus

TCCACTCGTTTGATTCTACACGCTAAAGCACAAACCACCGTGCTACAC------ATG---
GCTGCTGAAAGAGGCGCCATTGAAGATGTAGAA---
CTCGAAGAAGTACTCAAAGCTGGCTACGCTGGCATCCAGTGCGTTGAGTCCGGTGGTCCAGAGCCTGGAGTTGGC
TGCGCTGGTCGGGGTATCATCACT---GCCATTAACTTCCTCGAAGAAAATGGTGCTTAC---GAA---GACCTA---GAC-
--------TTCGTTAGCTATGACGTACTAGGTGACGTTGTTCGCGGTGGTTTTGCAATGCCAATTCGCGAAGGC---
AAAGCCCAAGAAATCTACATCGTT------

>EU052588.1_Uncultured_South China Sea_488

---GGCGCAGTTGAAGATGTAGAA---
CTAGACGAAGTATTGAAGCCAGGTTTCGGTGGCATTAAGTGTGTTGAATCTGGTGGTCCTGAGCCTGGTGTAGGTT
GCGCTGGCCGTGGTATTATTACT---GCCATCAACTTCCTAGAAGAAGAAGGAGCTTAT---ACA---GATCTA---GAT---
------TTCGTAAGCTATGACGTACTAGGTGACGTTGTTTGCGGTGGATTTGCAATGCCTATCCGTGAAAAC---
AAAGCTCAAGAAATCTACATCGTATGTTC-

>JN094200.1_Uncultured_South China Sea_18

---GCTGCTGAGCGTGGTGCGGTTGAAGACGTAGAA---
CTAGATGAAGTATTGAAACCAGGTTTCGGTGGCATTAAGTGTGTTGAGTCTGGTGGTCCTGAGCCTGGTGTAGGTT
GCGCTGGCCGTGGTATTATTACT---GCTATCAACTTCCTAGAAGAAGAAGGAGCTTAT---ACA---GATCTA---GAT----
-----TTCGTAAGCTATGACGTACTAGGTGACGTTGTTTGCGGTGGATTTGCAATGCCTATCCGTGAAAAC---
AAAGCTCAAGAAATCTACATCGTATGTTC-

>AY115593.1_Lyngbya_17

TCTACTCGTCTAATCTTAAACGCTAAAGCTCAAACCACTGTACTTCAC------GTT---
GCTGCAGAGCGCGGTGCAGTTGAAGACGTTGAA---
CTAGAGGAAGTATTGAAAGAAGGCTTCGCTGGTATCAAGTGCGTTGAGTCTGGTGGTCCTGAGCCTGGAGTTGGT
TGCGCAGGTCGTGGTATTATCACA---GCTATCAACTTCCTAGAAGAAGAAGGTGCTTAT---ACC---GATCTA---GAT--
-------TTCGTAAGTTATGACGTACTAGGTGACGTTGTTTGCGGTGGATTTGCAATGCCTATCCGTGAAAAC---
AAAGCTCAAGAAATCTACATCGTATGTTC-

>KC992980.1_Okeania_marine_4

---GCTGCAGAGCGCGGTGCAGTTGAAGATGTAGAG---
CTAGAAGAAGTATTGAAAGATGGTTTCGCTGGCATCAAGTGCGTTGAGTCTGGTGGTCCTGAGCCTGGAGTAGGT
TGTGCAGGTCGTGGTATTATCACT---GCTATCAACTTCCTAGAAGAAGAGGGTGCTTAT---ACC---GATCTA---GAT--
-------TTCGTAAGTTATGACGTACTAGGTGACGTTGTTTGCGGTGGATTTGCAATGCCTATCCGTGAAAAC---
AAAGCTCAAGAAATCTACATCGTATGTTC-

>JF896695.1_Uncultured_marine seagrass_11

---GAAGATGTAGAA---
CTAGACGAAGTATTGAAAGATGGTTTCGCAGGCATCCGTTGCGTTGAGTCTGGTGGTCCTGAGCCTGGAGTTGGTT
GTGCAGGTCGTGGTATTATCACT---GCTATCAACTTCCTAGAAGAAGAAGGTGCTTAT---CCA---GATTTA---GAT----

-----TTCGTAAGCTATGACGTACTAGGTGACGTTGTTTGCGGTGGATTTGCAATGCCTATCCGTGAAAAC---
AAAGCTCAAGAAATCTACATCGT-------

>KC992984.1_Okeania_marine_6

--GCTGCAGAGCGTGGTGCAGTTGAAGACGTAGAA---
CTAGACGAAGTATTGAAAGATGGTTTTGCAGGCATCCGTTGCGTTGAGTCTGGTGGTCCTGAGCCTGGTGTAGGTT
GCGCAGGTCGTGGTATTATCACT---GCTATCAACTTCCTAGAGGAAGAAGGTGCTTAT---ACA---GACCTA---GAT---
------TTCGTAAGTTATGACGTACTAGGTGACGTTGTTTGCGGTGGATTTGCAATGCCTATCCGTGAAAAC---
AAAGCTCAAGAAATCTACATCGTATGTTC-

>CP002432.1_Desulfurispirillum

TCCACCCGTCTGATTCTGCATGCCAAGGCACAGTCAACCGTTATGGAA------ATG---
GCGGCTGAGGCTGGTTCGGTTGAGGATCTGGAG---
CTGGATGATGTGCTCAAGACGGGCTACCTTGGCATCCGCTGTGTTGAGGCGGGTGGTCCTGAGCCTGGTGTTGGC
TGCGCTGGTCGCGGTGTTATCACG---GCCATCAACTTCCTGGAAGAGGAAGGCGCCTAC---GAAGAAGACCTG---
GAT---------TTCGTTTCCTACGACGTTCTGGGTGACGTAGTGTGTGGTGGTTTCGCCATGCCCATCCGCGAAGGC---
AAAGCTCAGGAGATCTACATTGT-------

>GQ441384.1_Uncultured_marine mat_2

TCTACCCGGCTCATTCTCCACTCCAAGGCTCAGAATACCATTATGGAA------ATG---
GCTGCTGAGGCCGGCACAGTAGAAGATTTAGAA---
TTAGAAGATGTATTAAAAACAGGTTATGGCGGCATCAAGTGTGTCGAATCCGGTGGGCCGGAACCGGGTGTCGGT
TGTGCCGGACGGGGTGTTATCACC---GCCATTAACTTTCTTGAAGAAGAAGGCGCTTAC---GAAGATGATCTC---
GAC---------TTCGTTTTCTATGATGTTCTGGGCGACGTTGTCTGCGGTGGCTTTGCCATGCCCATTCGTGAAAAC---
AAAGCACAAGAAATCTATATCGT-------

>FJ502283.1_Uncultured_meromictic lake_2

TCCACCCGTTTGATTCTACACGCAAAAGCGCAGAACTCGATCATGGAA------ATG---
GCTGCCGAAGCCGGTTCCGTCGAAGACCTCGAA---
CTGGAAGACGCGCTCAAAGTCGGTTACCGCGACATCAAGTGCGTGGAATCCGGCGGTCCAGAGCCGGGTGTCGGC
TGCGCAGGTCGCGGCGTGATTACC---GCAATTAACTTCCTCGAAGAAGAAGGCGCGTAT---ACCGATGATTTA---
GAT---------TTCGTATTCTATGACGTATTGGGCGACGTAGTTTGCGGCGGATTCGCCATGCCGATTCGTGAGAAT---
AAAGCGCAAGAGATTTACATCGTTTGCTC-

>EF202525.1_Thiorhodospira_2

TCTACGCGCCTGATTTTACACGCTAAGGCTCAGAATACCATCATGCAG------ATG---
GCCGCCGATGCGGGGAGCGTAGAAGATCTGGAG---
CTGGAAGATGTATTGAAAACCGGTTATGGCGGTATCAAGTGCGTTGAATCCGGTGGCCCAGAGCCGGGTGTTGGG
TGTGCCGGTCGTGGGGTTATCACC---GCGATTAACTTCCTGGAAGAGGAAGGCGCCTAC---GAAGAAGATCTC---

GAT---------TTTGTATTTTATGACGTGCTCGGTGACGTGGTGTGCGGTGGCTTTGCCATGCCGATTCGTGAAAAC---
AAGGCCCAGGAAATTTATATCGT-------

>JN638632.1_Uncultured_Black Sea_15

TCCACTCGTCTGATCCTGCATGCCAAGGCGCAGGATACGGTGATGCAT------CTG---
GCCGCCGAGGCGGGCAGTGTCGAGGACCTGGAG---
CTGGAGGATGTACTGTCGGTCGGTTATCGCGGTATCAAGTGCGTTGAGTCGGGTGGTCCTGAGCCAGGTGTCGGT
TGTGCTGGTCGTGGGGTTATCACC---GCCATTAACTTCCTGGAAGAGGAGGGGGCCTAT---GAAGAGGACCTC---
GAC---------TTCGTATTCTATGACGTTCTGGGTGACGTGGTGTGTGGTGGCTTCGCCATGCCCATCCGCGAGAAC---
AAGGCGCAGGAGATCTACATCGT-------

>GQ441376.1_Uncultured_marine mat_53

TCCACGCGTCTGATCCTGCACTCCAAGGCGCAGGACACCATCATGCAG------ATG---
GCGGCTGACGTTGGCTCGGTCGAGGATCTGGAG---
CTTGAGGATGTAATGGCGACTGGTTATGGCGGCATCAAGTGTGTTGAGTCCGGTGGCCCTGAGCCTGGTGTGGGC
TGTGCCGGCCGTGGCGTTATTACC---GCGATCAACTTCCTCGAAGAGGAAGGCGCCTAT---GAAGACGATCTG---
GAC---------TTCGTCTTCTACGACGTGCTCGGCGACGTGGTCTGTGGTGGTTTTGCCATGCCAATTCGCGAAAAC---
AAGGCGCAGGAGATCTACATCGTCTGCTC-

>GU192833.1_Uncultured_marine mat_5

TCGACCCGTCTGATCCTGCACTCCAAGGCGCAGGACACCATCATGCAG------ATG---
GCGGCTGACGCAGGCTCAGTTGAGGATCTGGAG---
CTTGAGGATGTAATGGCGACTGGTTATGGCGGCATCAAGTGTGTTGAGTCCGGTGGCCCTGAGCCTGGTGTGGGC
TGTGCCGGCCGTGGCGTTATTACC---GCGATCAACTTCCTCGAAGAGGAAGGCGCCTAT---GAAGACGATCTG---
GAC---------TTCGTTTTCTACGACGTGCTCGGCGACGTGGTCTGTGGTGGTTTCGCCATGCCCATTCGCGAAAAC---
AAGGCGCAGGAGATCTACATCGTCTGCTC-

>GU193858.1_Uncultured_marine mat_2

TCGACCCGTCTGATTCTGCACTCCAAGGCGCAGGACACCATCATGCAG------ATG---
GCGACTGACGCAGGCTCAGTTGAGGATCTGGAG---
CTTGAGGATGTCATGGCGACCGGTTACGGCGGCATCAAGTGCGTTGAATCCGGTGGCCCTGAGCCAGGTGTCGGC
TGCGCTGGCCGCGGCGTCATCACC---GCGATCAACTTCCTGGAAGAAGAAGGCGCCTAC---GAGGAAGCACTG---
GAC---------TTCGTCTTCTACGACGTGCTCGGCGACGTGGTCTGCGGTGGTTTCGCCATGCCAATTCGCGAGAAC---
AAGGCTCAGGAGATCTATATCGTCTGCTC-

>GQ441450.1_Uncultured_marine mat_17

TCGACCCGTCTGATCCTGCACTCCAAGGCGCAGGACACCATCATGCAG------ATG---
GCGGCTGACGCGGGCTCAGTTGAGGATCTGGAG---
CTTGAGGATGTTATGGCGACCGGTTATGGCGGCATCAAGTGCGTTGAGTCCGGTGGCCCTGAGCCAGGTGTTGGC
TGCGCTGGCCGCGGCGTTATTACC---GCGATCAACTTCCTGGAAGAAGAAGGCGCCTAC---GAGGAAGAGCTG---

GAC---------TTCGTCTTCTACGACGTACTTGGCGACGTGGTCTGTGGTGGTTTCGCCATGCCGATCCGCGAAAAC---AAGGCGCAGGAAATCTACATCGTCTGCTC-

>GU192816.1_Uncultured_marine mat_71

TCGACCCGTCTGATTCTGCACTCCAAGGCGCAGGACACCATCATGCAG------ATG---GCGGCTGACGCTGGCTCGGTTGAGGATCTGGAG---CTTGAGGATGTCATGGCGACCGGTTATGGCGGCATCAAGTGTGTTGAGTCCGGTGGCCCTGAGCCAGGTGTTGGCTGCGCTGGCCGCGGCGTCATCACC---GCGATCAACTTCCTGGAAGAAGAGGGTGCCTAC---GAAGAAGAGCTG---GAC---------TTCGTCTTCTACGACGTACTCGGCGACGTGGTCTGTGGTGGTTTCGCCATGCCGATCCGCGAAAAC---AAAGCGCAGGAGATCTATATCGTCTGCTC-

>GU193829.1_Uncultured_marine mat_9

TCGACCCGTCTGATCCTGCACTCCAAGGCGCAGGACACCATCATGCAG------ATG---GCGGCTGACGCGGGCTCAGTTGAGGATCTGGAG---CTTGAGGATGTTATGGCGACCGGTTATGGCGGCATCAAGTGTGTTGAGTCCGGCGGCCCTGAGCCTGGTGTTGGCTGCGCTGGCCGCGGCGTCATCACC---GCGATCAACTTCCTGGAAGAAGAAGGTGCCTAC---GAGGAAGACCTG---GAC---------TTCGTCTTCTACGACGTACTCGGTGACGTGGTCTGTGGTGGTTTCGCCATGCCGATCCGCGAGAAC---AAGGCGCAGGAGATCTATATCGTCTGCTC-

>GQ441369.1_Uncultured_marine mat_27

TCGACCCGTCTGATCCTGCACTCCAAGGCGCAGGACACCATCATGCAG------ATG---GCGGCTGACGCGGGCTCAGTTGAGGATCTGGAG---CTTGAGGATGTTATGGCGACCGGTTATGGCGGCATCAAGTGCGTTGAGTCCGGTGGCCCTGAGCCAGGTGTTGGCTGCGCTGGCCGCGGCGTCATCACT---GCGATCAACTTCCTGGAAGAGGAAGGTGCCTAT---GAAGAAGACCTG---GAC---------TTCGTCTTCTACGACGTGCTCGGCGACGTGGTCTGTGGTGGTTTCGCCATGCCAATTCGCGAGAAC---AAGGCGCAGGAGATCTATATCGTCTGCTC-

>GQ441410.1_Uncultured_marine mat_52

TCGACCCGTCTGATCCTGCACTCCAAGGCGCAGGACACCATCATGCAG------ATG---GCGGCTGACGCGGGCTCAGTTGAGGATCTGGAG---CTTGAGGATGTCATGGCGACCGGTTATGGCGGCATCAAGTGCGTTGAGTCCGGTGGCCCTGAGCCAGGTGTCGGCTGTGCTGGCCGCGGCGTCATCACT---GCGATCAACTTCCTGGAAGAAGAAGGTGCCTAT---GAAGAAGACCTG---GAC---------TTCGTCTTCTACGACGTACTCGGCGACGTGGTCTGCGGCGGTTTCGCCATGCCGATCCGCGAGAAC---AAGGCGCAGGAGATTTATATCGTCTGCTC-

>GU193057.1_Uncultured_marine mat_3

TCCACGCGCCTGATCCTGCATGCCAAGGCACAGAATACCATTATGGAG------ATG---GCCGCGGAGGCCGGTTCGGTCGAGGACCTGGAG---CTTGAAGACGTACTGAAGGTCGGCTATGGCGACATCAGGTGCGTTGAGTCCGGTGGTCCTGAGCCGGGTGTCGGCTGCGCAGGTCGCGGTATCATCACC---GCCATCAACTTCCTAGAAGAAGAAGGTGCCTAC---GAT---GATGTT---GAC-

--------TTAGTATCCTACGACGTATTGGGTGACGTTGTTTGCGGTGGATTCGCAATGCCTATCCGGGAAGGT---
AAAGCCCAAGAAATCTACATCGT-------

>JF897460.1_Uncultured_marine mat_2

TCGACCCGTCTGATCCTGCACTCCAAGGCGCAGGACACCATCATGCAG------ATG---
GCGGCTGACGCTGGCTCGGTTGAGGATCTGGAG---
CTTGAGGATGTCATGGCGACCGGTTATGGCGGCATCAAGTGCGTTGAGTCCGGCGGTCCAGAACCAGGTGTAGGC
TGCGCAGGTCGCGGTATCATCACC---GCCATCAACTTCCTAGAAGAAGAAGGTGCCTAC---GAT---GATGTT---GAC-
--------TTAGTATCCTACGACGTATTGGGTGACGTTGTTTGCGGTGGATTCGCAATGCCTATCCGGGAAGGT---
AAAGCCCAAGAAATCTACATCGT-------

>GU192734.1_Uncultured_marine mat_2

TCAACCCGTTTGATCCTGCACGCCAAGGCGCAGGACACCATTATGCAC------CTC---
GCCGCTGAAGCCGGCTCGGTGGAAGACCTGGAA---
CTTGAAGACGTCATGCGCGCTGGCTATGCCGACATCAAGTGCGTCGAGTCCGGCGGCCCGGAGCCTGGAGTTGGC
TGCGCTGGCCGCGGCGTCATCACC---GCGATCAACTTCCTCGAAGAAGAAGGCGCCTAT---GACGAAGAACTC---
GAT---------TTCGTCTTCTACGATGTACTCGGCGACGTCGCCTGCGGCGGCTTCGCGATGCCGATCCGCGAGAAC---
AAGGCGCAAGAGATCTACATCGT-------

>KF861190.1_Uncultured_soil_4

TCCACCCGTCTGATCCTTCACGCCAAGGCACAGAACACCATCATGCAT------CTG---
GCTGCAGAAGCCGGAAGCGTAGAAGACCTCGAG---
CTCGAGGATGTCCTCAAGACCGGCTACGGCGACATCCGCTGCGTCGAGTCGGGCGGCCCCGAACCCGGTGTTGGC
TGCGCTGGCCGCGGTGTCATCACC---GCGATCAACTTCCTGGAAGAGGAAGGAGCGTAC---GAGGGTGGACTG---
GAC---------TTCGTGTTCTATGACGTGCTGGGTGACGTGGTTTGCGGGGGGTTCGCTATGCCCATCCGCGAAAAC---
AAGGCTCAAGAGATCTACATCGT-------

>EF568432.1_Uncultured_Mediterranean_4

TCAACCCGTCTGATTCTGCATTCCAAGGCGCAGACCACCGTGATGCAC------CTG---
GCGGCCGAGGCTGGTACGGTAGAGGATCTGGAG---
CTGGAGGATGTACTGTCTGTCGGTTACGGCGATGTTAAGTGTGTTGAGTCGGGCGGTCCAGAGCCGGGCGTCGGT
TGCGCCGGCCGTGGGGTAATCACC---GCGATCAACTTCCTCGAAGAGGAAGGGGCCTAT---GACGAAGATCTC---
GAC---------TTCGTTTTCTATGATGTACTGGGCGACGTGGTATGCGGTGGTTTCGCGATGCCGATTCGTGAAAAC---
AAGGCGCAGGAGATCTACATCGTCTGTTC-

>EF174686.1_Uncultured_marine_2

TCTACCCGGCTTATTCTTCACTCTAAGGCCCAGAACACCATTATGGAA------ATG---
GCTGCAGAAGCCGGTACGGTGGAAGATCTTGAA---
TTGGAAGATGTATTGAAAACAGGTTATGGCGATATAAGGTGTGCTGAATCCGGTGGTCCTGAGCCTGGCGTTGGT
TGTGCTGGTCGCGGTGTTATAACC---GCTATCAACTTCCTCGAAGAGGAGGGTGCCTAC---GAAGACGATATA---

GAC---------TTTGTTTTCTACGATGTGTTAGGCGACGTAGTATGCGGTGGTTTTGCCATGCCCATACGTGAAAAC---
AAGGCGCAGGAAATCTACATCGT-------

>KF846687.1_Uncultured_soil_4

TCCACTCGATTGATCCTACACGCCAAGGCACAGAACACCATCATGGAG------ATG---
GCGGCAGAGGCGGGTTCGGTCGAGGATCTGGAA---
CTGGAAGATGTAATGAAGACCGGTTACGCCAATATCAAGTGTGTTGAGTCTGGTGGCCCTGAGCCCGGTGTCGGC
TGTGCGGGTCGTGGTGTCATCACC---GCCATCAACTTCCTGGAAGAAGAGGGTGCCTAT---GAGGAAGATCTG---
GAC---------TTCGTATTCTATGACGTACTAGGTGACGTGGTCTGCGGTGGTTTCGCAATGCCGATTCGTGAGAAC---
AAGGCGCAGGAGATCTACATCGTCTGCTC-

>DQ481454.1_Uncultured_marine plankton_7

TCCACTCGTTTGATCCTGCACTCCAAGGCTCAGACTACTGTTATGCAC------CTG---
GCTGCTGAAGCTGGCACTGTTGAAGACCTGGAG---
CTGGAAGATGTGCTCTCTGTGGGTTACGGCGACGTTAAGTGCGTAGAGTCTGGTGGTCCTGAGCCAGGCGTTGGT
TGCGCTGGTCGCGGTGTTATCACC---GCTATCAACTTCCTCGAAGAGGAAGGTGCTTAC---GACGAAGACCTC---
GAC---------TTCGTGTTCTACGACGTACTGGGTGACGTTGTGTGCGGTGGCTTCGCTATGCCTATCCGTGAAAAC---
AAGGCTCGGGAGATCTACATCGTTTGCTCT

>HQ455919.1_Uncultured_South China Sea_2

TCCACCCGTCTGATCCTGCACTCGAAAGCCCAGACCACAGTCATGCAC------CTG---
GCGGCGGAAGCCGGCTCTGTCGAAGACCTGGAA---
CTGGAAGACGTCATGGCCGTCGGTTACGGCGATGTGAAATGTGTCGAATCCGGTGGTCCTGAGCCCGGTGTCGGC
TGCGCCGGTCGCGGTGTCATCACC---GCCATCAACTTCCTGGAAGAGGAAGGCACCTAC---GACGAGGACCTG---
GAT---------TTCGTGTTTTACGACGTACTGGGTGACGTTGTCTGTGGCGGCTTCGCCATGCCAATCCGTGAGAAC---
AAGGCCCAGGAAATCTACATCGT-------

>HQ611420.1_Uncultured_marine_2

TCCACTCGTTTGATCCTGCACTCTAAAGCCCAAGAAACCATCATGCAC------CTG---
GCTGCTGAAGCTGGCTCCGTGGAGGACTTGGAA---
CTCGAAGACGTTTTGAAAGCTGGTTACGGCGATATCAAGTGTGTTGAATCTGGTGGTCCTGAGCCTGGTGTTGGCT
GTGCCGGTCGTGGTGTGATCACC---GCTATCAACTTCCTCGAAGAAGAAGGCGCTTAC---GAAGAAGACCTC---
GAC---------TTCGTGTTCTACGACGTGTTGGGTGACGTGGTGTGTGGTGGTTTCGCTATGCCCATCCGCCAGAAC---
AAAGCCCAGGAAATCTACATCGT-------

>EU693392.1_Uncultured_coral_5

TCCACCCGCCTGATCCTGCACTCCAAGGCCCAGGAGACCATCATGCAT------CTG---
GCCGCCGAGTCGGGCTCGGTCGAGGACCTGGAG---
CTTGAAGACGTATTGCGCATTGGCTATGCGGACATCAGGTGCGTCGAGTCCGGTGGTCCCGAGCCAGGTGTCGGC
TGCGCCGGGCGTGGGGTCATCACG---GCCATCAACTTCCTCGAAGAGGAAGGCGCCTAC---GACGACGACCTG---

GAC---------TTCGTCTTCTACGACGTGCTCGGAGACGTGGTGTGCGGCGGCTTCGCCATGCCCATCCGCGAGAAC---AAGGCCCAGGAGATCTACATTGT-------

>AB471122.1_Uncultured_soil_2

TCCACCCGTCTTATGCTCCACGAGAAGGCCCAGAACACGATCATGCAC------CTG---GCCGCAGAGGCCGGCGGCGTCGAGGATCTGGAG---CTGGATCAAGTCCTCAAGGTCGGCTACGGCAACACCAAGTGCGTCGAGTCCNGCGGCCCGGAGCCGGGCGTCGGCTGCGCGGGCCGCGGGGTCATCACG---GCCATTAACTTCCTCGAGGAAGAAGGCGCCTAC---GACAAGGACCTG---AAC---------TTCGTCTTCTACGACGTCCTCGGCGACGTGGTCTGCGGCGGCTTCGCCATGCCCATCCGCGAGAAG---AAGGCCCAGGAAATCTACATCGT-------

>EF133806.1_Uncultured_marine sediment_2

TCCACGCGCCTGATTCTGCACTCTAAGGCCCAGAACACCATTATGGAA------ATG---GCTGCCGAGGCCGGTACCGTGGAAGATCTCGAG---CTGGACGATGTCCTTAAGACGGGCTATAGCGACATCCGATGCGTCGAGTCCGGTGGCCCAGAGCCCGGTGTGGGTTGTGCCGGCCGCGGGGTGATCACC---GCCATCAATTTCTTGGAAGAGGAAGGCGCGTAC---GAAGACGACCTG---GAC---------TTCGTCTTCTACGACGTGCTGGGCGACGTGGTCTGCGGCGGTTTCGCCATGCCGATCCGCGAGAAC---AAGGC------------------------

>DQ142743.1_Uncultured_marine microbial mat_3

------------ATTCTGCATTCCAAAGCGCAGAACACAATCATGGAA------ATG---GCCGCAGAGGCCGGCACCGTGGAAGACCTCGAA---CTCGAAGACGTGCTCAAGGTCGGTTTCGGCGACATCAAGTGCGTGGAATCTGGCGGTCCTGAGCCGGGCGTTGGCTGCGCCGGCCGCGGCGTTATCACG---GCCATCAATTTCCTTGAAGAGGAAGGCGCATAC---ACAGACGATCTC---GAT---------TTCGTTTTCTACGACGTTTTGGGCGACGTGGTCTGCGGCGGCTTCGCGATGCCGATCCGCGAAAAT---AAGGCACAGGAAATCTACATCGTTTGCTC-

>GU192556.1_Uncultured_marine mat_11

TCGACCCGTCTGATCCTGCACGCGAAGGCGCAGGACACCATCATGCAC------ATG---GCCGCCGAGGCCGGTTCGGTCGAAGACCTGGAA---CTCGATGATGTCATGGCTACCGGCTATGCCGGCATCAAGTGCGTCGAATCCGGCGGTCCGGAGCCGGGCGTAGGTTGTGCCGGTCGTGGTGTCATCACC---GCGATCAACTTCCTGGAAGAGGAAGGTGCCTAT---GAGGATGACCTG---GAC---------TTCGTGTTCTATGACGTGCTCGGCGACGTCGTCCGCGGTGGCTTCGCGATGCCGATCCGCGAGAAC---AAGGCCCAGGAGATCTACATCGT-------

>KF872898.1_Uncultured_root_5

TCCACGCGTTTGATCCTGCACGCCAAGGCCCAGAACGCCGTCATGCAG------CTC---GCGGCTGAAGCGGGCAGCGTCGAAGACCTTGAG---CTTGAAGACGTACTCGCCGTCGGCTACGGTGGCGTCAAGTGTGTCGAGTCCGGCGGTCCGGAGCCCGGGGTCGGCTGCGCCGGTCGCGGCGTCATTACC---GCCATCAACTTCCTTGAAGAAGAAGGCGCCTAC---TCCGACGATCTC---

GAC---------TTCGTCTTCTACGACGTGCTCGGTGACGTGGTCTGCGGCGGCTTCGCCATGCCGATCCGCGAGAAC---AAGGCACAGGAAATCTACATCGT-------

>KF872908.1_Uncultured_root_4

TCCACACGCTTGATCCTGCACGCCAAGGCACAGAACGCCGTCATGCAG------CTT---GCCGCCGAAGCCGGTAGCGTCGAAGACCTTGAG---CTGGAAGACGTGTTGGCCGTCGGTTATGGTGGCATCAAGTGCGTCGAGTCCGGCGGTCCGGAACCCGGGGTCGGCTGCGCCGGTCGTGGCGTCATCACC---GCCATCAACTTCCTCGAAGAAGAAGGCGCTTAC---TCCGACGACCTC---GAC---------TTCGTCTTCTACGACGTGCTCGGCGACGTCGTCTGCGGCGGTTTCGCCATGCCGATTCGCGAGAAC---AAGGCGCAGGAAATCTACATCGTCTGCTC-

>HQ586273.1_Zhang_South China Sea_16

TCCACCCTCCTCATTCTGCACTCCAAAGCGCAGAACACCATCATGGAA------ATG---GCGGCTGAAGCTGGCACCGTTGAAGACCTCGAG---CTCGAAGATGTGCTCAAGGTTGGCTACGGCGACATCAAGTGCGTCGAATCCGGCGGCCCAGAGCCAGGCGTCGGTTGCGCCGGCCGCGGCGTCATCACC---GCCATCAACTTCCTTGAAGAAGAAGGCGCATAC---GAAGAAGACCTC---GAC---------TTCGTTTTCTACGACGTCCTCGGCGACGTTGTTTGCGGTGGCTTCGCTAAGCCGATCCGCGAAAAC---AAAGCTCAGGAAATCTACATCGTTTGCTC-

>JN097425.1_Uncultured_South China Sea_2

TCAACTCGTTTGATTCTGCATTCGAAGGCACAAAACTCGGTGATGGAA------CTG---GCTGCTGAAGCAGGTTCCGTGGAAGACTTGGAG---TTGGAAGACGTGCTGTCCGTGGGTTTCGGCGGTGTGAAGTGCGTCGAGTCTGGTGGTCCTGAGCCCGGCGTTGGTTGCGCTGGTCGCGGCGTTATCACC---GCCATCAACTTCCTGGAAGAAGAAGGCGCGTAT---GATGAAGACCTC---GAC---------TTCGTGTTCTACGACGTGCTGGGTGACGTGGTGTGCGGCGGTTTCGCTATGCCGATTCGCGAAAAT---AAAGCGCAGGAAATTTACATCGTTTGTTC-

>AY795616.1_Uncultured_soil_2

TCCACCCGTCTGATTCTGCACGCCAAGGCACAGAACACCATCATGGAG------ATG---GCAGCCGAGGCCGGTACGGTTGAGGATCTGGAG---TTGGAAGATGTGCTCAAGGTTGGCTATGGCGATATCAAGTGCGTCGAGTCCGGTGGCCCGGAGCCCGGCGTCGGGTGCGCCGGCCGTGGTGTGATCACT---GCCATCAACTTCCTGGAAGAGGAGGGCGCCTAT---GAGGAAGACCTC---GAC---------TTCGTCTTCTACGACGTGCTGGGTGACGTGGTGTGTGGCGGCTTCGCA---

>EF199958.1_Thioalkalispira

TCCACCCGTCTGATCCTGCACTCCAAGGCCCAGACCACGGTTATGCAT------CTG---GCCGCCGAGGCCGGTTCGGTCGAAGACCTGGAG---CTGGAAGATGTACTGTGCGTGGGCTACGGCGATGTGAAGTGCGTCGAATCCGGTGGTCCTGAGCCGGGTGTAGGTTGTGCCGGTCGTGGCGTAATCACC---GCAATCAACTTCCTGGAAGAGGAAGGCGCCTAC---GACGAAGACCTG---

GAC---------TTCGTATTCTATGACGTACTGGGAGTCGTAGTGTGGTGGCTTCGCCATGCCGATCCGCGAAAAC---
AAGGCCCAGGAGATCTA-------------

>KF846738.1_Uncultured_soil_4

TCCACGCGCCTGATTCTCCACGCCAAGGCACAGAACACCATCATGCAG------ATG---
GCCGCCGATGCGGGTTCGGTGGAAGACCTGGAA---
CTGGAAGACGTGCTCAAGGTCGGCTACGGCGACATCGCCTGTGTGGAATCCGGTGGTCCGGAGCCGGGTGTCGG
CTGCGCCGGCCGCGGCGTGATCACC---GCCATCAACTTCCTGGAAGAAGAGGGCGCCTAT---GAAGAAGACCTC---
GAC---------TTCGTGTTCTACGACGTGCTCGGCGACGTGGTCTGCGGCGGGTTCGCCATGCCGATTCGCGAGAAC---
AAGGCGCAGGAAATCTACATCGTCTGTTC-

>KF846607.1_Uncultured_soil_10

TCCACCCGCCTGATCCTCCACGCCAAGGCGCAAAACACCATCATGCAG------ATG---
GCGTCTGAAGCCGGCTCGGTGGAAGACCTGGAG---
CTGGAGGACGTGCTCAAGATCGGCTACGGCAACATCGCCTGCGTGGAATCCGGCGGCCCCGAACCGGGTGTGGG
CTGCGCCGGCCGCGGCGTGATTACC---GCCATCAACTTCCTGGAGGAAGAGGGCGCCTAC---GAGGACGATCTC---
GAC---------TTTGTGTTCTACGACGTGCTCGGCGACGTGGTGTGCGGCGGCTTCGCGATGCCGATTCGTGAGAAC---
AAGGCACAGGAAATCTACATCGTATGCTC-

>KF846624.1_Uncultured_soil_6

TCCACCCGTCTGATTCTCCACGCCAAGGCGCAAAACACCATCATGCAG------ATG---
GCATCCGAAGCCGGCTCGGTGGAGGACCTGGAG---
CTGGAGGACGTGCTCAAGGTCGGTTATGGCGACATCGCCTGCGTGGAATCCGGTGGTCCCGAGCCGGGCGTCGGC
TGCGCCGGTCGCGGCGTCATCACC---GCCATCAACTTCCTGGAAGAGGAAGGTGCCTAC---GAGGACGATCTC---
GAC---------TTCGTGTTCTACGACGTGCTCGGCGACGTGGTATGCGGCGGTTTCGCCATGCCGATTCGCGAGAAC---
AAGGCCCAGGAAATCTACATCGTCTGCTC-

>KF847824.1_Uncultured_soil_2

TCCACTCGCCTGATCCTGCACGCCAAAGCGCAGAACTCCATCATGCAG------ATG---
GCCGCGGACGCCGGCTCGGTGGAAGACCTGGAA---
CTGGAAGATGTGTTGAAGGTCGGTTACCGCGACATCAAGTGCGTCGAATCCGGCGGCCCCGAGCCGGGTGTGGG
CTGCGCCGGCCGCGGCGTGATCACT---GCGATCAACTTCCTGGAAGAGGAGGGCGCTTAC---GAGGAAGACCTC---
GAC---------TTCGTATTCTATGACGTACTCGGCGACGTGGTGTGTGGCGGTTTCGCCATGCCTATCCGCGAGAAC---
AAGGCGCAAGAAATCTACATCGTCTGCTC-

>HG422902.1_Uncultured_mire_2

------------ATCCTGCATGCCAAGGCCCAGAACTCCATCATGCAG------ATG---
GCGGCGGATGCCGGCTCGGTGGAAGACCTGGAG---
CTGGAAGACGTGCTGAAGGTCGGCTACCGCGACATCAAGTGTGTTGAATCCGGCGGCCCGGAGCCGGGAGTCGG
CTGCGCCGGTCGCGGTGTGATCACC---GCCATCAACTTCCTGGAAGAGGAAGGCGCTTAC---GAGGAGGACCTC---

GAC---------TTCGTGTTCTACGACGTGCTCGGTGATGTGGTCTGCGGCGGTTTCGCCATGCCCATCCGCGAGAAC---AAGGC------------------------

>KF846599.1_Uncultured_soil_6

TCTACCCGCCTGATCCTGCACGCTAAAGCTCAGAACTCCATCATGCAG------ATG---GCGGCCGATGCCGGTTCCGTAGAAGACCTGGAA---CTGGAGGACGTGCTGAAGGTTGGCTACCGCGACATCAAGTGCGTGGAATCCGGCGGTCCTGAGCCCGGCGTCGGCTGCGCCGGCCGTGGCGTGATTACC---GCGATCAACTTCCTGGAAGAGGAAGGTGCCTAC---GAGGAAGACCTC---GAC---------TTCGTATTCTATGACGTGCTCGGTGACGTGGTGTGTGGCGGCTTCGCCATGCCCATTCGCGAGAAC---AAGGCGCAGGAAATCTACATCGTCTGCTC-

>KF846672.1_Uncultured_soil_10

TCTACCCGCCTGATCCTGCATGCTAAAGCTCAGAACTCCATCATGCAG------ATG---GCGGCTGATGCCGGTTCCGTGGAAGACCTGGAG---CTGGAAGACGTGCTGAAGGTCGGCTATCGCGACATCAAATGCGTCGAATCCGGCGGTCCCGAGCCTGGCGTCGGCTGTGCCGGCCGTGGCGTGATTACC---GCGATCAACTTCCTGGAAGAAGAAGGCGCGTAC---GAGGAAGACCTC---GAC---------TTCGTGTTCTATGACGTGCTCGGCGACGTCGTGTGCGGCGGCTTTGCCATGCCGATTCGTGAGAAC---AAGGCGCAGGAAATCTACATCGTGTGTTC-

>KF846611.1_Uncultured_soil_7

TCTACCCGCCTGATCCTGCACGCAAAAGCGCAGAACTCCATCATGCAG------ATG---GCGGCTGACGCCGGCTCGGTGGAAGACCTGGAG---CTGGAAGACGTGCTGAAGGTCGGCTACCGCGACATCAAGTGCGTGGAATCCGGCGGCCCCGAGCCCGGCGTCGGCTGTGCCGGCCGTGGCGTGATTACC---GCGATCAACTTCTTGGAAGAGGAAGGCGCCTAC---GAGGAAGACCTC---GAC---------TTCGTTTTCTACGACGTGCTCGGCGACGTGGTGTGCGGCGGCTTCGCCATGCCCATCCGCGAGAAC---AAGGCACAGGAAATCTACATCGTGTGTTC-

>KF846597.1_Uncultured_soil_4

TCTACGCGCCTGATCCTGCACGCTAAAGCTCAAAACTCCATCATGCAG------ATG---GCGGCTGATGCCGGCTCGGTGGAAGACCTGGAA---CTGGAAGACGTGCTGAAGGTCGGCTACCGCGACATCAAGTGCGTGGAATCGGGTGGTCCCGAGCCCGGAGTCGGCTGTGCCGGCCGCGGCGTGATCACC---GCGATCAACTTCCTCGAAGAGGAAGGCGCGTAC---GAGGAAGACCTC---GAC---------TTCGTGTTCTACGACGTGCTCGGCGACGTGGTGTGCGGCGGCTTCGCCATGCCCATCCGCGAGAAC---AAGGCGCAGGAAATCTACATCGTCTGCTC-

>KF846610.1_Uncultured_soil_2

TCTACGCGCCTGATCCTGCACGCTAAAGCTCAAAACTCCATCATGCAG------ATG---GCGGCTGATGCCGGCTCGGTGGAAGACCTGGAG---CTGGAAGACGTGCTGAAGGTCGGCTACCGTGACATCAAGTGCGTGGAATCGGGTGGTCCCGAGCCTGGAGTCGGCTGTGCCGGCCGCGGCGTGATCACC---GCGATCAACTTCCTCGAAGAGGAAGGCGCTTAC---GAGGAAGACCTC---

GAC---------TTCGTGTTCTACGACGTGCTTGGTGACGTGGTGTGCGGCGGCTTCGCCATGCCCATCCGCGAGAAC---AAGGCACAGGAAATCTACATCGTGTGTTC-

>JF897144.1_Uncultured_marine mat_34

TCGACCCGTCTGATCCTGCACGCCAAGGCCCAGAACTCCATTATGGAG------ATG---GCGGCCGAAGCCGGCTCGGTCGAAGACCTCGAA---CTGGAAGACGTCCTCAAGATCGGCTACCGCGACATTAAATGCGTCGAGTCCGGCGGTCCTGAGCCGGGCGTCGGCTGCGCCGGTCGTGGCGTCATCACC---GCCATCAACTTCCTGGAAGAGGAAGGCGCCTAC---GAGGCCGATCTG---GAC---------TTCGTCTTTTATGACGTGCTCGGCGACGTGGTCTGCGGTGGCTTCGCCATGCCGATCCGCGAGAAC---AAGGCGCAGGAAATCTACATCGTTTGTTCT

>GU192736.1_Uncultured_marine mat_18

TCAACGCGCCTGATCCTGCACGCCAAGGCACAGAATAGCATCATGGAG------ATG---GCCGCCGAGGCCGGTTCGGTCGAAGACCTCGAA---CTGGAAGACGTCCTCAAGACCGGCTACCGCGACATCAAATGCGTCGAGTCTGGCGGCCCCGAGCCAGGCGTTGGCTGCGCTGGTCGCGGCGTCATCACC---GCTATCAACTTCTTGGAAGAGGAAGGCGCCTAC---GAGGCCGACCTT---GAC---------TTCGTCTTCTACGACGTGCTCGGTGACGTGGTCTGCGGCGGCTTCGCGATGCCGATTCGCGAGAAC---AAGGCGCAGGAGATCTACATCGTCTGCTCT

>AF059648.1_Marichromatium_2

TCGACCCGTCTGATCCTGCACTCGAAGGCCCAGGAGACCATCATGCAG------ATG---GCCGCCGACGCCGGTTCGGTCGAGGATCTGGAA---CTCGAGGACGTGCTCAAGGTTGGCTTCGGCGACATCAAGTGCGTCGAGTCCGGTGGCCCCGAGCCGGGCGTCGGCTGCGCCGGTCGCGGCGTCATCACC---GCGATCAACTTCCTCGAGGAGGAGGGCGCCTAC---GAGGAGGATCTC---GAC---------TTCGTCTTCTATGACGTGCTCGGCGACGTGGTCTGCGGTGGCTTCGCGATGCCGATCCGCGAGAAC---AAGGCGCAGGAGATCTACATCGTCTGCTC-

>CP003154.1_Thiocystis_brackish

TCCACCCGTTTGATCCTGCACGCCAAGGCACAGAATACCATCATGGAG------ATG---GCTGCCGAAGCCGGTTCGGTCGAAGACCTCGAA---CTGGAAGACGTACTCAAGGTCGGCTACCGCGATATCAAGTGCGTCGAGTCCGGCGGCCCCGAGCCGGGTGTCGGCTGTGCCGGTCGCGGCGTCATCACG---GCAATCAACTTCCTCGAAGAGGAAGGCGCCTAT---GAGGACGACCTG---GAC---------TTCGTGTTCTATGACGTGCTCGGCGACGTGGTGTGCGGCGGGTTCGCCATGCCGATCCGCGAGAAC---AAGGCGCAGGAAATCTACATCGTCTGCTC-

>EU622784.1_Thiocapsa

TCCACGCGCTTGATCCTGCACGCCAAGGCACAGAATACCATCATGGAG------ATG---GCCGCCGAAGCCGGTTCGGTGGAAGACCTCGAG---CTGGAAGACGTGATGAAGGTCGGCTACCGCAATATTAAGTGCGTCGAGTCCGGCGGCCCCGAGCCGGGTGTCGGCTGCGCCGGGCGCGGTGTCATCACG---GCGATCAACTTCCTCGAAGAGGAAGGCGCTTAC---GAGGAAGACCTG---

GAT---------TTCGTGTTTTACGACGTGCTCGGCGACGTGGTGTGCGGCGGGTTCGCCATGCCGATCCGCGAGAAC---AAGGCGCAGGAAATCTACATCGTCTGCTC-

>GU192656.1_Uncultured_marine mat_12

TCTACCCGTTTGATCCTGCACGCCAAGGCTCAGAATACCATCATGGAG------ATG---GCGGCCGAAGCGGGTTCGGTCGAGGATCTGGAG---CTGGAGGACGTGCTCAAGGTCGGTTACGGCAATATCAAGCGTGTCGAGTCGGGCGGCCCAGAGCCGGGCGTCGGCTGTGCGGGACGCGGCGTTATCACC---GCCATCAACTTCCTGGAAGAGGAAGGCGCCTAT---GAGGCTGACCTG---GAC---------TTCGTCTTCTACGACGTGCTCGGCGACGTGGTCTGCGGCGGGTTCGCCATGCCGATTCGCGAGAAC---AAGGCCCAGGAGATCTACATCGTCTGCTC-

>GU192731.1_Uncultured_marine mat_6

TCTACCCGTCTGATCCTGCACGCCAAGGCTCAGAATACCATCATGGAG------ATG---GCCGCCGAAGCGGGTTCGGTCGAGGATCTGGAG---CTGGAGGACGTGCTCAAGGTCGGTTACGGCAATATCAAGTGCGTCGAGTCGGGCGGCTCAGAGCCGGGCGTCGGTTGCGCCGGCCGCGGCGTCATCACC---GCCATCAACTTCCTGGAAGAGGAAGGCGCCTAT---GAGGCTGACCTG---GAC---------TTCGTCTTCTACGACGTGCTCGGCGACGTGGTCTGCGGCGGGTTCGCCATGCCGATCCGCGAGAAC---AAGGCCCAGGAGATCTATATCGTCTGCTC-

>EU622783.1_Thiocapsa

TCCACCCGTCTGATCCTGCACGCCAAGGCACAGAATACCATCATGCAG------ATG---GCGGCCGATGCCGGCTCGGTCGAAGACCTCGAG---CTGGAAGACGTGCTCAAGGTCGGTTACGGCAACATCAAGTGCGTCGAGTCGGGTGGCCCGGAGCCGGGCGTGGGTTGCGCCGGCCGCGGTGTCATCACG---GCCATCAACTTCCTCGAAGAGGAAGGCGCCTAT---GAGGACGATCTG---GAC---------TTCGTCTTCTACGACGTGCTCGGCGACGTGGTCTGCGGCGGGTTCGCCATGCCGATTCGCGAAAAC---AAGGCGCAAGAGATCTACATCGTCTGCTC-

>KF800062.1_Thiocapsa

TCGACGCGCCTGATCCTGCACGCCAAGGCACAGAATACCATCATGCAG------ATG---GCCGCTGACGCCGGCTCGGTCGAAGATCTGGAG---CTGGAAGACGTGCTCAAGGTCGGCTACGGCAACATCAAGTGCGTCGAGTCGAGCGGCCCGGAGCCGGGCGTGGGTTGCGCCGGTCGCGGTGTCATCACG---GCCATCAACTTCCTCGAAGAGGAAGGCGCTTAT---GAAGACGATCTG---GAC---------TTCGTCTTCTACGACGTGCTCGGCGACGTGGTCTGCGGCGGGTTCGCCATGCCGATTCGCGAAAAC---AAGGCGCAAGAGATCTACATCGTGTGCTC-

>EU622788.1_Allochromatium

TCGACCCGTCTGATCCTGCACTCCAAGGTTCAGAATACCATCATGGAG------ATG---GCCGCCGAAGCCGGTTCGGTCGAAGACCTCGAA---CTGGAAGATGTGCTCAAGGTCGGCTACGGCAACATCAAGTGCGTCGAGTCCGGCGGTCCTGAGCCGGGTGTGGGTTGCGCCGGTCGCGGCGTCATCACC---GCCATCAACTTCCTGGAAGAAGAAGGCGCTTAC---GAGGCCGATCTG---

GAC---------TTCGTGTTCTACGACGTACTCGGCGACGTGGTCTGCGGCGGATTCGCCATGCCGATCCGCGAGAAC---
AAGGCCCAGGAGATCTACATCGTCTGCTC-

>GU192553.1_Uncultured_marine mat_21

TCCACGCGCCTGATCCTGCATGCCAAGGCACAGAATACCATTATGGAG------ATG---
GCCGCCGAGGCCGGTTCGGTCGAGGACCTGGAA---
CTCGAAGATGTACTGAAGGTCGGCTATGGCGACATTAAGTGCGTTGAGTCCGGTGGTCCTGAGCCTGGTGTCGGC
TGCGCCGGTCGCGGCGTTATCACC---GCCATCAACTTCCTGGAAGAAGAAGGCGCCTAC---GACGCCGACCTC---
GAC---------TTCGTCTTCTACGACGTGCTCGGCGACGTGGTCTGCGGCGGTTTCGCCATGCCCATCCGCGAGAAC---
AAGGCACAAGAGATCTACATCGTCTGCTC-

>GQ441359.1_Uncultured_marine mat_15

TCCACGCGCCTGATCCTGCATGCCAAGGAACAAAATACCATTATGGAG------ATG---
GCCGCCGAGGCCGGTTCGGTCGAGGATCTGGAG---
CTTGAAGACGTACTAAAGGTCGGCTACGGCGACATCAAGTGCGTTGAGTCCGGTGGTCCTGAGCCTGGTGTCGGC
TGCGCCGGCCGCGGCGTCATCCCC---GCCATCAACTTCCTGGAAGAAGAAGGCGCCTAC---GAGGCCGACATC---
GAC---------TTCGTCTTCTACGACGTGCTCGGCGACGTGGTCTGCGGCGGTTTCGCCATGCCCATCCGCGAGAAC---
AAGGCACAAGAGATCTACATCGTCTGCTC-

>GQ441368.1_Uncultured_marine mat_101

TCCACGCGCCTGATCCTGCATGCCAAGGCACAGAATACCATTATGGAG------ATG---
GCCGCGGAGGCCGGTTCGGTCGAGGACCTGGAG---
CTTGAAGACGTACTGAAGGTCGGCTACGGCGACATCAGGTGCGTTGAGTCCGGTGGTCCTGAGCCCGGTGTCGGC
TGCGCCGGCCGCGGCGTCATCACC---GCCATCAACTTCCTCGAAGAAGAAGGCGCCTAC---GAGGCCGACATC---
GAC---------TTCGTCTTCTACGACGTGCTCGGCGACGTGGTCTGCGGCGGTTTCGCCATGCCCATTCGCGAGAAC---
AAGGCGCAGGAGATCTACATCGTCTGCTC-

>GU193051.1_Uncultured_marine mat_2

TCCACCCGTCTGATCCTGCATGCCAAGGCACAGAATACCATCATGGAG------ATG---
GCCGCCGAGGCAGGCTCTGTGGAAGACCTGGAG---
CTTGAGGATGTGCTGAAGGTCGGTTACGGCGACATCAAGTGCGTCGAGTCCGGCGGCCCTGAGCCCGGCGTCGGC
TGCGCTGGTCGCGGCGTCATCACC---GCCATCAACTTCCTGGAAGAAGAAGGCGCCTAC---GAGGCCGACCTC---
GAC---------TTCGTCTTCTACGACGTACTCGGCGACGTGGTCTGCGGCGGCTTCGCCATGCCCATCCGCGAGAAC---
AAGGCGCAGGAGATCTACATCGTCTGCTC-

>GU193042.1_Uncultured_marine mat_13

TCCACGCGCCTGATCCTGCACGCCAAGGCCCAGAATACCATCATGGAG------ATG---
GCTGCTGAGGCGGGCTCGGTGGAAGATCTGGAA---
CTCGAAGACGTGCTCAAGGTCGGCTACGGCGACATCAAGTGCGTCGAGTCCGGCGGCCCGGAGCCGGGTGTCGG
CTGCGCCGGTCGCGGCGTCATCACC---GCCATCAACTTCCTTGAAGAAGAAGGCGCCTAC---GAGGCCGACATC---

GAC---------TTCGTCTTCTACGACGTGCTCGGCGACGTGGTCTGCGGCGGCTTCGCCATGCCCATTCGCGAGAAC---AAGGCGCAGGAGATCTACATCGTCTGCTC-

>GU193072.1_Uncultured_marine mat_5

TCCACGCGCCTGATCCTGCACGCCAAGGCCCAGAATACCATCATGGAG------ATG---GCCGCAGAGGCCGGCTCGGTGGAAGATCTGGAG---CTCGAAGACGTGCTCAAGGTCGGCTACGGCGACATCAAGTGCGTCGAGTCCGGCGGCCCTGAGCCGGGTGTCGGCTGCGCCGGTCGCGGCGTCATCACC---GCCATCAACTTCCTCGAAGAAGAAGGCGCCTAC---GAGGCGGATCTC---GAC---------TTCGTTTTCTACGACGTGCTCGGCGACGTGGTCTGCGGCGGCTTCGCCATGCCCATCCGCGAGAAC---AAGGCACAGGAGATCTACATCGTCTGCTC-

>HM750370.1_Uncultured_rhizosphere_4

TCCACCCGCCTGGTCCTGCATGCCAAGGCCCAGAACACCATCATGCAT------CTG---GCCGCCGATGCCGGCAGCGTCGAGGATCTGGAT---CTCGAGGACGTGCTCAAGGTCGGCTTCGGCGAGATCAAGTGCGTGGAGTCCGGCGGACCCGAGCCCGGTGTGGGCTGTGCCGGCCGTGGCGTCATCACC---GCCATCAATTTCCTGGAAGAAGAAGGCGCCTAC---GAAGAGGACCTG---GAT---------TTCGTATTCTACGACGTGCTGGGTAACGTGGTTTGCGGCGGCTTCGCCATGCCGATTCGCGAGAAC---AAGGCCCAGGAAATCTACATCGT-------

>KF846622.1_Uncultured_soil_2

TCCACTCGTCTTATCCTCCACTCAAAGGCACAAAACACCATCATGGAG------ATG---GCGGCCGAGGCCGGTACGGTAGAGGACCTGGAA---CTGGAGGATGTATTAAAGACCGGCTTCGGCGACATCAAGTGCGTCGAGTCCGGCGGTCCTGAGCCTGGTGTCGGCTGCGCCGGCCGTGGCGTTATCACC---GCCATCAACTTCCTGGAGGAAGAGGGTGCCTAT---GAAGAGGATCTC---GAC---------TTTGTATTCTATGACGTGTTGGGCGACGTGGTGTGCGGTGGCTTCGCCATGCCTATCCGCGAGAAC---AAGGCCCAGGAGATCTACATCGTCTGCTCT

>KF861051.1_Uncultured_soil_9

TCCACCCGTCTGATCCTGCACTCCAAGGCCCAGACCACCGTCATGCAC------CTG---GCTGCCGAGGCCGGCACCGTCGAGGACCTGGAG---CTTGAGGACGTACTGTCCGTCGGCTACGGCGACGTCAAGTGCGTCGAGTCCGGTGGCCCGGAGCCGGGCGTCGGTTGCGCCGGTCGCGGGGTCATCACC---GCCATCAACTTCCTGGAAGAGGAAGGCGCCTAC---GACGAGGATCTG---AAC---------TTCGTGTTCTATGACGTCCTCGGCGACGTGGTTTGCGGTGGCTTCGCCATGCCCATCCGCGAGAAC---AAGGCCCAAGAGATCTACATCGT-------

>KF861067.1_Uncultured_soil_5

TCCACCCGGTTGATCCTGCACTCCAAGGCCCAGACCACCGTCATGCAC------CTG---GCCGCCGAGGCCGGCACCGTGGAGGATCTGGAA---CTGGAGGACGTGCTGGCCGTGGGTTACGGCGACGTCAAATGCGTGGAGTCCGGCGGTCCCGAGCCGGGCGTGGGCTGCGCCGGTCGCGGTGTGATCACC---GCCATCAACTTCCTGGAAGAGGAAGGCGCCTAC---GACGAGGATCTG---

GAC---------TTCGTGTTCTATGACGTGCTGGGCGATGTGGTGTGCGGCGGTTTCGCCATGCCCATCCGCGAGAAC---AAGGCCCAGGAGATCTACATCGT-------

>GU193070.1_Uncultured_marine mat_17

TCCACCCGTTTGATTCTGCATTCCAAGGCTCAGAACACCATCATGGAG------ATG---GCCGCCGAGGCCGGTACCGTTGAAGACCTGGAA---CTTGAAGATGTACTCAAGACCGGTTACGGCGAGATCAAGTGTGTTGAGTCCGGTGGTCCCGAGCCGGGTGTGGGTTGTGCCGGTCGCGGGGTAATCACC---GCCATCAACTTCCTGGAGGAAGAGGGCGCCTAC---GAGGAAGACCTG---GAC---------TTCGTATTCTACGACGTGCTGGGTGACGTGGTGTGTGGTGGTTTCGCCATGCCTATCCGTGAAAAC---AAGGCCCAGGAGATCTATATCGTCTGCTC-

>EF583605.1_Uncultured_soil_2

TCTACCCGTCTGATCCTGCACTCCAAGGCGCAGAACTCCGTGATGGAA------CTG---GCTGCCGAAGCCGGCTCCGTTGAGGATCTCGAA---CTCGAAGACGTGCTGTCCGTCGGTTTTGGCGGCGTGAAGTGCGTTGAATCCGGTGGCCCGGAGCCTGGCGTTGGTTGCGCAGGCCGCGGCGTGATCACC---GCGATCAACTTCCTCGAAGAGGAAGGCGCGTAT---GACGAAGACCTC---GAC---------TTCGTGTTCTACGACGTACTGGGCGACGTGGTGTGCGGCGGTTTTGCGATGCCCATTCGCGAGAAC---AAGGCGCA---------------------

>AY231572.1_Uncultured_root_4

TCTACCCGTCTGATCCTGCACTCCAAGGCGCAGAACTCCGTGATGGAA------CTG---GCTGCTGAAGCTGGCTCCGTGGAGGACCTGGAA---CTGGAAGACGTGCTGTCCGTCGGTTACGGCGGCGTGAAGTGCGTCGAGTCCGGTGGTCCGGAGCCTGGCGTTGGTTGCGCAGGCCGCGGCGTTATCACC---GCCATTAACTTCCTCGAAGAGGAAGGCGCTTAC---GACGAAGACCTC---GAC---------TTCGTGTTCTACGACGTGCTGGGCGACGTGGTGTGCGACGGTTTCGCGATGCCCATCCGCGAAAAC---AAGGCTCAGGAGATCTACATCGTGTGTTC-

>AM746519.1_Uncultured_root_4

---ACCCGTCTGATCCTGCACTCCAAGGCACAGAACTCCGTGATGGAA------CTG---GCTGCTGAAGCTGGCTCTGTTGAGGATCTGGAA---CTGGAAGACGTGCTGTCCGTCGGTTTCGGTGGCCTGAAGTGCGTTGAGTCCGGTGGCCCGGAGCCTGGCGTTGGTTGCGCAGGCCGCGGTGTGATTACC---GCCATTAACTTCCTCGAAGAGGAAGGCGCTTAC---GACGAAGACCTG---GAC---------TTCGTGTTCTACGACGTGCTGGGTGACGTGGTGTGCGGTGGTTTCGCGATGCCCATCCGCGAAAAC---AAGGCGCAAGAAATCTACATCGTTTGC---

>AY231530.1_Uncultured_root_8

TCTACTCGTCTGATCCTGCACTCCAAGGCACAGAACTCTGTGATGGAA------CTG---GCTGCTGAAGCTGGCTCTGTTGAGGATCTGGAA---CTGGAAGACGTGCTGTCCGTCGGTTTCGGTGGCGTGAAGTGCGTTGAGTCCGGTGGCCCGGAGCCTGGCGTTGGTTGCGCAGGCCGTGGCGTTATCACC---GCCATTAACTTCCTGGAAGAAGAAGGCGCTTAC---GACGAAGACCTC---

GAC---------TTCGTGTTCTACGACGTGCTGGGTGACGTGGTGTGCGGTGGTTTCGCGATGCCCATCCGCGAAAAC---AAGGCTCAGGAAATCTACATCGTTTGCTC-

>EU331527.1_Uncultured_soil_4

TCCACCCGCCTGATCCTGCACTCGAAGGCGCAGAACTCCGTGATGGAA------CTG---GCTGCAGAAGCCGGTAGCGACGAGGACCTGGAA---CTCGAAGACGTGTTGTCGGTTGGTTACGGCGGCGTGAAGTGCGTCGAGTCCGGTGGTCCTGAGCCCGGCGTCGGTTGCGCCGGCCGCGGCGTGATCACT---GCCATCAACTTCCTCGAAGAAGGAGGCGCGTAC---GACGACGAACTC---GAC---------TTCGTGTTCTACGACGTGCTGGGCGACGTGGTGTGCGGCGGTTTCGCGATGCCGATCCGCGAGAAC---AAGGCGCAGGAGATTTACATCGTCTGCTC-

>FJ008256.1_Uncultured_soil_2

TCGACTCGCCTGATCCTGCACTCGAAGGCCCAGAATTCCGTGATGGAA------CTG---GCCGCCGAAGCCGGCAGCGTGGAAGACCTCGAA---CTAGAAGACGTGTTGTCGGTTGGTTACGGCGGCGTGAAGTGCGTCGAGTCCGGCGGTCCTGAGCCAGGCGTCGGTTGCGCCGGCCGTGGCGTGATCACC---GCGATCAACTTCCTCGAAGAAGAAGGCGCGTAC---GACGACGAGCTC---GAC---------TTCGTGTTCTACGACGTGCTGGGCGACGTGGTGTGCGGCGGCTTCGCAATGCCGATCCGCGAAAAC---AAGGCGCAGGAAATCTACATCGTTTGCTC-

>AY768664.1_Uncultured_root_4

TCTACTCGACTGATCTTGCACTCCAAGGCACAAAACTCCGTGATGGAA------CTG---GCCGCCGAAGCGGGCTCTGTGGAAGACCTCGAA---CTCGAAGACGTGTTGTCGGTTGGTTACGGCGGCGTGAAGTGCGTTGAGTCCGGTGGTCCTGAGCCCGGCGTTGGTTGTGCTGGCCGCGGTGTGATTACC---GCGATTAACTTCCTCGAAGAGGAAGGCGCATAT---GACGACGAACTC---GAC---------TTCGTGTTCTACGACGTGCTGGGTGACGTGGTGTGCGGTGGATTCGCAATGCCGATTCGCGAAAAC---AAGGCGCAGGAAATCTACATCGTGTGCTC-

>AM746558.1_Uncultured_root_2

---ACCCGTCTGATTCTGCACTCCAAGGCACAGAACTCCGTGATGGAA------CTG---GCCGCCGAAGCGGGCAGCGTGGAAGACCTCGAA---CTCGAAGACGTACTGTCGGTTGGCTACGGCGGCGTGAAGTGCGTTGAGTCCGGTGGCCCTGAGCCCGGCGTTGGTTGCGCGGGCCGCGGCGTGATTACC---GCGATCAACTTCCTCGAAGAGGAAGGTGCGTAT---GACGACGAGCTC---GAC---------TTCGTGTTTTACGACGTGCTGGGCGACGTGGTGTGCGGCGGATTCGCGATGCCGATCCGTGAAAAC---AAGGCGCAGGAAATTTACATCGTGTGC---

>JX268267.1_Uncultured_soil_2

TCTACCCGTCTGATCCTGCACTCCAAGGCACAGAACTCCGTGATGGAA------CTG---GCTGCCGAAGCCGGCAGTGTGGAAGACCTCGAA---CTCGAAGACGTGCTGTCGGTTGGCTACGGTGGCGTGAAGTGCGTTGAGTCCGGTGGCCCTGAGCCCGGCGTTGGTTGCGCCGGCCGCGGTGTGATCACG---GCCATCAACTTCCTCGAAGAGGAAGGCGCGTAC---GACGAAGACCTC---

GAT---------TTCGTGTTCTACGACGTGCTGGGCGACGTGGTGTGCGGCGGATTCGCCATGCCGATTCGCGAGAAC---
AAGGCGCAGGAAATCTACATCGTGTGCTC-

>CP001965.1_Sideroxydans

TCGACCCGTTTGATTCTGCACTCGAAGGCACAAAACTCCGTGATGGAA------CTG---
GCTGCCGAAGCCGGTAGCGTCGAGGATCTGGAA---
CTGGAAGACGTACTGTCCGTCGGTTTTGGCGGCATCAAGTGCGTTGAATCCGGTGGTCCTGAGCCCGGCGTTGGTT
GTGCCGGCCGCGGCGTGATCACC---GCCATCAACTTCCTCGAAGAAGAAGGCGCGTAT---GACGAAGCACTC---
GAC---------TTCGTGTTCTACGACGTGCTGGGTGACGTGGTGTGCGGAGGTTTCGCGATGCCGATCCGCGAAAAC---
AAGGCGCAGGAGATCTACATCGTTTGCTC-

>HE600410.1_Uncultured_moss_2

---ACACGTCTAATCTTGCATTCGAAGGCACAAAACTCCGTGATGGAA------CTG---
GCTGCTGAAGCCGGTAGCGTCGAGGATCTCGAA---
CTTGAGGACGTTCTGTCGGTTGGTTTCGGCGGCATCAAGTGCGTTGAATCCGGTGGACCAGAGCCTGGCGTCGGTT
GTGCGGGTCGTGGTGTGATCACC---GCCATCAACTTCCTCGAAGAAGAAGGCGCGTAT---GACGAAGCACTC---
GAC---------TTCGTGTTTTACGACGTGCTGGGTGACGTGGTGTGCGGCGGTTTCGCGGTGCCGATTCGCGAGAAC---
AAGGC------------------------

>AM746527.1_Uncultured_root_4

---ACCCGCCTCATTCTGCACAGCAAGGCGCAAACCAGCGTCATGCAG------CTG---
GCCGCCGATGCGGGTTCGGTGGAAGACCTCGAA---
CTTGAGGATGTGATGAACATCGGCTACGGCGGCGTCAAGTGCGTCGAGTCCGGTGGTCCGGAGCCCGGGGTTGG
TTGCGCCGGCCGTGGCGTTATCACC---GCCATCAACTTCCTTGAAGAGGAAGGCGCCTAC---GACGACGAACTC---
GAC---------TTCGTGTTCTACGACGTGCTGGGCGACGTGGTTTGCGGCGGCTTCGCCATGCCGATCCGCGAAAAC---
AAGGCGCAGGAAATCTACATCGTCTGC---

>AY231515.1_Uncultured_root_4

TCGACCCGCCTGATCCTGCACAGCAAGGCGCAAACCAGCGTCATGCAG------CTG---
GCCGCCGAGGCCGGTTCGGTGGAGGACCTCGAA---
CTCGAGGACGTCATGAACATCGGCTACGGCGGCGTCAAGTGCGTCGAGTCCGGTGGCCCGGAGCCCGGGGTTGG
CTGTGCCGGCCGCGGCGTCATCACC---GCCATCAACTTCCTTGAGGAAGAAGGTGCCTAC---GACGACGAACTC---
GAC---------TTCGTGTTCTACGACGTGCTGGGCGACGTGGTCTGCGGCGGCTTCGCCATGCCGATCCGCGAGAAC---
AAGGCCCAGGAAATCTACATCGT-------

>EU097078.1_Uncultured_coastal_4

TCGACCCGACTCATCCTCCACTCCAAGGCTCAAACCACCGTGATGCAC------CTG---
GCTGCTGAAGCCGGTTCGGTGGAAGACCTCGAA---
CTCGAAGACGTGCTCTCCGTCGGTTTCGGCGGCACCAAGTGCGTTGAGTCCGGTGGTCCGGAGCCTGGCGTGGGC
TGCGCCGGTCGCGGCGTCATCACC---GCCATCAACTTCCTGGAAGAAGAAGGCGCCTAC---GACGAAGACCTG---

GAC---------TTCGTGTTCTACGACGTGCTCGGCGACGTGGTGTGTGGTGGTTTCGCCATGCCCATCCGCGAGAAC---AAGGCTCAGGAAATCTACATCGTCTGCTC-

>KF846735.1_Uncultured_soil_8

TCCACTCGTTTGATCCTTCATTCCAAGGCTCAGACCACGGTTATGCAT------CTG---GCCGCCGAGGCCGGTTCGGTCGAGGATCTGGAG---CTGGAAGACGTGCTGTCCGTCGGTTACGCCGGCATCAAGTGCGTCGAGTCCGGCGGCCCGGAGCCCGGAGTCGGTTGCGCCGGCCGCGGCGTTATCACC---GCCATCAACTTCCTGGAAGAGGAAGGCGCCTAC---GACGAGGATCTG---GAC---------TTCGTATTCTACGACGTGCTGGGTGACGTAGTGTGCGGCGGATTCGCCATGCCGATCCGCGAGAAC---AAGGCGCAGGAAATCTACATCGTGTGTTC-

>KF846806.1_Uncultured_soil_2

TCGACCCGTCTGATCCTGCACTCCAAGGCCCAGACCACTGTTATGCAT------CTG---GCCGCCGAGGCCGGTTCGGTTGAAGACCTGGAG---CTGGAAGACGTTCTGTCCGTCGGTTATGCCGGCATCAAGTGCGTCGAGTCCGGTGGTCCGGAGCCCGGCGTCGGTTGCGCCGGCCGCGGCGTTATCACC---GCCATCAACTTCCTGGAAGAGGAAGGCGCCTAC---GACGAGGAACTG---GAC---------TTCGTATTCTACGACGTGCTGGGTGACGTGGTGTGCGGCGGATTCGCCATGCCGATCCGCGAGAAC---AAGGCGCAGGAAATCTACATCGT-------

>FJ394999.1_Uncultured_rhizosphere_8

TCCACCCGCCTGATCCTCCACTCCAAGGCGCAGACCACCGTTATGCAC------CTG---GCCGCAGAGGCCGGTACGGTCGAAGACCTGGAA---CTGGAAGACGTGCTCTCCGTCGGTTTCGGCGGCATCAAGTGCGTTGAGTCCGGTGGCCCGGAGCCGGGTGTCGGCTGCGCCGGCCGCGGCGTCATCACC---GCCATCAACTTCCTCGAAGAGGAAGGCGCCTAC---GACGAAGACCTC---GAC---------TTCGTCTTCTACGACGTGCTGGGTGACGTGGTGTGCGGCGGCTTCGCCATGCCGATCCGCGAGAAC---AAGGCCCAGGAAATCTACATCGT-------

>AF216931.1_Unidentified_marine rhizosphere_10

TCCACCCGTCTGATCCTTCACTCCAAGGCTCAGACCACCGTTATGCAC------CTG---GCTGCCGAGGCCGGTACCGTTGAGGACCTCGAG---CTCGAGGACGTGCTCTCCGTCGGTTACGGCGGCATCAAGTGCGTCGAGTCCGGTGGTCCGGAGCCGGGCGTCGGCTGCGCCGGCCGCGGCGTTATCACC---GCCATCAACTTCCTCGAGGAAGAGGGTGCCTAC---GACGAGGACCTG---GAC---------TTCGTATTCTACGACGTGCTGGGTGACGTTGTGTGTGGTGGCTTCGCCATGCCGATCCGCGAGAAC---AAGGCTCAGGAAATCTACATCGT-------

>KF846699.1_Uncultured_soil_6

TCCACCCGCCTGATCCTCCACTCCAAGGCCCAGACCACCGTCATGCAC------CTG---GCCGCCGAGGCCGGTACCGTCGAGGACCTCGAG---CTCGAGGACGTGCTCTCCGTTGGTTACGGCGGCATCAAGTGCGTCGAGTCCGGTGGTCCGGAGCCGGGTGTCGGTTGCGCCGGTCGCGGCGTTATCACC---GCCATCAACTTCCTTGAGGAAGAGGGTGCCTAC---GACGAAGACCTC---

GAC---------TTCGTCTTCTACGACGTGCTGGGTGACGTGGTGTGCGGCGGCTTCGCCATGCCGATCCGCGAGAAC---AAGGCTCAGGAAATCTACATCGTCTGCTC-

>JX154784.1_Uncultured_soil_2

TCCACCCGCCTGATCCTGCACGCCAAGGCCCAGAACACCGTCATGCAC------CTG---GCGGCCGAAGCCGGCTCCGTCGAAGACCTGGGA---CTGGACGACGTCCTGGCCGTGGGCTACGGCGGCATCAAGTGCGTCGAGTCCGGTGGCCCGGAACCCGGCGTCGGCTGTGCCGGCCGTGGCGTGATCACC---GCCATCAACTTCCTTGAAGAAGAAGGCGCCTAC---GACGAAGACCTG---GAC---------TTCGTGTTCTACGACGTGCTCGGCGACGTGGTGTGCGGCGGTTTCGCCATGCCCATCCGCGAGAAC---AAGGCCCAGGAAATCTACATCGTCCGCTC-

>FJ687518.1_Pseudomonas_wastewater_6

TCCACCCGTCTGATCCTTCACAGCAAGGCTCAGAACTCTGTCATGGAA------CTG---GCTGCTGAAGCCGGCTCCGTGGAAGATCTGGAA---CTGGAAGACGTACTCTCCGTCGGCCTCGGCGGCATCAAGTGCGTTGAGTCCGGTGGCCCGGAACCTGGCGTCGGCTGCGCCGGCCGTGGTGTGATCACC---GCCATCAACTTCCTGGAAGAAGAAGGCGCCTAC---GACGAAGACCTC---GAC---------TTCGTTTTCTACGACGTGCTGGGTGACGTGGTGTGTGGCGGCTTCGCCATGCCCATCCGCGAAAAC---AAGGCTCAGGAAATCTACATCGTCTGCTC-

>JX268422.1_Uncultured_soil_8

TCCACCCGTCTGATCCTGCACAGCAAGGCTCAGAACTCCGTGATGGAA------CTG---GCTGCCGAGGCCGGCTCCGTGGAAGACCTGGAA---CTGGAAGACGTGCTCTCCGTCGGCTTCGGCGGCGTGAAGTGCGTGGAATCCGGTGGTCCTGAACCCGGCGTCGGCTGCGCCGGCCGTGGTGTGATTACC---GCCATCAACTTCCTGGAAGAAGAAGGCGCCTAC---GACGAAGACCTC---GAC---------TTCGTTTTCTACGACGTGCTGGGTGACGTGGTGTGCGGCGGCTTCGCCATGCCCATTCGCGAAAAC---AAGGCGCAAGAAATCTACATCGTCTGCTC-

>HQ611501.1_Uncultured_marine_6

TCCACCCGTCTGATTCTGCACTCCAAGGCGCAGACCACGGTGATGCAC------CTG---GCCGCTGAAGCCGGCTCGGTGGAAGATCTCGAA---CTCGAAGACGTGCTGTCTGTCGGCTACGGCGGCGTGAAGTGCGTCGAATCCGGCGGTCCGGAGCCGGGTGTCGGTTGCGCCGGCCGCGGCGTGATCACC---GCCATCAACTTCCTGGAAGAAGAAGGCGCCTAC---GACGAAGACCTC---GAC---------TTCGTGTTCTACGACGTGCTGGGTGACGTGGTGTGCGGTGGCTTCGCCATGCCGACCCGCGAAAAC---AAGGCTCAGGAAATCTACATCGTTTGCTC-

>AY231505.1_Uncultured_root_5

TCCACCCGTCTGATCCTCCACAGCAAGGCCCAGACCACCGTGATGCAC------CTG---GCCGCTGAAGCCGGCTCGGTTGAAGACCTCGAA---CTCGACGACGTCCTGTCGGTCGGCTTCGGTGGCGTGAAGTGCGTCGAGTCCGGTGGTCCGGAACCCGGCGTCGGCTGCGCCGGCCGTGGCGTTATCACC---GCCATCAACTTCCTGGAAGAAGAAGGCGCCTAC---GACGACGAACTC---

GAC---------TTCGTGTTCTACGACGTGCTGGGCGACGTGGTGTGTGGTGGCTTCGCGATGCCGATTCGCGAAAAC---AAGGCCCAGGAAATCTACATCGTCTGCTC-

>AB201046.1_Zoogloea_18

TCCACCCGTCTGATCCTGCACAGCAAGGCCCAGACCACCGTGATGCAC------CTG---GCTGCCGAAGCCGGCTCCGTCGAGGATCTCGAG---CTCGAGGACGTCCTGTCCGTCGGTTTTGGCGGCGTCAAGTGCGTTGAGTCCGGTGGCCCGGAACCCGGCGTCGGCTGCGCTGGCCGCGGCGTCATCACC---GCCATCAACTTCCTTGAAGAAGAAGGCGCCTAC---GACGAAGACCTC---GAC---------TTCGTGTTCTACGACGTGCTGGGCGACGTGGTCTGCGGTGGCTTCGCCATGCCGATCCGCGAGAAC---AAGGCTCAGGAAATCTACATCGTCTGCTC-

>JX154728.1_Uncultured_soil_7

TCAACCCGCCTGATCCTGCATAGCAAGGCTCAGACCACCGTTATGCAC------CTG---GCTGCTGAAGCCGGCTCGGTGGAAGATCTCGAA---CTGGAAGACGTCCTTTCCGTCGGTTTCGGCGGCGTCAAGTGCGTTGAATCCGGTGGTCCGGAACCCGGCGTCGGCTGTGCCGGCCGCGGCGTCATCACC---GCCATCAACTTCCTGGAAGAAGAAGGCGCATAC---GACGAAGACCTC---GAC---------TTCGTGTTCTACGACGTCCTCGGTGACGTGGTCTGCGGCGGTTTCGCCATGCCGATCCGCGAAAAC---AAGGCGCAGGAAATCTACATCGTCTGCTC-

>DQ425449.1_Uncultured_stem_11

---ACCCGCCTGATCCTGCACGCCAAGGCTCAGACCACCGTGATGCAC------CTG---GCTGCTGAAGCCGGCTCCGTGGAAGATCTCGAA---CTGGAAGACGTCCTTTCGGTTGGTTTCGGCGGCATCAAGTGCGTTGAATCCGGTGGTCCGGAACCCGGCGTCGGCTGTGCCGGCCGCGGCGTTATCGCC---GCCATCGACTTCCTGGAAGAAGAAGGCGCCTAC---GAGGACGATCTC---GAT---------TTCGTGTTCTATGACGTGCTCGGCGACGTGGTCTGCGGCGGCTTCGCCATGCCGATCCGCGAAAAC---AAGGCCCAGGAAATCTACATCGT-------

>HQ335718.1_Uncultured_soil_11

TCAACCCGTCTGATCCTGCACAGCAAGTGTCAGACCACTGTGATGCAT------CTG---GCTGCTGAAGCCGGCTCGGTGGAAGATCTGGAA---CTGGAAGACGTCCTTTCCGTCGGCTTCGGCGGCATCATGTGCGTCGAATCCGGTGGCCCGGAACCCGGTGTTGGCTGTGCCGGCCGCGGCGTCATCACC---GCCATCAACTTCCTGGAAGAAGAAGGCGCCTAC---ACCGAAGATCTC---GAC---------TTCGTGTTCTATGACGTGCTGGGTGACGTGGTTTGTGGTGGCTTTGCCATGCCGATCCGCGAAAAC---AAGGCTCAGGAAATCTACAT----------

>HQ335666.1_Uncultured_soil_9

TCCACCCGCCTGATCCTGCACAGCAAGGCTCAGACCACCGTGATGCAC------CTG---GCGGCTGAAGCCGGCTCGGTGGAAGACCTCGAA---CTCGAGGACGTCCTTTCCGTCGGTTTCGGCGGCATCAAGTGCGTCGAATCCGGTGGCCCGGAGCCAGGGGTTGGTTGTGCCGGCCGCGGCGTCATCACC---GCCATCAACTTCCTGGAAGAAGAAGGCGCCTAC---ACCGACGACCTC---

GAC---------TTCGTCTTCTACGACGTGCTGGGTGACGTGGTTTGTGGTGGCTTCGCCATGCCGATCCGCGAAAAC---
AAGGCGCAGGAAATCTACATCGT-------

>JN648840.1_Uncultured_soil_3

TCAACCCGCCTGATCCTGCACAGCAAGGCTCAGACCACCGTGATGCAC------CTG---
GCTGCTGAAGCCGGCTCGGTGGAAGATCTCGAA---
CTGGAAGACGTCCTCTCCGTCGGTTTCGGCGGCATCAAGTGCATCGAATCCGGTGGCCCGGAACCCGGCGTCGGC
TGTGCCGGCCGCGGCGTTATCACC---GCCATCAACTTCCTTGAAGAAGAAGGCGCCTAC---ACCGAAGACCTC---
GAC---------TTCGTGTTCTACGACGTGCTGGGTGACGTGGTTTGTGGTGGCTTCGCCATGCCGATCCGCGAAAAC---
AAGGCTCAGGAAATCTACATCGT-------

>KF151672.1_Uncultured_marine_2

TCAACCCGCCTGATCCTGCATAGCAAGGCTCAGACCACCGTGATGCAC------CTG---
GCTGCCGAAGCTGGCTCGGTGGAAGATCTGGAA---
CTGGAAGACGTCCTCTCCGTCGGTTTCGGCGGCATCAAGTGCGTTGAATCCGGTGGTCCGGAACCCGGCGTCGGCT
GTGCCGGCCGCGGCGTTATCACC---GCCATCAACTTCCTTGAAGATGAAGGCGCTTAC---GACGAAGACCTC---
GAC---------TTCGTGTTCTACGACGTGCTGGGTGAGGTGGTCTGCGGTGGCTTCGCCATGCCAATTCGCGAAAAC---
AAGGCTCGGGAAATCTACATCGTCTGTTC-

>KF541077.1_Uncultured_terrestrial grass_2

TCAACCCGCCTGATCCTGCACAGCAAGGCTCAGACCACCGTGATGCAC------CTG---
GCTGCTGAAGCCGGCTCGGTGGAAGATCTGGAA---
CTGGAAGACGTCCTCTCCGTCGGTTTCGGCGGCATCAAGTGCGTTGAATCCGGTGGTCCGGAACCCGGCGTCGGCT
GTGCCGGCCGCGGCGTTATCACC---GCCATCAACTTCCTTGAAGAAGAAGGCGCTTAC---GACGACGAACTC---
GAT---------TTCGTGTTCTACGACGTGCTGGGCGACGTGGTCTGCGGTGGCTTCGCCATGCCGATTCGCGAAAAC---
AAGGCTCAGGAAATCTACATCGTTTGCTC-

>EF174813.1_Uncultured_marine_2

TCCACCCGTCTGATTCTGCAGCTCGAGGCGCAGAACACCATCATGGAA------ATG---
GCCGCAGAGGCCGGTACCGTCGAAGACCTGGAG---
CTCGAAGACGTACTGAAGACCGGTTACGGCGACATCAAGTGCGTCGAGTCAGGTGGTCCTGAGCCGGGCGTCGGT
TGTGCCGGTCGTGGTGTTATCACC---GGCATCAACTTCCTCGAAGAGGAAGGCGCCTAC---GAAGACGACCTC---
GAT---------TTCGTCTTCTACGATGTCCTCGGCGACGTGGTCTGTGGTGGTTTCGCCATGCCGATCCGTGAAAAC---
AAAGGCCCGGGAGATCTACATCG-T-------

>HM999432.1_Uncultured_marine_25

GTGCTTAAGACCGGTTACGGCGACATCAAGTGCGTCGAGTCGGGTGGCCCTGAGCCGGGCGTCGGTTGTGCCGGC
CGTGGTGTTATCACC---GCGATCAACTTCCTCGAAGAGGAAGGTGCCTAC---GAAGACGATCTC---GAT---------

TTCGTGTTCTACGACGTACTCGGCGACGTGGTCTGCGGCGGCTTCGCTATGCCGATCCGCGAGAAC---
AAGGCCCAGGAGATTTACATCGTT------

>HQ455958.1_Uncultured_South China Sea_4

TCCACCCGTCTCATCCTTCACGCGAAGGCCCAGAACACCATCATGCAT------CTG---
GCCGCAGAAGCCGGGTCGGTGGAAGACCTGGAA---
CTCGAAGACGTGCTGAAGGTCGGTTACGGCGACATCAAATGCGTCGAATCCGGTGGCCCGGAGCCGGGCGTCGG
CTGTGCCGGCCGCGGCGTCATCACC---GCCATCAACTTCCTGGAAGAGGAAGGCGCCTAC---GACGAAGACCTC---
GAT---------TTCGTTTTCTATGACGTTCTCGGCGACGTTGTCTGCGGCGGCTTCGCCATGCCGATCCGCGAGAAC---
AAGGCCCAGGAAATCTACATCGTCTGTTC-

>HM750293.1_Uncultured_rhizoplane_21

TCGACCCGACTGATCCTGCAATCCAAGGCGCAGAACACCATCATGGAG------ATG---
GCCGCCGAGGCGGGCACCGTCGAAGACCTCGAG---
CTCGATGATGTGCTGAAGGCCGGTTACAAGGAGATCAAGTGCGTCGAGTCCGGCGGTCCGGAGCCGGGTGTCGG
CTGCGCCGGCCGTGGCGTGATCACC---GCGATCAACTTCCTCGAGGAAGAGGGCGCCTAT---GAAGAGGACCTG---
GAC---------TTCGTCTTCTACGACGTGCTGGGCGACGTGGTGTGTGGCGGCTTCGCCATGCCGATTCGCGAGAAC---
AAGGCGCAGGAAATCTACATCGT-------

>HM750289.1_Uncultured_rhizoplane_2

TCCACCCGCTTGATGCTGCACGCCAAGGCCCAGAACACCATCATGCAA------CTG---
GCCTCCGAGGCCGGCAGCGTGGAAGACCTGGAG---
CTGGAAGATGTGCTCAAGGTCGGTTACGGCGACGTCAAGTGTGTCGAGTCCGGCGGTCCCGAGCCGGGTGTCGGC
TGCGCGGGCCGTGGCGTGATTACG---TCCATCAACTTCCTGGAAGGGGAAGGCGCCTAC---GACGAGGACCTG---
GAT---------TTCGTCTTTTACGATGTGCTGGGTGACGTGGTCTGCGGCGGTTTCGCCATGCCCATCCGTGAGAAC---
AAGGCCCAGGAAATCTACATCGT-------

>KF846677.1_Uncultured_soil_2

TCCACCCGTCTGATCCTGCACGCCAAGGCTCAGAACACCATTATGCAC------CTG---
GCCGCCGAAGCCGGTTCCGTTGAGGATCTGGAA---
CTGGAAGACGTGCTGAAGGTGGGTTACGGTGGCATCAAGTGCGTCGAATCCGGTGGTCCGGAGCCGGGCGTCGG
CTGTGCCGGCCGTGGCGTGATTACC---GCCATCAACTTCCTGGAAGAGGAAGGCGCTTAC---GAGGAAGACCTC---
GAC---------TTCGTGTTCTACGACGTGCTGGGTGACGTGGTTTGCGGTGGTTTCGCCATGCCCATTCGCGAGAAC---
AAGGCTCAGGAAATCTACATCGT-------

>CP007268.1_Halorhodospira

TCCACGCGTCTGATCCTGCACGCCAAGGCACAGAATACCATCATGCAG------ATG---
GCTGCTGACGCCGGCAGCGTTGAAGATCTGGAA---
CTGGAAGATGTTCTCAAGACCGGCTTCGGCGACGTCAAGTGCGTCGAGTCCGGCGGTCCCGAGCCGGGTGTCGGC
TGCGCTGGTCGCGGTGTGATCACG---GCCATCAACTTCCTGGAAGAGGAAGGTGCCTAC---GAGGACGACTTG---

GAC---------TTCGTGTTCTACGACGTACTGGGTGACGTGGTCTGCGGTGGCTTCGCCATGCCCATCCGCGAGAAC---
AAGGCCCAGGAAATCTACATCGT-------

>KC222033.1_Uncultured_Mediterranean_14

TCAACGCGTCTGATCCTTCACTCCAAGGCCCAGAACACCATTATGGAA------ATG---
GCTGCAGAAGCAGGCACCGTGGAAGATCTGGAA---
CTGGAAGATGTACTGAAAACCGGTTACGGCGAAATCAAGTGTGTCGAGTCCGGTGGTCCCGAGCCAGGCGTGGGT
TGTGCCGGTCGCGGTGTTATCACC---GCCATCAACTTCCTCGAAGAGGAAGGCGCCTAC---GAAGAAGATCTC---
GAC---------TTTGTGTTCTATGACGTCCTCGGCGACGTGGTTTGCGGTGGCTTCGCCATGCCGATCCGCGAGAAC---
AAGGCGCAGGAAATCTACATCGT-------

>FJ686505.1_Uncultured_marine sediment_2

TCAACGCGCCTGATCCTGCACTCCAAGGCGCAGAACACCATCATGGAA------ATG---
GCTGCGGAAGCAGGCACCGTGGAAGATCTGGAA---
CTGGAAGATGTATTGAAAACCGGTTACGGCGAAATCAAGTGCGTCGAATCCGGTGGTCCTGAGCCAGGTGTCGGT
TGTGCAGGCCGCGGCGTTATCACC---GCCATCAACTTCCTAGAAGAAGAGGGCGCCTAC---GAAGAAGACCTG---
GAC---------TTCGTTTTCTACGACGTTCTCGGCGATGTGGTCTGCGGTGGATTCGCCATGCCGATCCGTGAAAAC---
AAGGCGCAGGAAATCTACATCGT-------

>KF846608.1_Uncultured_soil_2

TCCACGCGCCTGATCCTTCACTCCAAGGCCCAGAACACCATTATGGAA------ATG---
GCTGCTGAAGCAGGCACCGTTGAAGATCTGGAA---
CTGGAAGATGTATTGAAGACCGGTTACGGCGAAATCAAGTGTGTCGAATCCGGCGGTCCGGAGCCGGGTGTCGGT
TGCGCCGGCCGCGGCGTTATCACC---GCCATCAACTTCCTGGAAGAGGAAGGCGCCTAC---GACGAGGATCTG---
GAC---------TTCGTTTTTATGACGTGCTCGGCGACGTGGTGTGCGGCGGTTTCGCCATGCCGATTCGCGAAAAC---
AAGGCCCAGGAAATCTATATCGTCTGCTC-

>EF208176.1_Uncultured_soil_19

TCGACCCGTCTGATCCTGCACGCCAAGGCCCAGAACTCTATCATGCAG------ATG---
GCTGCTGAAGCCGGCTCCGTGGAAGATCTGGAA---
CTCGAAGACGTTCTGAAGGTGGGCTACCGCGACATCAAGTGCGTCGAATCGGGTGGCCCCGAGCCGGGCGTCGG
CTGTGCCGGTCGTGGTGTCATCACC---GCCATCAACTTCCTGGAAGAAGAAGGCGCCTAC---GAGGAAGACCTC---
GAC---------TTCGTGTTCTACGACGTGCTGGGTGACGTTGTGTGTGGTGGTTTCGCCATGCCCATCCGCGAGAAC---
AAGGCCCAGGAAATCTACATCGTCTGCTC-

>EF199953.1_Ectothiorhodospira_4

TCCACCCGTCTGATCCTGCACGCCAAGGCCCAGGAAACCATCATGCAG------ATG---
GCCGCCGATGCCGGCACCGTGGAAGACCTGGAA---
CTGGAAGACGTGCTCAAGACCGGTTATGCCGGCATCAAGTGCGTCGAGTCCGGCGGTCCCGAGCCGGGCGTGGG
TTGCGCCGGTCGCGGCGTCATCACC---GCCATCAACTTCCTGGAAGAGGAAGGCGCCTAT---GAAGAAGACCTG---

GAC---------TTCGTCTTCTACGACGTGCTCGGCGACGTGGTCTGCGGCGGTTTCGCCATGCCCATCCGCGAAAAC---AAGGCCCAGGAGATCTACATCGT-------

>HQ611745.1_Uncultured_marine_35

TCGACCCGCCTGATTCTGCACGCCAAGGCCCAGAACACCATCATGGAA------ATG---GCGGCGGAGGCCGGCACCGTGGAAGACCTGGAG---CTGGAGGACGTGCTCAAGGCCGGCTATGGCGGCATCAAGTGCGTCGAGTCCGGCGGCCCCGAGCCGGACGTCGGTTGCGCCGGTCGCGGGGTGATCACC---GCGATCAACTTCCTCGAAGAGGAAGGCGCCTAT---GAGGACGATCTG---GAC---------TTCGTCTTCTACGACGTGCTCGGTGACGTGGTGTGCGGCGGTTTCGCCATGCCCATCCGCGAGAAC---AAGGCCCAGGAAATCTACATCGTCTGCTC-

>AF216875.1_Unidentified_marine rhizosphere_30

TCCACCCGCCTGATGCTGCACTCCAAGGCCCAGAACACCATCATGCAC------CTG---GCCGCTGATGCGGGCAGCGTGGAAGACCTGGAG---CTCGAGGACGTGCTCAAGGTGGGTTACGGCGACGTCAAGTGCGTCGAGTCCGGTGGCCCGGAGCCGGGCGTCGGTTGCGCCGGCCGCGGTGTGATCACC---GCCATCAACTTCCTCGAGGAAGAGGGTGCCTAC---GACGAGGACCTG---GAC---------TTCGTCTTCTATGACGTGCTCGGCGACGTGGTGTGCGGCGGTTTCGCCATGCCCATCCGCGAGAAC---AAGGCCCAGGAAATCTACATCGT-------

>AF216933.1_Unidentified_marine rhizosphere_13

TCCACCCGCCTGATGCTGCACGCCAAGGCCCAGAATACCATCATGCAG------CTG---GCTTCCGATGCCGGCAGCGTGGAAGACCTGGAG---CTCGAGGACGTGCTCAAGGTTGGCTACGGCGACGTCAAGTGCGTCGAGTCCGGCGGTCCTGAGCCGGGCGTTGGCTGCGCCGGCCGCGGCGTCATCACC---TCCATCAACTTCCTCGAAGAGAAGGGTGCCTAC---GACGAAGACCTG---GAC---------TTCGTGTTCTACGACGTGCTGGGCGACGTGGTGTGCGGCGGCTTCGCCATGCCCATTCGCGAGAAC---AAGGCCCAGGAAATCTACATCGT-------

>HM750436.1_Uncultured_rhizoplane_3

TCCACCCGCCTCATGCTCCACGCCAAGGCCCAGAACACCATCATGCAC------CTG---GCGGCGGAAGCCGGCTCGGTGGAAGACCTGGAA---CTGGAAGATGTGCTGAAGATGGGTTACGGCGACGTCAAGTGCGTCGAGTCCGGGGGTCCCGAGCCGGGTGTCGGCTGCGCCGGCCGCGGCGTCATCACC---GCCATCAACTTCCTGGAGGAAGAGGGCGCCTAC---GACGAGGACCTG---GAC---------TTCGTCTTCTACGACGTGCTGGGCGACGTGGTGTGTGGCGGCTTCGCCATGCCGATCCGCGAGAAC---AAGGCCCAGGAAATCTACATCGT-------

>KF846691.1_Uncultured_soil_5

TCCACCCGGCTGATCCTGCACGCCAAGGCCCAGAACTCCATCATGCAG------ATG---GCGGCCGACGCCGGTTCCGTGGAAGACCTGGAA---CTGGAAGACGTTCTTAAAGTGGGTTATCGCGACATCAAGTGCGTCGAGTCCGGCGGCCCCGAACCGGGTGTCGGGTGTGCCGGCCGCGGCGTCATCACC---GCCATCAACTTCCTGGAAGAGGAAGGCGCCTAC---GAGGAAGACCTG---

GAC---------TTCGTGTTCTACGACGTGCTGGGCGACGTGGTGTGCGGCGGATTCGCCATGCCCATCCGCGAGAAC---
AAGGCGCAGGAGATCTACATCGT-------

>GU192763.1_Uncultured_marine mat_8

TCTACCCGCCTGATTCTGCACTCCAAGGCGCAGAACACCATCATGGAG------ATG---
GCGGCCGAGGCTGGGTCAGTCGAAGACCTCGAG---
CTGGAAGATGTGCTCAAGATGGGCTACGGCAACATCAAGTGCGTCGAGTCCGGCGGCCCTGAGCCGGGTGTTGGC
TGTGCCGGCCGTGGCGTTATCACC---GCCATCAACTTCCTGGAAGAGGAAGGTGCCTAC---GAGGACGACCTG---
GAC---------TTCGTCTTCTATGACGTGCTGGGCGACGTGGTCTGCGGCGGATTCGCCATGCCGATCCGCGAGAAC---
AAGGCGCAGGAGATCTACATCGTCTGCTC-

>KF846635.1_Uncultured_soil_4

TCCACCCGACTGATCCTCCACTCCAAGGCCCAGAACACCATCATGGAA------ATG---
GCCGCCGAAGCGGGCACGGTCGAAGACCTGGAG---
CTGGATGATGTATTGAAGACCGGCTTCGGCGACATCAAGTGCGTCGAGTCCGGCGGTCCCGAGCCGGGCGTCGGT
TGCGCCGGCCGCGGCGTAATCACC---GCCATCAACTTCCTGGAAGAGGAAGGCGCCTAC---GAGGAAGACCTC---
GAC---------TTCGTGTTTTACGACGTGCTGGGCGACGTGGTGTGCGGTGGTTTCGCCATGCCGATCCGCGAAAAC---
AAGGCCCAGGAGATCTACATCGTCTGCTC-

>FJ395003.1_Uncultured_rhizosphere_2

TCCACCCGCCTGATGCTGCATGCCAAGGCTCAGAACACCATCATGCAC------CTG---
GCCGCCGAGGCCGGTTCCGTCGAGGACCTGGAG---
CTGGAAGACGTGCTCAAGGTCGGCTACGGCGACGTCAAGTGCGTCGAGTCCGGCGGTCCCGAGCCGGGTGTCGG
CTGCGCCGGTCGCGGCGTCATCACC---GCCATCAACTTCCTGGAAGAGGAAGGCGCCTAC---GACGAGGACCTC---
GAC---------TTCGTATCCTACGACGTGCTCGGCGACGTGGTGTGCGGCGGTTTCGCCATGCCCATCCGCGAGAAC---
AAGGCTCAGGAAATCTACATCGT-------

>HM601483.1_Uncultured_marine_3

TCGACCCGACTGATTCTTCATTCCAAGGCTCAGAACACCATCATGGAA------ATG---
GCCGCCGAGGCCGGTACCGTTGAGGATCTGGAA---
CTGGAAGACGTACTGAAGACCGGTTACGGCGACATCAAGTGCGTCGAGTCCGGTGGTCCGGAGCCGGGCGTCGG
TTGTGCCGGCCGCGGTGTTATCACT---GCCATCAACTTCCTCGAAGAGGAAGGCGCCTAC---GAGGAGGACCTG---
GAC---------TTCGTCTTCTATGACGTTCTGGGTGACGTGGTCTGCGGCGGTTTCGCCATGCCCATCCGCGAGAAC---
AAGGCCCAGGAGATCTACATCGT-------

>AB189453.1_Pseudomonas_compost_14

TCCACCCGCCTGATCCTGCACTCCAAGGCGCAGAACACCATCATGGAA------ATG---
GCCGCCGAGGCCGGCACCGTGGAAGACCTGGAG---
CTCGAGGACGTGCTCAAAACCGGCTACGGCGACATCAAGTGCGTCGAGTCGGGCGGTCCGGAGCCGGGTGTGGG
CTGCGCCGGTCGCGGCGTGATCACC---GCGATCAACTTCCTCGAAGAGGAAGGCGCCTAC---GAGGATAACCTG---

GAC---------TTCGTCTTCTACGACGTGCTCGGCGACGTGGTCTGTGGCGGCTTCGCCATGCCCATCCGCGAGAAC---AAGGCCCAGGAGATCTACGTAGTCTGCTC-

>FR669144.1_Pseudomonas_root_2

TCCACCCGCTTGATCCTGCACTCCAAGGCGCAGAACACCATCATGGAA------ATG---GCCGCCGAGGCCGGTACCGTGGAAGACCTGGAA---CTTGAAGACGTGCTCAAGACCGGCTACGGCGACATCAAGTGCGTCGAGTCGGGCGGCCCTGAGCCGGGCGTGGGCTGTGCCGGTCGCGGCGTAATCACC---GCGATCAACTTCCTCGAAGAGGAAGGCGCCTAC---GAGGATGACCTG---GAT---------TTCGTCTTCTACGACGTGCTCGGCGACGTGGTCTGTGGCGGTTTCGCCATGCCCATCCGCGAGAAC---AAGGCCCAGGAAATCTATGTCGTCTGCTC-

>DQ176982.1_Uncultured_marine root_6

TCCACCCGTCTGATCCTGCACTCCAAGGCGCAGAACACCATCATGGAG------ATG---GCTGCCGAGGCGGGCACCGTCGAGGACCTGGAA---CTGGAAGATGTGCTGAAAACCGGTTACGGCGATATCAAGTGCGTCGAGTCCGGCGGCCCTGAGCCCGGTGTCGGTTGCGCCGGCCGCGGCGTTATCACC---GCCATCAACTTCCTGGAGGAAGAGGGCGCCTAT---GAAGACGACCTC---GAT---------TTCGTCTTCTACGACGTACTGGGCGACGTGGTGTGCGGCGGTTTCGCCATGCCCATCCGCGAGAAC---AAGGCGCAGGAGATCTACATCGTCTGCTC-

>KF515759.1_Uncultured_marine_3

TCCACCCGTCTGATCCTCCACTCCAAGGCGCAGAACACCATCATGGAA------ATG---GCTGCCGAGGCCGGTACCGTGGAAGACCTGGAG---CTGGAAGACGTTCTGAAAGTCGGTTATGGCGACATCAAGTGCGTCGAGTCCGGCGGTCCCGAGCCGGGTGTCGGTTGTGCCGGCCGCGGCGTCATCACC---GCCATCAACTTCCTGGAAGAGGAAGGTGCCTAC---GACGAAGATCTC---GAC---------TTCGTTTTCTACGACGTGCTGGGCGACGTGGTCTGCGGTGGCTCTGCCATGCCGATCCGCGAGAAC---AAGGCCCAGGAAATCTACATCGT-------

>AY098506.1_Uncultured_marine rhizosphere_2

TCCACCCGCCTGATTCTGCACTCCAAAGCGCAGAACACCATCATGGAG------ATG---GCGGCTGAGGCTGGCACCGTGGAAGATCTGGAA---CTGGAAGATGTGCTGAAAACCGGCTACGGCGACATCAAGTGCGTCGAGTCTGGTGGTCCTGAGCCTGGCGTCGGCTGTGCCGGCCGCGGTGTTATCACC---GCCATCAACTTCCTGGAAGAGGAAGGCGCCTAT---GAAGAAGACCTC---GAC---------TTCGTCTTCTACGACGTGCTCGGCGATGTTGTTTGCGGCGGGTT---

>KF846702.1_Uncultured_soil_30

TCCACCCGTCTGATTCTGCACTCCAAGGCGCAGAACACCATCATGGAG------ATG---GCCGCTGAGGCAGGCACCGTTGAAGATCTGGAG---CTGGAAGATGTGCTGAAAGTCGGCTACGGCGACATCAAGTGCGTCGAGTCCGGCGGTCCCGAGCCGGGCGTCGGCTGCGCCGGTCGCGGTGTTATCACC---GCCATCAACTTCCTGGAAGAGGAAGGCGCCTAC---GAGGAAGATCTC---

GAC---------TTCGTATTCTATGACGTACTGGGCGACGTGGTGTGTGGTGGTTTCGCCATGCCTATCCGCGAGAAC---AAGGCCCAGGAAATCTACATCGTCTGCTC-

>AF414618.1_Uncultured_marine seagrass_8

TCCACCCGCCTGATTCTGCACTCCAAGGCGCAGAACACCATCATGGAA------ATG---GCCGCTGAGGCCGGTACCGTGGAAGACCTGGAA---CTGGAAGACGTTCTGAAGACCGGCTACGGCGACATCAAGTGCGTCGAGTCCGGCGGTCCCGAGCCGGGTGTCGGCTGTGCAGGCCGCGGCGTTATCACC---GCCATCAACTTCCTGGAAGAGGAAGGCGCCTAC---GAGGAAGACCTC---GAC---------TTCGTATTCTACGACGTACTGGGCGACGTGGTGTGCGGCGGCTTCGCCATGCCCATCCGCGAGAAC---AAGGCCCAGGAAATCTACATCGT-------

>AF216896.1_Unidentified_marine rhizosphere_16

TCCACCCGTCTGATTCTGCACTCCAAGGCGCAGAACACCATCATGGAG------ATG---GCTGCCGAGGCTGGCACCGTGGAAGACCTGGAG---CTGGAAGATGTACTGAAAACCGGCTACGGCGACATCAAGTGCGTCGAGTCCGGCGGTCCCGAGCCGGGTGTCGGCTGTGCCGGCCGCGGTGTTATCACT---GCCATCAACTTCCTGGAAGAGGAAGGCGCCTAC---GAGGAAGACCTC---GAC---------TTCGTCTTCTACGACGTGCTCGGCGACGTGGTGTGCGGCGGATTCGCCATGCCCATCCGTGAGAAC---AAGGCCCAGGAAATCTACATCGT-------

>AF216912.1_Unidentified_marine rhizosphere_2

TCCACCCGTCTGATTCTGCACTCCAAGGCGCAGAACACCATCATGGAA------ATG---GCTGCCGAAGCCGGTACCGTCGAAGACCTGGAA---CTGGAAGATGTGCTGAAAACCGGTTACGGCGACATCAAGTGTGTCGAGTCCGGTGGTCCCGAGCCGGGTGTCGGCTGTGCCGGCCGCGGTGTTATCACC---GCCATCAACTTCCTGGAAGAGGAAGGCGCCTAT---GAGGAAGACCTC---GAC---------TTCGTCTTCTACGATGTGCTCGGCGACGTGGTGTGCGGCGGTTTCGCCATGCCCATCCGCGAGAAC---AAAGCCCAGGAAATCTACATCGT-------

>GU193482.1_Uncultured_marine mat_7

TCCACCCGTCTGATTCTGCACTCCAAAGCTCAAAATACGGTCATGCAC------TTA---GCCGCAGAAGCCGGCACGGTGGAAGACTTGGAA---CTCGAAGATGTAATGCAAGTTGGCTACGGCGATGTGCGTTGCGTTGAATCTGGTGGCCCCGAACCTGGTGTAGGCTGTGCGGGTCGTGGTGTAATCACC---GCCATTAACTTCTTAGAAGAAGAAGGTGCTTAC---GACGAAGAATTA---GAC---------TTTGTGTTTTATGATGTGTTGGGCGACGTAGTTTGTGGTGGTTTCGCCATGCCCATCCGCGAAAAC---AAAGCACAAGAAATTTACATCGT-------

>JF897137.1_Uncultured_marine mat_44

TCCACCCGTCTGATTCTGCACTCCAAAGCGCAAAATACGGTCATGCAC------TTA---GCCGCAGAAGCCGGCACGGTGGAAGACTTGGAA---CTCGAAGATGTAATGCAAGTTGGCTACGGCGATGTGCGTTGCGTTGAATCTGGTGGCCCCGAACCCGGTGTAGGCTGTGCGGGTCGTGGCGTAATCACC---GCCATTAACTTCTTAGAAGAAGAAGGTGCTTAC---GACGACGAATTA---

GAC---------TTTGTGTTTTATGATGTGTTGGGCGACGTGGTTTGTGGTGGTTTCGCCATGCCGATCCGCGAGAAC---AAGGCGCAGGAAATCTACATCGTTTGCTCT

>HF565544.1_Uncultured_marine plankton_4

TCCACTCGTCTGATCCTGCACGCAAAAGCCCAAAACTCCATCATGCAG------ATG---GCGGCTGAAGCCGGTTCGGTCGAAGACCTCGAA---CTCGAAGATGTACTCAAAGTTGGCTACCGCGACATTAAATGCGTTGAATCCGGTGGTCCAGAGCCAGGCGTTGGCTGTGCAGGCCGTGGGGTAATTACC---GCCATCAACTTCCTCGAAGAAGAAGGCGCGTAT---GAAGAAGACCTC---GAC---------TGCGTATTCTATGACGTACTCGGTGACGTTGTGTGCGGTGGTTTCGCGATGCCTATCCGTGAAAAC---AAAGCGCAAGAAATCTACATCGTTTGCTC-

>HF565547.1_Uncultured_marine plankton_2

TCCACCCGTCTGATCCTGCACGCTAAAGCCCAGAACCCCATCATGGAA------ATG---GCCGCCAATGCAGGCAGCGTGGAAGACCTCGAA---CTCGAAGACGTACTCAAAGTCGGTTACCGCGACATCGAATGCGTTGAATCCGGCGGTCCAGAGCCAGGTGTTGGCTGTGCAGGCCGTGGGGTTATTACC---GCCATCAACTTCCTCGAAGAAGAAGGCGCGTAT---GAAGAAGACCTC---GAC---------TTCGTATTCTACGACGTACTCGGTGACGTTGTGTGCGGTGGCTTCGCGATGCCTATCCGTGAAAAC---AAAGCGCAAGAAATCTACATCGCTTGCTCT

>HF565541.1_Uncultured_marine plankton_8

TCCACACGCTTGATCCTGCACGCTAAAGCCCAAAACTCCATCATGGAA------ATG---GCAGCAAACGCAGGCAGCGTGGAAGACCTCGAA---CTTGAAGATGTCATGAAAGTTGGCTACCGCGACATTAAATGCGTCGAATCCGGCGGTCCAGAGCCAGGTGTTGGCTGTGCAGGCCGTGGGGAATCACC---GCCATCAACTTCCTCGAAGAAGAAGGCGCGTAT---GAAGAAGACCTC---GAC---------TTCGTATTCTACGACGTACTCGGTGACGTGGTGTGCGGCGGTTTCGCGATGCCTATCCGCGAAAAC---AAAGCTCAAGAAATCTACATCGTTTGCTC-

>HF565531.1_Uncultured_marine plankton_11

TCCACACGCTTGATCCTGCACGCTAAAGCTCAAAACTCCATCATGGAA------ATG---GCTGCTAATGCAGGCAGCGTGGAAGACCTCGAA---CTCGAAGACGTACTCAAAGTCGGCTACCGCGACATTAAATGCGTTGAATCCGGCGGCCCAGAGCCGGGTGTTGGCTGTGCAGGCCGTGGGGTAATAACC---GCCATCAACTTCTTGGAAGAAGAAGGCGCGTAT---GAAGAAGACCTC---GAC---------TTCGTATTCTATGACGTACTCGGTGACGTTGTGTGCGGTGGTTTCGCAATGCCTATCCGCGAAAAC---AAAGCGCAAGAAATCTACATCGTTTGCTC-

>HF954374.1_Methylomonas

------------ATCCTGCACTCAAAAGCGCAAACCACCATCATGCAT------TTG---GCTGCCGAAGCTGGTAGCGTCGAAGACTTGGAA---CTGGAAGACGTATTGAAAGTCGGCTACGGCGACGTGAAATGCGTTGAATCAGGCGGTCCAGAGCCAGGAGTTGGTTGCGCTGGCCGTGGTGTTATCACC---GCGATCAACTTCCTGGAAGAGGAAGGCGCATAC---GACGACGAACTC---

GAC---------TTCGTATTCTACGACGTATTGGGTGACGTTGTTTGCGGC---

>AF484672.1_Methylomonas

TCAACTCGACTGATCCTGCATGCAAAAGCGCAAAACTCCATCATGCAA------ATG---
GCGGCTGATGCCGGTAGCGTGGAGGATCTGGAA---
TTGGAAGATGTATTGAAAGTCGGTTACGGCGACGTTAAATGCGTTGAGTCCGGCGGTCCTGAGCCTGGAGTGGGT
TGCGCTGGTCGCGGTGTAATCACG---GCGATCAACTTTTTGGAAGAGGAAGGCGCCTAC---GACGACGATCTG---
GAC---------TTCGTGTTCTACGACGTATTGGGCGACGTTGTGTGCGGTGGTTTCGCGATGCCGATTCGTGAAAAC---
AAGGCGCAGGAAATTTACATCGTTTGCTC-

>HE801219.1_Methylomonas

TCCACTCGTTTGATCTTACATGCTAAAGCACAAAACTCTATCATGCAA------ATG---
GCGGCAGACGCCGGCAGCGTGGAAGATTTGGAA---
CTGGAAGATGTATTGAAAGTTGGATACGGCGATGTGAAATGCGTTGAATCCGGCGGTCCAGAGCCAGGAGTTGGT
TGCGCAGGCCGTGGTGTTATCACC---GCTATCAACTTCCTGGAAGAGGAAGGCGCTTAC---ACCGATGACCTG---
GAC---------TTCGTGTTTTATGATGTATTGGGTGACGTTGTATGCGGTGGTTTCGCGATGCCTATCCGCGAAAAC---
AAAGCTCAAGAAATTTACATCGTTTGCTC-

>HF954362.1_Methylomonas

------------ATTCTGCATGCAAAAGCGCAAAACTCCATTATGCAA------ATG---
GCAGCTGACGCCGGTAGCGTTGAAGATTTGGAA---
CTGGAAGATGTATTGAAAGTCGGCTATCGCGACATCAAATGCGTTGAATCCGGTGGTCCGGAGCCAGGTGTAGGC
TGTGCGGGTCGTGGTGTTATCACC---GCCATCAACTTTTTGGAAGAAGAAGGCGCTTAC---GACGAAGACTTA---
GAT---------TTCGTATTCTACGATGTACTGGGTGACGTTGTTTGCGGC---

>AF484673.1_Methylomonas

TCCACACGTTTAATTCTACACGCAAAAGCGCAAAACTCCATCATGCAA------ATG---
GCGGCCGATGCAGGTAGCGTTGAAGATTTGGAA---
TTGGAAGACGTATTGAAAGTGGGTTACCGCGACATCAAATGCGTTGAGTCCGGCGGTCCAGAGCCAGGGGTTGGT
TGTGCAGGCCGTGGTGTTATCACT---GCCATCAACTTCCTGGAAGAAGAAGGCGCTTAC---GACGAAAACCTG---
GAT---------TTCGTATTCTACGATGTATTGGGTGACGTTGTATGCGGCGGTTTCGCGATGCCTATCCGCGAAAAC---
AAAGCGCAAGAAATTTACATCGTTTGCTC-

>CP002738.1_Methylomonas_marine

TCCACACGTTTAATTCTACACGCAAAAGCGCAAAACTCCATCATGCAA------ATG---
GCGGCCGATGCAGGTAGCGTTGAAGATTTGGAA---
TTGGAAGACGTATTGAAAGTGGGTTACCGCGACATTAAATGCGTTGAGTCCGGCGGCCCAGAGCCAGGCGTTGGT
TGTGCCGGCCGCGGTGTTATCACT---GCCATCAACTTCCTGGAAGAAGAAGGCGCTTAC---GACGAAAACCTG---

GAC---------TTCGTGTTCTACGACGTATTGGGTGACGTTGTGTGCGGCGGTTTCGCGATGCCGATTCGCGAAAAC---AAAGCGCAAGAAATTTATATCGTTTGCTC-

>HM210360.1_Uncultured_marine_4

TCAACACGATTGATTCTACATTCAAAAGCTCAAACAACAGTTATGCAT------CTG---GCCGCCGAGGCTGGCACAGTGGAAGATCTTGAG---TTGGAAGATGTATTGTCAGTCGGCTACGGTGACATTAAGTGTGTTGAATCGGGTGGTCCTGAGCCAGGTGTTGGTTGCGCGGGTCGTGGCGTAATCACT---GCTATTAACTTTCTTGAAGAAGAAGGTGCTTAT---GACGAAGATTTA---GAT---------TTTGTGTTCTATGACGTACTAGGTGACGTTGTATGTGGCGGGTTTGCAATGCCTATCCGTGAGAAC---AAGGCACAGGAAATCTACATCGTT------

>EF174766.1_Uncultured_marine_5

TCTACCCGTTTGATCCTTCACTCAAAGGCGCAAAATACCATCATGGAA------GCC---GCAGCAGAGCAAGGCTCGGTAGAAGATATTGAA---TTAGAAGATGTACTAAAGATTGGCTACGGCAATGTGAAATGTGTCGAGTCAGGTGGCCCTGAGCCGGGCGTTGGGTGTGCTGGTCGTGGTGTTATCACC---GCTATCAACTTCTTAGAAGAAGAAGGCGCATAC---GAAGATGATCTC---GAT---------TTTGTCTTCTATGACGTACTGGGTGATGTGGTATGTGGCGGGTTCGCCATGCCTATTCGTGAAAAC---AAAGCGCAAGAGATCTATATCGTT------

>DQ481439.1_Uncultured_marine plankton_8

TCAACTCGTTTGATCCTTCACTCTAAAGCTCAAAACACAATCATGCAG------ATG---GCAGCTGATGCCGGTTCTGTAGAAGATCTGGAA---CTTGAAGATGTACTGAAAGTCGGTTACGGCGATGTCAAATGTACAGAATCAGGGGGTCCAGAGCCAGGGGTTGGTTGTGCCGGTCGTGGTGTTATCACG---GCGATTAACTTCCTTGAGGAAGAAGGCGTTTAC---GAAGAAGATCTT---GAT---------TTCGTATTCTATGACGTACTTGGTGACGTTGTATGTGGCGGTTTCGCGATGCCAATTCGTGAAAAC---AAAGCCCAGGAAATCTATATCGTATGTTC-

>EU916280.1_Uncultured_Baltic_11

TCAACACGTTTGATCCTTCACGCCAAAGCTCAGAACACAATCATGCAG------ATG---GCTGCTGATGCAGGTTCTGTTGAAGATTTGGAA---TTGGAAGATGTACTTAAGATCGGTTATGGCGATATTAAATGTACAAAATCTGGTGGTCCAGAACCAGGGGTAGGTTGTGCAGGTCGTGGTGTTATCACG---GCGATTAACTTCCTTGAAGAAGAAGGGGCTTAT---ACTGATGTCCTC---GAC---------TTCGTTTTCTATGACGTTCTTGGTGACGTTGTGTGTGGTGGTTTCGCGATGCCAATTCGTGAAAAC---AAAGCACAAGAAATTCACATCGTATGTTC-

>FJ502301.1_Uncultured_meromictic lake_2

TCTACCCGTTTAATTCTGCACGCAAAAGCACAAACCACCATCATGAGC------TTA---GCTGCTGAAAGCGGCAGTGTTGAAGATTTAGAG---CTTGAAGATGTCTTGAAAGTGGGTTACGCCGGCATCAAATGCGTTGAATCAGGTGGTCCAGAACCAGGTGTAGGCTGTGCGGGTCGTGGGGTTATTACT---GCGATCAACTTTTTGGAAGAAGAAGGGGCTTAC---GACGATGAACTT---

GAT---------TTCGTTTTTATGATGTACTTGGGGATGTTGTGTGTGGTGGTTTTGCCATGCCAATCCGTGAGAAC---AAAGCGCAAGAAGTTTACNTCGTTTGCTC-

>AY937260.1_Methylobacter

TCTACCCGTTTAATTCTTCATGCAAAAGCTCAAACTACTATTATGAGC------TTG---GCTGCTGAAGCGGGTAGTGTTGAAGATTTAGAG---CTTGAAGATGTACTGAAAGCGGGTTACCGCGGTATTAAATGCGTTGAATCAGGTGGTCCTGAGCCTGGTGTCGGTTGTGCGGGTCGCGGCGTTATCACT---GCGATTAACTTCCTTGAAGAAGAAGGTGCTTAT---GATGATGAACTT---GAC---------TTCGTTTTTATGATGTACTTGGGGATGTTGTGTGTGGTGGCTTTGCCATGCCAATCCGTGAAAAC---AAGGCGCAAGAAATTTACATCGTTTGCTC-

>AB524080.1_Methylovulum

-----------------------AAAGCACAAACCACCATCATGAGC------TTG---GCTGCGGAAGCAGGCAGCGTGGAAGATCTGGAA---TTGGAAGACGTGTTGAAAGTCGGTTACGGCGGCATCAAATGCGTTGAATCAGGCGGCCCTGAGCCAGGCGTCGGCTGTGCAGGTCGTGGTGTTATCACC---GCGATTAACTTCCTCGAAGAAGAAGGGGCTTAT---GATGATGAACTT---GAC---------TTCGTTTTTATGATGTACTTGGGGATGTTGTGTGTGGTGGTTTTGCCATGCCAATCCGTGAGAAC---AAGGCGCAAGAAATTTACATCGTTTGCTC-

>AF484677.1_Methylobacter_5

TCTACTCGTNTGATCCTGCACTCAAAAGCACAAACGACCATCATGAGC------CTG---GCGGCTGAAGCCGGCAGCGTTGAAGATCTTGAA---CTGGAAGATGTGTTGAAAGTCGGCTTCGGCGGCGTTAAATGCGTTGAATCTGGCGGCCCTGAGCCCGGCGTCGGCTGTGCCGGCCGCGGTGTAATCACG---GCCATCAACTTCCTCGAGGAAGAAGGAGCTTAC---GATGATGAACTC---GAT---------TTCGTGTTCTATGATGTTCTTGGGGATGTTGTGTGTGGYGGCTTTGCCATACCAATCCGTGAAAAC---AAGGCGCAGGAAATTTACATCGTTTGCTC-

>AF484675.1_Methylobacter

TCTACTCGYCTGATGCTGCAYGCAAAAGCGCAAACRACCATCATGAGT------CTG---GCGGCTGAAGCCGGTAGCGTTGAAGATCTGGAA---TTGGAAGATGTRTTGAAAGACGGCTTCGGCGGCGTTAAATGCGTTGAATCAGGCGGCCCTGAGCCNGGCGTCGGCTGTGCYGGCCGCGGTGTAATCACG---GCCATTAACTTCCTSGAAGAAGAAGGAGCCTAT---GATGATGAACTC---GAY---------TTCGTGTTCTAYGAYGTACTNGGGGACGTTGTGTGTGGYGGYTTTGCGATGCCAATCCGTGAAAAC---AAGGCGCAGGAAATTTACATCGTTTGCTC-

>AF484676.1_Methylobacter

TCTACTCGTCTGATGCTGCAYKCAAAAGCGCAAACRACCATCATGAGT------CTG---GTGGCTGAAGCCGGCAGCGTTGAAGATCTTGAA---CTGGAAGATGTATTGAAAGCCGGCTTCGGCGGCGTTAAATGCGTTGAATCAGGCGGCCCTGAGCCNGGTGTCGGCTGTGCYGGCCGCGGTGTAATCACG---GCCATTAACTTCCTSGARGAAGAAGGAGCCTAY---GATGATGAACTC---

GAY---------TTCGTGTTCTAYGAYGTWCTNGGGGAYGTTGTGTGTGGYGGCTTTGCSATGCCAATCCGTGAAAAC---AAGGCGCAGGAAATTTACATCGTTTGCTC-

>AY180983.1_Uncultured_root_3

---GGTACGGTCGATGATCTGGAG---CTGGCTGACGTGCTCAAGGTTGGCTTCGGCGGCGTAAAATGCGTGGAGTCCGGAGGTCCGGAACCAGGCGTCGGCTGTGCCGGCCGCGGCGTGATTACG---GCGATCAACTTCCTCGAAGCCGAGGGTGCATAC---ACGCCCGATCTC---GAT---------TTCGTGTTCTATGACGTCCTTGGTGACGTCGTGTGCGGCGGGTTCGCCATGCCGATCCGGGAGAAC---AAGGCCGAAGAGATCTACATCGTCTGTTCT

>EU693417.1_Uncultured_coral_10

------------ATTCTTCACTCTAAAGCTCAGAACACAATCATGGAG------ATG---GCTGCTGAAGCCGGAACGGTTGAAGATCTTGAG---TTCGAAGATGTATTGAAAGTTGGGTACGGCGATGTTCGCTGTGTCGAATCAGGCGGTCCTGAGCCTGGTGTTGGTTGTGCTGGTCGCGGTGTGATCACA---GCTATTAACTTTCTTGAAGAAGAGGGCGCTTAC---GAGGGTGAATTA---GAT---------TTTGTTTTTTACGACGTTCTTGGTGACGTTGTATGCGGTGGCTTCGCGATGCCAATTCGTGAAAAC---AAAGCGCAAGAAATCTACATCGTT------

>JN638619.1_Uncultured_Black Sea_12

---GAGTCCGGTGGTCCTGAGCCAGGAGTTGGTTGTGCTGGTCGTGGTGTTATTACA---GCTATTAACTTTCTTGAAGAAGAGGGTGCTTAC---GATGATGAGCTA---GAT---------TTCGTTTCTTACGATGTTCTTGGTGACGTTGTTTGTGGTGGATTTGCTATGCCAATTCGTGAAGGT---AAAGCACAAGAAATTTACATCGTT------

>KF546415.1_Uncultured_marine_2

TCCACTCGTTTAATGTTGCACTGTAAAGCACAAACCACTGTTTTACAT------TTA---GCTGCAGAAAGAGGAACTGTAGAGGATATCGAG---CTTGATGAAGTAGTAATTCCTGGTTACAACAACGTTTTATGCGTTGAGTCCGGTGGCCCTGAGCCTGGAGTTGGATGTGCTGGTCGTGGTATTATTACT---GCTATCAACTTCCTTGAAGAAGAAGGTGCTTAC---GAAAAC---CTA---GAT---------TTCGTATCTTATGATGTATTAGGAGACGTTGTTTGTGGTGGTTTCGCTATGCCTATCCGTGAAGGA---AAAGCACAAGAAATCTACATCGTT------

>AY896465.1_Uncultured_marine_3

TCCACACGATCTATGTTGCATTGTAAAGCACTAACCACTGTTTTACAT------TTA---GCTGCAGAAAGAGGAACTGTAGAAGATATTGAA---CTTGATGAAGTAGTAATTCCTGGCTATAACAACGTTTTATGCGTTGAGTCCGGTGGTCCTGATCCTGGAGTTGGATGTGCTGGTCGTGGTATTATTACT---GCTATCAACTTCCTTGAAGAAGAAGGTGCTTAC---GAAAAC---CTA---GAT---------TTCGTATCTTATGATGTATTAAGAGACGTTGTTTGTGGTGGTTTCGCTATGCCTATCCGTGAAGGA---AAAGCACAAGAAATCTACCTCGTT------

>DQ481383.1_Uncultured_marine plankton_7

TCCACACGTTTAATGTTGCATTGTAAAGCACAAACCACTGTTTTACAT------TTA---
GCTGCCGAAAGAGGAACTGTAGAAGATATTGAA---
CTTGATGAAGTAGTAATTCCTGGCTATAACAACGTTTTATGCGTTGAGTCCGGTGGTCCTGAGCCTGGAGTTGGAT
GTGCTGGTCGTGGTATTATTACT---GCTATCAACTTCCTTGAAGAAGAAGGTGCTTAC---GAAAAC---CTA---GAT----
-----ATCGTATCTTATGATGTATTAGGAGACGTTGTTTGTGGTGGTTTCGCTATGCCTATCCGTGAAGGA---
AAAGCACAAGAAATTTACATCGTCTGTTCT

>KF546409.1_Uncultured_marine_416

TCCACACGTCTAATGTTGCATTGTAAAGCACAAACCACTGTTTTACAT------TTA---
GCTGCAGAAAGAGGAACTGTAGAAGATATTGAA---
CCTGATGAAGTAGTAATTCCTGGCTATAACAACGTTTTATGCGTTGAGTCCGGTGGTCCTGAGCCTGGAGTTGGAT
GTGCCGGTCGTGGTATTATTACT---GCTATCAACTTCCTTGAAGAAGAAGGTGCTTAC---GAAAAC---CTA---GAT----
-----TTCGTATCTTATGATGTATTAGGAGACGTTGTTTGTGGTGGTTTCGCTATGCCTATCCGTGAAGGA---
AAAGCACAAGAAATCTACATCGTTACCTCT

>EF631898.1_Uncultured_marine_10

TCTACCCGTTTGATTCTGCATTCAAAAGCACGAAACACCATTATGGAA------ATG---
GCGGCCGAAGCGGGCACCGTTGAAGATCTGGAA---
TTGGAAGATGTACTGAAAGTGGGTTATGGCGATGTGCGTTGCGTTGAATCCGGTGGCCCGGAGCCTGGAGTGGG
ATGTGCAGGCCGTGGTGTTATTACC---GCTATTAACTTCTTGGAAGAAGAAGGTGCCTAC---GAAGATGACATC---
GAT---------TTCGTTTTCTATGACGTACTGGGTGACGTTGTGTGCGGCGGATTCGCTATGCCCATTCGTGAAACC---
AAAGCGCAGGAAATCTATATCGT-------

>AY896431.1_Uncultured_marine_4

TCCACTCGACTTATTCTTCATGCTAAGGCACAAAATAACATTATGGAA------ATG---
GCAGCCGAAGCGGGCACTGTAGAAGATCTCGAG---
CTCGAAGATGTGTTGAAAGTTGGTTACTCCGACATTAAATGCGTTGAGTCTGGCGGTCCTGAGCCAGGTGTTGGTT
GTGCCGGCCGCGGTGTTATCACC---GCGATCAACTTCCTTGAAGAAGAGGGTGCCTAC---GAAGAAGACCTG---
GAT---------TTCGTTTTCTACGACGTACTTGGTGATGTTGTCTGTGGTGGCTTTGCGATGCCGATTCGTGAAAAC---
AAGGCCCAAGAAATCTACATCGT-------

>JN097347.1_Uncultured_South China Sea_4

TCGACTCGTTTGATGCTGCATGCCAAAGCTCAAAACACGATCATGCAA------ATG---
GCTGCCGACGCCGGTAGCGTGGAAGACCTGGAA---
CTGGAAGACGTTCTTAAGATCGGCTACGGCGATACCAAGTGCGTCGAATCAGGCGGTCCTGAACCTGGCGTGGGT
TGTGCAGGCCGCGGCGTGATTACC---GCCATTAACTTCCTGGAAGAAGAAGGCGCATAC---GACGAAGAATTG---
AGC---------TTCGTGTTCTACGACGTTCTGGGGGACGTGGTTTGTGGCGGTTTTGCCATGCCGATTCGCGAAAAC---
AAGGCTCAGGAAATCTATATCGTT------

>GU196880.1_Uncultured_marine dinoflagellate_5

TCCACTCGTCTGATTCTGCATTCAAAAGCACAAACCACAGTGATGCAT------CTG---
GCCGCTGAAGCCGGTTCTGTTGAAGATCTGGAG---
CTGGAAGATGTGATGGCGGTCGGTTATGGCGATGTGAAATGTGTAGAGTCCGGTGGCCCTGAACCTGGTGTAGGT
TGCGCAGGACGCGGGGTAATTACC---GCGATTAACTTCCTGGAAGAGGAAGGTGCCTAC---GACGAAGATCTG---
GAT---------TTTGTGTTCTACGACGTACTGGGTGATGTGGTGTGTGGTGGTTTTGCGATGCCAATTCGTGAAAAC---
AAAGCACAGGAGATCTACATCGT-------

>AF414616.1_Uncultured_marine seagrass_4

TCCACCCGCCTGATCCTGCACGCTAAAGCCCAGACCACCATCATGCAT------CTG---
GCCGCTGAAGCTGGTTCGGTTGAAGATCTGGAA---
CTGGAAGACGTTCTGAAAGCCGGTTTTAAAGATATTAAATGTGTTGAATCTGGCGGTCCTGAGCCGGGGGTTGGTT
GTGCTGGCCGTGGTGTTATCACC---GCCATCAACTTCCTGGAAGAAGAAGGCGCTTAC---GAAGAAGATCTC---
GAC---------TTTGTGTTCTACGACGTTCTAGGCGACGTTGTGTGCGGTGGCTTCGCTATGCCGATCCGTGAAAAC---
AAAGCACAGGAAATCTACATCGT-------

>KC256777.1_Pseudanabaena_shore

TCCACTCGACTCATTCTTCACGCGAAGGCGCAAAACACCATTATGGAA------ATG---
GCAGCTGAAGCCGGTTCGGTTGAAGACTTAGAA---
TTAGAAGATGTATTACGTGTCGGTTATGCCGGTATTCGCTGTGTTGAGTCTGGTGGTCCAGAACCAGGTGTTGGTT
GTGCGGGTCGTGGTGTTATTACG---GCAATTAACTTTTTAGAAGAAGAAGGCGCGTAC---GGAGAAGATTTA---
GAC---------TTCGTATTTTACGACGTCCTCGGTGATGTAGTGTGTGGTGGTTTTGCGATGCCAATTCGCGAAAAC---
AAAGCACAAGAAATCTACATCGT-------

>DQ481444.1_Uncultured_marine plankton_2

TCCACTCGCTTGATCCTTCACGCAAAAGCGCAAAACACCATTATGGAA------ATG---
GCAGCAGAGGCCGGTTCTGTTGAAGACTTGGAA---
TTAGAAGACGTACTTAAAGTGGGTTACGGCGGTGTGCGCTGCGTTGAGTCGGGTGGCCCTGAGCCGGGTGTCGGT
TGTGCCGGTCGTGGTGTGATCACG---GCAATTAACTTCTTAGAAGAAGAAGGCGCGTAT---GAAGAAGACTTA---
GAC---------TTCGTTTTCTACGATGTACTGGGTGACGTTGTATGTGGTGGTTTCGCGATGCCGATCCGCGAGAAC---
AAAGCACAAGAGATCTACATCGTT------

>EF174691.1_Uncultured_marine_2

TCCGCGCGTTTAATCCTTCACGCGAAAGCGCAAAACACCATTATGGAA------ATG---
GCGGCAGAAGCCGGTTCGGTTGAAGACTTAGAA---
CTGGAAGATGTATTAAAAGTCGGTTACGGCGGTGTGCGCTGTGTTGAGTCGGGCGGTCCTGAGCCAGGCGTCGGT
TGTGCCGGTCGTGGTGTGATCACA---GCAATCAACTTCTTGGAAGAAGAAGGCGCGTAC---GAAGAAGATTTA---
GAC---------TTCGTTTTTTATGACGTACTGGGTGACGTTGTGTGTGGTGGTTTCGCGGTGCCTATCCGCGAGAAC---
AAAGCACAAGAAATCTACATCGTT------

>EF174699.1_Uncultured_marine_2

TCCACGCGTTTAATCCTTCACGCGAAAGCGCAAAACACCATTATGGAA------ATG---
GCGGCAGAAGCTGGTTCAGTAGAAGATTTAGAA---
CTGGAAGATGTATTAAAAGTCGGTTACGGCGGTGTGCGCTGTGTTGAGTCGGGCGGTCCTGAGCCAGGCGTGGGT
TGTGCCGGTCGTGGTGTAATCACT---GCAATCAACTTCTTGGAAGAAGAAGGCGCGTAC---GAAGAAGATTTA---
GAC---------TTCGTTTTTTACGACGTACTGGGTGACGTTGTATGTGGTGGTTTCGCGATGCCTATCCGCGAAAAC---
AAAGCACAAGAAATCTACATCGTT------

>HM210377.1_Gamma1_marine_9

TCCACGCGTTCCATCCTGCACTCCAAAGCTCAAACCACTGTGATGCAT------CTG---
GCTGCCGAAGCCGGCACCGTGGAAGATCTGGAG---
CTGGAAGATGTGCTGTCTGTCGGTTACGGCGATGTAAAATGCGTCGAGTCCGGTGGTCCCGAGCCGGGTGTCGGC
TGCGCCGGTCGCGGTGTTATCACC---GCCATCAACTTCCTGGAAGAGGAAGGCGCTTAC---TACGAAGATCTG---
GAC---------TTCGTATTCTACGATGTCCTGGGCGACGTGATCTGCGGTGGCTTTGCTATGCCCATCCGTGAAAAC---
AAAGCGCAAGAAATCTACATCGT-------

>HQ660857.1_Uncultured_marine_6

TCAACACGTCTAATCCTGCATACAAAAATGCAAAATACCATCATGGAG------ATG---
GCAGCTGAAGCGGGCACGGTAGAAGATATCGAA---
TTAGAAGATGTTCTGCTAACGGGTTACGCTGGTATTAAATGTGTTGAATCTGGTGGTCCTGAGCCTGGTGTTGGTT
GCGCTGGTCGCGGTGTAATAACA---GCGATTAACTTCCTTGAAGAGGAAGGTGCATAT---GAAGAAGATTTA---
GAC---------TTCGTATTTTATGACGTACTAGGCGATGTTGTATGTGGTGGTTTTGCGATGCCTATTCGTGAAAAC---
AAAGCACAAGAGATCTACATCGTTTGTTCT

>GQ426265.1_Uncultured_hot spring_2

---ACCCGTCTGATCCTTCACTCTAAAGCACAAGCCAGTGTTATGCAT------CTG---
GCTGCTGAAGCCGGAACTGTTGAGGATATCGAG---
CTCGAAGACGTTCTGTCTATCGGCTACGGTGGTGTTAAGTGTGTTGAATCTGGTGGTCCTGAGCCAGGCGTTGGTT
GCGCTGGTCGCGGTGTTATCACC---GCTATCAACTTCCTCGAAGAGGAAGGGGCATAC---GATGAAGATCTC---
GAC---------TTCGTATTTTACGATGTACTGGGTGACGTTGTCTGCGGCGGCTTCGCGATGCCAATTCGTGAAAAC---
AAGGCGCAGGAGATCTACATCGTTGTCTCT

>AF082989.1_Vibrio_2

------------ATTCTACACTCCAAAGCACAGAATACCATCATGGAG------ATG---
GCGGCTGAAGCGGGGACGGTTGAAGACATCGAA---
CTAGAAGATGTACTTAAAATCGGTTACGGCGATGTTCGTTGTGTTGAGTCAGGCGGTCCAGAGCCAGGTGTTGGCT
GTGCGGGTCGCGGTGTTATCACG---GCCATCAACTTTTTGGAAGAAGAAGGAGCCTAC---GAGGACGACTTG---
GAC---------TTCGTATTCTACGACGTACTGGGTGACGTTGTATGTGGTGGTTTTGCGATGCCAATTCGTGAAAAC---
AAAGCAGAAGAAATCTACATCGTT------

>EF554362.1_Vibrio_root

TCTACTCGTTTGATTCTGCACTCTAAAGCGCAAAACACCATCATGGAA------ATG---
GCCGCCCAAGCCGGTACGGTTGAAGACATCGAA---
TTAGAAGATGTATTGAAAGTCGGTTTTGGCGATGTTCGCTGCGTTGAATCAGGCGGTCCAGAGCCAGGTGTTGGTT
GTGCTGGTCGTGGTGTTATCACC---GCTATCAACTTCCTCGAAGAAGAAGGTGCCTAC---GAAGACGATTTG---
GAC---------TTCGTATTCTATGACGTACTGGGT--

>AF016614.1_Unidentified_marine_4

TCAACTCGTCTCATCCTGCACTCAAAAGCACAAAACACCATCATGGAG------ATG---
GCAGCGGAAGCCGGTACGGTTGAAGACATCGAA---
TTAGAAGATGTATTGAAAGTCGGTTATGGCGATGTTCGTTGTGTTGAATCAGGCGGTCCAGAGCCAGGCGTAGGT
TGTGCCGGTCGCGGTGTTAACACA---GCAATCAACTTCCTCGAAGAAGAAGGTGCGTAT---GAAGAAGACTTA---
GAT---------TTCGTTTTCTACGACGTATTGGGTGACGTTGTGTGTGGTGGTTTCGCGATGCCAATTCGTGAAAAC---
AAAGCGCAAGAAATCTACATCGTATGTTCT

>AF111110.2_Vibrio_8

TCAACTCGTCTCATCCTGCACTCAAAAGCACAAAACACCATCATGGAA------ATG---
GCAGCGGAAGCCGGTACGGTTGAAGACATCGAA---
CTAGAAGATGTATTGAAAGTCGGTTATGGCGATGTTCGCTGTGTGGAATCAGGCGGCCCTGAGCCAGGCGTAGGT
TGTGCTGGTCGCGGTGTTATCACA---GCAATCAACTTCCTCGAAGAAGAAGGCGCGTAT---GAAGATGACTTA---
GAT---------TTCGTTTTCTACGACGTATTGGGTGACGTTGTGTGTGGTGGTTTCGCGATGCCAATTCGTGAAAAC---
AAAGCGCAAGAAATCTACATCGTATGTTCT

>KF861046.1_Uncultured_soil_15

TCCACCCGGCTGATCCTGCACTCCAAGGCGCAGACCTCGGTGATGGAA------AAA---
GCCGCTGATCTTGGCACCGTGGAAGATCTCGAA---
CTCGAGGACGTGCTCCAGGTCGGCTACAAGAACGTCAAGTGCGTGGAGTCCGGCGGCCCCGAGCCGGGCGTGGG
CTGCGCCGGCCGCGGCGTTATCACC---GCCATCAACTTCCTGGAAGAGGAAGGCGCTTAC---ACCGACGACCTG---
GAC---------TTCGTCTTCTACGACGTACTCGGAGATGTGGTCTGCGGCGGTTTCGCCATGCCCATCCGCGAAAAC---
AAGGCCCAGGAAATCTACATCGTCTGCTC-

>KF861055.1_Uncultured_soil_2

TCCACCCGGCTGATCCTGCACTCCAAGGCGCAGACCTCGGTGATGGAA------AAA---
GCCGCCGAGCTGGGCACCGTGGAAGATCTCGAG---
CTCGAGGACGTGCTCCAGGTCGGCTACAAGAACGTCAAGTGCGTGGAGTCCGGCGGCCCTGAGCCGGGTGTCGG
CTGCGCCGGCCGCGGCGTTATCACC---GCCATCAACTTCCTGGAAGAAGAAGGCGCCTAT---ACCGACGACCTG---
GAC---------TTCGTCTTCTACGACGTACTCGGCGACGTGGTCTGCGGCGGTTTCGCCATGCCCATCCGGGAAAAC---
AAGGCCCAGGAGATCTACATCGTCTGCTC-

>KF861175.1_Uncultured_soil_2

TCCACCCGTCTGATCCTGCACTCCAAGGCCCAGACCACCGTTATGGAA------ATG---
GCTGCCGAGATGGGCACCGTTGAAGATCTGGAA---
CTGGAAGATGTACTTCAGGTTGGCTACGGCAACGTCAAGTGCGTTGAGTCCGGTGGTCCTGAGCCCGGTGTTGGC
TGTGCCGGCCGTGGTGTAATCACC---GCCATCAACTTCCTGGAAGAAGAAGGCGCCTAC---ACCGACGACCTG---
GAC---------TTCGTCTTCTACGACGTACTGGGTGACGTGGTCTGCGGCGGTTTCGCCATGCCGGTCCGTGAGAAC---
AAGGCTCAGGAAATCTACATCGTTTGTTC-

>AY724121.2_Uncultured_soil_7

TCCACCCGTCTGATCCTGCACTCCAAGGCACAAACCACGGTCATGGAC------ATG---
GCCGCCGAAATGGGCACCGTGGAAGATCTCGAG---
CTGGAGGATGTACTCCAGGTTGGTTACAAGGACATCAAGTGCGTCGAGTCCGGCGGTCCTGAGCCGGGTGTGGGC
TGTGCCGGTCGCGGGGTAATCACC---GCCATCAACTTCCTCGAAGAGGAAGGCGCCTAC---ACCGACGACCTG---
GAC---------TTTGTCTTCTACGACGTACTGGGTGACGTGGTTTGTGGTGGTTTCGC--------------------------------------
----

>AY724144.2_Uncultured_soil_2

TCCACCCGTCTGATCCTGCACTCCAAGGCACAAACCTCGGTCATGGAG------ATG---
GCCGCCGAGATGGGCACCGTGGAAGATCTCGAG---
CTGGAGGATGTACTCCAGGTTGGTTACAAGGACATCAAGTGCGTCGAGTCCGGCGGTCCTGAGCCGGGTGTGGGT
TGTGCCGGTCGCGGGGTAATCACC---GCCATCAACTTCCTCGAAGAGGAAGGGGCCTAC---ACCGACGACCTG---
GAC---------TTCGTCTTCTACGACGTACTGGGGGATGTGGTCTGCGGCGGCTTCGC-------------------------------------
----

>JF701923.1_Alteromonadales_cryopeg

------------ATGCTGCATGCCAAAGCGCAAAACACCATTATGGAA------ATG---
GCCGCTGAACAGGGGTCGGTTGAAGATCTTGAA---
TTAGAAGATGTGCTCAAAGTCGGTTACGGCGGGGTGAAATGCGTTGAATCAGGTGGCCCTGAGCCGGGCGTTGGT
TGTGCGGGTCGTGGGGTTATCACC---GCAATTAACTTCCTTGAAGAAGAAGGCGCTTAT---GACGAAGACCTC---
GAT---------TTTGTTTTCTATGACGTGCTTGGGGATGTGGTTTGCGGTGGATTTGCCATGCCAATTCGTGAAAAC---
AAAGCGCAAGAAATTTACATCGT-------

>AF046853.1_Marine_microbial mat_5

TCTACTCGACTTATGCTTCACGCAAAAGCTCAGAACACCATTATGGAA------ATG---
GCTGCTGAAGCGGGTTCCGTTGAAGATCTTGAA---
CTTGAAGATGTTCTAAAAGTTGGATACGGCGGGGTTAAATGTGTTGAATCAGGTGGCCCAGAGCCAGGCGTTGGT
TGTGCTGGTCGTGGGGTTATCACA---GCGATTAACTTCCTCGAAGAAGAAGGCGCTTAC---GATGATGACTTA---
GAC---------TTTGTTTTTTATGACGTACTTGGTGACGTTGTATGCGGTGGGTTCGCAATGCCTATTCGCGAAAAC---
AAAGCGCAAGAAATCTACATCGTATGTTCT

>DQ913883.1_Celerinatantimonas_5

TCTACTCGCCTTATGCTTCACGCGAAAGCTCAGAACACCATTATGGAA------ATG---
GCCGCTGAAGCAGGTTCGGTTGAAGATCTTGAA---
CTAGAGGATGTGCTAAAAGTTGGCTACGGCGGGGTGAAATGCGTTGAATCAGGTGGCCCAGAGCCAGGTGTTGG
TTGTGCTGGTCGTGGGGTTATCACA---GCGATTAACTTCCTCGAAGAAGAAGGCGCTTAC---GATGATGACTTA---
GAC---------TTTGTTTTTATGACGTACTAGGTGACGTTGTATGCGGCGGGTTCGCAATGCCTATTCGCGAAAAC---
AAAGCGCAGGAAATCTACATCGT-------

>EF174730.1_Uncultured_marine_8

------------ATCCTGCACTCAAAAGCGCAAAGCACCATCATGGAA------GCG---
GCGGCAGAGCAAGGTTCTGTTGAGGATATCGAA---
CTAGAAGATGTATTGAAAGTCGGTTATGGCGATGTGCGCTGTGTGGAATCAGGCGGTCCTGAGCCAGGTGTTGGC
TGTGCAGGTCGTGGGGTAATCACG---GCGATCAACTTCCTTGAAGAAGAGGGTGCTTAC---GAAGAAGAGCTT---
GAT---------TTCGTGTTCTACGACGTACTGGGTGACGTGGTATGTGGTGGTTTTGCAATGCCAATTCGTGAGAAC---
AAAGCGCAAGAGATCTACATCGTT------

>EF568567.1_Uncultured_Mediterranean_22

TCTACCCGTTTGATTCTGCATTCAAAAGCACAAACTACTATTATGCAC------CTG---
GCTGCTGAAGCAGGCAGTGTTGAAGATTTGGAG---
CTGGAAGATGTATTGAAAGTGGGCTACGGCGATGTGAAATGCGTCGAGTCTGGTGGCCCTGAGCCGGGTGTTGGT
TGTGCCGGTCGCGGTGTTATCACG---GCGATCAACTTCCTGGAGGAAGAAGGCGCTTAC---GAAGATGACCTG---
GAT---------TTCGTTTTCTACGATGTATTGGGTGACGTTGTTTGTGGTGGTTTCGCGATGCCGATTCGTGAAAAC---
AAGGCGCAAGAGATTTACATCGTTTGTTC-

>HM801245.1_Uncultured_marine_11

------------ATTCTTCACTCAAAAGCTCAGAATACCATTTTGGAA------ATG---
GCAGCTGAAGCGGGAACGGTTGAAGATCTTGAA---
TTAGAAGACGTATTAAAAGCCGGTTACGGCGATATCAAATGCGTTGAATCTGGTGGTCCAGAACCCGGTGTTGGTT
GTGCTGGTCGCGGTGTTATTACT---GCGATTAACTTCTTAGAAGAAGAAGGTGCATAC---GAAGATGATCTT---GAT-
--------TTTGTTTTCTACGACGTATTAGGCGACGTTGTGTGCGGTGGATTCGCGATGCCAATTCGTGAAAAC---
AAAGCGCGAGAAATCTACATCGTATGCTCT

>M63691.1_Klebsiella_2

TCAACCCGGCTGATTCTTCACTCTAAAGCGCAAAACACGATTATGGAA------ATG---
GCTGCTGAAGCTGGCTCTGTTGAAGATATCGAA---
CTGGAAGATGTATTGAAAGTCGGTTACGGCGACGTGCGCTGTGTTGAGTCTGGTGGTCCTGAGCCTGGTGTTGGC
TGTGCCGGTCGCGGGGTGATTACG---GCAATTAACTTCCTTGAAGAAGAAGGTGCTTAC---GAAGAAGATCTG---
GAC---------TTTGTGTTCTATGACGTTCTTGGTGACGTTGTGTGTGGTGGTTTCGCGATGCCAATTCGTGAAAAC---
AAAGCACAGGAAATCTATATCGT-------

>HM210381.1_Uncultured_marine_2

TCTACACGTTTAATCCTGCACTCCAAAGCACAGAACACCATTATGGAA------ATG---
GCTGCTGAAGCAGGCACTGTTGAAGATCTGGAA---
CTCGAAGATGTATTAAAAGTGGGCTACGGCGACTTCAAGTGCGTTGAGTCCGGCGGTCCTGAGCCTGGTGTTGGTT
GTGCCGGTCGCGGCGTAATCACT---GCGATCAACTTCCTGGAAGAAGAAGGCGCTTAT---GACGAAGACCTG---
GAT---------TTCGTTTTCTACGATGTACTGGGTGACGTTGTATGCGGAGGGTTCGCAATGCCGATTCTTGAAAAC---
AAGGCACAGGAAATTTACATCGTT------

>HM750333.1_Uncultured_rhizosphere_4

TCCACACGTCTGATTCTGCATGCCAAGGCACAGAACACCATTATGGAA------ATG---
GCGGCTGAAGCCGGTACCGTTGAAGATCTCGAA---
CTCGAAGATGTATTAAAGAACGGTTACGGCGACATCAAACGTGTTGAGTCCGGTGGTCCTGAGCCAGGTGTTGGC
TGTGCCGGCCGTGGTGTGATCACC---GCCATCAACTTCCTCGAAGAGGAAGGAGCATAC---GAAGAAGACCTC---
GAC---------TTTGTTTTCTACGACGTACTCGGTGACGTTGTCTGCGGTGGCTTCGCCATGCCTATCCGTGAAAAC---
AAGGCGCAGGAGATCTACATCGT-------

>AF216886.1_Unidentified_marine rhizosphere_5

TCCACTCGTCTGATTCTTCATTCCAAGGCGCAGAACACCATCATGGAG------ATG---
GCTGCTGAGGCCGGTACGGTTGAAGACCTGGAA---
CTTGAAGATGTATTGAAAACCGGCTACGGCGACATCAAGTGCGTTGAGTCTGGTGGCCCTGAGCCAGGTGTTGGC
TGCGCGGGTCGCGGTGTCATCACT---GCCATCAACTTCCTCGAAGAGGAAGGTGCCTAC---GAAGAAGACCTC---
GAC---------TTCGTTTTCTACGACGTACTGGGTGATGTTGTGTGTGGTGGTTTCGCCATGCCTATTCGTGAAAAC---
AAGGCACAGGAAATCTACATCGT-------

>FJ394951.1_Uncultured_rhizosphere_6

TCCACTCGTCTGATTCTTCATTCCAAGGCGCAGAACACCATCATGGAG------ATG---
GCTGCTGAGGCCGGTACGGTTGAAGACCTGGAA---
CTTGAAGATGTATTGAAGACCGGCTACGGCGACATCAAGTGCGTTGAGTCCGGTGGCCCTGAGCCTGGTGTTGGC
TGCGCGGGTCGCGGTGTAATCACT---GCCATCAACTTCCTGGAAGAGGAAGGTGCATAC---GATGAAGACCTC---
GAC---------TTCGTTTTCTATGATGTACTTGGTGATGTTGTGTGTGGTG---TCGGCATGCCTATCCGTGAAAAC---
AAGGCCCAGGAAATCTACATCGT-------

>HQ455840.1_Uncultured_South China Sea_11

TCTACTCGTCTGATCCTGCACGCCAAGGCACAGAACACCATCATGGAG------ATG---
GCGGCCGAGAAGGGCTCCGTTGAAGATCTGGAA---
CTCGAAGATGTGCTGCAAATCGGTTACGGCGGTGTACGTTGTGCCGAATCTGGTGGTCCTGAGCCAGGAGTCGGT
TGTGCTGGCCGTGGTGTTATCACT---GCCATCAACTTCCTCGAAGAAGAGGGCGCCTAC---GATGCCGATCTG---
GAC---------TTTGTTTTCTACGACGTACTCGGTGACGTAGTGTGTGGCGGTTTCGCCATGCCAATTCGTGAAAAC---
AAGGCTCAGGAAATCTACATCGTCTGCTC-

>CP001616.1_Tolumonas_freshwater sediment

TCTACTCGTTTGATCCTGCACGCAAAAGCCCAGAATACCATCATGGAA------ATG---
GCAGCAGAAGTTGGTTCTGTTGAAGATCTGGAA---
CTGGAAGACGTATTACAGATCGGTTACGGCGGAGTTCGTTGTGCTGAGTCTGGTGGCCCAGAGCCAGGAGTTGGT
TGTGCAGGTCGTGGTGTTATCACT---GCTATCAACTTCCTGGAAGAAGAAGGCGCGTAC---GAAGAAGATTTA---
GAC---------TTCGTATTCTACGACGTACTGGGTGACGTAGTGTGTGGCGGTTTCGCTATGCCAATCCGTGAAAAC---
AAAGCTCAGGAAATCTACATCGTTTGCTC-

>GU196952.1_Uncultured_marine dinoflagellate_12

TCTACCCGTTTGATCCTTCACGCCAAGGCCCAGAGTACCATCATGGAA------ATG---
GCTGCTGAAGCCGGCTCTGTGGAAGATCTGGAA---
CTCGAAGATGTACTGCAGATCGGTTACGGCAACGTCCGTTGCGCCGAGTCTGGTGGTCCTGAGCCAGGAGTTGGT
TGTGCCGGTCGTGGTGTTATCACC---GCCATCAACTTCCTGGAAGAAGAAGGTGCCTAC---GAAGAAGATCTG---
GAC---------TTCGTATTCTACGACGTACTGGGTGACGTAGTGTGTGGTGGTTTCGCCATGCCAATCCGTGAAAAC---
AAGGCTCAGGAAATCTACATCGTCTGCTC-

>DQ982428.1_Uncultured_root_10

------------ATCCTGCACGCCAAGGCCCAGAACACCATCATGGAA------ATG---
GCCGCTGAAGTCGGCTCCGTGGAAGATCTGGAA---
CTTGAAGACGTGCTGCAGATCGGTTACGGCGGCGTGCGCTGTGCCGAGTCTGGTGATCCGGAGCCAGGAGTAGGT
TGTGCCGGTCGTGGTGTTATCACC---GCCATCAACTTCCTGGAAGAAGAAGGCGCCTAC---GCCGACGATCTG---
GAC---------TTCGTGTTCTATGACGTACTGGGTGACGTAGTGTGTGGCGGTTTCGCCATGCCCATCCGCGAAAAC---
AAGGCCCAAGGAAATCTACATCG-T-------

>DQ426028.1_Uncultured_stem_42

---ACCCGCCTGATCCTGCACGCCAAGGCCCAGAATACCATCATGGAA------ATG---
GCCGCGGAAAAGGGTTCTGTAGAGGATCTGGAA---
CTCGAAGACGTGCTGCAGATCGGTTACGGCGGCGTGCGCTGTGCCGAGTCTGGTGGTCCTGAGCCAGGAGTTGGT
TGTGCCGGTCGTGGTGTTATCACC---GCCATCAACTTCCTGGAAGAAGAAGGCGCGTAT---GAAGCCGACCTC---
GAC---------TTCGTATTCTATGACGTGCTGGGTGACGTGGTGTGTGGCGGTTTCGCCATGCCAATCCGCGGAAAA---
ACAAGGCCC-AGGAAATCTACATCG-T-------

>HQ606011.1_Uncultured_marine_4

TCTACCCGTCTGATCCTGCACGCCAAGGCCCAGAATACCATCATGGAA------ATG---
GCCGCAGAAAAGGGTTCTGTAGAAGATCTGGAA---
CTCGAAGACGTGCTGCAGATCGGTTACGGCGGTGTGCGCTGTGCCGAGTCTGGTGGTCCTGAGCCAGGAGTTGGT
TGTGCCGGTCGTGGTGTTATCACC---GCCATCAACTTCCTGGAAGAAGAAGGCGCGTAT---GAGGCCGACCTC---
GAC---------TTCGTATTCTACGACGTGCTGGGTGACGTGGTGTGTGGCGGTTTCGCCATGCCAATCCGCGAAAAC---
AAGGCACAAGAAATCTACATCGTCTGCTCT

>HM601497.1_Uncultured_marine_4

TCGACCCGTTTGATCCTGCACTCCAAAGCACAAAACACCATCATGCAG------ATG---
GCCGCTGATGCAGGTTCTGTTGAAGACCTCGAA---
TTGGAAGACGTTCTGAAAGTCGGTTACGGCGATATCGCATGCGTCGAATCCGGCGGTCCGGAACCGGGCGTTGGT
TGCGCTGGTCGTGGTGTTATCACC---GCCATCAACTTCTTGGAAGAAGAAGGCGCATAT---GAAGAAGATCTG---
GAC---------TTCGTATTCTACGACGTTCTGGGCGACGTTGTTTGCGGTGGTTTCGCTATGCCGATCCGCGAAAAC---
AAAGCTCAAGAGATCTACATCGTTTGCTC-

>FJ394944.1_Uncultured_rhizosphere_4

TCTACTCGTCTGATTCTTCACGCTAAGGCTCAGAATACTATTATGGAA------ATG---
GCTGCTGAAGCAGGCACAGTTGAAGATCTGGAA---
CTGGAAGATGTACTGAAAGTAGGATACCGCGACATCAAATGTGTTGAATCAGGTGGTCCTGAGCCAGGTGTTGGC
TGCGCCGGCCGTGGTGTAATCACT---GCGATTAACTTCCTCGAGGAAGAGGGTGCATAC---GAAGAAGACCTG---
GAC---------TTCGTGTTCTATGACGTACTGGGTGACGTTGTATGCGGTGGCTTCGCTATGCCGATTCGTGAAAAC---
AAGGCGCAGGAAATCTACATCGT-------

>HE599439.1_Uncultured_marine sediment_2

---GCGGGTACGGTTGAAGATTTGGAA---
CTGGAAGACGTACTCAAAACCGGTTACGGCGAAATCAAGTGCGTTGAGTCTGGCGGCCCTGAGCCTGGCGTGGGT
TGCGCTGGTCGCGGTGTAATCACC---GCAATTAACTTCCTTGAAGAGGAAGGCGCTTAT---GAAGAAGACTTG---
GAT---------TTCGTATTCTACGACGTGCTGGGTGACGTGGTCTGCGGTGGCTTCGCCATGCCGATTCGCGAAAAC---
AAAGCACAGGAAATTTATATCGTTTGCTC-

>EF568414.1_Uncultured_Mediterranean_8

TCAACTCGTTTAATTCTTCACTCTAAAGCTCAAACCACTGTTATGCAC------TTG---
GCTGCTGAAGCTGGTACGGTAGAAGATTTAGAA---
CTCGAAGATGTATTGTCTGTTGGTTACGGTGATGTTAAGTGCGTTGAGTCTGGTGGCCCTGAGCCAGGCGTTGGCT
GCGCTGGCCGAGGCGTAATTATC---GCGATCAACTTTCTTGAAGAAGAAGGTGCATAC---GATGAAGACCTA---
GAC---------TTCGTATTTTACGATGTGTTGGGTGACGTTGTATGCGGCGGTTTCGCGATGCCGATTCGTGAAAAC---
AAAGCGCAAGAAATTTACATCGTATGTTC-

>AF046832.1_Marine_microbial mat_4

TCAACTCGTCTTATCCTACACACTAAGATGCAAAACACCATCATGGAA------ATG---
GCAGCGGAAGCCGGCACCGTTGAAGACATCGAA---
CTAGAAGATGTATTACTCACTGGTTACAGCGGCATTAAATGTGTTGAGTCAGGTGGCCCAGAGCCTGGCGTTGGTT
GTGCGGGTCGCGGTGTTATCACA---GCAATCAATTTCCTAGAAGAAGAAGGAGCATAC---GAAGAAGATTTA---
GAT---------TTTGTTTTTTACGATGTATTGGGTGACGTTGTGTGTGGTGGTTTCGCGATGCCAATTCGTGAAAAT---
AAAGCACAAGAAATCTATATCGTATGTTCT

>AF046827.1_Marine_microbial mat_5

TCAACTCGTCTTATCGTACACACTAAGATGCGAAATACCATCATGGAA------ATG---
GCAGCGGAAGCCGGTACTGTTGAAGACATCGAA---
TTAGAAGATGTACTACTGACTGGTTACGGTGACGTTAAATGTGTTGAGTCAGGCGGTCCAGAGCCAGGCGTTGGTT
GCGCTGGTCGTGGTGTAATTACG---GCGATCAACTTCCTGGAAGAAGAAGGTGCGTAC---GATGAAGATCTA---
GAT---------TTCGTATTCTACGATGTTCTCGGTGACGTTGTCTGTGGTGGTTTCGCGATGCCAATTCGTGAAAAC---
AAAGCGCAAGAAATCTACATAGT------T

>AF046831.1_Marine_microbial mat_2

TCAACCCGCCTGATTATACACACTAAGATGCAAAACACCATCATGGAA------AAG---
GCAGCGGAAGCCGGTACTGTTGAAGACATCGAG---
CTAGAAGATGTACTACTAACTGGTTACGGTGACGTTAAATGTGTTGAGTCAGGCGGTCCAGAGCCAGGCGTTGGTT
GTGCAGGTCGTGGTGTAATCACA---GCGATCAACTTTCTGGAAGAAGAAGGTGCGTAC---GATGAAGACCTG---
GAT---------TTCCTATTTTACGATGTTCTTGGTGACGTTGTGTGTGGTGGTTTCGCGATGCCAATTCGTGAAAAC---
AAAGCGCAAGAAATCTACATCGT------T

>EF203422.1_Vibrio

TCAACTCGTCTTATTCTACACACGAAGATGCAAAACACCATCATGGAA------ATG---
GCAGCGGAAGCCGGTACTGTGGAAGACATCGAA---
CTAGAAGATGTACTACTCACTGGTTACGGTGACGTTAAATGTGTTGAGTCAGGCGGTCCTGAGCCAGGCGTTGGTT
GTGCGGGTCGTGGTGTAATCACA---GCGATCAACTTCCTGGAAGAAGAAGGTGCGTAC---GACGAAGATCTA---
GAT---------TTCGTATTTATCGATGTTCTTGGTGACGTTGTGTGTGGTGGTTTCGCGATGCCAATTCGTGAAAAC---
AAAGCACAAGAAATCTACATCGT------T

>EU151775.1_Uncultured_marine_06

TCAACTCGTCTTATCCTACACACTAAGATGCAAAACACCATCATGGAA------ATG---
GCAGCGGAAGCCGGTACTGTTGAAGACATCGAA---
CTAGAAGATGTACTACTCACTGGTTACGGTGACGTTAAATGTGTTGAGTCAGGCGGTCCAGAGCCAGGCGTTGGTT
GTGCTGGTCGTGGTGTAATCACA---GCGATCAACTTCCTGGAAGAAGAAGGTGCATAC---GAAGAAGACCTG---
GAT---------TTCGTATTCTATGATGTTCTTGGTGACGTTGTGTGTGGTGGTTTCGCGATGCCAATTCGTGAAAAC---
AAAGCACAAGAAATCTACATCGT------T

>HQ455942.1_Uncultured_South China Sea_4

TCAACTCGTCTTATCCTACACACTAAGATGCAAAACACCATCATGGAA------ATG---
GCAGCGGAAGCCGGTACTGTTGAAGACATCGAA---
CTAGAAGATGTACTACTCACTGGTTACGGTGACGTTAAATGTGTTGAGTCAGGCGGTCCAGAGCCAGGCGTTGGTT
GTGCAGGTCGTGGTGGAATCACA---GCGATCAACTTCCTAGAAGAAGAAGGTGCATAC---GAAGAAGACCTA---
GAT---------TTCGTATTCTACGACGTTCTTGGTGACGTTGTATGTGGTGGTTTCGCGATGCCTGTTCGTGAAAAC---
AAAGCGCAAGAAATCTACATCGT------T

>EF468423.1_Uncultured_marine_5

TCAACTCGTCTAATTCTACACACTAAAATGCAAAACACCATCATGGAA------ATG---
GCGGCTGAAGCAGGTACTGTAGAAGATATCGAA---
CTAGAAGATGTACTACTAACTGGTTACGGCGATGTGAAATGTGTTGAATCAGGCGGTCCAGAGCCAGGTGTTGGT
TGTGCAGGTCGTGGTGTTATCACA---GCGATTAACTTCTTAGAAGAAGAAGGCGCATAT---GAAGAAGACCTG---
GAC---------TTCGTATTCTACGATGTACTTGGTGACGTTGTTTGCGGCGGCTTTGCCATGCCAAT-----------------------
----

>JX064477.1_Uncultured_marine_2

TCAACTCGTCTAATTCTACATACTAAAATGCAAAACACCATCATGGAA------ATG---
GCGGCTGAAGCTGGTACTGTGGAAGATATCGAA---
CTAGAAGATGTACTACTAACTGGTTACAGTGATATTAAATGTGTTGAATCAGGTGGTCCAGAACCAGGCGTTGGTT
GCGCAGGCCGTGGTGTTATCACA---GCGATCAACTTCCTAGAAGAAGAAGGCGCATAC---GATGATGAACTA---
GAC---------TTCGTATTCTACGATGTATTGGGTGACGTGGTATGTGGTGGCTTCGCGATGCCAATTCGTGAAAAT---
AAAGCTCAAGAAATCTACATCGTATG----

>GU196870.1_Uncultured_marine dinoflagellate_3

TCGACTCGTCTTATTTTACACGCGAAAGCTCAAAATACCATTATGGAA------ATG---
GCTGCTGAAGCGGGTTCTGTTGAAGACATCGAA---
CTAGAAGATGTACTAAAAGTGGGTTACGGCGGTGTTCGTTGTGTTGAATCAGGCGGTCCAGAGCCAGGTGTTGGT
TGTGCCGGTCGTGATGTAATTACA---GCGATTAACTTCCTAGAAGAAGAAGGCGCTTAC---GAAGAAGATTTA---
GAC---------TTCGTATTCTACGACGTACTAGGTGACGTTGTGTGTGGTGGTTTCGCCATGCCTATTCGTGAAAAC---
AAAGCTCAAGAAATCTATATCGT-------

>AF389822.1_Uncultured_marine seagrass_6

TCCACCCGTCTGATGCTGCATTCAAAAGCACAGAACACGATCATGGAA------ATG---
GCAGCGCAGGCCGGCACCGTGGAAGACTTGGAA---
CTGGAAGATGTACTCAAAGTCGGTTATGGCGACGTGAAGTGCGTTGAGTCCGGTGGTCCGGAGCCAGGCGTTGGT
TGTGCAGGTCGTGGTGTTATCACC---GCCATCAACTTCCTCGAAGAAGAAGGCGCATAC---GATGAAGACCTC---
GAC---------TTCGTATTTTACGATGTACTGGGTGACGTTGTGTGTGGTGGTTTCGCCATGCCGATTCGTGAAAAC---
AAGGCCCAGGAAATCTACATCGT-------

>KF151567.1_GammaETSP1_marine_136

TCAACTCGTCTGATCCTTCACTCAAAAGCTCAAAACACAATCATGGAA------ATG---
GCTGCCGAAGCCGGTACCGTGGAAGATCTTGAG---
TTAGAAGATGTATTAAAAATGGGTTACGGCAACGTTAAGTGCGTTGAGTCCGGTGGTCCAGAGCCAGGTGTTGGT
TGTGCCGGCCGTGGTGTTATCACT---GCTATCAACTTCTTAAAAGAAGAAGGTGCTTAC---GACGATGACCTA---
GAC---------TTCGTATTCTACGATGTATTGGGTGACGTGGTATGTGGTGGATTCGCCATGCCCATTCGTGAGAAC---
AAAGCGCAAGAAATCTACATTGTTTGTTCT

>HF559580.1_Uncultured_wood_2

------------ATTCTCCATTCCAAGGCCCAGAACACCATCATGGAA------ATG---
GCGGCTGAAGCGGGCACCGTGGAAGATCTGGAA---
CTTGAAGATGTACTCAAGGCCGGTTACATGGGGATCAAATGTGTCGAGTCCGGTGGCCCGGAGCCAGGTGTTGGC
TGTGCCGGCCGCGGTGTAATCACC---GCCATCAACTTCCTTGAAGAGGAGGGCGCCTAC---GACGAAAACCTG---
GAC---------TTCGTGTTCTACGACGTACTCGGTGATGTGGTGTGCGGCGGGTTCGCCATGCCGATCCGCGAGAAC---
AAGGCCCAGGAAATCTACATCGTCTG----

>HM210359.1_Uncultured_marine_2

TCAACACATCTAATCCTTCACTCTAAAGCTCAAACTACGGTAATGCAC------TTG---
GCTGCTGAAGCGGGTACTGTAGAAGATCTTGAG---
CTCGAAGATGTATTGTCTGTAGGCTACGGCGACGTTAAGTGTGTTGAGTCTGGTGGCCCAGAGCCAGGTGTTGGTT
GTGCGGGCCGTGGTGTTATCACT---GCAATTAACTTTCTTGAAGAAGAAGGTGCTTAT---GACGAAGATCTT---GAC-
--------TTCGTATTCTACGATGTACTAGGTGATGTTGTATGTGGTGGTTTTGCGATGCCTATTCGTGAAAAC---
AAAGCACAAGAGATCTACATCGTTTGTTCT

>AY896428.1_GammaP_marine_2

TCTACTCGTCTGATCCTTCACTCTAAAGCTCAAACTACTGTTATGCAC------TTG---
GCTGCAGAAGCAGGCACGGTAGAAGACCTGGAG---
CTGGAAGATGTACTGTCTGTTGGCTACGGCGACGTTAAATGCGTTGAGTCTGGTGGCCCTGAGCCAGGTGTTGGTT
GTGCAGGTCGTGGTGTAATCACT---GCAATCAACTTCCTGGAAGAAGAAGGTGCCTAT---GACGAAGACCTA---
GAC---------TTCGTATTCTACGACGTATTGGGTGACGTTGTATGTGGTGGTTTCGCGATGCCTATTCGTGAAAAC---
AAAGCACAAGAAATCTACATCGTATGTTCT

>HM210343.1_Gamma2_marine_4

TCAACTCGTCTGATCCTTCACTCTAAAGCTCAAACTACTGTTATGCAT------CTG---
GCTGCTGAGGCCGGTACCGTAGAAGACCTGGAG---
CTGGAAGATGTATTGTCTGTCGGTTACGGCGACGTTAAATGTGTTGAGTCTGGTGGTCCTGAGCCAGGCGTAGGTT
GTGCCGGTCGTGGTGTAATCACT---GCCATCAACTTCCTGGAAGAAGAAGGCGCTTAC---GACGAAGATCTG---
GAC---------TTCGTATTCTACGATGTACTGGGTGACGTTGTATGTGGTGGTTTCGCGATGCCAATTCGTGAAAAC---
AAAGCTCAAGAGATCTACATCGTATGTTCT

>AF216920.1_Unidentified_marine rhizosphere_5

TCTACTCGTTTGATTCTGCATTCTAAAGCACAGAATACCATCATGGAA------ATG---
GCCGCTGAAGCCGGCACCGTGGAAGATCTGGAA---
CTGGAAGATGTATTGAAGGTCGGCTACGGCGATATCAAATGTGTTGAATCAGGTGGTCCAGAGCCGGGTGTGGGT
TGTGCCGGCCGTGGTGTTATCACC---TCTATTAACTTCCTGGAAGAAGAAGGCGCCTAC---GACGAAGATCTC---
GAC---------TTCGTATTCTACGATGTGCTGGGTGACGTTGTCTGCGATGGTTTCGCCATGCCTATTCGTGAAAAC---
AAGGCACAGGAGATCTACATCGT-------

>EF568445.1_Uncultured_Mediterranean_2

TCAACGCGTTTGATTCTTCATTCTAAAGCACAAACAACTGTAATGCAC------TTG---
GCTGCTGAAGCAGGAACAGTCGAAGATCTGGAG---
CTCGAAGATGTATTGTCGGTAGGTTACGGCGATGTTAAGTGTGTTGAGTCTGGTGGTCCTGAGCCAGGTGTTGGTT
GTGCTGGTCGTGGTGTAATTACA---GCGATTAACTTTCTTGAAGAAGAAGGCGCCTAC---GATGAAGATCTC---
GAC---------TTCGTATTCTACGATGTATTGGGTGACGTTGTGTGTGGTGGTTTTGCGATGCCTATTCGTGAAAAT---
AAAGCGCAGGAAATTTACATCGTT------

>HM210363.1_Gamma4_marine_134

TCCACTCGTCTGATTCTTCACTCCAAAGCACAGAACACCATCATGGAA------ATG---
GCTGCTGAAGCCGGCACCGTGGAAGATCTGGAA---
CTGGAAGATGTATTAAAAGCCGGTTACGGCGACATCCGCTGTGTTGAATCCGGTGGCCCGGAGCCAGGTGTTGGA
TGTGCCGGTCGCGGTGTAATCACT---GCAATCAACTTCCTGGAAGAGGAAGGTGCATAT---GAAGATGACCTG---
GAC---------TTCGTATTCTACGATGTACTGGGCGATGTTGTATGCGGTGGCTTTGCTATGCCGATCCGTGAAAAC---
AAAGCGCAGGAAATCTACATTGT-------

>HM801623.1_Uncultured_marine_4

TCCACTCGTTTGATCCTTCACTCCAAAGCTCAGAACACCATCATGGAG------ATG---
GCTGCTGAAGCGGGCACTGTGGAAGATCTGGAA---
CTGGAAGACGTACTGAAAAGTGGTTACGGAGACATCCGTTGCGTTGAGTCCGGTGGTCCAGAGCCTGGTGTTGGT
TGCGCCGGTCGCGGTGTCATCACA---GCGATCAACTTCCCCGAAGAAGGAGGTGCTTAC---GAAGACGATCTG---
GAC---------TTCGTGTTCTACGACGTACTGGGTGATGTTGTGTGTGGTGGTTTCGCCATGCCGATTCGTGAGAAC---
AAAGCTCAGGAAATCTACATCGTT------

>GU193776.1_Uncultured_marine mat_2

TCTACCCGTCTTATTCTCCACGCCAAGGCACAAAACACCATCATGGAA------ATG---
GCTGCCGAGGCCGGCACCGTTGAAGATCTGGAA---
CTGGAAGACGTACTTAAGACCGGCTACGGCAACATTAAGTGCGTTGAGTCCGGTGGTCCTGAGCCAGGTGTCGGT
TGCGCTGGTCGTGGTGTTATCACC---GCCATCAACTTTCTGGAAGAAGAAGGCGCCTAC---GAGGAAGACCTC---
GAT---------TTCGTATTCTACGATGTACTGGGTGGCGTAGTGTGCGGTGGCTTCGCCATGCCGATCCGTGAAAAC---
AAGGCGCAGGAAATCTACATCGTTTGTTC-

>HM999196.1_Uncultured_marine_18

GGCTATGGCGATGTGAAATGTGTTGAATCAGGTGGTCCAGAGCCAGGTGTTGGTTGCGCTGGTCGCGGTGTAATT
ACA---GCGATCAACTTCCTTGAAGAGGAGGGCGCATAC---GAAGAAGACCTA---GAC---------
TTTGTATTCTACGATGTACTGGGTGACGTGGTATGTGGTGGTTTCGCAATGCCTATTCGTGAAAAC---
AAAGCTCAGGAAATCTACATCGTT------

>AY351672.1_Azotobacter

TCCACCCGCTTGATCCTCCACGCCAAGGCACAGAACACCATCATGGAG------ATG---
GCCGCTGAAGCCGGCACCGTGGAAGATCTGGAA---
CTGGATGACGTGCTCAAAACCGGTTATGCCGGCATCAAGTGCGTTGAATCCGGTGGCCCGGAGCCAGGTGTTGGC
TGCGCCGGCCGCGGCGTGATCACC---GCCATCAACTTCCTCGAAGAAGAGGGCGCCTAC---AGCGACGACCTC---
GAC---------TTCGTGTTCTACGACGTACTGGGTGACGTGGTTTGCGGTGGCTTCGCCATGCCGATCCGCGAAAAC---
AAGGCCCAGGAGATCTACATCGT-------

>EF133786.1_Uncultured_marine sediment_5

------------ATTCTGCACTCTAAAGCACAGAATACCATCATGGAG------ATG---
GCTGCTGAGGCGGGCACTGTAGAAGATCTGGAA---
CTGGAAGATGTACTTAAAGCCGGTTATGGCGACATTAAGTGTGTTGAATCAGGTGGTCCTGAGCCAGGTGTTGGTT
GTGCCGGTCGTGGTGTCATCACC---GCTATCAACTTCCTGGAAGAGGAAGGCGCATAC---GAGGAGGACCTA---
GAC---------TTCGTATTCTATGGCGTACTGGGTGACGTGGTTTGTGGTGGTTTCGCTATGCCGATTCGCGAGAAC---
AAAGCT-----------------------

>JN645330.1_Uncultured_marine sediment_2

TCAACCCGTTTGATTCTGCATTCTAAAGCGCAGAACACAATCATGGAG------ATG---
GCTGCCGAGGCAGGTACTGTTGAAGATTTGGAA---
CTGGAAGATGTACTTAAGGCCGGTTACGGCGACATTAAGTGTGTTGAGTCAGGTGGTCCTGAGCCAGGTGTTGGT
TGTGCTGGTCGTGGTGCAATCACC---GCTATCAACTTCCTTGAAGAGGAAGGTGCTTAC---GAAGAAGATCTG---
GAC---------TTCGTTTTCTATGACGTACTGGGTGACGTTGTATGTGGTGGTTTCGCGATGCCTATCCGCGAAAAC---
AAAGCACAGGAAATCTACATCGTATGTTC-

>DQ078038.1_Uncultured_marine sediment_4

TCTACTCGACTGATCCTTCACTCTAAAGCCCAAAACACCATTATGGAA------ATG---
GCTGCTGAAGCTGGCACCGTTGAAGATTTGGAA---
TTAGAAGATGTATTAAAAACTGGATACGGCGACATCAAGTGTGTTGAGTCCGGTGGTCCTGAGCCAGGTGTTGGTT
GTGCAGGTCGTGGTGTAATCACT---GCAATCAACTTCCTCGAAGAGGAAGGTGCATAC---GAAGACGATTTG---
GAT---------TTTGTTTTTTATGACGTACTGGGTGACGTTGTGTGCGGTGGCTTCGCTATGCCTATCCGTGAAAAC---
AAAGCACAGGAAATCTACATCGT-------

>DQ481281.1_Uncultured_marine plankton_5

TCTACCCGTCTTATCCTTCACTCTAAAGCACAAAACACCATCATGGAA------ATG---
GCCGCGGAAGCAGGCACAGTTGAAGATCTAGAA---
CTGGAAGATGTATTGAAAACCGGTTATGGCGATATCAAATGTGTTGAGTCCGGTGGTCCAGAGCCAGGTGTTGGT
TGTGCCGGCCGTGGTGTAATCACT---GCTATCAACTTCCTTGAGGAAGAAGGTGCATAC---GAAGACGATCTA---
GAC---------TTCGTATTCTACGACGTACTTGGTGACGTTGTATGTGGTGGTTTCGCTATGCCTATCCGCGAAAAC---
AAAGCACAAGAAATCTACATCGTT------

>DQ481278.1_Uncultured_marine plankton_9

------------ATCCTTCACTCTAAAGTCCAAAACACCATCATGGAA------ATG---
GCTGCCGAAGCTGGCACCGTTGAAGATCTGGAA---
CTGGAAGATGTATTAAAAACCGGTTACGGCGACATCAAGTGTGTTGAGTCCGGTGGTCCAGAGCCAGGTGTTGGC
TGTACCGGTCGCGGTGTAATCACT---GCAATCAACTTCCTCGAAGAAGAAGGTGCATAC---GAAGACGATCTT---
GAC---------TTTGTTTTCTACGACGTACTGGGTGACGTTGTATGTGGTGGATTCGCTATGCCTATCCGCGAAAAC---
AGAGCTCAAGAAATCTACATCGT-------

>HM601534.1_Uncultured_marine_5

TCAACGCGTTTGATCCTTCACTCTAAAGCTCAAAACACAATCATGGAA------ATG---
GCTGCGGAAGCAGGTACGGTTGAAGACCTAGAG---
TTGGAAGACGTATTAAAAACTGGTTACGGCGACATTAAGTGCGTTGAGTTAGGTGGTCCAGAGCCAGGTGTTGGT
TGTGCGGGTCGCGGTGTAATCACA---GCGATTAACTTCCTTGAAGAGGAAGGTGCTTAC---GAAGACGACCTA---
GAC---------TTCGTTTTCTACGACGTACTTGGTGACGTTGTATGTGGTGGTTTCGCCATGCCTATTCGTGAGAAC---
AAGGCACAAGAAATCTACATCGTT------

>KF151621.1_Uncultured_marine_12

TCAACGCGTTTGATTCTGCATTCTAAAGCACAAAACACCATCATGGAA------ATG---
GCAGCTGAAGCCGGTACGGTTGAAGACTTGGAG---
TTGGAAGACGTATTAAAAACGGGTTACGGCGACATCAAATGCGTTGAGTCTGGTGGTCCAGAGCCAGGTGTTGGT
TGTGCTGGTCGCGGTGTAATCACT---GCTATCAACTTCCTTGAGGAAGAAGGTGCATAC---GAAGACGATCTT---
GAC---------TTCGTATTCTACGACGTACTGGGTGACGTTGTATGTGGTGGTTTCGCGATGCCAATTCGTGAAAAC---
AAAGCTCAAGAAATCTACATCGTT------

>KF151661.1_GammaETSP3_marine_28

TCAACGCGTTTGATTCTGCACTCTAAAGCACAAAACACCATCATGGAA------ATG---
GCTGCTGAAGCCGGTACGGTTGAAGACCTAGAG---
TTGGAAGACGTATTAAAAACGGGCTACGGCGACATCAAGTGCGTTGAGTCTGGTGGTCCAGAACCAGGTGTTGGT
TGTGCTGGTCGCGGTGTAATCACT---GCTATCAACTTCCTTGAGGAAGAAGGTGCGTAC---GAAGACGATCTT---
GAC---------TTCGTATTCTACGACGTATTGGGTGACGTTGTATGTGGTGGTTTCGCGATGCCCATTCGTGAAAAC---
AAAGCTCAAGAAATCTACATCGTTGTATCT

>DQ481450.1_Uncultured_marine plankton_2

TCCACTCGTGGGATCCTTCACTCCAAAGCTCAAAACACCATTATGGAA------ATG---
GCTGCGGAAGCAGGCACTGTTGAAGATCTGGAA---
CTGGAAGATGTACTGAAGACTGGTTACGGCGACGTTCGCTGTGTTGAGTCTGGTGGTCCAGAGCCGGGTGTTGGT
TGTGCCGGTCGTGGTGTAATCACC---GCTATCAACTTCCTGGAAGAGGAAGGTGCCTAC---GAAGAACACCTG---
GAC---------TGCGTGTTCTACGACGTGCTGGGTGACGTAGTCTGTGGTGGTTTCGCGATGCCTATTCGTGAAAAC---
AAAGCACAGGAAATCTACATCGTT------

>DQ825712.1_Uncultured_marine_2

TCTACACGTCTGATTCTGCACACTAAAGCACAGAACACAATCATGGAG------ATG---
GCTGCTGAAGCTGGCACGGTTGAAGATCTGGAG---
CTGGAAGATGTATTGAAGGCTGGATACGGCGACATCAAGTGCGTTGAATCCGGTGGTCCAGAGCCAGGCGTTGGT
TGTGCAGGTCGTGGTGTAATCACG---GCGATCAACTTCCTGGAGGAGGAAGGTGCATAC---GAAGAAGATCTG---
GAT---------TTCGTTTTCTACGACGTACTGGGTGACGTTGTATGCGGTGGTTTCGCGATGCCTATCCGTGAAAAT---
AAAGCACAGGAAATCTACATCGTT------

>HM601505.1_Uncultured_marine_5

TCTACCCGTCTTATTCTTCACGCCAAAGCTCAAAACACCATTATGGAA------ATG---
GCTGCCGAAGCTGGAACCGTTGAAGACCTGGAA---
CTGGAAGACGTACTTAAAGTTGGCTACGGCGACGTACGCTGCGTTGAGTCCGGTGGTCCAGAGCCCGGTGTTGGT
TGTGCCGGTCGCGGTGTAATTACT---GCCATTAACTTCCTTGAGGAAGAAGGTGCGTAC---GAAGAAGACATT---
GAC---------TTTGTTTTCTACGACGTACTGGGTGACGTTGTGTGTGGTGGTTTCGCTATGCCCATTCGTGAAAAC---
AAAGCTCAGGAAATCTACATCGT-------

>EF631899.1_Uncultured_marine_27

TCAACTCGTCTGATTCTGCACTCAAAAGCTCAGAACACCATCATGGAA------ATG---
GCGGCTGAAGCTGGCACCGTTGAAGATCTCGAA---
CTGGAAGATGTACTGAAGTCCGGTTACGGCGATGTTCGCTGTGTTGAATCAGGTGGTCCAGAGCCAGGCGTTGGT
TGTGCAGGTCGTGGTGTTATCACG---GCGATTAACTTCCTCGAAGAGGAAGGTGCATAC---GAAGACGACCTC---
GAC---------TTCGTTTTCTATGACGTACTGGGTGACGTTGTTTGCGGTGGCTTCGCTATGCCTATCCGCGAAAAC---
AAAGCACAGGAAATCTACATCGTATGTTCT

>HQ455851.1_Uncultured_South China Sea_2

TCTACTCGTCTGATTCTGCACTCTAAAGCCCAGAACACCATCATGGAA------ATG---
GCTGCTGAAGCCGGTACCGTTGAAGATCTGGAA---
CTGGAAGATGTGCTGAAAAGCGGTTACGGCGACGTTCGTTGTGTTGAATCTGGCGGTCCAGAGCCCGGTGTTGGC
TGTGCTGGCCGTGGTGTAATCACT---GCGATCAACTTCCTGGAAGAAGAAGGCGCATAC---GAAGACGATCTG---
GAC---------TTCGTATTCTATGACGTACTGGGTGACGTTGTGTGTGGTGGTTTCGCCATGCCAATTCGTGAAAAC---
AAGGCTCAGGAAATCTACATCGTT------

>AF059643.1_Unidentified_marine_2

TCAACCCGTCTGATCCTGCACTCAAAAGCTCAGAACACCATCATGGAA------ATG---
GCTGCTGAAGCCGGCACCGTGGAAGATCTGGAA---
CTGGAAGATGTACTGAAAGCCGGTTACGGCGATATCCGTTGTGTTGAATCCGGTGGTCCAGAGCCAGGCGTAGGT
TGCGCTGGTCGTGGTGTTATCACC---GCCATTAACTTCCTGGAAGAAGAAGGCGCATAC---GAAGATGATCTG---
GAC---------TTCGTATTCTACGACGTACTGGGTGACGTTGTGTGTGGTGGTTTCGCGATGCCTATCCGTGAAAAC---
AAAGCTCAAGAAATCTACATCGTATGTTC-

>HM063825.1_Uncultured_marine sediment_4

TCAACGCGTTTGATCCTGCACTCAAAAGCCCAAAACACCATCATGGAA------ATG---
GCGGCAGAAGCTGGCACCGTGGAAGACCTGGAA---
CTGGAAGATGTATTGAAATCCGGTTACGGCGACGTCCGTTGTGTTGAGTCTGGCGGTCCAGAGCCAGGTGTTGGTT
GTGCCGGACGTGGTGTTATCACT---GCGATCAACTTCCTTGAAGAGGAAGGCGCTTAC---GAGGACGACCTC---
GAC---------TTCGTTTTCTACGACGTACTCGGTGACGTTGTGTGCGGTGGTTTCGCGATGCCTATTCGTGAAAAC---
AAAGCTCAGGAAATCTACATCGTTTGCTC-

>CP001614.2_Teredinibacter

TCTACCCGTCTTATCCTTCACGCCAAGGCTCAAAACACCATCATGGAA------ATG---
GCTGCAGAAGCCGGTACCGTTGAAGACCTGGAA---
CTGGAAGATGTATTGAAAGTTGGCTACGGCGACGTTAAGTGCGTTGAGTCCGGCGGTCCAGAGCCCGGTGTTGGC
TGTGCTGGTCGTGGTGTAATCACT---GCCATCAACTTCCTGGAAGAAGAAGGCGCTTAC---GAAGACGATCTC---
GAC---------TTCGTATTCTACGACGTACTGGGTGACGTTGTATGTGGTGGTTTCGCTATGCCTATTCGTGAAAAC---
AAGGCTCAGGAAATCTACATCGTT------

>JN645307.1_Uncultured_marine sediment_5

TCAACCCGTCTGATCCTGCATTCAAAGGCTCAGAACACCATCATGGAA------ATG---
GCTGCGGAAGCCGGCACGGTGGAAGATCTGGAA---
CTGGAAGATGTATTAAAAGCCGGTTACGGCGACATCAAGTGTGTTGAGTCCGGTGGCCCAGAGCCCGGTGTTGGT
TGTGCCGGTCGTGGTGTGATCACG---GCCATTAACTTCCTCGAAGAGGAAGGTGCTTAC---GAAGACGATCTG---
GAT---------TTCGTGTTCTACGACGTGCTGGGTGACGTTGTTTGCGGTGGCTTCGCGATGCCGATCCGTGAAAAC---
AAGGCTCAGGAAATCTACATCGTATGTTC-

>KF151785.1_Uncultured_marine_6

TCCACTCGTCTGATCCTGCACTCAAAGGCTCAGAACACCATCATGGAA------ATG---
GCTGCGGAAGCAGGCACCGTGGAAGATCTGGAA---
CTGGAAGATGTATTGAAAGCTGGTTACGGCGACATCAAGTGTGTTGAGTCCGGCGGTCCAGAGCCAGGTGTTGGT
TGTGCAGGTCGTGGTGTTATCACT---GCAATTAACTTCCTCGAAGAGGAAGGCGCTTAC---GAAGACGATCTG---
GAT---------TTCGTTTTCTACGACGTTCTGGGTGACGTTGTATGTGGTGGTTTCGCGATGCCTATTCGTGAAAAC---
AAGGCTCAGGAAATCTACATCGTT------

>EF196650.1_Uncultured_marine sediment_2

TCCACCCGCCTGATCCTGCACTCCATATCCCAGAACACCGTGATGGAA------ATG---
GCCGCCCAGGCCGGTACCGTCGAGGATCTGGAG---
CTGGAAGACGTGCTGCAGGTCGGTTATGGCGACGTCAAGTGCGTCGAGTCCGGCGGCCCGGAGCCGGGCGTTGG
CTGCGCCGGTCGCGGCGTGATCACC---GCGATCAACTTCCTTGAGGAAGAGGGTGCCTAC---AGCGAGGATCTG---
GAC---------TTCGTGTTCTACGACGTACTGGGTGACGTGGTGTGCGGCGGTTTCGCCATGCCGATCCGCGAGAAC---
AAGGCCCAGGAGATCTACATCGTCTGCTC-

>HQ335630.1_Uncultured_soil_2

TCCACCCGCCTGATCCTGCACTCGAAGGCTCAGAACACCATCATGGAA------ATG---
GCTGCCGAAGCCGGCACCGTGGAAGATCTGGAA---
TTGGAAGATGTGTTGAAGGTCGGCTACGGCGACATCAAGTGCGTCGAGTCCGGTGGTCCTGAGCCGGGCGTCGGT
TGTGCCGGCCGTGGCGTTATCACC---GCCATCAACTTCCTGGAAGAAGAAGGCGCCTAC---GACGAAAATCTC---
GAC---------TTCGTGTTCTACGACGTGTTGGGTGACGTGGTGTGCGGCGGTTTCGCCATGCCCATTCGCGAAAAC---
AAGGCGCAGGAAATCTACATCGT-------

>AF216884.1_Unidentified_marine rhizosphere_18

TCTACTCGTCTGATCCTGCACTCCAAGGCCCAGAACACCATCATGGAA------ATG---
GCTGCCGAAGCCGGTACCGTGGAAGATCTGGAG---
CTGGAAGATGTGATGAAGGTCGGTTACGGCGACATCAAGTGCGTCGAGTCCGGTGGTCCTGAGCCGGGTGTTGGC
TGTGCCGGTCGTGGCGTTATCACT---TCCATCAACTTCCTGGAAGAAGAAGGCGCCTAT---GACGAAGACCTG---
GAC---------TTCGTGTTCTATGACGTGTTGGGCGACGTGGTGTGCGGTGGTTTCGCCATGCCTATCCGTGAGAAC---
AAGGCCCAGGAAATCTACATCGT-------

>FJ394926.1_Uncultured_rhizosphere_3

TCTACTCGCCTGATCCTGCACTCCAAGGCTCAGAATACTATCATGGAA------ATG---
GCTGCTGAAGCCGGCACCGTGGAAGATCTGGAA---
CTGGAAGATGTGTTGAAGGTTGGCTACGGCGACATCAAGTGCGTCGAGTCTGGTGGTCCTGAGCCGGGTGTTGGC
TGTGCCGGTCGTGGCGTTATCACT---TCCATCAACTTCCTGGAAGAAGAAGGCGCCTAT---GACGAAGACCTG---
GAC---------TTCGTGTTCTATGACGTGTTGGGCGACGTGGTGTGCGGTGG--TCGCC------
ATCCATCACTCGTGAAAACAAGGCCCAGGAAATCTACATCGT-------

>HQ456101.1_Uncultured_South China Sea_5

TCCACCCGTTTGATCCTTCACTCCAAGGCTCAGAACACCATTATGGAA------ATG---
GCAGCTGAAGCTGGCACCGTTGAAGATCTGGAA---
CTGGAAGACGTTCTGAAAACCGGTTACGGCGACATCCGTTGCGTAGAGTCTGGCGGTCCTGAGCCCGGTGTTGGT
TGTGCCGGCCGTGGTGTTATCACC---GCTATCAACTTCCTCGAAGAGGAAGGCGCCTAC---GAAGACGATCTC---
GAC---------TTCGTATTCTACGACGTACTCGGCGACGTTGTTTGCGGTGGCTTCGCTATGCCTATCCGTGAAAAC---
AAAGCTCAGGAAATTTACATCGT-------

>KF151528.1_Uncultured_marine_9

TCTACCCGTTTGATCCTTCACTCCAAAGCTCAGAACACCATTATGGAA------ATG---
GCTGCTGAAGCTGGCACCGTTGAAGATCTGGAA---
CTGGAAGACGTTCTGAAAACCGGATACGGCGACATCCGTTGCGTAGAGTCTGGCGGTCCTGAGCCCGGTGTTGGT
TGTGCCGGCCGTGGTGTTATCACC---GCTATCAACTTCCTCGAAGAGGAAGGCGCCTAC---GAAGACGATCTC---
GAC---------TTCGTTTTCTACGACGTACTCGGTGACGTTGTTTGCGGTGGCTTCGCTATGCCTATCCGTGAAAAC---
AAAGCTCAGGATATCTACATCGT-------

>EU331546.1_Uncultured_soil_4

TCTACCCGTTTGATCCTGCATGCAAAAGCGCAAAACTCGATCATGCAA------ATG---
GCTGCCGACGCCGGCAGCGTCGAAGATCTGGAA---
CTGGAAGACGTATTGAAAGTCGGTTACGGCGACATTAAATGCGTTGAATCCGGCGGTCCGGAACCCGGCGTCGGC
TGCGCGGGACGCGGCGTCATCACG---GCGATCAACTTCCTGGAAGAAGAAGGCGCTTAC---GACGAAGATCTG---
GAT---------TTCGTCTTCTACGACGTGCTCGGTGACGTGGTCTGCGGCGGATTCGCGATGCCGATCCGCGAAAAC---
AAGGCGCAGGAAATTTATATCGTCTGCTC-

>JX268263.1_Uncultured_soil_4

TCTACCCGTTTGATTCTGCATGCGAAAGCGCAAAACTCGATCATGCAA------ATG---
GCGGCCGACGCGGGCAGCGTCGAGGATCTGGAG---
CTGGAAGACGTACTGAAGGTCGGCTACGGCGACATCAAATGCGTCGAATCCGGCGGCCCTGAACCCGGCGTCGGC
TGCGCCGGCCGCGGCGTGATCACT---GCGATCAACTTCCTGGAAGAAGAAGGCGCCTGC---GACGAGGATTTG---
GAT---------TTCGTATTCTACGACGTACTCGGCGACGTCGTCTGCGGCGGTTTCGCGATGCCGATCCGCGAAAAC---
AAGGCGCAGGAAATTTATATCGTCTGCTC-

>AY644349.1_Azomonas

TCGACTCGCCTGATTCTGCACTCCAAAGCCCAGAACACCATCATGGAA------ATG---
GCTGCTGAAGCCGGTACCGTTGAAGATCTGGAG---
CTGGAAGACGTTCTGAAAGTAGGTTTCGGTGGCGTCAAGTGTGTCGAGTCCGGTGGTCCTGAGCCGGGTGTAGGT
TGCGCTGGCCGTGGTGTTATCACT---GCAATCAACTTCCTCGAAGAGGAAGGTGCTTAT---GAAGAAGATCTG---
GAC---------TTCGTATTCTACGACGTACTGGGTGACGTAGTGTGTGGCGGCTTCGCCATGCCGATTCGTGAAAAC---
AAGGCTCAGGAAATCTACATCGTCTGCTC-

>EU672874.1_Methylogaea_soil

------------------------------CAGAACTCCATCATGCAA------ATG---
GCCGCCGACGCGGGCAGCGTGGAGGACCTGGAG---
CTGGAAGACGTGCTCAAGGTCGGCTACGGCAACGTCAAATGCGTGGAATCCGGCGGCCCTGAGCCGGGCGTCGG
CTGCGCCGGCCGCGGCGTGATCACC---GCCATCAACTTCCTGGAAGAAGAAGGCGCCTAC---GAGGAAGACCTG---
GAC---------TTCGTGTTCTACGACGTACTGGGCGACGTGGTGTGCGGCGGTTTCGCCATGCCCATCCGCGAAAAC---
AAGGCCCAGGAAATCTACATCGTCTGCTC-

>M32371.1_A.vinelandii

TCCACCCGCCTGATCCTGCACTCCAAGGCCCAGGGCACCGTCATGGAA------ATG---
GCCGCGTCCGCCGGCTCGGTCGAAGACCTGGAG---
CTGGAAGACGTGCTGCAGATCGGCTTCGGCGGCGTCAAGTGCGTCGAATCCGGTGGCCCGGAGCCGGGCGTCGC
TTGCGCCGGCCGTGGCGTGATCACC---GCGATCAACTTCCTGGAAGAAGAAGGCGCCTAC---AGCGACGACCTG---
GAC---------TTCGTGTTCTATGACGTGCTGGGCGACGTGGTATGCGGCGGCTTCGCCATGCCGATCCGCGAGAAC---
AAGGCCCAGGAAATCTACATCGTCTGCTC-

>X03916.1_Azotobacter_14

TCCACCCGCCTGATCCTGCATTCCAAGGCCCAGAACACCGTCATGGAG------ATG---GCCGCATCCGCCGGCTCGGTGGAAGACCTCGAG---CTGGAAGACGTGCTGCAGATCGGCTACGGCGGCGTCAAGTGCGTCGAGTCCGGCGGTCCTGAGCCGGGCGTCGGCTGCGCCGGCCGCGGCGTGATCACC---GCGATCAACTTCCTGGAAGAGGAAGGCGCCTAC---AGCGACGACCTG---GAC---------TTCGTGTTCTACGACGTGCTGGGCGACGTGGTGTGCGGTGGCTTCGCCATGCCGATCCGCGAAAAC---AAGGCTCAGGAAATCTACATCGTTTGCTC-

>AY196418.1_Uncultured_soil_4

TCCACTCGCCTGATCCTGCACTCCAAGGCCCAGAACACCGTCATGGAG------ATG---GCCGCATCCGCCGGCTCGGTGGAAGACCTGGAG---CTGGAAGACGTGCTGCAGATCGGCTTCGGCGGCGTCAAGTGCGTCGAGTCCGGCGGCCCTGAGCCGGGCGTCGGCTGCGCCGGTCGCGGCGTGATCACC---GCGATCAACTTCCTCGAAGAGGAAGGCGCCTAC---AGCGACGACCTG---GAC---------TTCGTGTTCTACGACGTGCTGGGCGACGTGGTGTGCGGCGGCTTCGCCATGCCGATCCGCGAGAAC---AAGGCCCAGGAAATCTACATCGTCTGCTC-

>KF861371.1_Uncultured_soil_2

TCCACCCGCCTGATCCTGCACTCCAAGGCCCAGAGCACCGTCATGGAG------ATG---GCCGCATCCGCCGGTTCGGTCGAGGACCTGGAG---CTGGAGGACGTGCTGCAGATCGGCTTCGGCGGCGTCAAGTGCGTGGAGTCCGGCGGCCCCGAGCCGGGCGTCGGCTGCGCCGGCCGCGGCGTGATCACC---GCGATCAACTTCCTGGAAGAGGAAGGCGCCTACAAC------GACCTG---GAC---------TTCGTGTTCTACGACGTGCTGGGCGACGTGGTGTGCGGCGGCTTCGCCATGCCGATCCGCGAGAAC---AAGGCCCAGGAAATCTACATCGTCTGCTC-

>AF216883.1_Azomonas_2

TCCACTCGTCTGATCCTGCACTCCAAAGCCCAGAACACCATCATGGAA------ATG---GCTGCTGAAGCCGGTACCGTTGAAGATCTGGAG---CTGGAAGACGTTCTGAAAGTCGGCTTCGGTGGCGTTAAGTGCGTTGAGTCCGGTGGTCCAGAGCCAGGCGTTGGTTGCGCTGGCCGTGGTGTAATCACC---GCCATCAACTTCTTAGAAGAAGAAGGCGCGTAC---GAAGACGATCTG---GAC---------TTCGTATTCTACGACGTACTGGGTGACGTAGTTTGCGGTGGCTTCGCTATGCCCATCCGTGAAAAC---AAAGCTCAAGAAATCTACATCGTTTGCTC-

>KJ021871.1_Marinobacterium_root

TCTACCCGTCTGATCCTGCACTCCAAGGCCCAGAACACCATCATGGAA------ATG---GCTGCTGAAGCCGGCACCGTGGAAGACCTGGAA---CTTGAAGATGTAATGAAAACTGGCTACGGCGAAGTTAAGTGCGTCGAGTCTGGTGGTCSTGAGCCGGGTGTTGGTTGCGCCGGCCGCGGTGTAATCACC---GCTATCAACTTCCTCGAAGAGGAAGGCGCCTAC---GAAGACGATCTC---GAC---------TTCGTATTCTACGACGTACTGGGTGACGTTGTATGCGGTGGCTTCGCTATGCCGATCCGCGAAAAC---AAAGCTCAGGAAATCTACATCGTATGCTC-

>EU035273.1_Paracoccus_5

TCCACCCGTCTGATCCTGCACTCCAAGGCCCAGAACACCATCATGGAA------ATG---GCTGCTGAAGCCGGCACCGTGGAAGACCTGGAA---CTTGAAGATGTAATGAAGAATGGCTACGGCGACGTTAAGTGCGTCGAGTCCGGTGGTCCTGAGCCGGGTGTTGGTTGCGCCGGCCGCGGTGTAATTACT---GCCATTAACTTCCTTGAAGAGGAAGGTGCCTAC---GAAGACGATCTC---GAC---------TTCGTATTCTACGACGTACTCGGTGACGTTGTATGCGGTGGCTTTGCTATGCCGATCCGCGAAAAC---AAGGCCCAGGAAATCTACATTGTT------

>DQ176978.1_Uncultured_marine root_5

TCTACCCGTCTGATCCTGCACTCCAAGGCCCAGAACACCATCATGGAA------ATG---GCTGCTGAAGCCGGCACCGTAGAAGATCTGGAA---CTGGAAGATGTAATGAAGAACGGCTACGGCGACGTTAAGTGCGTCGAGTCCGGTGGTCCGGAGCCGGGTGTTGGTTGCGCCGGCCGCGGCGTAATCACG---GCTATCAACTTCCTCGAAGAGGAAGGCGCCTAC---GAAGACGATCTC---GAC---------TTCGTATTCTACGACGTACTCGGTGACGTTGTATGCGGTGGCTTCGCTATGCCGATCCGCGAAAAC---AAGGCCCAGGAAATCTACATCGTT------

>HQ455885.1_Uncultured_South China Sea_13

TCTACCCGCCTGATCCTGCACTCCAAGGCCCAGAACACCATCATGGAA------ATG---GCTGCCGAAGCCGGCACCGTTGAAGATCTGGAA---CTGGAAGATGTAATGAAGACCGGCTACGGCGACGTCAAGTGTGTCGAGTCTGGTGGTCCTGAGCCGGGTGTTGGTTGTGCCGGCCGCGGTGTTATCACC---GCCATCAACTTCCTCGAAGAGGAAGGTGCCTAC---GAAGACGATCTG---GAC---------TTCGTGTTCTACGGCGTACTGGGTGACGTTGTATGCGGTGGCTTCGCGATGCCGATCCGCGAAAAC---AAGGCTCAGGAAATCTACATCGT-------

>KF861200.1_Uncultured_soil_2

TCCACCCGCCTGATCCTGCACTCCAAGGCCCAGAACACCATCATGGAA------ATG---GCTGCCGAAGCGGGCACCGTTGAAGATCTGGAA---CTGGAAGATGTAATGAAGACCGGCTACGGCGACGTCAAGTGCGTCGAGTCCGGTGGTCCGGAGCCGGGTGTTGGCTGTGCCGGTCGCGGTGTTATCACT---GCGATCAACTTCCTCGAAGAGGAAGGCGCCTAC---GAAGACGATCTG---GAC---------TTCGTGTTCTACGACGTACTGGGTGACGTTGTGTGTGGCGGCTTCGCCATGCCGATCCGTGAAAAC---AAGGCTCAGGAAATCTACATTGT-------

>AF059621.1_Unidentified_marine_5

TCTACTCGTCTGATCCTGCACTCCAAGGCCCAGAACACCATCATGGAA------ATG---GCTGCCGAAGCCGGTACCGTTGAAGATCTGGAA---CTGGAAGATGTACTGAAGACCGGTTACGGCGACGTTAAGTGCGTTGAGTCCGGTGGTCCGGAGCCGGGTGTTGGTTGCGCCGGTCGTGGTGTTATCACC---GCTATCAACTTCATGGAAGAGGAAGGTGCCTAC---GAAGACGATCTG---GAC---------TTCGTCTTCTACGACGTACTGGGTGACGTTGTATGCGGTGGCTTCGCGATGCCTATCCGTGAAAAC---AAAGCTCAGGAAATCTACATTGTTTGCTCT

>JN097352.1_Uncultured_South China Sea_27

TCTACTCGTCTGATCCTGCACTCCAAAGCTCAGAACACCATCATGGAA------ATG---
GCTGCCGAAGCCGGTACCGTTGAAGATCTGGAA---
CTGGAAGATGTGCTGAAAGCCGGTTACGGCGGCATCAAGTGCGTTGAGTCCGGTGGTCCGGAGCCTGGCGTTGGT
TGCGCTGGTCGTGGTGTTATCACC---GCTATCAACTTCCTGGAAGAGGAAGGCGCGTAC---GAAGACGATCTG---
GAC---------TTCGTATTCTACGACGTACTGGGTGACGTTGTATGCGGTGGCTTCGCTATGCCGATCCGCGAAAAC---
AAGGCTCAGGAAATCTACATCGTTTGCTCT

>AY684103.1_Uncultured_soil_21

TCCACTCGCCTGATCCTGCACTCCAAGGCCCAGAACACCATCATGGAA------ATG---
GCTGCCGAGGCCGGCACCGTTGAAGATCTCGAG---
CTGGAAGACGTGCTCAAGGTCGGCTACGGCGGCGTCAAGTGCGTCGAGTCGGGTGGCCCGGAGCCAGGTGTTGG
CTGCGCCGGCCGTGGCGTGATCACC---GCGATCAACTTCCTCGAAGAGGAAGGCGCCTAT---GACGAAGACCTG---
GAC---------TTCGTGTTCTACGACGTGCTCGGCGACGTGGTGTGCGGCGGCTTCGCCATGCCCATCCGCGAGAAC---
AAGGCCCAGGAAATCTACATCGTTTGCTC-

>EF178501.1_Uncultured_marine_8

TCCACTCGTCTGATCCTGCACTCCAAAGCCCAGAACACCATCACGGAA------ATG---
GCTGCCGAAGCCGGTACCGTTGAAGATCTGGAA---
CTGGAAGACGTACTGAAAGTCGGTTACGGCGGCGTCAAGTGCACCGAGTCTGGTGGTCCTGAGCCGGGCGTTGGC
TGTGCTGGCCGTGGTGTTATCACC---GCCATCAACTTCCTGGAAGAAGAAGGCGCCTAC---GAAGACGATCTG---
GAC---------TTCGTATTCTACGACGTACTGGGTGACGTTGTTTGCGGTGGCTTCGCCATGCCGATCCGCGAAAAC---
AAGGCTCAGGAAATCTACATCGTTTGCTC-

>EF634054.1_Azotobacter_soil

--------------------------------AAACACCATTATGGGAAA----ATG---
GCTGCCGAGGCCGGCACCGTCGAAAACCTCGAG---
CTGGAAGACGTGCTCAAGGTCGGCTTCGGTGGTGTCAAGTGCGTTGAGTCCGGTGGTCCGGAGCCGGGCGTCGG
CTGCGCCGGCCGTGGCGTGATCACC---GCGATCAACTTCCTGGAAGAGGAAGGCGCCTAT---GACGAGGACCTG---
GAC---------TTCGTGTTCTACGACGTACTGGGCGACGTGGTGTGCGGTGGCTTCGCCATGCCCATCCGCGAGAAC---
AAGGCTCAGGAAATCTACATCGTCTGCTC-

>JQ023583.1_Uncultured_coral_22

TCCACTCGCCTGATTCTGCACTCCAAGGCTCAGAACACCATCATGGAA------ATG---
GCTGCCGAGGCCGGCACCGTCGAAGACCTCGAG---
CTGGAAGACGTGCTCAAGGTCGGCTTCCGCGGCGTCAAGTGCGTTGAGTCCGGTGGTCCGGAGCCGGGCGTGGG
CTGCGCCGGCCGTGGCGTGATCACC---GCGATCAACTTCCTGGAAGAGGAAGGCGCCTAC---GACGAGGACCTG---
GAC---------TTCGTGTTCTACGACGTACTGGGCGACGTGGTGTGCGGTGGCTTCGCCATGCCCATCCGCGAAAAC---
AAGGCTCAGGAAATCTACATCGTCTGCTC-

>CP005095.1_Azotobacter_20

TCCACCCGCCTGATCCTGCACTCCAAGGCCCAGAACACCATCATGGAA------ATG---
GCTGCCGAAGCCGGTACCGTGGAAGATCTGGAG---
CTGGAAGACGTGCTGAAGGCTGGCTACGGCGGCGTCAAGTGCGTTGANTCCGGTGGTCCGGAGCCGGGCGTTGG
CTGCGCCGGCCGTGGTGTTATCACC---GCCATCAACTTCCTGGAAGAGGAAGGCGCCTAC---GAAGACGATCTG---
GAC---------TTCGTATTCTNCGACGTGCTGGGCGACGTGGTGTGTGGCGGCTTCGCCATGCCGATCCGCGAGAAC---
AAGGCCCAGGAAATCTACATCGTTCTGCTC-

>M11579.1_Azotobacter

TCCACCCGCCTGATCCTGCACTCCAAGGCCCAGAACACCATCATGGAA------ATG---
GCTGCCGAAGCCGGTACCGTGGAAGATCTGGAG---
CTGGAAGACGTGCTGAAGGCTGGCTACGGCGGCGTCAAGTGCGTTGAGTCCGGTGGTCCGGAGCCGGGCGTTGG
CTGCGCCGGCCGTGGTGTTATCACA---GCAATCAACTTCCTGGAAGAGGAAGGCGCCTAC---GAAGACGATCTG---
GAC---------TTCGTATTCTACGACGTCCTGGGCGACGTGGTGTGTGGCGGCTTCGCCATGCCGATCCGCGAGAAC---
AAGCCCCAAGAAATCTACATCGTCTGCTC-

>AY196413.1_Uncultured_soil_2

TCCACTCGCCTGATCCTGCACTCCAAGGCCCAGAACACCATCATGGAA------ATG---
GCTGCCGAAGCCGGCACCGTGGAAGATCTGGAG---
CTGGAAGACGTGCTGAAGGTCGGCTACGGCGGCGTCAAGTGCGTTGAGTCCGGTGGTCCGGAGCCGGGCGTTGG
TTGCGCTGGCCGTGGTGTTATCACC---GCGATCAACTTCCTGGAAGAGGAAGGCGCCTAC---AGCGACGACCTG---
GAC---------TTCGTGTTCTACGACGTGCTGGGCGACGTGGTGTGCGGCGGCTTCGCCATGCCGATCCGCGAGAAC---
AAGGCCCAGGAGATCTACATCGTCTGCTC-

>M73020.1_A.chroococcum_74

TCCACTCGCCTGATCCTGCACTCCAAGGCCCAGAACACCATCATGGAA------
ATGTHTGCTGCCGAAGCCGGCACCGTGGAAGATCTGGAG---
CTGGAAGACGTGCTGAAGGTCGGCTACGGCGGCGTCAAGTGCGTTGAGTCCGGTGGTCCGGAGCCGGGCGTTGG
CTGCGCTGGCCGTGGTGTTATCACC---GCGATCAACTTCCTGGAAGAGGAAGGCGCCTAC---GAAGACGATCTG---
GAC---------TTCGTATTCTACGACGTACTGGGCGACGTGGTGTGCGGTGGCTTCGCCATGCCGATCCGCGAAAAC---
AAGGCTCAGGAAATCTACATCGTTTGCTC-

>KF881107.1_Azotobacter_soil_2

---------------CTGCACTCCAAGGCCCAGAACACCATCATGGAA------ATG---
GCCGCCGAAGCCGGCACCGTGGAAGATCTGGAG---
CTGGAAGACGTGCTGAAGGTCGGCTACGGCGGCGTCAAGTGCGTTGAGTCCGGTGGTCCGGAGCCGGGTGTTGG
TTGCGCTGGCCGTGGTGTGATCACC---GCGATCAACTTCCTGGAAGAGGAAGGCGCCTAC---GAAGACGACCTG---
GAC---------TTCGTATTCTACGACGTGCTGGGTGACGTGGTGTGCGGTGGCTTCGCCATGCCGATCCGCGAGAAC---
AAGGCCTAGGAAATCTACATCGTTT-----
