# Supplementary material for: Widespread Distribution and Expression of Gamma A (UMB), an Uncultured, Diazotrophic, γ-Proteobacterial nifH Phylotype
Source: PLoS One. 2015 Jun 23;10(6):e0128912. doi: 10.1371/journal.pone.0128912 (PMC4477881; doi:10.1371/journal.pone.0128912)
Supplement: S2 Alignment — (PDF) [file pone.0128912.s002.pdf]

>AY896371.1_GammaA/1-327

TCCACACGTCTTATTCTGCACTCAAAAGCGCAAAACACA---
ATAATGGAGATGGCTGCACAGGCTGGCACGGTAGAGGATCTTGAGCTTGAAGAAGTGCTTAAGGTTGGCTTTGGC
---GACATTAAGTGC------
GTGGAGTCAGGTGGTCCTGAGCCTGGAGTTGGCTGTGCCGGTCGTGGTGTTATTACCGCGATTAACTTTCTTGAAG
AGGAAGGTGCATA---CTCAG------ACGACTTAGATTTCGTTTTTTATGATGTTCTAGG----------
TGATGTGGTTTGTGGCGGATTTGCAATGCCTATTCGTGAGAATAAGGCTCAGGAAATCTACATTGTTTGCTCT

>HM210397.1_Gamma3/1-312

TCCACGCGTCTTATCTTGCACTCCAAAGCCCAAAACACA---
ATCATGGAAATGGCTGCCCAAGCAGGTACGGTTGAGGATTTAGAGCTTGATGACGTACTTAAGGTTGGCTACGGC-
--GATATTAAGTGT------
GTAGAAGCAGGCGGTCCAGAGCCCGGCGTTGGTTGTGCCGGTCGCGGTGTTATTACCGCGATTAACTTCCTTGAG
GAAGAAGGTGCATA---TGAAG------ACGATCTAGACTTTGTTTTCTATGATGTTCTGGG----------
CGACGTGGTCTGCGGCGGCTTTGCTATGCCAATTCGTGAAAACAAAGCTCAGGAGATC---------------

>DQ913881.1_Celerinatantimonas/1-320

TCTACTCGCCTTATGCTTCACGCGAAAGCTCAGAACACC---
ATTATGGAAATGGCTGCTGAAGCAGGTTCGGTTGAAGATCTTGAACTAGAGGATGTGCTAAAAGTTGGCTACGGC-
--GGGGTGAAATGC------
GTTGAATCAGGTGGCCCAGAGCCGGGCGTTGGTTGTGCTGGTCGTGGGGTTATCACAGCGATTAACTTCCTCGAA
GAAGAAGGCGCTTA---CGATG------ATGACTTAGATTTTGTTTTTTATGACGTACTTGG----------
TGACGTTGTATGCGGCGGGTTCGCAATGCCTATTCGCGAAAACAAAGCGCAGGAAATCTACATCGT-------

>JF701923.1_Alteromonadales/1-308

------------ATGCTGCATGCCAAAGCGCAAAACACC---
ATTATGGAAATGGCCGCTGAACAGGGGTCGGTTGAAGATCTTGAATTAGAAGATGTGCTCAAAGTCGGTTACGGC-
--GGGGTGAAATGC------
GTTGAATCAGGTGGCCCTGAGCCGGGCGTTGGTTGTGCGGGTCGTGGGGTTATCACCGCAATTAACTTCCTTGAAG
AAGAAGGCGCTTA---TGACG------AAGACCTCGATTTTGTTTTCTATGACGTGCTTGG----------
GGATGTGGTTTGCGGTGGATTTGCCATGCCAATTCGTGAAAACAAAGCGCAAGAAATTTACATCGT-------

>AY937260.1_Methylobacter/1-326

TCTACCCGTTTAATTCTTCATGCAAAAGCTCAAACTACT---
ATTATGAGCTTGGCTGCTGAAGCGGGTAGTGTTGAAGATTTAGAGCTTGAAGATGTACTGAAAGCGGGTTACCGC-
--GGTATTAAATGC------
GTTGAATCAGGTGGTCCTGAGCCTGGTGTCGGTTGTGCGGGTCGCGGCGTTATCACTGCGATTAACTTCCTTGAAG
AAGAAGGTGCTTA---TGATG------ATGAACTTGACTTCGTTTTTTATGATGTACTTGG----------
GGATGTTGTGTGTGGTGGCTTTGCCATGCCAATCCGTGAAAACAAGGCGCAAGAAATTTACATCGTTTGCTC-

>AJ563957.1_Methylobacter/1-326

TCTACTCGTTTGATCCTGCACTCAAAAGCACAAACGACC---
ATCATGAGCCTGGCGGCTGAAGCCGGCAGCGTTGAAGATCTTGAACTGGAAGATGTGTTGAAAGTCGGCTTCGGC
---GGCGTTAAATGC------
GTTGAATCTGGCGGCCCTGAGCCCGGCGTCGGCTGTGCCGGCCGCGGTGTAATCACGGCCATCAACTTCCTCGAG
GAAGAAGGAGCTTA---CGATG------ATGAACTCGATTTCGTTTTTTATGATGTTCTTGG----------
GGATGTTGTGTGTGGCGGCTTTGCCATGCCAATCCGTGAAAACAAGGCGCAGGAAATTTACATCGTTTGCTC-

>AB524080.1_Methylovulum/1-302

------------------------AAAGCACAAACCACC---
ATCATGAGCTTGGCTGCGGAAGCAGGCAGCGTGGAAGATCTGGAATTGGAAGACGTGTTGAAAGTCGGTTACGG
C---GGCATCAAATGC------
GTTGAATCAGGCGGCCCTGAGCCAGGCGTCGGCTGTGCAGGTCGTGGTGTTATCACCGCGATTAACTTCCTCGAAG
AAGAAGGGGCTTA---TGATG------ATGAACTTGACTTCGTTTTTTATGATGTACTTGG----------
GGATGTTGTGTGTGGTGGTTTTGCCATGCCAATCCGTGAGAACAAGGCGCAAGAAATTTACATCGTTTGCTC-

>DQ665842.1_Methylosoma/1-270

GCTGCGGAAGCAGGCAGCGTGGAAGATTTGGAATTGGAAGACGTGTTGAAAGTCGGTTACGGC---
GGCATCAAATGC------
GTTGAATCCGGCGGCCCAGAGCCAGGCGTTGGCTGTGCAGGTCGTGGTGTTATCACGGCGATTAACTTCCTTGAA
GAAGAAGGGGCTTA---CGACG------ATGAATTGGATTTCGTTTTTTATGATGTTCTTGG----------
GGATGTTGTGTGTGGTGGTTTCGCCATGCCAATCCGTGAAAACAAGGCGCAAGAAATTTACATCGTT------

>KC256777.1_Pseudanabaena/1-320

TCCACTCGACTCATTCTTCACGCGAAGGCGCAAAACACC---
ATTATGGAAATGGCAGCTGAAGCCGGTTCGGTTGAAGACTTAGAATTAGAAGATGTATTACGTGTCGGTTATGCC--
-GGTATTCGCTGT------
GTTGAGTCTGGTGGTCCAGAACCAGGTGTTGGTTGTGCGGGTCGTGGTGTTATTACGGCAATTAACTTTTTAGAAG
AAGAAGGCGCGTA---CGGAG------AAGATTTAGACTTCGTATTTTACGACGTCCTCGG----------
TGATGTAGTGTGTGGTGGTTTTGCGATGCCAATTCGCGAAAACAAAGCACAAGAAATCTACATCGT-------

>AF134809.2_Vibrio/1-320

TCAACTCGCCTTATCCTCCACACTAAGATGCAAAATACC---
ATCATGGAAATGGCAGCAGAAGCTGGCACAGTTGAAGACATCGAACTAGAAGATGTGCTGCTGACTGGGTACGG
G---GATGTTAAATGT------
GTTGAATCAGGCGGCCCAGAGCCAGGTGTTGGTTGTGCTGGCCGTGGTGTCATCACAGCGATCAACTTCCTAGAA
GAAGAAGGGGCGTA---CGAAG------AAGATATAGATTTCGTATTCTACGATGTTCTTGG----------
TGATGTGGTATGTGGTGGTTTCGCCATGCCCATCCGTGAAAACAAAGCACAAGAAATCTATATCGT-------

>EF203422.1_Vibrio/1-321

TCAACTCGTCTTATTCTACACACGAAGATGCAAAACACC---
ATCATGGAAATGGCAGCGGAAGCCGGTACTGTGGAAGACATCGAACTAGAAGATGTACTACTCACTGGTTACGGT-
--GACGTTAAATGT------
GTTGAGTCAGGCGGTCCTGAGCCAGGCGTTGGTTGTGCGGGTCGTGGTGTAATCACAGCGATCAACTTCCTGGAA
GAAGAAGGTGCGTA---CGACG------AAGATCTAGATTTCGTATTTATCGATGTTCTTGG----------
TGACGTTGTGTGTGGTGGTTTCGCGATGCCAATTCGTGAAAACAAAGCACAAGAAATCTACATCGTT------

>AF082989.1_Vibrio/1-309

------------ATTCTACACTCCAAAGCACAGAATACC---
ATCATGGAGATGGCGGCTGAAGCGGGGACGGTTGAAGACATCGAACTAGAAGATGTACTTAAAATCGGTTACGG
C---GATGTTCGTTGT------
GTTGAGTCAGGCGGTCCAGAGCCAGGTGTTGGCTGTGCGGGTCGCGGTGTTATCACGGCCATCAACTTTTTGGAA
GAAGAAGGAGCCTA---CGAGG------ACGACTTGGACTTCGTATTCTACGACGTACTGGG----------
TGACGTTGTATGTGGTGGTTTTGCGATGCCAATTCGTGAAAACAAAGCAGAAGAAATCTACATCGTT------

>AF111110.2_Vibrio/1-327

TCAACTCGTCTCATCCTGCACTCAAAAGCACAAAACACC---
ATCATGGAAATGGCAGCGGAAGCCGGTACGGTTGAAGACATCGAACTAGAAGATGTATTGAAAGTCGGTTATGGC
---GATGTTCGCTGT------
GTGGAATCAGGCGGCCCTGAGCCAGGCGTAGGTTGTGCTGGTCGCGGTGTTATCACAGCAATCAACTTCCTCGAA
GAAGAAGGCGCGTA---TGAAG------ATGACTTAGATTTCGTTTTCTACGACGTATTGGG----------
TGACGTTGTGTGTGGTGGTTTCGCGATGCCAATTCGTGAAAACAAAGCGCAAGAAATCTACATCGTATGTTCT

>EF554362.1_Vibrio/1-255

TCTACTCGTTTGATTCTGCACTCTAAAGCGCAAAACACC---
ATCATGGAAATGGCCGCCCAAGCCGGTACGGTTGAAGACATCGAATTAGAAGATGTATTGAAAGTCGGTTTTGGC-
--GATGTTCGCTGC------
GTTGAATCAGGCGGTCCAGAGCCAGGTGTTGGTTGTGCTGGTCGTGGTGTTATCACCGCTATCAACTTCCTCGAAG
AAGAAGGTGCCTA---CGAAG------ACGATTTGGACTTCGTATTCTATGACGTACTGGG----------T-----------------------
----

>DQ364801.1_Klebsiella/1-305

TCGACCCGTCTGATCCTTCACGCTAAAGCCCAGAACACC---
ATCATGGAGATGGCGGCGGAAGTGGGCTCGGTCGAGGATCTGGAGCTCGAAGACGTTCTGCAAATCGGCTATGG
C---GATGTCCGTTGC------
GCCGAATCCGGCGGCCCGGAGCCAGGCGTCGGCTGCGCCGGACGCGGGGTGATCACCGCCATCAACTTCCTCGAG
GAAGAAGGCGCCTA---TGAAG------AAGATTTGGATTTCGTCTTCTATGACGTCCTCGG----------
CGACGTGGTCTGCGGCGGCTTCGCCATGCCGATCCGCGAAAACAAGGCTCA---------------------

>EF199958.1_Thioalkalispira/1-314

TCCACCCGTCTGATCCTGCACTCCAAGGCCCAGACCACG---
GTTATGCATCTGGCCGCCGAGGCCGGTTCGGTCGAAGACCTGGAGCTGGAAGATGTACTGTGCGTGGGCTACGGC
---GATGTGAAGTGC------
GTCGAATCCGGTGGTCCTGAGCCGGGTGTAGGTTGTGCCGGTCGTGGCGTAATCACCGCAATCAACTTCCTGGAA
GAGGAAGGCGCCTA---CGACG------AAGACCTGGACTTCGTATTCTATGACGTACTGGG----------
AGTCGTAGTGTGTGGTGGCTTCGCCATGCCGATCCGCGAAAACAAGGCCCAGGAGATCTA-------------

>HM210377.1_Gamma1/1-320

TCCACGCGTTTGATCCTGCACTCCAAAGCTCAAACCACT---
GTGATGCATCTGGCTGCCGAAGCCGGCACCGTGGAAGATCTGGAGCTGGAAGATGTGCTGTCTGTCGGTTACGGC
---GATGTAAAATGC------
GTCGAGTCCGGTGGTCCCGAGCCGGGTGTCGGCTGCGCCGGTCGCGGTGTTATCACCGCCATCAACTTCCTGGAA
GAGGAAGGCGCTTA---CGACG------AAGATCTGGACTTCGTATTCTACGATGTCCTGGG----------
CGACGTGATCTGCGGTGGCTTTGCTATGCCCATCCGTGAAAACAAAGCGCAAGAAATCTACATCGT-------

>AY896428.1_GammaP/1-327

TCTACTCGTCTGATCCTTCACTCTAAAGCTCAAACTACT---
GTTATGCACTTGGCTGCAGAAGCAGGCACGGTAGAAGACCTGGAGCTGGAAGATGTACTGTCTGTTGGCTACGGC
---GACGTTAAATGC------
GTTGAGTCTGGTGGCCCTGAGCCAGGTGTTGGTTGTGCAGGTCGTGGTGTAATCACTGCAATCAACTTCCTGGAAG
AAGAAGGTGCCTA---TGACG------AAGACCTAGACTTCGTATTCTACGACGTATTGGG----------
TGACGTTGTATGTGGTGGTTTCGCGATGCCTATTCGTGAAAACAAAGCACAAGAAATCTACATCGTATGTTCT

>HE801219.1_Methylomonas/1-326

TCCACTCGTTTGATCTTACATGCTAAAGCACAAAACTCT---
ATCATGCAAATGGCGGCAGACGCCGGCAGCGTGGAAGATTTGGAACTGGAAGATGTATTGAAAGTTGGATACGG
C---GATGTGAAATGC------
GTTGAATCCGGCGGTCCAGAGCCAGGAGTTGGTTGCGCAGGCCGTGGTGTTATCACCGCTATCAACTTCCTGGAA
GAGGAAGGCGCTTA---CACCG------ATGACCTGGACTTCGTGTTTTATGATGTATTGGG----------
TGACGTTGTATGCGGTGGTTTCGCGATGCCTATCCGCGAAAACAAAGCTCAAGAAATTTACATCGTTTGCTC-

>HF954371.1_Methylomonas/1-270

TCTACTCGTTTGATTCTGCATGCGAAAGCGCAAAACTCC---
ATCATGCAAATGGCAGCCGACGCCGGCAGCGTAGAAGACTTGGAACTGGAAGACGTATTGAAAGTGGGTTACCGC
---GACATTAAATGC------
GTTGAGTCAGGCGGTCCAGAGCCAGGCGTAGGTTGCGCCGGCCGTGGTGTTATCACCGCGATCAACTTCTTGGAA
GAAGAAGGTGCTTA---CACCG------ACGACCTCGACTTCGTATTCTACGATGTATTGGG----------
TGACGTTGTGTGCGGC---

```
>CP002738.1_Methylomonas/1-326

TCCACACGTTTAATTCTACACGCAAAAGCGCAAAACTCC---
ATCATGCAAATGGCGGCCGATGCAGGTAGCGTTGAAGATTTGGAATTGGAAGACGTATTGAAAGTGGGTTACCGC
---GACATTAAATGC------
GTTGAGTCCGGCGGCCCAGAGCCAGGCGTTGGTTGTGCCGGCCGCGGTGTTATCACTGCCATCAACTTCCTGGAA
GAAGAAGGCGCTTA---CGACG------AAAACCTGGACTTCGTGTTCTACGACGTATTGGG----------
TGACGTTGTGTGCGGCGGTTTCGCGATGCCGATTCGCGAAAACAAAGCGCAAGAAATTTATATCGTTTGCTC-

>HF954372.1_Methylomonas/1-254

------------ATCCTGCACTCTAAAGCGCAAACCACC---
ATCATGCACTTGGCTGCTGAAGCCGGTAGCGTTGAGGATTTGGAATTGGAAGACGTGTTGAAAGTCGGTTACGGC-
--GACGTTAAATGC------
GTTGAATCAGGCGGTCCTGAGCCAGGAGTCGGTTGCGCCGGCCGTGGCGTTATCACCGCGATCAACTTCCTGGAA
GAAGAAGGCGCTTA---CGACG------AAGACCTCGACTTCGTGTTCTACGACGTATTGGG----------CGACGTTGTCTG-
-------------------------------------------------------------

>M63691.1_Klebsiella/1-320

TCAACCCGGCTGATTCTTCACTCTAAAGCGCAAAACACG---
ATTATGGAAATGGCTGCTGAAGCTGGCTCTGTTGAAGATATCGAACTGGAAGATGTATTGAAAGTCGGTTACGGC-
--GACGTGCGCTGT------
GTTGAGTCTGGTGGTCCTGAGCCTGGTGTTGGCTGTGCCGGTCGCGGGGTGATTACGGCAATTAACTTCCTTGAAG
AAGAAGGTGCTTA---CGAAG------AAGATCTGGACTTTGTGTTCTATGACGTTCTTGG----------
TGACGTTGTGTGTGGTGGTTTCGCGATGCCAATTCGTGAAAACAAAGCACAGGAAATCTATATCGT-------

>CP001616.1_Tolumonas/1-326

TCTACTCGTTTGATCCTGCACGCAAAAGCCCAGAATACC---
ATCATGGAAATGGCAGCAGAAGTTGGTTCTGTTGAAGATCTGGAACTGGAAGACGTATTACAGATCGGTTACGGC-
--GGAGTTCGTTGT------
GCTGAGTCTGGTGGCCCAGAGCCAGGAGTTGGTTGTGCAGGTCGTGGTGTTATCACTGCTATCAACTTCCTGGAAG
AAGAAGGCGCGTA---CGAAG------AAGATTTAGACTTCGTATTCTACGACGTACTGGG----------
TGACGTAGTGTGTGGCGGTTTCGCTATGCCAATCCGTGAAAACAAAGCTCAGGAAATCTACATCGTTTGCTC-

>HM210363.1_Gamma4/1-320

TCCACTCGTCTGATTCTTCACTCCAAAGCACAGAACACC---
ATCATGGAAATGGCTGCTGAAGCCGGCACCGTGGAAGATCTGGAACTGGAAGATGTATTAAAAGCCGGTTACGGC
---GACATCCGCTGT------
GTTGAATCCGGTGGCCCGGAGCCAGGTGTTGGATGTGCCGGTCGCGGTGTAATCACTGCAATCAACTTCCTGGAA
GAGGAAGGTGCATA---TGAAG------ATGACCTGGACTTCGTATTCTACGATGTACTGGG----------
CGATGTTGTATGCGGTGGCTTTGCTATGCCGATCCGTGAAAACAAAGCGCAGGAAATCTACATTGT-------
```

>AF216883.1_Azomonas/1-320

TCCACTCGTCTGATCCTGCACTCCAAAGCCCAGAACACC---
ATCATGGAAATGGCTGCTGAAGCCGGTACCGTTGAAGATCTGGAGCTGGAAGACGTTCTGAAAGTCGGCTTCGGT-
--GGCGTTAAGTGC------
GTTGAATCCGGTGGTCCAGAGCCAGGCGTTGGTTGCGCTGGCCGTGGTGTTATCACCGCCATCAACTTCTTAGAAG
AAGAAGGCGCGTA---CGAAG------ACGATCTGGACTTCGTATTCTACGACGTACTGGG----------
TGACGTAGTTTGCGGTGGCTTCGCTATGCCAATCCGTGAAAACAAAGCTCAGGAAATCTACATCGT-------

>AY644349.1_Azomonas/1-326

TCGACTCGCCTGATTCTGCACTCCAAAGCCCAGAACACC---
ATCATGGAAATGGCTGCTGAAGCCGGTACCGTTGAAGATCTGGAGCTGGAAGACGTTCTGAAAGTAGGTTTCGGT-
--GGCGTCAAGTGT------
GTCGAGTCCGGTGGTCCTGAGCCGGGTGTAGGTTGCGCTGGCCGTGGTGTTATCACTGCAATCAACTTCCTCGAAG
AGGAAGGTGCTTA---TGAAG------AAGATCTGGACTTCGTATTCTACGACGTACTGGG----------
TGACGTAGTGTGTGGCGGCTTCGCCATGCCGATTCGTGAAAACAAGGCTCAGGAAATCTACATCGTCTGCTC-

>CP001614.2_Teredinibacter/1-321

TCTACCCGTCTTATCCTTCACGCCAAGGCTCAAAACACC---
ATCATGGAAATGGCTGCAGAAGCCGGTACCGTTGAAGACCTGGAACTGGAAGATGTATTGAAAGTTGGCTACGGC
---GACGTTAAGTGC------
GTTGAGTCCGGCGGTCCAGAGCCCGGTGTTGGCTGTGCTGGTCGTGGTGTAATCACTGCCATCAACTTCCTGGAAG
AAGAAGGCGCTTA---CGAAG------ACGATCTCGACTTCGTATTCTACGACGTACTGGG----------
TGACGTTGTATGTGGTGGTTTCGCTATGCCTATTCGTGAAAACAAGGCTCAGGAAATCTACATCGTT------

>KJ021871.1_Marinobacterium/1-326

TCTACCCGTCTGATCCTGCACTCCAAGGCCCAGAACACC---
ATCATGGAAATGGCTGCTGAAGCCGGCACCGTGGAAGACCTGGAACTTGAAGATGTAATGAAAACTGGCTACGGC
---GAAGTTAAGTGC------
GTCGAGTCTGGTGGTCSTGAGCCGGGTGTTGGTTGCGCCGGCCGCGGTGTAATCACCGCTATCAACTTCCTCGAAG
AGGAAGGCGCCTA---CGAAG------ACGATCTCGACTTCGTATTCTACGACGTACTGGG----------
TGACGTTGTATGCGGTGGCTTCGCTATGCCGATCCGCGAAAACAAAGCTCAGGAAATCTACATCGTATGCTC-

>HQ586273.1_Zhang/1-326

TCCACCCGCCTCATTCTGCACTCCAAAGCGCAGAATACC---
ATCATGGAAATGGCGGCTGAAGCTGGCACCGTTGAAGACCTCGAGCTCGAAGATGTGCTCAAGGTTGGCTACGGC
---GACATCAAGTGC------
GTCGAATCCGGCGGTCCAGAGCCAGGCGTCGGTTGCGCCGGTCGCGGCGTCATCACCGCCATCAACTTCCTTGAA
GAAGAAGGCGCATA---CGAAG------AAGACCTCGACTTCGTTTTCTACGACGTCCTCGG----------
CGACGTTGTTTGCGGTGGCTTCGCTATGCCGATCCGCGAAAACAAAGCTCAGGAAATCTACATCGTTTGCTC-

>AY351672.1_Azotobacter/1-320

TCCACCCGCTTGATCCTCCACGCCAAGGCACAGAACACC---
ATCATGGAGATGGCCGCTGAAGCCGGCACCGTGGAAGATCTGGAACTGGATGACGTGCTCAAAACCGGTTATGCC
---GGCATCAAGTGC------
GTTGAATCCGGTGGCCCGGAGCCAGGTGTTGGCTGCGCCGGCCGCGGCGTGATCACCGCCATCAACTTCCTCGAA
GAAGAGGGCGCCTA---CAGCG------ACGACCTCGACTTCGTGTTCTACGACGTACTGGG----------
TGACGTGGTTTGCGGTGGCTTCGCCATGCCGATCCGCGAAAACAAGGCCCAGGAGATCTACATCGT-------

>EU622788.1_Allochromatium/1-326

TCGACCCGTCTGATCCTGCACTCCAAGGTTCAGAATACC---
ATCATGGAGATGGCCGCCGAAGCCGGTTCGGTCGAAGACCTCGAACTGGAAGATGTGCTCAAGGTCGGCTACGGC
---AACATCAAGTGC------
GTCGAGTCCGGCGGTCCTGAGCCGGGTGTGGGTTGCGCCGGTCGCGGCGTCATCACCGCCATCAACTTCCTGGAA
GAAGAAGGCGCTTA---CGAGG------CCGATCTGGACTTCGTGTTCTACGACGTACTCGG----------
CGACGTGGTCTGCGGCGGATTCGCCATGCCGATCCGCGAGAACAAGGCCCAGGAGATCTACATCGTCTGCTC-

>X13519.1_A.vinelandii/1-326

TCCACCCGCCTGATCCTGCACTCCAAGGCCCAGGGCACC---
GTCATGGAAATGGCCGCGTCCGCCGGCTGGGTCGAAGACCTGGAGCTGGAAGACGTGCTGCAGATCGGCTTCGG
C---GGCGTCAAGTGC------
GTCGAATCCGGTGGCCCGGAGCCGGGCGTCGGCTGCGCCGGCCGTGGCGTGATCACCGCGATCAACTTCCTAGAA
GAAGAAGGCGCCTA---CAGCG------ACGACCTAGACTTCGTGTTCTATGACGTGCTGGG----------
CGACGTGGTATGCGGCGGCTTCGCCATGCCGATCCGCGAGAACAAGGCCCAGGAAATCTACATCGTCTGCTC-

>FR669148.1_Pseudomonas/1-326

TCCACCCGCTTGATCCTGCACTCCAAGGCGCAGAACACC---
ATCATGGAAATGGCCGCCGAGGCCGGTACCGTGGAAGACCTGGAACTTGAAGACGTGCTCAAGACCGGCTACGG
C---GACATCAAGTGC------
GTCGAGTCGGGCGGCCCTGAGCCGGGCGTGGGCTGTGCCGGTCGCGGCGTAATCACCGCGATCAACTTCCTCGAA
GAGGAAGGCGCCTA---CGAGG------ATGACCTGGATTTCGTCTTCTACGACGTGCTCGG----------
CGACGTGGTCTGTGGCGGTTTCGCCATGCCCATCCGCGAGAACAAGGCCCAGGAAATCTATGTCGTCTGCTC-

>M73020.1_A.chroococcum/1-329

TCCACTCGCCTGATCCTGCACTCCAAGGCCCAGAACACCATCATGGAAATGTHTGCTGCCGAAGCCGGCACCGTGG
AAGATCTGGAGCTGGAAGACGTGCTGAAGGTCGGCTACGGC---GGCGTCAAGTGC------
GTTGAGTCCGGTGGTCCGGAGCCGGGCGTTGGCTGCGCTGGCCGTGGTGTTATCACCGCGATCAACTTCCTGGAA
GAGGAAGGCGCCTA---CGAAG------ACGATCTGGACTTCGTATTCTACGACGTACTGGG----------
CGACGTGGTGTGCGGTGGCTTCGCCATGCCGATCCGCGAAAACAAGGCTCAGGAAATCTACATCGTTTGCTC-

>AY660961.1_Pseudomonas/1-251

ATCATGGAGATGGCCGCCGAGGCCGGCACCGTGGAAGACCTCGAGCTGGAAGACGTGCTCAAGGTCGGCTTCGG
T---GGCGTCAAGTGC------
GTCGAGTCGGGCGGCCCGGAGCCGGGCGTCGGCTGCGCCGGCCGCGGCGTGATCACCGCGATCAACTTCCTCGA
GGAGGAAGGCGCCTA---TGACG------ACGACCTCGACTTCGTGTTCTACGACGTGCTCGG----------
CGACGTGGTGTGCGGCGGCTTCGCCATGCCCATCCG-------------------------------------

>DQ821943.1_Thermochromatium/1-320

TCGACCCGTCTGATCTTGCACAGCAAGGCCCAGACCACG---
GTCATGCACCTGGCCGCCGAGGCGGGGTCGGTCGAAGACCTCGAGCTCGAGGATGTGTTGCAGGTCGGCTTCGGC
---GGGGTCAAGTGC------
GTCGAATCCGGCGGACCTGAGCCGGGTGTCGGGTGTGCCGGTCGCGGGGTGATCACTGCGATCAACTTTCTCGAG
GAGGAAGGCGCCTA---TGAGG------AAGATCTCGATTTCGTTTTCTACGACGTGCTCGG----------
CGACGTCGTCTGCGGTGGCTTTGCCATGCCGATCCGCGAAAACAAGGCCCAGGAGATCTATATCGT-------

>HM210343.1_Gamma2/1-317

TCAACTCGTCTGATCCTTCACTCTAAAGCTCAAACTACT---
GTTATGCATCTGGCTGCTGAGGCCGGTACCGTAGAAGACCTGGAGCTGGAAGATGTATTGTCTGTCGGTTACGGC-
--GACGTTAAATGT------
GTTGAGTCTGGTGGTCCTGAGCCAGGCGTAGGTTGTGCCGGTCGTGGTGTAATCACTGCCATCAACTTCCTGGAAG
AAGAAGGCGCTTA---CTACG------AATATCTGGACTTCGTATTCTACGATGTACTGGG----------
TGACGTTGTATGTGGTGGTTTCGCGATGCCAATTCGTGAAAACAAAGCTCAAGAGATCTACAT----------

>KF151819.1_GammaETSP2/1-358

TCTACACGCCTTATTTTGCATTCCAAAGCTCAAAACACA---
ATCATGGAAATGGCTGCTCAGGCAGGCACGGTTGAGGATCTCGAGCTCGATGACGTGCTTAAAGTTGGTTATGGT-
--AACATTAAGTGT------
GTAGAAGCAGGCGGTCCAGAGCCCGGCGTTGGGTGTGCCGGTCGTGGTGTTATTACTGCGATTAATTTCCTTGAA
GAAGAGGGTGCGTA---TGAAG------AAGATCTAGACTTTGTTTTCTATGATGTTCTTGG----------
CGACGTGGTCTGCGGCGGTTTTGCTATGCCAATTCGCGAAAATAAGGCTCAAGAAATTTACATTGTCTGCTCT

>KF151567.1_GammaETSP1/1-358

TCAACTCGTCTGATCCTTCACTCAAAAGCTCAAAACACA---
ATCATGGAAATGGCTGCCGAAGCCGGTACCGTGGAAGATCTTGAGTTAGAAGATGTATTAAAAATGGGTTACGGC-
--AACGTTAAGTGC------
GTTGAGTCCGGTGGTCCAGAGCCAGGTGTTGGTTGTGCCGGCCGTGGTGTTATCACTGCTATCAACTTCTTAGAAG
AAGAAGGTGCTTA---CGACG------ATGACCTAGACTTCGTATTCTATGATGTATTGGG----------
TGACGTGGTATGTGGTGGATTCGCCATGCCCATTCGTGAGAACAAAGCGCAAGAAATCTACATTGTTTGTTCT

>KF151661.1_GammaETSP3/1-358

TCAACGCGTTTGATTCTGCACTCTAAAGCACAAAACACC---
ATCATGGAAATGGCTGCTGAAGCCGGTACGGTTGAAGACCTAGAGTTGGAAGACGTATTAAAAACGGGCTACGGC
---GACATCAAGTGC------
GTTGAGTCTGGTGGTCCAGAACCAGGTGTTGGTTGTGCTGGTCGCGGTGTAATCACTGCTATCAACTTCCTTGAGG
AAGAAGGTGCGTA---CGAAG------ACGATCTTGACTTCGTATTCTACGACGTATTGGG----------
TGACGTTGTATGTGGTGGTTTCGCGATGCCCATTCGTGAAAACAAAGCTCAAGAAATCTACATCGTTGTATCT

>AB198375.1_PCRReagent_2

TCCACCCGTCTGATCCTTCACGCTAAAGCCCAGAACACC---
ATCATGGAGATGGCGGCGGAAGTGGGCTCGGTCGAGGATCTGGAGCTCGAAGACGTTCTGCAAATCGGCTATGG
C---GATGTCCGTTGC------
GCCGAATCCGGCGGCCCGGAGCCAGGCGTCGGCTGCGCCGGACGCGGGGTGATCACCGCCATCAACTTCCTCGAG
GAAGAGGGCGCCTA---TGAAG------AAGATTTGGATTTCGTCTTCTATGACGTCCTCGG----------C----------------------
----

>AB198386.1_PCRReagent_3

TCCACCCGCCTGATCCTGCACGCTAAAGCACAGAACACC---
ATTATGGAGATGGCCGCGGAAGTCGGCTCGGTCGAGGATCTCGAGCTCGAAGACGTGCTGCAAATTGGCTACGGC
---GACGTGCGCTGC------
GCGGAATCCGGCGGCCCGGAGCCAGGCGTCGGCTGCGCGGGGCGCGGCGTGATCACGGCGATCAACTTTCTTGA
AGAAGAAGGCGCCTA---CGAGG------ACGATCTCGATTTTGTCTTCTATGACGTGCTCGG----------C--------------------
----

>HM042879.1_PCRReagent_2

TCCACCCGCCTGATCCTGCACGCCAAAGCACAGAACACC---
ATTATGGAGATGGCCGCGGAAGTCGGCTCGGTCGAGGACCTCGAACTCGAAGACGTTCTGCAAATTGGCTACGGC
---GACGTGCGCTGC------
GCGGAATCCGGCGACCCGGAGCCAGGCGTCGGCTGTGCGGGACGCGGCGTGATCACGGCGATCAACTTTCTTGA
AGAAGAAGGCGCCTA---CAAGG------ACGATCTCGATTTCGTGTCCTATGACGTGCTCGG----------
CGACGTGGTCTGCGGTGGCTTCGCCATGCCGATCCGCGAAAACAAAGCCCAGGAGATCTACATCGTCTGCTCC

>HM042885.1_PCRReagent_4

TCCACCCGCCTGATCCTGCACGCCAAAGCACAGAACACC---
ATTATGGAGATGGCCGCGGAAGTCGGCTCGGTCGAGGACCTCGAACTCGAAGACGTTCTGCAAATTGGCTACGGC
---GACGTGCGCTGC------
GCGGAATCCGGCGGCCCGGAGCCAGGCGTCGGCTGTGCGGGACGCGGCGTGATCACGGCGATCAACTTTCTTGA
AGGAGAAGGCGCCTA---CGAGG------ACGATCTCGATTTCGTGTTCTATGACGTACTCGG----------
CGACGTGGTCTGCGGTGGCTTCGCCATGCCGATCCGCGAAAACAAAGCCCAGGAGATCTACATCGTCTGCTCC
