# Supplementary material for: Widespread Distribution and Expression of Gamma A (UMB), an Uncultured, Diazotrophic, γ-Proteobacterial nifH Phylotype
Source: PLoS One. 2015 Jun 23;10(6):e0128912. doi: 10.1371/journal.pone.0128912 (PMC4477881; doi:10.1371/journal.pone.0128912)
Supplement: S3 Alignment — (PDF) [file pone.0128912.s003.pdf]

>AY896371.1_GammaA_313_23

TCCACACGTCTTATTCTGCACTCAAAAGCGCAAAACACAATAATGGAGATG---
GCTGCACAGGCTGGCACGGTAGAG---
GATCTTGAGCTTGAAGAAGTGCTTAAGGTTGGCTTTGGCGACATTAAGTGCGTGGAGTCAGGTGGTCCTGAGCCT
GGAGTTGGCTGTGCTGGTCGTGGTGTTATTACCGCG---ATTAACTTCCTTGAAGAGGAAGGTGCATAC---
TCAGACGACTTAGATTTCGTTTTTATGATGTT---
CTAGGTGATGTGGTTTGTGGCGGATTTGCAATGCCTATTCGTGAGAATAAGGCTCAGGAAATCTACATTGTTTGCT
CT

>HQ611810.1_Uncultured_28_5

TCTACACGTCTTATTCTGCACTCAAAAGCGCAAAACACAATAATGGAGATG---
GCTGCACAGGCTGGCACGGTAGAG---
GATCTTGAGCTTGAAGAAGTGCTTAAGGTTGGCTTTGGCGATGTTAAGTGCGTGGAGTCAGGTGGTCCTGAGTCT
GGAGTTGGCTGTGCTGGTCGTGGTGTTATTACCGCG---ATTAATTTCCTTGAAGAGGAAGGTGCATAC---
ACAGACGACTTAGATTTCGTTTTCTATGATGTT---
CTAGGTGATGTGGTTTGTGGCGGATTTGCAATGCCTATTCGTGAGAACAAGGCTCAGGAAATCTACATTGTTTGCT
CT

>KF151819.1_GammaETSP2_42

TCTACACGCCTTATTTTGCATTCCAAAGCTCAAAACACAATCATGGAAATG---
GCTGCTCAGGCAGGCACGGTTGAG---
GATCTCGAGCTCGATGACGTGCTTAAAGTTGGTTATGGTAACATTAAGTGTGTAGAAGCAGGCGGTCCAGAGCCC
GGCGTTGGGTGTGCCGGTCGTGGTGTTATTACTGCG---ATTAATTTCCTTGAAGAAGAGGGTGCGTAT---
GAAGAAGATCTAGACTTTGTTTTCTATGATGTT---
CTTGGCGACGTGGTCTGCGGCGGTTTTGCTATGCCAATTCGCGAAAATAAGGCTCAAGAAATTTACATTGTCTGCTC
T

>KF151762.1_Uncultured_4_2

TCTACACGACTTATTCTGCACTCAAAAGCGCAAAACACTATAATGGAGATG---
GCCGCGCAGGCTGGCACGGTAGAA---
GATCTCGAGCTTGAAGAAGTGCTTAAAGTTGGTTATGGCGACATTAAGTGCGTTGAATCAGGTGGACCAGAGCCA
GGTGTTGGCTGTGCCGGTCGTGGTGTTATTACCGCG---ATTAACTTCCTTGAAGAGGAAGGTGCATAT---
TCAGACGACCTAGATTTCGTATTTTATGATGTC---
CTTGGGGACGTGGTCTGTGGTGGATTTGCAATGCCTATTCGTGAGAATAAGGCTCAGGAAATTTACATTGTTTGTT
CT

>KF151817.1_Uncultured_14_1

TCTACACGCCTTATTTTGCATTCCAAAGCTCAAAACACAATCATGGAAATG---
GCTGCTCAGGCAGGCACGGTTGAG---
GATCTCGAGCTAGAAGATGTTCTAAAGGTTGGATACGGCGACATTAAGTGTGTAGAAGCAGGCGGTCCAGAGCCC

GGCGTTGGATGTGCCGGTCGCGGTGTTATTACCGCA---ATTAATTTCCTTGAAGAAGAGGGTGCGTAT---
GAAGACGATTTGGACTTTGTTTTCTATGACGTT---
CTTGGCGACGTGGTCTGCGGCGGTTTTGCTATGCCAATTCGCGAAAATAAGGCTCAAGAAATTTACATTGTCTGCTC
T

>HQ611853.1_Uncultured_6_1

TCTACACGTCTCATCTTGCACTCCAAGGCTCAAAACACCATCATGGAGATG---
GCTGCTCAGGCAGGCACGGTTGAG---
GATTTAGAGCTGGAAGACGTTCTGAAGGTTGGATATGGCGACATTAAGTGTGTAGAAGCAGGCGGTCCAGAGCCC
GGCGTTGGTTGTGCCGGCCGCGGCGTTATCACTGCG---ATTAACTTTCTTGAGGAAGAGGGTGCATAT---
GAAGACGATCTAGACTTTGTTTTCTATGATGTC---
CTTGGCGACGTGGTCTGCGGCGGTTTTGCTATGCCAATTCGCGAGAATAAGGCTCAAGAAATCTACATTGTTTGCT
CT

>HQ611839.1_Uncultured_13_1

TCTACGCGTCTTATCTTGCATTCCAAAGCCCAAAACACAATCATGGAAATG---
GCTGCCCAGGCAGGTACGGTTGAG---
GATTTAGAGCTTGATGATGTACTTAAGGTTGGCTACGGCGATATTAAGTGTGTAGAAGCGGGCGGTCCAGAGCCC
GGCGTTGGGTGTGCCGGTCGCGGTGTTATTACTGCG---ATTAACTTTCTTGAAGAAGAGGGTGCATAT---
GAAGACGATCTAGACTTTGTTTTCTATGATGTT---
CTGGGCGACGTGGTCTGCGGCGGCTTTGCTATGCCAATTCGTGAAAACAAAGCTCAAGAGATCTACATTGTTTGCT
CT

>HM210397.1_Gamma3_23_4

TCCACGCGTCTTATCTTGCACTCCAAAGCCCAAAACACAATCATGGAAATG---
GCTGCCCAGGCAGGTACGGTTGAG---
GATTTAGAGCTTGATGACGTACTTAAGGTTGGCTACGGCGATATTAAGTGTGTAGAAGCAGGCGGTCCAGAGCCC
GGCGTTGGTTGTGCCGGTCGCGGTGTTATTACCGCG---ATTAACTTCCTTGAGGAAGAAGGTGCATAC---
GAAGACGATCTAGACTTTGTTTTCTATGATGTT---
CTGGGCGACGTGGTCTGCGGCGGCTTTGCTATGCCAATTCGTGAAAACAAAGCTCAAGAGATCTACATTGTTTGCT
CT

>HQ456037.1_Uncultured_11_5

TCCACACGTCTTATTCTGCACTCAAAAGCGCAAAACACAATAATGGAGATG---
GCTGCACAGGCAGGCACGGTAGAG---
GATCTGGAGCTAGAAGACGTGCTTAAAGTTGGTTATGGTGACATTAAGTGTGTAGAATCAGGTGGTCCCGAGCCC
GGTGTTGGGTGTGCTGGTCGTGGTGTTATCACTGCG---ATTAATTTCCTCGAAGAGGAAGGCGCGTAT---
GAAGACGATCTGGACTTTGTTTTTTACGACGTT---
CTTGGGGACGTGGTTTGTGGCGGTTTTGCCATGCCAATTCGTGAAAACAAAGCGCAGGAAATCTACATTGTTTGCT
CT

>AY896456.1_Uncultured_14_5

TCCACCCGTCTTATCTTGCATTCTAAGGCTCAAAATACCATCATGGAAATG---
GCTGCTCAGGCAGGTACGGTAGAG---
GATCTTGAGCTTGATGACGTGCTTAAAGTTGGATATGGCAACATTAAGTGTGTAGAATCAGGTGGTCCCGAGCCCG
GTGTTGGGTGTGCTGGTCGTGGTGTTATCACTGCG---ATTAATTTCCTCGAAGAGGAAGGCGCGTAT---
GAAGACGATCTGGACTTTGTTTTTTACGACGTT---
CTTGGGGACGTGGTTTGTGGCGGTTTTGCCATGCCAATTCGTGAAAACAAAGCGCAGGAAATCTACATTGTTTGCT
CT

>AB679086.1_Uncultured_4_3

TCCACGCGTCTTATTCTGCATTCAAAAGCGCAAAATACTATCATGGAAATG---
GCTGCAGAGGCTGGCACGGTAGAG---
GATCTAGAGCTCGAAGAAGTGCTGAAGGTCGGTTTTGGTGATATTAAATGTGTCGAATCAGGCGGTCCAGAGCCT
GGCGTTGGTTGTGCTGGCCGCGGTGTTATTACGGCA---ATTAACTTTCTTGAAGAGGAAGGTGCTTAC---
GAAGAAGACTTAGATTTCGTTTTCTATGATGTA---
TTGGGAGACGTCGTTTGCGGCGGCTTCGCGATGCCCATCCGTGAAAACAAAGCCCAAGAAATATACATCGTTTGCT
C

>CP001968.1_Denitrovibrio

TCAACAAGACTTATACTTCACTCTAAAGCACAATCAACTATAATGGAGCTC---
GCTGCTGAAGCAGGCTCAGTTGAA---
GACCTTGAACTTGATGACGTTCTCAAAGCCGGTTATCTTGATATACGCTGCGTAGAGGCAGGCGGTCCGGAACCCG
GTGTCGGCTGTGCTGGTCGTGGTGTTATTACCGCT---ATCAACTTCCTTGAGGAAGAGGGTGCATAC---
GAAGAAGACCTCGATTTCGTTTCATATGACGTT---
CTCGGTGACGTTGTTTGCGGTGGTTTCGCAATGCCTATTCGTGAGGGTAAAGCTCAGGAGATATACATTGTT------

>AY896428.1_GammaP_03

TCTACTCGTCTGATTCTTCACTCTAAAGCTCAAACTACTGTTATGCACTTG---GCTGCAGAAGCAGGCACGGTAGAA-
--
GACCTGGAGCTGGAAGATGTACTGTCTGTTGGCTACGGCGACGTTAAATGCGTTGAGTCTGGTGGCCCTGAGCCA
GGTGTTGGTTGTGCAGGTCGTGGTGTAATCACTGCA---ATCAACTTCCTGGAAGAAGAAGGTGCCTAT---
GACGAAGACCTAGACTTCGTATTCTACGACGTA---
TTGGGTGACGTTGTATGTGGTGGTTTCGCGATGCCTATTCGTGAAAACAAAGCACAAGAAATCTACATCGTATGTT
CT

>HQ586273.1_Zhang_17

TCCACCCGCCTCATTCTGCACTCCAAAGCGCAGAATACCATCATGGAAATG---
GCGGCTGAAGCTGGCACCGTTGAA---
GACCTCGAGCTCGAAGATGTGCTCAAGGTTGGCTACGGCGACATCAAGTGCGTCGAATCCGGCGGTCCAGAGCCA
GGCGTCGGTTGCGCCGGTCGCGGCGTCATCACCGCC---ATCAACTTCCTTGAAGAAGAAGGCGCATAC---

GAAGAAGACCTCGACTTCGTTTTCTACGACGTC---
CTCGGCGACGTTGTTTGCGGTGGCTTCGCTATGCCGATCCGCGAAAACAAGGCTCAGGAAATCTACATCGTTTGCT
C-

>M63691.1_Klebsiella_03

TCAACCCGGCTGATTCTTCACTCTAAAGCGCAAAACACGATTATGGAAATG---
GCTGCTGAAGCTGGCTCTGTTGAA---
GATATCGAACTGGAAGATGTATTGAAAGTCGGTTACGGCGACGTGCGCTGTGTTGAGTCTGGTGGTCCTGAGCCT
GGTGTTGGCTGTGCCGGTCGCGGGGTGATTACGGCA---ATTAACTTCCTTGAAGAAGAAGGTGCTTAC---
GAAGAAGATCTGGACTTTGTGTTCTATGACGTT---
CTTGGTGACGTTGTGTGTGGTGGTTTCGCGATGCCAATTCGTGAAAACAAAGCACAGGAAATCTATATCGT-------

>HM210363.1_Gamma4_133

TCAACTCGTCTGATCCTTCACTCAAAAGCTCAAAACACAATCATGGAAATG---
GCTGCCGAAGCCGGTACCGTGGAA---
GATCTTGAGTTAGAAGATGTATTAAAAATGGGTTACGGCAACGTTAAGTGCGTTGAGTCCGGTGGTCCAGAGCCA
GGTGTTGGTTGTGCCGGCCGTGGTGTTATCACTGCT---ATCAACTTCTTAGAAGAAGAAGGTGCTTAC---
GACGATGACCTAGACTTCGTATTCTATGATGTA---
TTGGGTGACGTGGTATGTGGTGGATTCGCCATGCCCATTCGTGAGAACAAAGCGCAAGAAATCTACATTGTTTGTT
CT

>DQ913881.1_Celerinatantimonas_06

TCTACTCGCCTTATGCTTCACGCGAAAGCTCAGAACACCATTATGGAAATG---
GCTGCTGAAGCAGGTTCGGTTGAA---
GATCTTGAACTAGAGGATGTGCTAAAAGTTGGCTACGGCGGGGTGAAATGCGTTGAATCAGGTGGCCCAGAGCCG
GGCGTTGGTTGTGCTGGTCGTGGGGTTATCACAGCG---ATTAACTTCCTCGAAGAAGAAGGCGCTTAC---
GATGATGACTTAGATTTTGTTTTTTATGACGTA---
CTTGGTGACGTTGTATGCGGCGGGTTCGCAATGCCTATTCGCGAAAACAAAGCGCAGGAAATCTACATCGT-------

>AF111110.2_Vibrio_12

TCAACTCGTCTCATCCTGCACTCAAAAGCACAAAACACCATCATGGAAATG---
GCAGCGGAAGCCGGTACGGTTGAA---
GACATCGAACTAGAAGATGTATTGAAAGTCGGTTATGGCGATGTTCGCTGTGTGGAATCAGGCGGCCCTGAGCCA
GGCGTAGGTTGTGCTGGTCGCGGTGTTATCACAGCA---ATCAACTTCCTCGAAGAAGAAGGCGCGTAT---
GAAGATGACTTAGATTTCGTTTTCTACGACGTA---
TTGGGTGACGTTGTGTGTGGTGGTTTCGCGATGCCAATTCGTGAAAACAAAGCGCAAGAAATCTACATCGTATGTT
CT

>HM210377.1_Gamma1_12

TCCACGCGTTTGATCCTGCACTCCAAAGCTCAAACCACTGTGATGCATCTG---
GCTGCCGAAGCCGGCACCGTGGAA---
GATCTGGAGCTGGAAGATGTGCTGTCTGTCGGTTACGGCGATGTAAAATGCGTCGAGTCCGGTGGTCCCGAGCCG
GGTGTCGGCTGCGCCGGTCGCGGTGTTATCACCGCC---ATCAACTTCCTGGAAGAGGAAGGCGCTTAC---
GACGAAGATCTGGACTTCGTATTCTACGATGTC---
CTGGGCGACGTGATCTGCGGTGGCTTTGCTATGCCCATCCGTGAAAACAAAGCGCAAGAAATCTACATCGT-------

>HM210343.1_Gamma2_05

TCAACTCGTCTGATCCTTCACTCTAAAGCTCAAACTACTGTTATGCATCTG---GCTGCTGAGGCCGGTACCGTAGAA-
--
GACCTGGAGCTGGAAGATGTATTGTCTGTCGGTTACGGCGACGTTAAATGTGTTGAGTCTGGTGGTCCTGAGCCA
GGCGTAGGTTGTGCCGGTCGTGGTGTAATCACTGCC---ATCAACTTCCTGGAAGAAGAAGGCGCTTAC---
GACGAAGATCTGGACTTCGTATTCTACGATGTA---
CTGGGTGACGTTGTATGTGGTGGTTTCGCGATGCCAATTCGTGAAAACAAAGCTCAAGAGATCTACATCGTATGTT
CT

>CP002738.1_Methylomonas

TCCACACGTTTAATTCTACACGCAAAAGCGCAAAACTCCATCATGCAAATG---
GCGGCCGATGCAGGTAGCGTTGAA---
GATTTGGAATTGGAAGACGTATTGAAAGTGGGTTACCGCGACATTAAATGCGTTGAGTCCGGCGGCCCAGAGCCA
GGCGTTGGTTGTGCCGGCCGCGGTGTTATCACTGCC---ATCAACTTCCTGGAAGAAGAAGGCGCTTAC---
GACGAAAACCTGGACTTCGTGTTCTACGACGTA---
TTGGGTGACGTTGTGTGCGGCGGTTTCGCGATGCCGATTCGCGAAAACAAAGCGCAAGAAATTTATATCGTTTGCT
C-

>KF151567.1_GammaETSP1_136

TCAACTCGTCTGATCCTTCACTCAAAAGCTCAAAACACAATCATGGAAATG---
GCTGCCGAAGCCGGTACCGTGGAA---
GATCTTGAGTTAGAAGATGTATTAAAAATGGGTTACGGCAACGTTAAGTGCGTTGAGTCCGGTGGTCCAGAGCCA
GGTGTTGGTTGTGCCGGCCGTGGTGTTATCACTGCT---ATCAACTTCTTAGAAGAAGAAGGTGCTTAC---
GACGATGACCTAGACTTCGTATTCTATGATGTA---
TTGGGTGACGTGGTATGTGGTGGATTCGCCATGCCCATTCGTGAGAACAAAGCGCAAGAAATCTACATTGTTTGTT
CT

>KF151661.1_GammaETSP3_28

TCAACGCGTTTGATTCTGCACTCTAAAGCACAAAACACCATCATGGAAATG---
GCTGCTGAAGCCGGTACGGTTGAA---
GACCTAGAGTTGGAAGACGTATTAAAAACGGGCTACGGCGACATCAAGTGCGTTGAGTCTGGTGGTCCAGAACCA
GGTGTTGGTTGTGCTGGTCGCGGTGTAATCACTGCT---ATCAACTTCCTTGAGGAAGAAGGTGCGTAC---
GAAGACGATCTTGACTTCGTATTCTACGACGTA---

TTGGGTGACGTTGTATGTGGTGGTTTCGCGATGCCCATTCGTGAAAACAAAGCTCAAGAAATCTACATCGTTGTAT
CT
