# Supplementary figures and images for: Widespread Distribution and Expression of Gamma A (UMB), an Uncultured, Diazotrophic, γ-Proteobacterial nifH Phylotype
Source: PLoS One. 2015 Jun 23;10(6):e0128912. doi: 10.1371/journal.pone.0128912 (PMC4477881; doi:10.1371/journal.pone.0128912)

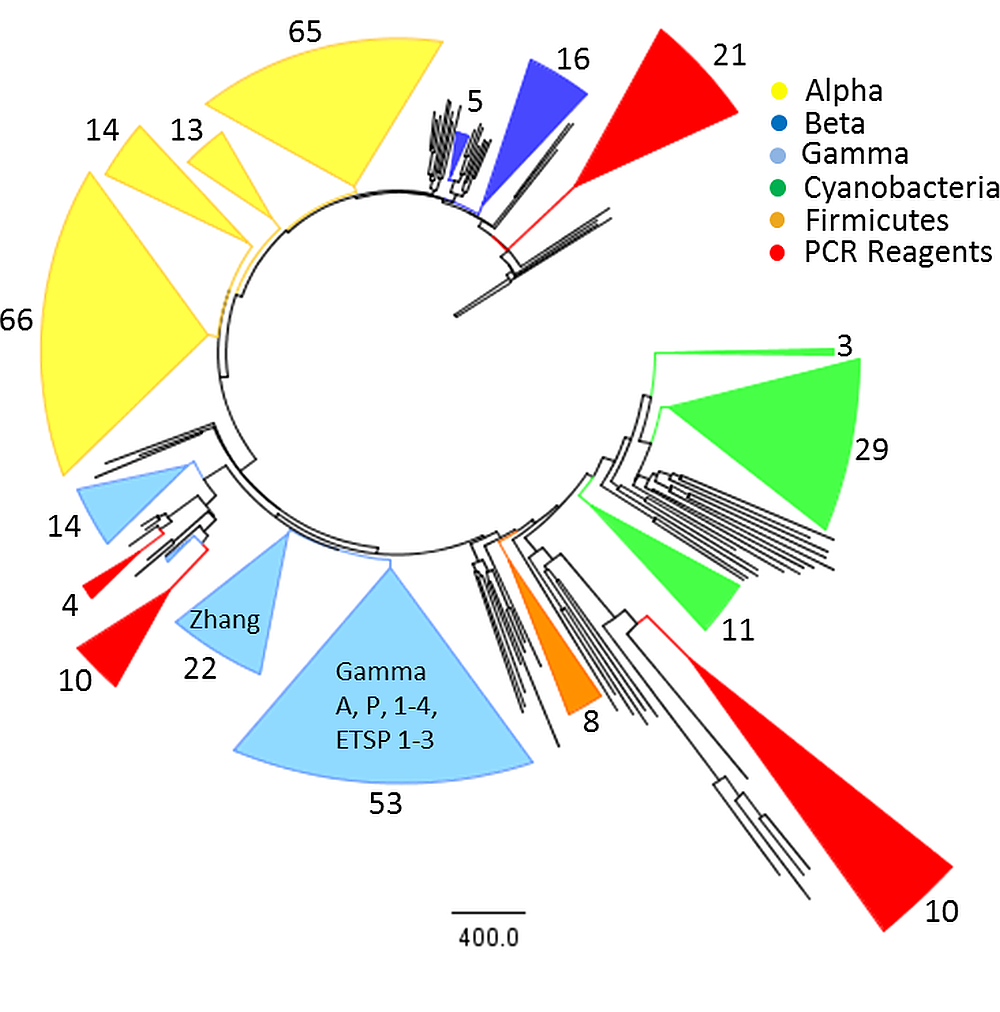

Supplement: S1 Fig — The tree was constructed using the neighbor joining method. Clades have been collapsed taxonomically by class according to the given color scheme. The number of sequences comprising each clade is shown. Clades that are not collapsed contained sequences originating from multiple taxonomic classes. The clades containing the qPCR phylotype targets are labeled. Clades containing sequences originating from PCR reagents are colored red. Sequences from all clades identified as γ-proteobacteria were used in further analyses. (TIF) [file pone.0128912.s004.tif]

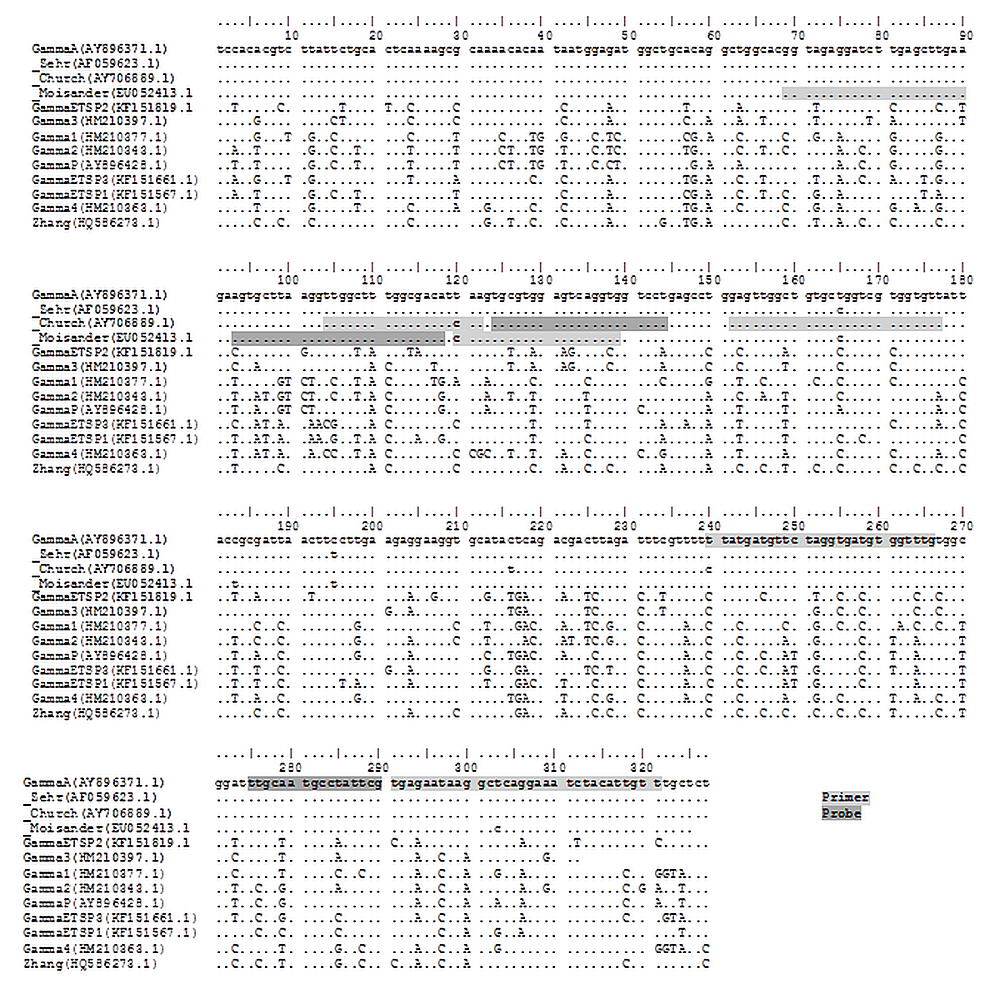

Supplement: S2 Fig — The full Gamma A amplicon is shown at the top. The amplicons used in other studies follow, identified by either the first author’s name or probe name and sequence accession number in brackets. The first six sequences (bold font) are in the Marine 1 clade. The Gamma A primers (light grey bars) and probes (dark grey bars) used in qPCR studies are shown. (TIF) [file pone.0128912.s005.tif]

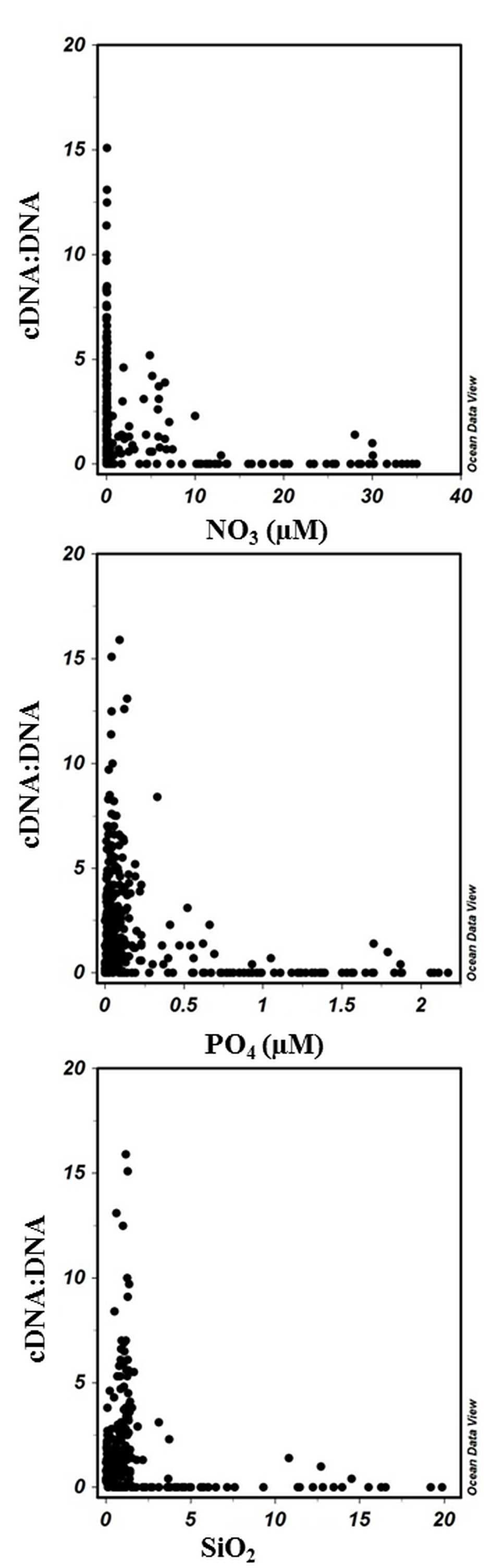

Supplement: S3 Fig — Note that the x-axis scale changes in each panel. (TIF) [file pone.0128912.s006.tif]

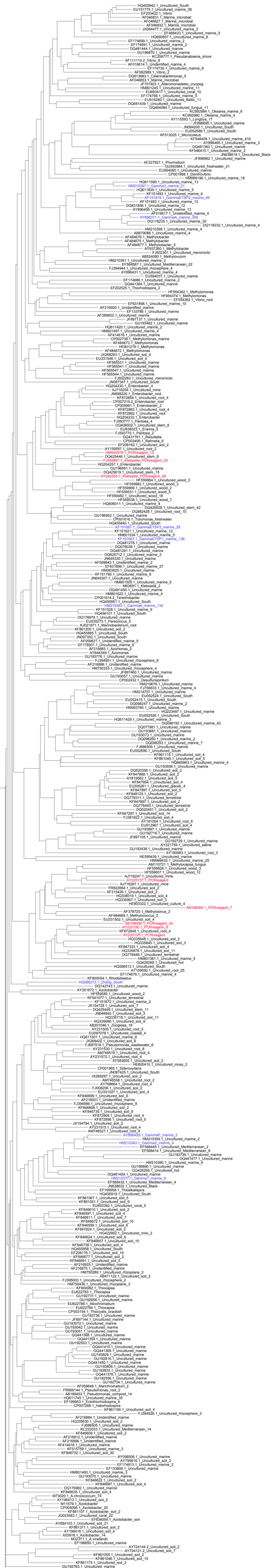

Supplement: S4 Fig — This tree is a reproduction of Fig 1, including the accession numbers, first word in title, the number of sequences in the OTU, and isolation environmental source (if available). (PDF) [file pone.0128912.s007.pdf]
