# Supplementary material for: Widespread Distribution and Expression of Gamma A (UMB), an Uncultured, Diazotrophic, γ-Proteobacterial nifH Phylotype
Source: PLoS One. 2015 Jun 23;10(6):e0128912. doi: 10.1371/journal.pone.0128912 (PMC4477881; doi:10.1371/journal.pone.0128912)
Supplement: S1 Table — (PDF) [file pone.0128912.s008.pdf]

S1 Table. List of accession numbers for sequences identified as γ-proteobacteria.

AB184893.1_Uncultured_root
AB184897.1_Uncultured_root
AB184908.1_Uncultured_root
AB189453.1_Pseudomonas_compost
AB198366.1_PCRreagent
AB198367.1_PCRreagent
AB198368.1_PCRreagent
AB198369.1_PCRreagent
AB198370.1_PCRreagent
AB198371.1_PCRreagent
AB198372.1_PCRreagent
AB198373.1_PCRreagent
AB198374.1_PCRreagent
AB198375.1_PCRreagent
AB198376.1_PCRreagent
AB198377.1_PCRreagent
AB198378.1_PCRreagent
AB198379.1_PCRreagent
AB198380.1_PCRreagent
AB198381.1_PCRreagent
AB198382.1_PCRreagent
AB198383.1_PCRreagent
AB198384.1_PCRreagent
AB198385.1_PCRreagent
AB198386.1_PCRreagent
AB198387.1_PCRreagent
AB198388.1_PCRreagent
AB198389.1_PCRreagent
AB198390.1_PCRreagent
AB198391.1_PCRreagent
AB201045.1_Zoogloea
AB201046.1_Zoogloea
AB208313.1_Uncultured_root
AB430882.1_Azotobacter_soil
AB471048.1_Uncultured_soil
AB471080.1_Uncultured_soil
AB471122.1_Uncultured_soil
AB471165.1_Uncultured_soil
AB471195.1_Uncultured_soil
AB471267.1_Uncultured_soil
AB471273.1_Uncultured_soil
AB524080.1_Methylovulum

AB665496.1_Uncultured_South China Sea
AB678802.1_Uncultured_marine
AB678803.1_Uncultured_marine
AB678804.1_Uncultured_marine
AB678807.1_Uncultured_South China Sea
AB678808.1_Uncultured_marine
AB678809.1_Uncultured_marine
AB678811.1_Uncultured_marine
AB678812.1_Uncultured_marine
AB678813.1_Uncultured_marine
AB678814.1_Uncultured_marine
AB678815.1_Uncultured_marine
AB678816.1_Uncultured_marine
AB678817.1_Uncultured_marine
AB678818.1_Uncultured_marine
AB678819.1_Uncultured_marine
AB678820.1_Uncultured_marine
AB678821.1_Uncultured_marine
AB678823.1_Uncultured_marine
AB678824.1_Uncultured_marine
AB678826.1_Uncultured_marine
AB678827.1_Uncultured_marine
AB678829.1_Uncultured_marine
AB678831.1_Uncultured_marine
AB678833.1_Uncultured_marine
AB678835.1_Uncultured_marine
AB678837.1_Uncultured_marine
AB678839.1_Uncultured_marine
AB678840.1_Uncultured_marine
AB678844.1_Uncultured_marine
AB678845.1_Uncultured_marine
AB678846.1_Uncultured_marine
AB678847.1_Uncultured_marine
AB678848.1_Uncultured_marine
AB678849.1_Uncultured_marine
AB678850.1_Uncultured_marine
AB678851.1_Uncultured_marine
AB678852.1_Uncultured_marine
AB678853.1_Uncultured_marine
AB678854.1_Uncultured_marine
AB678855.1_Uncultured_marine
AB678856.1_Uncultured_marine
AB678857.1_Uncultured_marine
AB678858.1_Uncultured_marine

AB678859.1_Uncultured_marine
AB678860.1_Uncultured_marine
AB678861.1_Uncultured_marine
AB678862.1_Uncultured_marine
AB678863.1_Uncultured_marine
AB678864.1_Uncultured_marine
AB678867.1_Uncultured_marine
AB678868.1_Uncultured_marine
AB678869.1_Uncultured_marine
AB678870.1_Uncultured_marine
AB678871.1_Uncultured_marine
AB678873.1_Uncultured_marine
AB678874.1_Uncultured_marine
AB678877.1_Uncultured_marine
AB678878.1_Uncultured_marine
AB678882.1_Uncultured_marine
AB678883.1_Uncultured_marine
AB678884.1_Uncultured_marine
AB678888.1_Uncultured_marine
AB678889.1_Uncultured_marine
AB678891.1_Uncultured_marine
AB678893.1_Uncultured_marine
AB678894.1_Uncultured_marine
AB678896.1_Uncultured_marine
AB678898.1_Uncultured_marine
AB678899.1_Uncultured_marine
AB678901.1_Uncultured_marine
AB678902.1_Uncultured_marine
AB678903.1_Uncultured_marine
AB678905.1_Uncultured_marine
AB678906.1_Uncultured_marine
AB678907.1_Uncultured_marine
AB678908.1_Uncultured_marine
AB678909.1_Uncultured_marine
AB678910.1_Uncultured_marine
AB678912.1_Uncultured_marine
AB678914.1_Uncultured_marine
AB678915.1_Uncultured_marine
AB678916.1_Uncultured_marine
AB678917.1_Uncultured_marine
AB678919.1_Uncultured_marine
AB678921.1_Uncultured_marine
AB678922.1_Uncultured_marine
AB678923.1_Uncultured_marine

AB678924.1_Uncultured_marine
AB678925.1_Uncultured_marine
AB678926.1_Uncultured_marine
AB678927.1_Uncultured_marine
AB678929.1_Uncultured_marine
AB678930.1_Uncultured_marine
AB678931.1_Uncultured_marine
AB678933.1_Uncultured_marine
AB678941.1_Uncultured_marine
AB678942.1_Uncultured_marine
AB678943.1_Uncultured_marine
AB678945.1_Uncultured_marine
AB678946.1_Uncultured_marine
AB678947.1_Uncultured_marine
AB678948.1_Uncultured_marine
AB678950.1_Uncultured_marine
AB678951.1_Uncultured_marine
AB678952.1_Uncultured_marine
AB678954.1_Uncultured_marine
AB678955.1_Uncultured_marine
AB678956.1_Uncultured_marine
AB678957.1_Uncultured_marine
AB678959.1_Uncultured_marine
AB678960.1_Uncultured_marine
AB678963.1_Uncultured_marine
AB678964.1_Uncultured_marine
AB678965.1_Uncultured_marine
AB678966.1_Uncultured_marine
AB678967.1_Uncultured_marine
AB678968.1_Uncultured_marine
AB678969.1_Uncultured_marine
AB678970.1_Uncultured_marine
AB678972.1_Uncultured_marine
AB678973.1_Uncultured_marine
AB678974.1_Uncultured_marine
AB678975.1_Uncultured_marine
AB678977.1_Uncultured_marine
AB678979.1_Uncultured_marine
AB678980.1_Uncultured_marine
AB678981.1_Uncultured_marine
AB678982.1_Uncultured_marine
AB678983.1_Uncultured_marine
AB678984.1_Uncultured_marine
AB678986.1_Uncultured_marine

AB678987.1_Uncultured_marine
AB678988.1_Uncultured_marine
AB678990.1_Uncultured_marine
AB678991.1_Uncultured_marine
AB678992.1_Uncultured_marine
AB678993.1_Uncultured_marine
AB678994.1_Uncultured_marine
AB678996.1_Uncultured_marine
AB678997.1_Uncultured_marine
AB678998.1_Uncultured_marine
AB678999.1_Uncultured_marine
AB679000.1_Uncultured_marine
AB679002.1_Uncultured_marine
AB679003.1_Uncultured_marine
AB679004.1_Uncultured_marine
AB679005.1_Uncultured_marine
AB679007.1_Uncultured_marine
AB679008.1_Uncultured_marine
AB679009.1_Uncultured_marine
AB679011.1_Uncultured_marine
AB679012.1_Uncultured_marine
AB679014.1_Uncultured_South China Sea
AB679015.1_Uncultured_South China Sea
AB679016.1_Uncultured_South China Sea
AB679017.1_Uncultured_South China Sea
AB679019.1_Uncultured_South China Sea
AB679021.1_Uncultured_South China Sea
AB679026.1_Uncultured_South China Sea
AB679027.1_Uncultured_South China Sea
AB679028.1_Uncultured_marine
AB679029.1_Uncultured_marine
AB679031.1_Uncultured_marine
AB679033.1_Uncultured_marine
AB679034.1_Uncultured_marine
AB679035.1_Uncultured_marine
AB679036.1_Uncultured_marine
AB679037.1_Uncultured_South China Sea
AB679038.1_Uncultured_South China Sea
AB679039.1_Uncultured_marine
AB679040.1_Uncultured_South China Sea
AB679041.1_Uncultured_marine
AB679045.1_Uncultured_marine
AB679046.1_Uncultured_marine
AB679048.1_Uncultured_South China Sea

AB679050.1_Uncultured_South China Sea
AB679052.1_Uncultured_South China Sea
AB679054.1_Uncultured_marine
AB679057.1_Uncultured_marine
AB679059.1_Uncultured_marine
AB679061.1_Uncultured_marine
AB679062.1_Uncultured_marine
AB679063.1_Uncultured_South China Sea
AB679064.1_Uncultured_marine
AB679065.1_Uncultured_South China Sea
AB679066.1_Uncultured_South China Sea
AB679067.1_Uncultured_South China Sea
AB679068.1_Uncultured_South China Sea
AB679069.1_Uncultured_South China Sea
AB679070.1_Uncultured_South China Sea
AB679071.1_Uncultured_marine
AB679073.1_Uncultured_South China Sea
AB679074.1_Uncultured_South China Sea
AB679075.1_Uncultured_marine
AB679076.1_Uncultured_marine
AB679078.1_Uncultured_marine
AB679079.1_Uncultured_South China Sea
AB679080.1_Uncultured_South China Sea
AB679081.1_Uncultured_South China Sea
AB679082.1_Uncultured_South China Sea
AB679083.1_Uncultured_South China Sea
AB679084.1_Uncultured_South China Sea
AB679085.1_Uncultured_South China Sea
AB679086.1_Uncultured_marine
AB679087.1_Uncultured_South China Sea
AB679088.1_Uncultured_marine
AB679089.1_Uncultured_marine
AB727470.1_Uncultured_marine
AB727471.1_Uncultured_marine
AB727473.1_Uncultured_marine
AB727474.1_Uncultured_marine
AB727478.1_Uncultured_marine
AB727482.1_Uncultured_marine
AB727483.1_Uncultured_marine
AB727493.1_Uncultured_marine
AB727497.1_Uncultured_marine
AB727498.1_Uncultured_marine
AB727505.1_Uncultured_marine
AB827422.1_Uncultured_sewage

AB827423.1_Uncultured_sewage
AB827427.1_Uncultured_sewage
AB827438.1_Uncultured_sewage
AF013025.1_Microcoleus
AF016602.1_Unidentified_marine copepod
AF016603.1_Unidentified_marine copepod
AF016608.1_Unidentified_marine invertebrate
AF016609.1_Unidentified_marine invertebrate
AF016613.2_Unidentified_marine
AF016614.1_Unidentified_marine
AF016617.1_Unidentified_marine
AF016618.1_Unidentified_marine
AF035486.1_Uncultured_river
AF035491.1_Uncultured_river
AF035497.1_Uncultured_river
AF035500.1_Uncultured_river
AF035504.1_Uncultured_river
AF035506.1_Uncultured_river
AF035508.1_Uncultured_river
AF035509.1_Uncultured_river
AF035510.1_Uncultured_river
AF035518.1_Uncultured_river
AF035522.1_Uncultured_river
AF046827.1_Marine_microbial mat
AF046828.1_Marine_microbial mat
AF046829.1_Marine_microbial mat
AF046831.1_Marine_microbial mat
AF046832.1_Marine_microbial mat
AF046833.1_Marine_microbial mat
AF046835.1_Marine_microbial mat
AF046837.1_Marine_microbial mat
AF046841.1_Marine_microbial mat
AF046842.1_Marine_microbial mat
AF046845.1_Marine_microbial mat
AF046846.1_Marine_microbial mat
AF046847.1_Marine_microbial mat
AF046849.1_Marine_microbial mat
AF046850.1_Marine_microbial mat
AF046851.1_Marine_microbial mat
AF046853.1_Marine_microbial mat
AF046854.1_Marine_microbial mat
AF049036.1_Uncultured_ice
AF049037.1_Uncultured_ice
AF049038.1_Uncultured_ice

AF049039.1_Uncultured_ice
AF049040.1_Uncultured_ice
AF049043.1_Uncultured_ice
AF059621.1_Unidentified_marine
AF059622.1_Unidentified_marine
AF059623.1_Zehr_marine
AF059627.1_Unidentified_marine
AF059629.1_Unidentified_marine
AF059630.1_Unidentified_marine
AF059631.1_Unidentified_marine
AF059632.1_Unidentified_marine
AF059633.1_Unidentified_marine
AF059634.1_Unidentified_marine
AF059635.1_Unidentified_marine
AF059636.1_Unidentified_marine
AF059637.1_Unidentified_marine
AF059638.1_Unidentified_marine
AF059639.1_Unidentified_marine
AF059640.1_Unidentified_marine
AF059641.1_Unidentified_marine
AF059642.1_Unidentified_marine
AF059643.1_Unidentified_marine
AF059646.1_Unidentified_marine
AF059647.1_Unidentified_marine
AF059648.1_Marichromatium
AF059649.1_Marichromatium
AF082989.1_Vibrio
AF099781.1_Uncultured_soil
AF111110.2_Vibrio
AF134809.2_Vibrio
AF212874.1_Uncultured_freshwater lake
AF212878.1_Uncultured_freshwater lake
AF212879.1_Uncultured_freshwater lake
AF212887.1_Uncultured_freshwater lake
AF212888.1_Uncultured_freshwater lake
AF216875.1_Unidentified_marine rhizosphere
AF216876.1_Unidentified_marine rhizosphere
AF216877.1_Nitrogen-fixing_marine rhizosphere
AF216878.1_Nitrogen-fixing_marine rhizosphere
AF216880.1_Nitrogen-fixing_marine rhizosphere
AF216883.1_Azomonas
AF216884.1_Unidentified_marine rhizosphere
AF216885.1_Unidentified_marine rhizosphere
AF216886.1_Unidentified_marine rhizosphere

AF216887.1_Unidentified_marine rhizosphere
AF216888.1_Unidentified_marine rhizosphere
AF216889.1_Unidentified_marine rhizosphere
AF216890.1_Unidentified_marine rhizosphere
AF216891.1_Unidentified_marine rhizosphere
AF216893.1_Unidentified_marine rhizosphere
AF216894.1_Unidentified_marine rhizosphere
AF216896.1_Unidentified_marine rhizosphere
AF216897.1_Unidentified_marine rhizosphere
AF216898.1_Unidentified_marine rhizosphere
AF216906.1_Unidentified_marine rhizosphere
AF216907.1_Unidentified_marine rhizosphere
AF216911.1_Unidentified_marine rhizosphere
AF216912.1_Unidentified_marine rhizosphere
AF216913.1_Unidentified_marine rhizosphere
AF216920.1_Unidentified_marine rhizosphere
AF216923.1_Unidentified_marine rhizosphere
AF216926.1_Unidentified_marine rhizosphere
AF216927.1_Unidentified_marine rhizosphere
AF216931.1_Unidentified_marine rhizosphere
AF216932.1_Unidentified_marine rhizosphere
AF216933.1_Unidentified_marine rhizosphere
AF216934.1_Unidentified_marine rhizosphere
AF216936.1_Unidentified_marine rhizosphere
AF216937.1_Unidentified_marine rhizosphere
AF216938.1_Unidentified_marine rhizosphere
AF227927.1_Phormidium
AF227933.1_Marine
AF286685.1_Uncultured_freshwater lake
AF286686.1_Uncultured_freshwater lake
AF286687.1_Uncultured_freshwater lake
AF286688.1_Uncultured_freshwater lake
AF286689.1_Uncultured_freshwater lake
AF286694.1_Uncultured_freshwater lake
AF286695.1_Uncultured_freshwater lake
AF299425.1_Unidentified_marine
AF315429.1_Uncultured_soil
AF315433.1_Uncultured_soil
AF331985.1_Uncultured_root
AF331986.1_Uncultured_root
AF331988.1_Uncultured_root
AF331990.1_Uncultured_root
AF331991.1_Uncultured_root
AF374339.1_Unidentified_marine sediments

AF378720.1_Methylosinus
AF389719.1_Uncultured_marine seagrass
AF389720.1_Uncultured_marine seagrass
AF389793.1_Uncultured_marine seagrass
AF389820.1_Uncultured_marine seagrass
AF389821.1_Uncultured_marine seagrass
AF389822.1_Uncultured_marine seagrass
AF414610.1_Uncultured_marine seagrass
AF414616.1_Uncultured_marine seagrass
AF414617.1_Uncultured_marine seagrass
AF414618.1_Uncultured_marine seagrass
AF414619.1_Uncultured_marine seagrass
AF414649.1_Uncultured_marine seagrass
AF414650.1_Uncultured_marine seagrass
AF414652.1_Uncultured_marine seagrass
AF414656.1_Uncultured_marine seagrass
AF414666.1_Uncultured_marine seagrass
AF414668.1_Uncultured_marine seagrass
AF414670.1_Uncultured_marine seagrass
AF484669.1_Methylococcus
AF484670.1_Methylococcus
AF484671.1_Methylococcus
AF484672.1_Methylomonas
AF484673.1_Methylomonas
AF484674.1_Methylomonas
AF484675.1_Methylobacter
AF484676.1_Methylobacter
AF484677.1_Methylobacter
AF484678.1_Methylobacter
AJ297529.2_Pseudomonas_root
AJ313263.1_Uncultured_root
AJ313304.1_Uncultured_root
AJ563955.1_Methylosinus
AJ563956.1_Methylosinus
AJ563957.1_Methylobacter
AJ716234.1_Uncultured_mine spoils
AJ716244.1_Uncultured_mine spoils
AJ716247.1_Uncultured_mine spoils
AJ716250.1_Uncultured_mine spoils
AJ716254.1_Uncultured_mine spoils
AJ716259.1_Uncultured_mine spoils
AJ716260.1_Uncultured_mine spoils
AJ716261.1_Uncultured_mine spoils
AJ716267.1_Uncultured_mine spoils

AJ716269.1_Uncultured_mine spoils
AJ716271.1_Uncultured_mine spoils
AJ716285.1_Uncultured_mine spoils
AJ716386.1_Uncultured_mine spoils
AJ716387.1_Uncultured_mine spoils
AJ716392.1_Uncultured_mine spoils
AJ716398.1_Uncultured_mine spoils
AJ716404.1_Uncultured_mine spoils
AJ716409.1_Uncultured_mine spoils
AJ716412.1_Uncultured_mine spoils
AJ716413.1_Uncultured_mine spoils
AJ716414.1_Uncultured_mine spoils
AJ716415.1_Uncultured_mine spoils
AJ716416.1_Uncultured_mine spoils
AJ716419.1_Uncultured_mine spoils
AJ716420.1_Uncultured_mine spoils
AJ716421.1_Uncultured_mine spoils
AJ716422.1_Uncultured_mine spoils
AJ716423.1_Uncultured_mine spoils
AJ716424.1_Uncultured_mine spoils
AJ871103.1_Uncultured_soil
AM110721.1_Methylocapsa_fungus
AM746512.1_Uncultured_root
AM746514.1_Uncultured_root
AM746515.1_Uncultured_root
AM746517.1_Uncultured_root
AM746518.1_Uncultured_root
AM746519.1_Uncultured_root
AM746520.1_Uncultured_root
AM746521.1_Uncultured_root
AM746524.1_Uncultured_root
AM746527.1_Uncultured_root
AM746528.1_Uncultured_root
AM746529.1_Uncultured_root
AM746530.1_Uncultured_root
AM746531.1_Uncultured_root
AM746536.1_Uncultured_root
AM746540.1_Uncultured_root
AM746542.1_Uncultured_root
AM746544.1_Uncultured_root
AM746547.1_Uncultured_root
AM746548.1_Uncultured_root
AM746550.1_Uncultured_root
AM746558.1_Uncultured_root

AM746563.1_Uncultured_root
AM746566.1_Uncultured_root
AM746568.1_Uncultured_root
AM746570.1_Uncultured_root
AM746573.1_Uncultured_root
AM746577.1_Uncultured_root
AM746579.1_Uncultured_root
AM746585.1_Uncultured_root
AM746586.1_Uncultured_root
AM746587.1_Uncultured_root
AM746590.1_Uncultured_root
AM746594.1_Uncultured_root
AM746609.1_Uncultured_root
AY040524.1_Uncultured_microbial mat
AY091875.1_Uncultured_salt marsh
AY091901.1_Uncultured_salt marsh
AY091910.1_Uncultured_salt marsh
AY098502.1_Uncultured_marine rhizosphere
AY098504.1_Uncultured_marine rhizosphere
AY098505.1_Uncultured_marine rhizosphere
AY098506.1_Uncultured_marine rhizosphere
AY098507.1_Uncultured_marine rhizosphere
AY115593.1_Lyngbya
AY120644.1_Uncultured_hydrothermal vent
AY120651.1_Uncultured_hydrothermal vent
AY120661.1_Uncultured_hydrothermal vent
AY137215.1_Uncultured_marine mat
AY159591.1_Uncultured_root
AY159592.1_Uncultured_root
AY159596.1_Uncultured_root
AY159597.1_Uncultured_root
AY159599.1_Uncultured_root
AY159601.1_Uncultured_root
AY180983.1_Uncultured_root
AY180986.1_Uncultured_root
AY180992.1_Uncultured_root
AY181004.1_Uncultured_root
AY181005.1_Uncultured_root
AY181006.1_Uncultured_root
AY181007.1_Uncultured_root
AY181008.1_Uncultured_root
AY181010.1_Uncultured_root
AY181013.1_Uncultured_root
AY181017.1_Uncultured_root

AY181018.1_Uncultured_root
AY181023.1_Uncultured_root
AY196364.1_Uncultured_soil
AY196412.1_Uncultured_soil
AY196413.1_Uncultured_soil
AY196414.1_Uncultured_soil
AY196415.1_Uncultured_soil
AY196416.1_Uncultured_soil
AY196417.1_Uncultured_soil
AY196418.1_Uncultured_soil
AY196419.1_Uncultured_soil
AY196420.1_Uncultured_soil
AY196421.1_Uncultured_soil
AY196428.1_Uncultured_soil
AY196439.1_Uncultured_soil
AY221759.1_Uncultured_saline lake
AY221761.1_Uncultured_saline lake
AY221768.1_Uncultured_saline lake
AY221770.1_Uncultured_saline lake
AY221771.1_Uncultured_saline lake
AY221772.1_Uncultured_saline lake
AY221774.1_Uncultured_saline lake
AY221777.1_Uncultured_saline lake
AY221778.1_Uncultured_saline lake
AY221779.1_Uncultured_saline lake
AY221784.1_Uncultured_saline lake
AY221789.1_Uncultured_saline lake
AY221809.1_Uncultured_saline lake
AY221816.1_Symploca
AY221827.1_Klebsiella
AY221828.1_Vibrio
AY223914.1_Uncultured_Chesapeake
AY223915.1_Uncultured_Chesapeake
AY223934.1_Uncultured_Chesapeake
AY223935.1_Uncultured_Chesapeake
AY223945.1_Uncultured_Chesapeake
AY223950.1_Uncultured_Chesapeake
AY223953.1_Uncultured_Chesapeake
AY223979.1_Uncultured_Chesapeake
AY224002.1_Uncultured_Chesapeake
AY224004.1_Uncultured_Chesapeake
AY224005.1_Uncultured_Chesapeake
AY224030.1_Uncultured_River
AY225105.1_PCRreagent

AY225106.1_PCRreagent
AY225107.1_PCRreagent
AY231505.1_Uncultured_root
AY231508.1_Uncultured_root
AY231511.1_Uncultured_root
AY231512.1_Uncultured_root
AY231513.1_Uncultured_root
AY231515.1_Uncultured_root
AY231516.1_Uncultured_root
AY231517.1_Uncultured_root
AY231518.1_Uncultured_root
AY231519.1_Uncultured_root
AY231520.1_Uncultured_root
AY231521.1_Uncultured_root
AY231522.1_Uncultured_root
AY231530.1_Uncultured_root
AY231531.1_Uncultured_root
AY231537.1_Uncultured_root
AY231538.1_Uncultured_root
AY231547.1_Uncultured_root
AY231572.1_Uncultured_root
AY231573.1_Uncultured_root
AY231576.1_Uncultured_root
AY231577.1_Uncultured_root
AY231581.1_Uncultured_root
AY232355.1_Uncultured_cyanobacterial mat
AY232368.1_Uncultured_cyanobacterial mat
AY232374.1_Uncultured_cyanobacterial mat
AY232381.1_Uncultured_cyanobacterial mat
AY232387.1_Uncultured_cyanobacterial mat
AY232388.1_Uncultured_cyanobacterial mat
AY232389.1_Uncultured_cyanobacterial mat
AY242355.1_Klebsiella
AY351672.1_Azotobacter
AY367396.1_Raoultella
AY424712.1_Uncultured_soil
AY424718.1_Uncultured_soil
AY526259.1_Uncultured_soil
AY526272.1_Uncultured_soil
AY526285.1_Uncultured_soil
AY544164.1_Delftia
AY644349.1_Azomonas
AY684103.1_Uncultured_soil
AY684104.1_Uncultured_soil

AY684105.1_Uncultured_soil
AY684106.1_Uncultured_soil
AY684107.1_Uncultured_soil
AY684108.1_Uncultured_soil
AY684109.1_Uncultured_soil
AY684110.1_Uncultured_soil
AY684111.1_Uncultured_soil
AY684112.1_Uncultured_soil
AY684113.1_Uncultured_soil
AY684114.1_Uncultured_soil
AY684115.1_Uncultured_soil
AY684116.1_Uncultured_soil
AY684119.1_Uncultured_soil
AY684121.1_Uncultured_soil
AY706889.1_Church_marine plankton
AY706890.1_Uncultured_marine plankton
AY706891.1_Uncultured_marine plankton
AY706892.1_Uncultured_marine plankton
AY706893.1_Uncultured_marine plankton
AY706894.1_Uncultured_marine plankton
AY706895.1_Uncultured_marine plankton
AY706896.1_Uncultured_marine plankton
AY706903.1_Uncultured_marine plankton
AY724121.2_Uncultured_soil
AY724144.2_Uncultured_soil
AY768664.1_Uncultured_root
AY768677.1_Uncultured_root
AY768679.1_Uncultured_root
AY768682.1_Uncultured_root
AY787538.1_Uncultured_wastewater
AY787539.1_Uncultured_wastewater
AY787541.1_Uncultured_wastewater
AY787548.1_Uncultured_wastewater
AY787552.1_Uncultured_wastewater
AY787559.1_Uncultured_wastewater
AY787561.1_Uncultured_wastewater
AY787566.1_Uncultured_wastewater
AY787576.1_Uncultured_wastewater
AY795610.1_Uncultured_soil
AY795611.1_Uncultured_soil
AY795612.1_Uncultured_soil
AY795613.1_Uncultured_soil
AY795614.1_Uncultured_soil
AY795616.1_Uncultured_soil

AY800134.1_Uncultured_marine
AY800135.1_Uncultured_marine
AY800136.1_Uncultured_marine
AY800137.1_Uncultured_marine
AY800138.1_Uncultured_marine
AY800139.1_Uncultured_marine
AY800140.1_Uncultured_marine
AY800141.1_Uncultured_marine
AY800142.1_Uncultured_marine
AY800143.1_Uncultured_marine
AY819559.1_Uncultured_soil
AY819561.1_Uncultured_soil
AY819562.1_Uncultured_soil
AY819563.1_Uncultured_soil
AY819566.1_Uncultured_soil
AY819567.1_Uncultured_soil
AY819568.1_Uncultured_soil
AY819570.1_Uncultured_soil
AY819574.1_Uncultured_soil
AY821847.1_Uncultured_marine
AY829667.1_Uncultured_soil
AY829668.1_Uncultured_soil
AY829671.1_Uncultured_soil
AY829702.1_Uncultured_soil
AY829707.1_Uncultured_soil
AY855090.1_Uncultured_root
AY855095.1_Uncultured_root
AY896304.1_Uncultured_marine
AY896306.1_Uncultured_marine
AY896308.1_Uncultured_marine
AY896311.1_Uncultured_marine
AY896312.1_Uncultured_marine
AY896314.1_Uncultured_marine
AY896315.1_Uncultured_marine
AY896320.1_Uncultured_marine
AY896329.1_Uncultured_marine
AY896330.1_Uncultured_marine
AY896334.1_Uncultured_marine
AY896354.1_Uncultured_marine
AY896355.1_Uncultured_marine
AY896356.1_Uncultured_marine
AY896357.1_Uncultured_marine
AY896358.1_Uncultured_marine
AY896359.1_Uncultured_marine

AY896360.1_Uncultured_marine
AY896361.1_Uncultured_marine
AY896369.1_Uncultured_marine
AY896370.1_Uncultured_marine
AY896371.1_GammaA_marine
AY896372.1_Uncultured_marine
AY896378.1_Uncultured_marine
AY896382.1_Uncultured_marine
AY896400.1_Uncultured_marine
AY896401.1_Uncultured_marine
AY896417.1_Uncultured_marine
AY896418.1_Uncultured_marine
AY896419.1_Uncultured_marine
AY896420.1_Uncultured_marine
AY896421.1_Uncultured_marine
AY896422.1_Uncultured_marine
AY896424.1_Uncultured_marine
AY896425.1_Uncultured_marine
AY896426.1_Uncultured_marine
AY896427.1_Uncultured_marine
AY896428.1_GammaP_marine
AY896430.1_Uncultured_marine
AY896431.1_Uncultured_marine
AY896432.1_Uncultured_marine
AY896433.1_Uncultured_marine
AY896434.1_Uncultured_marine
AY896435.1_Uncultured_marine
AY896436.1_Uncultured_marine
AY896437.1_Uncultured_marine
AY896438.1_Uncultured_marine
AY896439.1_Uncultured_marine
AY896440.1_Uncultured_marine
AY896456.1_Uncultured_marine
AY896464.1_Uncultured_marine
AY896465.1_Uncultured_marine
AY896466.1_Uncultured_marine
AY896467.1_Uncultured_marine
AY896468.1_Uncultured_marine
AY896469.1_Uncultured_marine
AY912544.1_Uncultured_soil
AY912545.1_Uncultured_soil
AY912560.1_Uncultured_soil
AY912625.1_Uncultured_soil
AY912627.1_Uncultured_soil

AY912668.1_Uncultured_soil
AY912878.1_Uncultured_soil
AY937260.1_Methylobacter
AY940052.1_Uncultured_seamount
AY940054.1_Uncultured_seamount
AY974262.1_Uncultured_marine
AY974263.1_Uncultured_marine
AY974264.1_Uncultured_marine
AY974265.1_Uncultured_marine
AY974266.1_Uncultured_marine
CP000304.1_Pseudomonas
CP001157.1_Azotobacter
CP001614.2_Teredinibacter
CP001616.1_Tolumonas_freshwater sediment
CP001842.1_Candidatus_marine
CP001965.1_Sideroxydans
CP001968.1_Denitrovibrio
CP002432.1_Desulfurispirillum
CP002622.1_Pseudomonas
CP002738.1_Methylomonas_marine
CP003154.1_Thiocystis_brackish
CP003218.1_Klebsiella
CP003242.1_Vibrio
CP003406.1_Rahnella
CP003683.1_Klebsiella
CP005094.1_Azotobacter
CP005095.1_Azotobacter
CP005991.1_Enterobacter
CP007031.1_Marichromatium
CP007215.2_Enterobacter_root
CP007268.1_Halorhodospira
CP008700.1_Klebsiella
CP008788.1_Klebsiella_swab
CP008841.1_Klebsiella_soil
DQ058415.1_Rhizobium
DQ062516.1_Uncultured_marine
DQ062517.1_Uncultured_marine
DQ062518.1_Uncultured_marine
DQ062522.1_Uncultured_marine
DQ062524.1_Uncultured_marine
DQ062525.1_Uncultured_marine
DQ062526.1_Uncultured_marine
DQ062532.1_Uncultured_marine
DQ062533.1_Uncultured_marine

DQ062535.1_Uncultured_marine
DQ062537.1_Uncultured_marine
DQ077981.1_Uncultured_marine sediment
DQ078009.1_Uncultured_marine sediment
DQ078016.1_Uncultured_marine sediment
DQ078027.1_Uncultured_marine sediment
DQ078033.1_Uncultured_marine sediment
DQ078037.1_Uncultured_marine sediment
DQ078038.1_Uncultured_marine sediment
DQ078039.1_Uncultured_marine sediment
DQ078041.1_Uncultured_marine sediment
DQ078042.1_Uncultured_marine sediment
DQ078047.1_Uncultured_marine sediment
DQ098162.1_Uncultured_marine
DQ098164.1_Uncultured_marine
DQ098167.1_Uncultured_marine
DQ098169.1_Uncultured_marine
DQ098172.1_Uncultured_marine
DQ098174.1_Uncultured_marine
DQ098177.1_Uncultured_marine
DQ098187.1_Uncultured_marine
DQ098192.1_Uncultured_marine
DQ098199.1_Uncultured_marine
DQ098202.1_Uncultured_marine
DQ098203.1_Uncultured_marine
DQ098206.1_Uncultured_marine
DQ098207.1_Uncultured_marine
DQ098210.1_Uncultured_marine
DQ098225.1_Uncultured_marine
DQ098232.1_Uncultured_marine
DQ098235.1_Uncultured_marine
DQ098246.1_Uncultured_marine
DQ098247.1_Uncultured_marine
DQ098250.1_Uncultured_marine
DQ098251.1_Uncultured_marine
DQ098253.1_Uncultured_marine
DQ098257.1_Uncultured_marine
DQ098258.1_Uncultured_marine
DQ098260.1_Uncultured_marine
DQ118197.1_Uncultured_marine
DQ118200.1_Uncultured_marine
DQ118206.1_Uncultured_marine
DQ118208.1_Uncultured_marine
DQ118209.1_Uncultured_marine

DQ118214.2_Uncultured_marine
DQ118218.1_Uncultured_marine
DQ118220.1_Uncultured_marine
DQ118222.1_Uncultured_marine
DQ118223.1_Uncultured_marine
DQ118229.1_Uncultured_marine
DQ118230.1_Uncultured_marine
DQ118232.1_Uncultured_marine
DQ118233.1_Uncultured_marine
DQ118234.1_Uncultured_marine
DQ118235.1_Uncultured_marine
DQ118236.2_Uncultured_marine
DQ118237.2_Uncultured_marine
DQ118238.2_Uncultured_marine
DQ140465.1_Uncultured_saline pond
DQ140562.1_Uncultured_saline pond
DQ140579.1_Uncultured_saline pond
DQ140589.1_Uncultured_saline pond
DQ140682.1_Uncultured_saline pond
DQ140704.1_Uncultured_saline pond
DQ142734.1_Uncultured_marine microbial mat
DQ142737.1_Uncultured_marine microbial mat
DQ142743.1_Uncultured_marine microbial mat
DQ142745.1_Uncultured_marine microbial mat
DQ176978.1_Uncultured_marine root
DQ176979.1_Uncultured_marine root
DQ176980.1_Uncultured_marine root
DQ176981.1_Uncultured_marine root
DQ176982.1_Uncultured_marine root
DQ176983.1_Uncultured_marine root
DQ176984.1_Uncultured_marine root
DQ176985.1_Uncultured_marine root
DQ176986.1_Uncultured_marine root
DQ176987.1_Uncultured_marine root
DQ176995.1_Uncultured_marine root
DQ176996.1_Uncultured_marine root
DQ177015.1_Uncultured_marine root
DQ177017.1_Uncultured_marine root
DQ177018.1_Uncultured_marine root
DQ177022.1_Uncultured_marine root
DQ177023.1_Uncultured_marine root
DQ177024.1_Uncultured_marine root
DQ177026.1_Uncultured_marine root
DQ177027.1_Uncultured_marine root

DQ177028.1_Uncultured_marine root
DQ177029.1_Uncultured_marine root
DQ177030.1_Uncultured_marine root
DQ177031.1_Uncultured_marine root
DQ177032.1_Uncultured_marine root
DQ177033.1_Uncultured_marine root
DQ177035.1_Uncultured_marine root
DQ224529.1_Uncultured_saline pond
DQ224638.1_Uncultured_saline pond
DQ269145.1_Uncultured_marine
DQ304819.1_Uncultured_root
DQ337969.1_Uncultured_cyanobacterial mat
DQ398382.1_Uncultured_freshwater lake
DQ398388.1_Uncultured_freshwater lake
DQ398402.1_Uncultured_freshwater lake
DQ398450.1_Uncultured_freshwater lake
DQ398459.1_Uncultured_freshwater lake
DQ398469.1_Uncultured_freshwater lake
DQ398483.1_Uncultured_freshwater lake
DQ398527.1_Uncultured_freshwater lake
DQ398532.1_Uncultured_freshwater lake
DQ398536.1_Uncultured_freshwater lake
DQ398551.1_Uncultured_freshwater lake
DQ402560.1_Uncultured_marine rhizosphere
DQ402585.1_Uncultured_marine rhizosphere
DQ402609.1_Uncultured_marine rhizosphere
DQ402624.1_Uncultured_marine rhizosphere
DQ402630.1_Uncultured_marine rhizosphere
DQ402634.1_Uncultured_marine rhizosphere
DQ402647.1_Uncultured_marine rhizosphere
DQ402649.1_Uncultured_marine rhizosphere
DQ402650.1_Uncultured_marine rhizosphere
DQ402651.1_Uncultured_marine rhizosphere
DQ402653.1_Uncultured_marine rhizosphere
DQ402660.1_Uncultured_marine rhizosphere
DQ402681.1_Uncultured_marine rhizosphere
DQ402699.1_Uncultured_marine rhizosphere
DQ402733.1_Uncultured_marine rhizosphere
DQ402735.1_Uncultured_marine rhizosphere
DQ402739.1_Uncultured_marine rhizosphere
DQ402756.1_Uncultured_marine rhizosphere
DQ402787.1_Uncultured_marine rhizosphere
DQ402795.1_Uncultured_marine rhizosphere
DQ402796.1_Uncultured_marine rhizosphere

DQ402836.1_Uncultured_marine rhizosphere
DQ402842.1_Uncultured_marine rhizosphere
DQ402848.1_Uncultured_marine rhizosphere
DQ402887.1_Uncultured_marine rhizosphere
DQ402927.1_Uncultured_marine rhizosphere
DQ402931.1_Uncultured_marine rhizosphere
DQ402933.1_Uncultured_marine
DQ404410.1_Uncultured_marine
DQ404413.1_Uncultured_marine
DQ404418.1_Uncultured_marine
DQ404419.1_Uncultured_marine
DQ404424.1_Uncultured_marine
DQ404427.1_Uncultured_marine
DQ404433.1_Uncultured_marine
DQ404434.1_Uncultured_marine
DQ404435.1_Uncultured_marine
DQ404436.1_Uncultured_marine
DQ404438.1_Uncultured_marine
DQ404439.1_Uncultured_marine
DQ423541.1_Uncultured_rhizosphere
DQ423543.1_Uncultured_rhizosphere
DQ423547.1_Uncultured_rhizosphere
DQ425339.1_Uncultured_stem
DQ425342.1_Uncultured_stem
DQ425366.1_Uncultured_stem
DQ425377.1_Uncultured_stem
DQ425379.1_Uncultured_stem
DQ425395.1_Uncultured_stem
DQ425408.1_Uncultured_stem
DQ425426.1_Uncultured_stem
DQ425431.1_Uncultured_stem
DQ425442.1_Uncultured_stem
DQ425446.1_Uncultured_stem
DQ425449.1_Uncultured_stem
DQ425480.1_Uncultured_stem
DQ425484.1_Uncultured_stem
DQ425489.1_Uncultured_stem
DQ425518.1_Uncultured_stem
DQ425525.1_Uncultured_stem
DQ425538.1_Uncultured_stem
DQ425565.1_Uncultured_stem
DQ425572.1_Uncultured_stem
DQ425585.1_Uncultured_stem
DQ425590.1_Uncultured_stem

DQ425595.1_Uncultured_stem
DQ425597.1_Uncultured_stem
DQ425604.1_Uncultured_stem
DQ425619.1_Uncultured_stem
DQ425634.1_Uncultured_stem
DQ425637.1_Uncultured_stem
DQ425650.1_Uncultured_stem
DQ425651.1_Uncultured_stem
DQ425657.1_Uncultured_stem
DQ425664.1_Uncultured_stem
DQ425668.1_Uncultured_stem
DQ425685.1_Uncultured_stem
DQ425697.1_Uncultured_stem
DQ425698.1_Uncultured_stem
DQ425699.1_Uncultured_stem
DQ425703.1_Uncultured_stem
DQ425713.1_Uncultured_stem
DQ425720.1_Uncultured_stem
DQ425723.1_Uncultured_stem
DQ425733.1_Uncultured_stem
DQ425985.1_Uncultured_stem
DQ425989.1_Uncultured_stem
DQ425992.1_Uncultured_stem
DQ426001.1_Uncultured_stem
DQ426002.1_Uncultured_stem
DQ426003.1_Uncultured_stem
DQ426004.1_Uncultured_stem
DQ426005.1_Uncultured_stem
DQ426006.1_Uncultured_stem
DQ426008.1_Uncultured_stem
DQ426009.1_Uncultured_stem
DQ426011.1_Uncultured_stem
DQ426016.1_Uncultured_stem
DQ426017.1_Uncultured_stem
DQ426024.1_Uncultured_stem
DQ426026.1_Uncultured_stem
DQ426028.1_Uncultured_stem
DQ426029.1_Uncultured_stem
DQ426030.1_Uncultured_stem
DQ426032.1_Uncultured_stem
DQ426038.1_Uncultured_stem
DQ426041.1_Uncultured_stem
DQ426042.1_Uncultured_stem
DQ426044.1_Uncultured_stem

DQ426046.1_Uncultured_stem
DQ426047.1_Uncultured_stem
DQ426048.1_Uncultured_stem
DQ426050.1_Uncultured_stem
DQ426053.1_Uncultured_stem
DQ426056.1_Uncultured_stem
DQ426059.1_Uncultured_stem
DQ426064.1_Uncultured_stem
DQ426065.1_Uncultured_stem
DQ426066.1_Uncultured_stem
DQ426067.1_Uncultured_stem
DQ426083.1_Uncultured_stem
DQ426084.1_Uncultured_stem
DQ426089.1_Uncultured_stem
DQ426097.1_Uncultured_stem
DQ426100.1_Uncultured_stem
DQ426101.1_Uncultured_stem
DQ426104.1_Uncultured_stem
DQ426115.1_Uncultured_stem
DQ426116.1_Uncultured_stem
DQ426118.1_Uncultured_stem
DQ426120.1_Uncultured_stem
DQ426123.1_Uncultured_stem
DQ426127.1_Uncultured_stem
DQ426131.1_Uncultured_stem
DQ426139.1_Uncultured_stem
DQ426260.1_Uncultured_stem
DQ426273.1_Uncultured_stem
DQ426315.1_Uncultured_stem
DQ426347.1_Uncultured_stem
DQ426357.1_Uncultured_stem
DQ426368.1_Uncultured_stem
DQ426399.1_Uncultured_stem
DQ431161.1_Raoultella
DQ480876.1_Uncultured_soil
DQ480885.1_Uncultured_soil
DQ480899.1_Uncultured_soil
DQ480910.1_Uncultured_soil
DQ480984.1_Uncultured_soil
DQ481264.1_Uncultured_marine plankton
DQ481265.1_Uncultured_marine plankton
DQ481266.1_Uncultured_marine plankton
DQ481267.1_Uncultured_marine plankton
DQ481268.1_Uncultured_marine plankton

DQ481269.1_Uncultured_marine plankton
DQ481270.1_Uncultured_marine plankton
DQ481271.1_Uncultured_marine plankton
DQ481272.1_Uncultured_marine plankton
DQ481273.1_Uncultured_marine plankton
DQ481274.1_Uncultured_marine plankton
DQ481275.1_Uncultured_marine plankton
DQ481276.1_Uncultured_marine plankton
DQ481277.1_Uncultured_marine plankton
DQ481278.1_Uncultured_marine plankton
DQ481279.1_Uncultured_marine plankton
DQ481280.1_Uncultured_marine plankton
DQ481281.1_Uncultured_marine plankton
DQ481282.1_Uncultured_marine plankton
DQ481283.1_Uncultured_marine plankton
DQ481284.1_Uncultured_marine plankton
DQ481285.1_Uncultured_marine plankton
DQ481286.1_Uncultured_marine plankton
DQ481287.1_Uncultured_marine plankton
DQ481288.1_Uncultured_marine plankton
DQ481289.1_Uncultured_marine plankton
DQ481313.1_Uncultured_marine plankton
DQ481314.1_Uncultured_marine plankton
DQ481319.1_Uncultured_marine plankton
DQ481320.1_Uncultured_marine plankton
DQ481322.1_Uncultured_marine plankton
DQ481323.1_Uncultured_marine plankton
DQ481325.1_Uncultured_marine plankton
DQ481326.1_Uncultured_marine plankton
DQ481327.1_Uncultured_marine plankton
DQ481328.1_Uncultured_marine plankton
DQ481329.1_Uncultured_marine plankton
DQ481330.1_Uncultured_marine plankton
DQ481331.1_Uncultured_marine plankton
DQ481333.1_Uncultured_marine plankton
DQ481334.1_Uncultured_marine plankton
DQ481335.1_Uncultured_marine plankton
DQ481336.1_Uncultured_marine plankton
DQ481370.1_Uncultured_marine plankton
DQ481383.1_Uncultured_marine plankton
DQ481384.1_Uncultured_marine plankton
DQ481392.1_Uncultured_marine plankton
DQ481393.1_Uncultured_marine plankton
DQ481395.1_Uncultured_marine plankton

DQ481418.1_Uncultured_marine plankton
DQ481422.1_Uncultured_marine plankton
DQ481423.1_Uncultured_marine plankton
DQ481424.1_Uncultured_marine plankton
DQ481425.1_Uncultured_marine plankton
DQ481426.1_Uncultured_marine plankton
DQ481427.1_Uncultured_marine plankton
DQ481430.1_Uncultured_marine plankton
DQ481431.1_Uncultured_marine plankton
DQ481432.1_Uncultured_marine plankton
DQ481434.1_Uncultured_marine plankton
DQ481435.1_Uncultured_marine plankton
DQ481436.1_Uncultured_marine plankton
DQ481438.1_Uncultured_marine plankton
DQ481439.1_Uncultured_marine plankton
DQ481440.1_Uncultured_marine plankton
DQ481441.1_Uncultured_marine plankton
DQ481442.1_Uncultured_marine plankton
DQ481443.1_Uncultured_marine plankton
DQ481444.1_Uncultured_marine plankton
DQ481445.1_Uncultured_marine plankton
DQ481446.1_Uncultured_marine plankton
DQ481447.1_Uncultured_marine plankton
DQ481448.1_Uncultured_marine plankton
DQ481449.1_Uncultured_marine plankton
DQ481450.1_Uncultured_marine plankton
DQ481451.1_Uncultured_marine plankton
DQ481452.1_Uncultured_marine plankton
DQ481453.1_Uncultured_marine plankton
DQ481454.1_Uncultured_marine plankton
DQ481455.1_Uncultured_marine plankton
DQ481456.1_Uncultured_marine plankton
DQ481457.1_Uncultured_marine plankton
DQ481458.1_Uncultured_marine plankton
DQ481459.1_Uncultured_marine plankton
DQ481461.1_Uncultured_marine plankton
DQ518583.1_Uncultured_marine
DQ518585.1_Uncultured_marine
DQ518586.1_Uncultured_marine
DQ518587.1_Uncultured_marine
DQ520345.1_Uncultured_soil
DQ520346.1_Uncultured_soil
DQ520348.1_Uncultured_soil
DQ520355.1_Uncultured_soil

DQ520356.1_Uncultured_soil
DQ520451.1_Uncultured_soil
DQ520453.1_Uncultured_soil
DQ520484.1_Uncultured_soil
DQ520496.1_Uncultured_soil
DQ520501.1_Uncultured_soil
DQ520527.1_Uncultured_soil
DQ776311.1_Uncultured_terrestrial rhizosphere
DQ776326.1_Uncultured_terrestrial rhizosphere
DQ776341.1_Uncultured_terrestrial rhizosphere
DQ776349.1_Uncultured_terrestrial rhizosphere
DQ776351.1_Uncultured_terrestrial rhizosphere
DQ776387.1_Uncultured_terrestrial rhizosphere
DQ776399.1_Uncultured_terrestrial rhizosphere
DQ776400.1_Uncultured_terrestrial rhizosphere
DQ776425.1_Uncultured_terrestrial rhizosphere
DQ776446.1_Uncultured_terrestrial rhizosphere
DQ776493.1_Uncultured_terrestrial rhizosphere
DQ776501.1_Uncultured_terrestrial rhizosphere
DQ776503.1_Uncultured_terrestrial rhizosphere
DQ776513.1_Uncultured_terrestrial rhizosphere
DQ776518.1_Uncultured_terrestrial rhizosphere
DQ776529.1_Uncultured_terrestrial rhizosphere
DQ776534.1_Uncultured_terrestrial rhizosphere
DQ776535.1_Uncultured_terrestrial rhizosphere
DQ776555.1_Uncultured_terrestrial rhizosphere
DQ776559.1_Uncultured_terrestrial rhizosphere
DQ776581.1_Uncultured_terrestrial rhizosphere
DQ776672.1_Uncultured_terrestrial rhizosphere
DQ776679.1_Uncultured_terrestrial rhizosphere
DQ776748.1_Uncultured_terrestrial rhizosphere
DQ789165.1_Uncultured_marine
DQ789166.1_Uncultured_marine
DQ821944.1_Uncultured_Chesapeake
DQ821948.1_Uncultured_marine
DQ821958.1_Uncultured_marine
DQ821960.1_Uncultured_marine
DQ821965.1_Uncultured_river
DQ825711.1_Uncultured_marine
DQ825712.1_Uncultured_marine
DQ825713.1_Uncultured_marine
DQ825716.1_Uncultured_marine
DQ825718.1_Uncultured_marine
DQ825721.1_Uncultured_marine

DQ825722.1_Uncultured_marine
DQ825723.1_Uncultured_marine
DQ825724.1_Uncultured_marine
DQ825725.1_Uncultured_marine
DQ825726.1_Uncultured_marine
DQ825727.1_Uncultured_marine
DQ825728.1_Uncultured_marine
DQ825729.1_Uncultured_marine
DQ825730.1_Uncultured_marine
DQ825735.1_Uncultured_marine
DQ825739.1_Uncultured_marine
DQ825742.1_Uncultured_marine
DQ825743.1_Uncultured_marine
DQ825747.1_Uncultured_marine
DQ825750.1_Uncultured_marine
DQ825752.1_Uncultured_marine
DQ831856.1_Uncultured_marine
DQ913881.1_Celerinatantimonas
DQ913882.1_Celerinatantimonas
DQ913883.1_Celerinatantimonas
DQ913884.1_Celerinatantimonas
DQ913885.1_Celerinatantimonas
DQ982000.1_Uncultured_root
DQ982001.1_Uncultured_root
DQ982018.1_Uncultured_root
DQ982019.1_Uncultured_root
DQ982026.1_Uncultured_root
DQ982055.1_Uncultured_root
DQ982126.1_Uncultured_root
DQ982127.1_Uncultured_root
DQ982128.1_Uncultured_root
DQ982129.1_Uncultured_root
DQ982130.1_Uncultured_root
DQ982131.1_Uncultured_root
DQ982134.1_Uncultured_root
DQ982135.1_Uncultured_root
DQ982384.2_Uncultured_root
DQ982389.2_Uncultured_root
DQ982391.2_Uncultured_root
DQ982396.1_Uncultured_root
DQ982399.2_Uncultured_root
DQ982404.1_Uncultured_root
DQ982406.1_Uncultured_root
DQ982408.1_Uncultured_root

DQ982425.1_Uncultured_root
DQ982428.1_Uncultured_root
DQ982430.1_Uncultured_root
DQ982433.2_Uncultured_root
DQ982435.1_Uncultured_root
DQ982438.1_Uncultured_root
DQ982443.2_Uncultured_root
DQ982484.1_Uncultured_root
DQ982719.1_Uncultured_soil
DQ982742.2_Uncultured_soil
DQ982752.2_Uncultured_soil
DQ982769.2_Uncultured_soil
DQ982791.1_Uncultured_soil
DQ982841.2_Uncultured_soil
DQ982842.2_Uncultured_soil
DQ982852.2_Uncultured_soil
DQ983054.2_Uncultured_soil
DQ983095.2_Uncultured_soil
DQ983150.2_Uncultured_soil
DQ983172.2_Uncultured_soil
DQ983180.2_Uncultured_soil
EF133785.1_Uncultured_marine sediment
EF133786.1_Uncultured_marine sediment
EF133787.1_Uncultured_marine sediment
EF133788.1_Uncultured_marine sediment
EF133789.1_Uncultured_marine sediment
EF133790.1_Uncultured_marine sediment
EF133791.1_Uncultured_marine sediment
EF133792.1_Uncultured_marine sediment
EF133793.1_Uncultured_marine sediment
EF133794.1_Uncultured_marine sediment
EF133796.1_Uncultured_marine sediment
EF133797.1_Uncultured_marine sediment
EF133798.1_Uncultured_marine sediment
EF133806.1_Uncultured_marine sediment
EF133811.1_Uncultured_marine sediment
EF133812.1_Uncultured_marine sediment
EF133813.1_Uncultured_marine sediment
EF133814.1_Uncultured_marine sediment
EF133817.1_Uncultured_marine sediment
EF133818.1_Uncultured_marine sediment
EF133819.1_Uncultured_marine sediment
EF133821.1_Uncultured_marine sediment
EF133822.1_Uncultured_marine sediment

EF159986.1_Uncultured_marine mat
EF160027.1_Uncultured_marine mat
EF160029.1_Uncultured_marine mat
EF174669.1_Uncultured_marine
EF174676.1_Uncultured_marine
EF174683.1_Uncultured_marine
EF174686.1_Uncultured_marine
EF174687.1_Uncultured_marine
EF174691.1_Uncultured_marine
EF174693.1_Uncultured_marine
EF174696.1_Uncultured_marine
EF174699.1_Uncultured_marine
EF174700.1_Uncultured_marine
EF174702.1_Uncultured_marine
EF174703.1_Uncultured_marine
EF174705.1_Uncultured_marine
EF174707.1_Uncultured_marine
EF174709.1_Uncultured_marine
EF174715.1_Uncultured_marine
EF174716.1_Uncultured_marine
EF174718.1_Uncultured_marine
EF174720.1_Uncultured_marine
EF174724.1_Uncultured_marine
EF174725.1_Uncultured_marine
EF174728.1_Uncultured_marine
EF174730.1_Uncultured_marine
EF174731.1_Uncultured_marine
EF174734.1_Uncultured_marine
EF174738.1_Uncultured_marine
EF174740.1_Uncultured_marine
EF174741.1_Uncultured_marine
EF174745.1_Uncultured_marine
EF174748.1_Uncultured_marine
EF174749.1_Uncultured_marine
EF174751.1_Uncultured_marine
EF174752.1_Uncultured_marine
EF174755.1_Uncultured_marine
EF174759.1_Uncultured_marine
EF174763.1_Uncultured_marine
EF174766.1_Uncultured_marine
EF174767.1_Uncultured_marine
EF174768.1_Uncultured_marine
EF174769.1_Uncultured_marine
EF174773.1_Uncultured_marine

EF174776.1_Uncultured_marine
EF174777.1_Uncultured_marine
EF174780.1_Uncultured_marine
EF174781.1_Uncultured_marine
EF174784.1_Uncultured_marine
EF174785.1_Uncultured_marine
EF174813.1_Uncultured_marine
EF174818.1_Uncultured_marine
EF174821.1_Uncultured_marine
EF174824.1_Uncultured_marine
EF174826.1_Uncultured_marine
EF174834.1_Uncultured_marine
EF174835.1_Uncultured_marine
EF174839.1_Uncultured_marine
EF174842.1_Uncultured_marine
EF174848.1_Uncultured_marine
EF174849.1_Uncultured_marine
EF174852.1_Uncultured_marine
EF174858.1_Uncultured_marine
EF174859.1_Uncultured_marine
EF174868.1_Uncultured_marine
EF174879.1_Uncultured_marine
EF174880.1_Uncultured_marine
EF174882.1_Uncultured_marine
EF174885.1_Uncultured_marine
EF174886.1_Uncultured_marine
EF178501.1_Uncultured_marine
EF185785.1_Uncultured_marine sediment
EF185787.1_Uncultured_marine sediment
EF185788.1_Uncultured_marine sediment
EF191079.1_Nitrogen-fixing_marine
EF196650.1_Uncultured_marine sediment
EF196651.1_Uncultured_marine sediment
EF196653.1_Uncultured_marine sediment
EF199926.1_Uncultured_marine sediment
EF199953.1_Ectothiorhodospira
EF199954.1_Ectothiorhodospira
EF199955.1_Ectothiorhodospira
EF199956.1_Ectothiorhodospira
EF199958.1_Thioalkalispira
EF202525.1_Thiorhodospira
EF203422.1_Vibrio
EF204556.1_Uncultured_marine
EF204560.1_Uncultured_marine

EF204561.1_Uncultured_marine
EF204562.1_Uncultured_marine
EF204563.1_Uncultured_marine
EF208162.1_Uncultured_soil
EF208174.1_Uncultured_soil
EF208175.1_Uncultured_soil
EF208176.1_Uncultured_soil
EF208177.1_Uncultured_soil
EF208178.1_Uncultured_soil
EF438102.1_Uncultured_marine
EF438108.1_Uncultured_marine
EF438112.1_Uncultured_marine
EF468423.1_Uncultured_marine
EF468424.1_Uncultured_marine
EF468435.1_Uncultured_marine
EF468452.1_Uncultured_marine
EF468456.1_Uncultured_marine
EF468459.1_Uncultured_marine
EF470528.1_Uncultured_marine
EF470529.1_Uncultured_marine
EF470533.1_Uncultured_marine
EF470537.1_Uncultured_marine
EF494084.1_Uncultured_marine sediment
EF494085.1_Uncultured_marine sediment
EF494086.1_Uncultured_marine sediment
EF494087.1_Uncultured_marine sediment
EF494088.1_Uncultured_marine sediment
EF494089.1_Uncultured_marine sediment
EF494090.1_Uncultured_marine sediment
EF521120.1_Uncultured_soil
EF554362.1_Vibrio_root
EF568414.1_Uncultured_Mediterranean
EF568415.1_Uncultured_Mediterranean
EF568416.1_Uncultured_Mediterranean
EF568418.1_Uncultured_Mediterranean
EF568419.1_Uncultured_Mediterranean
EF568420.1_Uncultured_Mediterranean
EF568421.1_Uncultured_Mediterranean
EF568423.1_Uncultured_Mediterranean
EF568426.1_Uncultured_Mediterranean
EF568432.1_Uncultured_Mediterranean
EF568435.1_Uncultured_Mediterranean
EF568439.1_Uncultured_Mediterranean
EF568440.1_Uncultured_Mediterranean

EF568445.1_Uncultured_Mediterranean
EF568515.1_Uncultured_Mediterranean
EF568521.1_Uncultured_Mediterranean
EF568522.1_Uncultured_Mediterranean
EF568526.1_Uncultured_Mediterranean
EF568530.1_Uncultured_Mediterranean
EF568545.1_Uncultured_Mediterranean
EF568547.1_Uncultured_Mediterranean
EF568550.1_Uncultured_Mediterranean
EF568555.1_Uncultured_Mediterranean
EF568556.1_Uncultured_Mediterranean
EF568559.1_Uncultured_Mediterranean
EF568560.1_Uncultured_Mediterranean
EF568562.1_Uncultured_Mediterranean
EF568563.1_Uncultured_Mediterranean
EF568566.1_Uncultured_Mediterranean
EF568567.1_Uncultured_Mediterranean
EF568568.1_Uncultured_Mediterranean
EF568569.1_Uncultured_Mediterranean
EF568570.1_Uncultured_Mediterranean
EF568571.1_Uncultured_Mediterranean
EF568572.1_Uncultured_Mediterranean
EF568573.1_Uncultured_Mediterranean
EF568575.1_Uncultured_Mediterranean
EF568576.1_Uncultured_Mediterranean
EF568577.1_Uncultured_Mediterranean
EF568578.1_Uncultured_Mediterranean
EF568579.1_Uncultured_Mediterranean
EF568580.1_Uncultured_Mediterranean
EF568581.1_Uncultured_Mediterranean
EF568582.1_Uncultured_Mediterranean
EF568583.1_Uncultured_Mediterranean
EF568584.1_Uncultured_Mediterranean
EF568585.1_Uncultured_Mediterranean
EF568586.1_Uncultured_Mediterranean
EF568587.1_Uncultured_Mediterranean
EF568588.1_Uncultured_Mediterranean
EF568589.1_Uncultured_Mediterranean
EF568590.1_Uncultured_Mediterranean
EF583570.2_Uncultured_soil
EF583571.1_Uncultured_soil
EF583574.1_Uncultured_soil
EF583579.1_Uncultured_soil
EF583596.1_Uncultured_soil

EF583598.1_Uncultured_soil
EF583601.1_Uncultured_soil
EF583604.1_Uncultured_soil
EF583605.1_Uncultured_soil
EF631834.1_Uncultured_marine
EF631851.1_Uncultured_marine
EF631855.1_Uncultured_marine
EF631898.1_Uncultured_marine
EF631899.1_Uncultured_marine
EF631900.1_Uncultured_marine
EF631901.1_Uncultured_marine
EF631902.1_Uncultured_marine
EF631903.1_Uncultured_marine
EF631904.1_Uncultured_marine
EF631905.1_Uncultured_marine
EF631906.1_Uncultured_marine
EF631907.1_Uncultured_marine
EF631908.1_Uncultured_marine
EF631909.1_Uncultured_marine
EF634050.1_Azotobacter_soil
EF634054.1_Azotobacter_soil
EF988338.1_Uncultured_soil
EF988354.1_Uncultured_soil
EF988358.1_Uncultured_soil
EF988360.1_Uncultured_soil
EF988361.1_Uncultured_soil
EF988362.1_Uncultured_soil
EF988366.1_Uncultured_soil
EF988367.1_Uncultured_soil
EF988368.1_Uncultured_soil
EF988369.1_Uncultured_soil
EF988379.1_Uncultured_soil
EF988425.1_Uncultured_soil
EF988427.1_Uncultured_soil
EF988612.1_Uncultured_soil
EF988615.1_Uncultured_soil
EF988617.1_Uncultured_soil
EF988619.1_Uncultured_soil
EU035272.1_Oceanimonas
EU035273.1_Paracoccus
EU035278.1_Aeromonas
EU047963.1_Uncultured_soil
EU048002.1_Uncultured_soil
EU048046.1_Uncultured_soil

EU048059.1_Uncultured_soil
EU052297.1_Uncultured_South China Sea
EU052318.1_Uncultured_South China Sea
EU052319.1_Uncultured_South China Sea
EU052322.1_Uncultured_South China Sea
EU052324.1_Uncultured_South China Sea
EU052326.1_Uncultured_South China Sea
EU052327.1_Uncultured_South China Sea
EU052329.1_Uncultured_South China Sea
EU052333.1_Uncultured_South China Sea
EU052334.1_Uncultured_South China Sea
EU052335.1_Uncultured_South China Sea
EU052337.1_Uncultured_South China Sea
EU052339.1_Uncultured_South China Sea
EU052342.1_Uncultured_South China Sea
EU052343.1_Uncultured_South China Sea
EU052350.1_Uncultured_South China Sea
EU052380.1_Uncultured_South China Sea
EU052395.1_Uncultured_South China Sea
EU052396.1_Uncultured_South China Sea
EU052405.1_Uncultured_South China Sea
EU052406.1_Uncultured_South China Sea
EU052407.1_Uncultured_South China Sea
EU052408.1_Uncultured_South China Sea
EU052409.1_Uncultured_South China Sea
EU052411.1_Uncultured_South China Sea
EU052412.1_Uncultured_South China Sea
EU052413.1_Moisander_South China Sea
EU052415.1_Uncultured_South China Sea
EU052438.1_Uncultured_South China Sea
EU052524.1_Uncultured_South China Sea
EU052526.1_Uncultured_South China Sea
EU052529.1_Uncultured_South China Sea
EU052532.1_Uncultured_South China Sea
EU052537.1_Uncultured_South China Sea
EU052538.1_Uncultured_South China Sea
EU052543.1_Uncultured_South China Sea
EU052545.1_Uncultured_South China Sea
EU052549.1_Uncultured_South China Sea
EU052550.1_Uncultured_South China Sea
EU052555.1_Uncultured_South China Sea
EU052568.1_Uncultured_South China Sea
EU052577.1_Uncultured_South China Sea
EU052578.1_Uncultured_South China Sea

EU052579.1_Uncultured_South China Sea
EU052580.1_Uncultured_South China Sea
EU052588.1_Uncultured_South China Sea
EU052591.1_Uncultured_South China Sea
EU052592.1_Uncultured_South China Sea
EU052593.1_Uncultured_South China Sea
EU052611.1_Uncultured_South China Sea
EU052625.1_Uncultured_South China Sea
EU052652.1_Uncultured_South China Sea
EU052656.1_Uncultured_South China Sea
EU052659.1_Uncultured_South China Sea
EU052660.1_Uncultured_South China Sea
EU052666.1_Uncultured_South China Sea
EU090271.1_Uncultured_soil
EU097078.1_Uncultured_coastal
EU151773.1_Uncultured_marine
EU151774.1_Uncultured_marine
EU151775.1_Uncultured_marine
EU151780.1_Uncultured_marine
EU151782.1_Uncultured_marine
EU151783.1_Uncultured_marine
EU151785.1_Uncultured_marine
EU151788.1_Uncultured_marine
EU151796.1_Uncultured_marine
EU151863.1_Uncultured_marine
EU159532.1_Uncultured_marine
EU159533.1_Uncultured_marine
EU159534.1_Uncultured_marine
EU159535.1_Uncultured_marine
EU159536.1_Uncultured_marine
EU159537.1_Uncultured_marine
EU159538.1_Uncultured_marine
EU159539.1_Uncultured_marine
EU159540.1_Uncultured_marine
EU159541.1_Uncultured_marine
EU159542.1_Uncultured_marine
EU159543.1_Uncultured_marine
EU159544.1_Uncultured_marine
EU159546.1_Uncultured_marine
EU159547.1_Uncultured_marine
EU159548.1_Uncultured_marine
EU159549.1_Uncultured_marine
EU159550.1_Uncultured_marine
EU159551.1_Uncultured_marine

EU159552.1_Uncultured_marine
EU159553.1_Uncultured_marine
EU187507.1_Uncultured_marine
EU187510.1_Uncultured_marine
EU187511.1_Uncultured_marine
EU187512.1_Uncultured_marine
EU187515.1_Uncultured_marine
EU187519.1_Uncultured_marine
EU187524.1_Uncultured_marine
EU187531.1_Uncultured_marine
EU187533.1_Uncultured_marine
EU187538.1_Uncultured_marine
EU187543.1_Uncultured_marine
EU187544.1_Uncultured_marine
EU187546.1_Uncultured_marine
EU187549.1_Uncultured_marine
EU187567.1_Uncultured_marine
EU187568.1_Uncultured_marine
EU187572.1_Uncultured_marine
EU305261.1_Uncultured_glacier
EU305266.1_Uncultured_glacier
EU305273.1_Uncultured_glacier
EU305282.1_Uncultured_glacier
EU305285.1_Uncultured_glacier
EU305288.1_Uncultured_glacier
EU305291.1_Uncultured_glacier
EU331502.1_Uncultured_soil
EU331503.1_Uncultured_soil
EU331505.1_Uncultured_soil
EU331506.1_Uncultured_soil
EU331507.1_Uncultured_soil
EU331514.1_Uncultured_soil
EU331516.1_Uncultured_soil
EU331522.1_Uncultured_soil
EU331526.1_Uncultured_soil
EU331527.1_Uncultured_soil
EU331528.1_Uncultured_soil
EU331529.1_Uncultured_soil
EU331530.1_Uncultured_soil
EU331531.1_Uncultured_soil
EU331545.1_Uncultured_soil
EU331546.1_Uncultured_soil
EU331547.1_Uncultured_soil
EU331548.1_Uncultured_soil

EU331549.1_Uncultured_soil
EU331562.1_Uncultured_soil
EU381320.1_Uncultured_sediment
EU381321.1_Uncultured_sediment
EU381380.1_Uncultured_water
EU544214.1_Uncultured_soil
EU594013.1_Uncultured_marine sponge
EU594014.1_Uncultured_marine sponge
EU594015.1_Uncultured_marine sponge
EU594016.1_Uncultured_marine sponge
EU594017.1_Uncultured_marine sponge
EU594018.1_Uncultured_marine sponge
EU594020.1_Uncultured_marine sponge
EU594022.1_Uncultured_marine sponge
EU594023.1_Uncultured_marine sponge
EU594024.1_Uncultured_marine sponge
EU594025.1_Uncultured_marine sponge
EU594026.1_Uncultured_marine sponge
EU594027.1_Uncultured_marine sponge
EU594029.1_Uncultured_marine sponge
EU594031.1_Uncultured_marine sponge
EU594032.1_Uncultured_marine sponge
EU594033.1_Uncultured_marine sponge
EU594034.1_Uncultured_marine sponge
EU594035.1_Uncultured_marine sponge
EU594036.1_Uncultured_marine sponge
EU594037.1_Uncultured_marine sponge
EU594038.1_Uncultured_marine sponge
EU594055.1_Uncultured_marine sponge
EU594059.1_Uncultured_marine sponge
EU594060.1_Uncultured_marine sponge
EU594061.1_Uncultured_marine sponge
EU594065.1_Uncultured_marine sponge
EU594066.1_Uncultured_marine sponge
EU594085.1_Uncultured_marine sponge
EU594088.1_Uncultured_marine sponge
EU594091.1_Uncultured_marine sponge
EU594093.1_Uncultured_marine sponge
EU622783.1_Thiocapsa
EU622784.1_Thiocapsa
EU622788.1_Allochromatium
EU626505.1_Uncultured_soil
EU672874.1_Methylogaea_soil
EU693338.1_Azotobacter

EU693339.1_Bacillus
EU693341.1_Acinetobacter_soil
EU693378.1_Uncultured_coral
EU693389.1_Uncultured_coral
EU693392.1_Uncultured_coral
EU693393.1_Uncultured_coral
EU693395.1_Uncultured_coral
EU693397.1_Uncultured_coral
EU693400.1_Uncultured_coral
EU693406.1_Uncultured_coral
EU693407.1_Uncultured_coral
EU693411.1_Uncultured_coral
EU693413.1_Uncultured_coral
EU693417.1_Uncultured_coral
EU693418.1_Uncultured_coral
EU693419.1_Uncultured_coral
EU693420.1_Uncultured_coral
EU693421.1_Uncultured_coral
EU693422.1_Uncultured_coral
EU693423.1_Uncultured_coral
EU693424.1_Uncultured_coral
EU693425.1_Uncultured_coral
EU693426.1_Uncultured_coral
EU693427.1_Uncultured_coral
EU693429.1_Uncultured_coral
EU693430.1_Uncultured_coral
EU693431.1_Uncultured_coral
EU693433.1_Uncultured_coral
EU693437.1_Uncultured_coral
EU693438.1_Uncultured_coral
EU693439.1_Uncultured_coral
EU693441.1_Uncultured_coral
EU856741.1_Uncultured_coastal
EU912940.1_Uncultured_soil
EU912967.1_Uncultured_soil
EU912973.1_Uncultured_soil
EU913005.1_Uncultured_soil
EU913009.1_Uncultured_soil
EU913035.1_Uncultured_soil
EU913087.1_Uncultured_soil
EU915049.1_Uncultured_microbial mat
EU915050.1_Uncultured_microbial mat
EU915051.1_Uncultured_microbial mat
EU915056.1_Uncultured_microbial mat

EU916280.1_Uncultured_Baltic
EU916281.1_Uncultured_Baltic
EU916285.1_Uncultured_Baltic
EU916286.1_Uncultured_Baltic
EU916287.1_Uncultured_Baltic
EU916294.1_Uncultured_Baltic
EU916301.1_Uncultured_Baltic
EU916306.1_Uncultured_Baltic
EU916316.1_Uncultured_Baltic
EU916323.1_Uncultured_Baltic
EU916326.1_Uncultured_Baltic
EU916327.1_Uncultured_Baltic
EU916329.1_Uncultured_Baltic
EU916332.1_Uncultured_Baltic
EU916423.1_Uncultured_Baltic
EU916425.1_Uncultured_Baltic
EU916431.1_Uncultured_Baltic
EU916440.1_Uncultured_Baltic
EU916444.1_Uncultured_Baltic
EU916454.1_Uncultured_Baltic
EU916456.1_Uncultured_Baltic
EU938523.1_Erwinia
EU978409.1_Uncultured_marine
EU978418.1_Uncultured_marine
FJ008256.1_Uncultured_soil
FJ008294.1_Uncultured_soil
FJ008385.1_Uncultured_soil
FJ008391.1_Uncultured_soil
FJ008442.1_Uncultured_soil
FJ008459.1_Uncultured_soil
FJ008474.1_Uncultured_soil
FJ008489.1_Uncultured_soil
FJ008502.1_Uncultured_soil
FJ008512.1_Uncultured_soil
FJ008517.1_Uncultured_soil
FJ008575.1_Uncultured_soil
FJ008578.1_Uncultured_soil
FJ230093.1_Uncultured_South China Sea
FJ230095.1_Uncultured_South China Sea
FJ230098.1_Uncultured_South China Sea
FJ230099.1_Uncultured_South China Sea
FJ230142.1_Uncultured_South China Sea
FJ230143.1_Uncultured_South China Sea
FJ230146.1_Uncultured_South China Sea

FJ230155.1_Uncultured_South China Sea
FJ230156.1_Uncultured_South China Sea
FJ230158.1_Uncultured_South China Sea
FJ230159.1_Uncultured_South China Sea
FJ230160.1_Uncultured_South China Sea
FJ230161.1_Uncultured_South China Sea
FJ230162.1_Uncultured_South China Sea
FJ230163.1_Uncultured_South China Sea
FJ230164.1_Uncultured_South China Sea
FJ230166.1_Uncultured_South China Sea
FJ230169.1_Uncultured_South China Sea
FJ230176.1_Uncultured_South China Sea
FJ230189.1_Uncultured_South China Sea
FJ230191.1_Uncultured_South China Sea
FJ230195.1_Uncultured_South China Sea
FJ230196.1_Uncultured_South China Sea
FJ230197.1_Uncultured_South China Sea
FJ230201.1_Uncultured_South China Sea
FJ230203.1_Uncultured_South China Sea
FJ230204.1_Uncultured_South China Sea
FJ381621.1_Uncultured_soil
FJ381622.1_Uncultured_soil
FJ381624.1_Uncultured_soil
FJ381638.1_Uncultured_soil
FJ394926.1_Uncultured_rhizosphere
FJ394927.1_Uncultured_rhizosphere
FJ394928.1_Uncultured_rhizosphere
FJ394929.1_Uncultured_rhizosphere
FJ394930.1_Uncultured_rhizosphere
FJ394931.1_Uncultured_rhizosphere
FJ394932.1_Uncultured_rhizosphere
FJ394933.1_Uncultured_rhizosphere
FJ394934.1_Uncultured_rhizosphere
FJ394935.1_Uncultured_rhizosphere
FJ394936.1_Uncultured_rhizosphere
FJ394937.1_Uncultured_rhizosphere
FJ394938.1_Uncultured_rhizosphere
FJ394939.1_Uncultured_rhizosphere
FJ394940.1_Uncultured_rhizosphere
FJ394941.1_Uncultured_rhizosphere
FJ394942.1_Uncultured_rhizosphere
FJ394944.1_Uncultured_rhizosphere
FJ394945.1_Uncultured_rhizosphere
FJ394946.1_Uncultured_rhizosphere

FJ394947.1_Uncultured_rhizosphere
FJ394948.1_Uncultured_rhizosphere
FJ394949.1_Uncultured_rhizosphere
FJ394950.1_Uncultured_rhizosphere
FJ394951.1_Uncultured_rhizosphere
FJ394952.1_Uncultured_rhizosphere
FJ394953.1_Uncultured_rhizosphere
FJ394955.1_Uncultured_rhizosphere
FJ394956.1_Uncultured_rhizosphere
FJ394957.1_Uncultured_rhizosphere
FJ394958.1_Uncultured_rhizosphere
FJ394959.1_Uncultured_rhizosphere
FJ394960.1_Uncultured_rhizosphere
FJ394961.1_Uncultured_rhizosphere
FJ394963.1_Uncultured_rhizosphere
FJ394966.1_Uncultured_rhizosphere
FJ394967.1_Uncultured_rhizosphere
FJ394968.1_Uncultured_rhizosphere
FJ394969.1_Uncultured_rhizosphere
FJ394970.1_Uncultured_rhizosphere
FJ394971.1_Uncultured_rhizosphere
FJ394972.1_Uncultured_rhizosphere
FJ394973.1_Uncultured_rhizosphere
FJ394974.1_Uncultured_rhizosphere
FJ394975.1_Uncultured_rhizosphere
FJ394976.1_Uncultured_rhizosphere
FJ394977.1_Uncultured_rhizosphere
FJ394978.1_Uncultured_rhizosphere
FJ394979.1_Uncultured_rhizosphere
FJ394980.1_Uncultured_rhizosphere
FJ394981.1_Uncultured_rhizosphere
FJ394982.1_Uncultured_rhizosphere
FJ394983.1_Uncultured_rhizosphere
FJ394984.1_Uncultured_rhizosphere
FJ394985.1_Uncultured_rhizosphere
FJ394991.1_Uncultured_rhizosphere
FJ394992.1_Uncultured_rhizosphere
FJ394993.1_Uncultured_rhizosphere
FJ394994.1_Uncultured_rhizosphere
FJ394995.1_Uncultured_rhizosphere
FJ394996.1_Uncultured_rhizosphere
FJ394997.1_Uncultured_rhizosphere
FJ394999.1_Uncultured_rhizosphere
FJ395000.1_Uncultured_rhizosphere

FJ395001.1_Uncultured_rhizosphere
FJ395003.1_Uncultured_rhizosphere
FJ395004.1_Uncultured_rhizosphere
FJ395005.1_Uncultured_rhizosphere
FJ395006.1_Uncultured_rhizosphere
FJ395007.1_Uncultured_rhizosphere
FJ395011.1_Uncultured_rhizosphere
FJ395014.1_Uncultured_rhizosphere
FJ395015.1_Uncultured_rhizosphere
FJ395016.1_Uncultured_rhizosphere
FJ395017.1_Uncultured_rhizosphere
FJ395018.1_Uncultured_rhizosphere
FJ395019.1_Uncultured_rhizosphere
FJ395020.1_Uncultured_rhizosphere
FJ395021.1_Uncultured_rhizosphere
FJ395022.1_Uncultured_rhizosphere
FJ395026.1_Uncultured_rhizosphere
FJ395027.1_Uncultured_rhizosphere
FJ395028.1_Uncultured_rhizosphere
FJ395029.1_Uncultured_rhizosphere
FJ395030.1_Uncultured_rhizosphere
FJ395031.1_Uncultured_rhizosphere
FJ436232.1_Uncultured_soil
FJ460304.1_Uncultured_freshwater
FJ469773.1_Uncultured_soil
FJ502283.1_Uncultured_meromictic lake
FJ502291.1_Uncultured_meromictic lake
FJ502292.1_Uncultured_meromictic lake
FJ502293.1_Uncultured_meromictic lake
FJ502300.1_Uncultured_meromictic lake
FJ502301.1_Uncultured_meromictic lake
FJ502305.1_Uncultured_meromictic lake
FJ502316.1_Uncultured_meromictic lake
FJ593756.1_Klebsiella
FJ593757.1_Klebsiella
FJ593758.1_Klebsiella
FJ593759.1_Klebsiella
FJ593760.1_Klebsiella
FJ593761.1_Klebsiella
FJ593762.1_Klebsiella
FJ593763.1_Klebsiella
FJ593764.1_Klebsiella
FJ593765.1_Klebsiella
FJ593766.1_Klebsiella

FJ593767.1_Klebsiella
FJ593768.1_Klebsiella
FJ593769.1_Klebsiella
FJ593770.1_Pantoea
FJ593771.1_Pantoea
FJ593772.1_Pantoea
FJ593773.1_Pantoea
FJ593774.1_Pantoea
FJ593775.1_Pantoea
FJ593776.1_Pantoea
FJ593777.1_Pantoea
FJ593778.1_Pantoea
FJ593779.1_Pantoea
FJ593780.1_Pantoea
FJ593860.1_Klebsiella
FJ593861.1_Klebsiella
FJ593862.1_Klebsiella
FJ593863.1_Klebsiella
FJ593864.1_Klebsiella
FJ593867.1_Klebsiella
FJ593868.1_Enterobacter
FJ593869.1_Pantoea
FJ609152.1_Uncultured_coastal
FJ609153.1_Uncultured_coastal
FJ609156.1_Uncultured_coastal
FJ609157.1_Uncultured_coastal
FJ609159.1_Uncultured_coastal
FJ609162.1_Uncultured_coastal
FJ609164.1_Uncultured_coastal
FJ609166.1_Uncultured_coastal
FJ609167.1_Uncultured_coastal
FJ669432.1_Uncultured_marine sediment
FJ669438.1_Uncultured_marine sediment
FJ669439.1_Uncultured_marine sediment
FJ669486.1_Uncultured_marine sediment
FJ686490.1_Uncultured_marine sediment
FJ686492.1_Uncultured_marine sediment
FJ686494.1_Uncultured_marine sediment
FJ686499.1_Uncultured_marine sediment
FJ686501.1_Uncultured_marine sediment
FJ686503.1_Uncultured_marine sediment
FJ686505.1_Uncultured_marine sediment
FJ686506.1_Uncultured_marine sediment
FJ686509.1_Uncultured_marine sediment

FJ686510.1_Uncultured_marine sediment
FJ686511.1_Uncultured_marine sediment
FJ686514.1_Uncultured_marine sediment
FJ686515.1_Uncultured_marine sediment
FJ686522.1_Uncultured_marine sediment
FJ686524.1_Uncultured_marine sediment
FJ686528.1_Uncultured_marine sediment
FJ687518.1_Pseudomonas_wastewater
FJ687522.1_Aeromonas_wastewater
FJ756578.1_Uncultured_marine
FJ756579.1_Uncultured_marine
FJ756585.1_Uncultured_marine
FJ756595.1_Uncultured_marine
FJ756599.1_Uncultured_marine
FJ756602.1_Uncultured_marine
FJ756605.1_Uncultured_marine
FJ756606.1_Uncultured_marine
FJ756609.1_Uncultured_marine
FJ756610.1_Uncultured_marine
FJ756613.1_Uncultured_marine
FJ756618.1_Uncultured_marine
FJ756622.1_Uncultured_marine
FJ756624.1_Uncultured_marine
FJ756626.1_Uncultured_marine
FJ756627.1_Uncultured_marine
FJ756628.1_Uncultured_marine
FJ756633.1_Uncultured_marine
FJ756638.1_Uncultured_marine
FJ756639.1_Uncultured_marine
FJ756640.1_Uncultured_marine
FJ756641.1_Uncultured_marine
FJ756645.1_Uncultured_marine
FJ756647.1_Uncultured_marine
FJ756656.1_Uncultured_marine
FJ756659.1_Uncultured_marine
FJ756660.1_Uncultured_marine
FJ756666.1_Uncultured_marine
FJ756667.1_Uncultured_marine
FJ756672.1_Uncultured_marine
FJ756675.1_Uncultured_marine
FJ756687.1_Uncultured_marine
FJ756696.1_Uncultured_marine
FJ756698.1_Uncultured_marine
FJ756701.1_Uncultured_marine

FJ756712.1_Uncultured_marine
FJ756715.1_Uncultured_marine
FJ756722.1_Uncultured_marine
FJ807369.1_Uncultured_marine root
FJ807381.1_Uncultured_marine root
FJ807385.1_Uncultured_marine root
FJ949524.1_Uncultured_Baltic
FJ949526.1_Uncultured_Baltic
FJ949553.1_Uncultured_Baltic
FN555033.1_Uncultured_root
FN555044.1_Uncultured_root
FN555060.1_Uncultured_root
FN555081.1_Uncultured_root
FN557266.1_Uncultured_root
FN557283.1_Uncultured_root
FN557288.1_Uncultured_root
FN557292.1_Uncultured_root
FN649278.1_Uncultured_termite
FN665811.1_Uncultured_root
FN665897.1_Uncultured_root
FN665898.1_Uncultured_root
FN665899.1_Uncultured_root
FN665900.1_Uncultured_root
FN665902.1_Uncultured_root
FN665903.1_Uncultured_root
FN665904.1_Uncultured_root
FN665905.1_Uncultured_root
FN665906.1_Uncultured_root
FN665907.1_Uncultured_root
FN665908.1_Uncultured_root
FN665909.1_Uncultured_root
FN665910.1_Uncultured_root
FN665911.1_Uncultured_root
FN665913.1_Uncultured_root
FN665914.1_Uncultured_root
FN665915.1_Uncultured_root
FN665916.1_Uncultured_root
FN665917.1_Uncultured_root
FN813563.1_Pseudomonas_soil
FN813564.1_Pseudomonas_soil
FN813565.1_Pseudomonas_soil
FN813566.1_Pseudomonas_soil
FR669138.1_Pseudomonas_root
FR669139.1_Pseudomonas_root

FR669141.1_Pseudomonas_root
FR669144.1_Pseudomonas_root
FR669148.1_Pseudomonas_root
FR822663.1_Uncultured_soil
FR822664.1_Uncultured_soil
FR822665.1_Uncultured_soil
FR822669.1_Uncultured_soil
FR822670.1_Uncultured_soil
GQ241353.1_Rhizobium
GQ426251.1_Uncultured_hot spring
GQ426265.1_Uncultured_hot spring
GQ426268.1_Uncultured_hot spring
GQ426269.1_Uncultured_hot spring
GQ426272.1_Uncultured_hot spring
GQ441359.1_Uncultured_marine mat
GQ441368.1_Uncultured_marine mat
GQ441369.1_Uncultured_marine mat
GQ441376.1_Uncultured_marine mat
GQ441384.1_Uncultured_marine mat
GQ441392.1_Uncultured_marine mat
GQ441395.1_Uncultured_marine mat
GQ441396.1_Uncultured_marine mat
GQ441403.1_Uncultured_marine mat
GQ441410.1_Uncultured_marine mat
GQ441419.1_Uncultured_marine mat
GQ441443.1_Uncultured_marine mat
GQ441445.1_Uncultured_marine mat
GQ441450.1_Uncultured_marine mat
GQ441456.1_Uncultured_marine mat
GQ441462.1_Uncultured_marine mat
GQ441471.1_Uncultured_marine mat
GQ441477.1_Uncultured_marine mat
GQ441500.1_Uncultured_marine mat
GQ441537.1_Uncultured_marine mat
GQ441571.1_Uncultured_marine mat
GQ441595.1_Uncultured_marine mat
GQ441626.1_Uncultured_marine mat
GQ441663.1_Uncultured_marine mat
GQ441681.1_Uncultured_marine mat
GQ441696.1_Uncultured_marine mat
GQ441781.1_Uncultured_marine mat
GQ441789.1_Uncultured_marine mat
GQ441816.1_Uncultured_marine mat
GQ441825.1_Uncultured_marine mat

GQ441879.1_Uncultured_marine mat
GQ441923.1_Uncultured_marine mat
GQ441927.1_Uncultured_marine mat
GQ441937.1_Uncultured_marine mat
GQ441978.1_Uncultured_marine mat
GQ441986.1_Uncultured_marine mat
GQ442005.1_Uncultured_marine mat
GQ442013.1_Uncultured_marine mat
GQ442028.1_Uncultured_marine mat
GQ442034.1_Uncultured_marine mat
GQ442035.1_Uncultured_marine mat
GQ442045.1_Uncultured_marine mat
GQ442046.1_Uncultured_marine mat
GQ442047.1_Uncultured_marine mat
GQ442048.1_Uncultured_marine mat
GQ442066.1_Uncultured_marine mat
GQ442125.1_Uncultured_marine mat
GQ442132.1_Uncultured_marine mat
GQ442152.1_Uncultured_marine mat
GQ442159.1_Uncultured_marine mat
GQ442196.1_Uncultured_marine mat
GQ442229.1_Uncultured_marine mat
GQ442232.1_Uncultured_marine mat
GQ442252.1_Uncultured_marine mat
GQ442255.1_Uncultured_marine mat
GQ442262.1_Uncultured_marine mat
GQ442313.1_Uncultured_marine mat
GQ442315.1_Uncultured_marine mat
GQ442320.1_Uncultured_marine mat
GQ442325.1_Uncultured_marine mat
GQ442330.1_Uncultured_marine mat
GQ442331.1_Uncultured_marine mat
GQ442362.1_Uncultured_marine mat
GQ442364.1_Uncultured_marine mat
GQ442390.1_Uncultured_marine mat
GQ442393.1_Uncultured_marine mat
GQ442397.1_Uncultured_marine mat
GQ442446.1_Uncultured_marine mat
GQ442462.1_Uncultured_marine mat
GQ442477.1_Uncultured_marine mat
GQ442485.1_Uncultured_marine mat
GQ442495.1_Uncultured_marine mat
GQ442499.1_Uncultured_marine mat
GQ442508.1_Uncultured_marine mat

GQ442512.1_Uncultured_marine mat
GQ442513.1_Uncultured_marine mat
GQ442514.1_Uncultured_marine mat
GQ442516.1_Uncultured_marine mat
GQ442519.1_Uncultured_marine mat
GQ442523.1_Uncultured_marine mat
GQ442525.1_Uncultured_marine mat
GQ442526.1_Uncultured_marine mat
GQ442527.1_Uncultured_marine mat
GQ442535.1_Uncultured_marine mat
GQ442546.1_Uncultured_marine mat
GQ442548.1_Uncultured_marine mat
GQ442549.1_Uncultured_marine mat
GQ442550.1_Uncultured_marine mat
GQ442551.1_Uncultured_marine mat
GQ442552.1_Uncultured_marine mat
GQ442553.1_Uncultured_marine mat
GQ442556.1_Uncultured_marine mat
GQ442557.1_Uncultured_marine mat
GQ442558.1_Uncultured_marine mat
GQ442561.1_Uncultured_marine mat
GQ442564.1_Uncultured_marine mat
GQ442569.1_Uncultured_marine mat
GQ442570.1_Uncultured_marine mat
GQ442574.1_Uncultured_marine mat
GQ442585.1_Uncultured_marine mat
GQ442586.1_Uncultured_marine mat
GQ442587.1_Uncultured_marine mat
GQ442589.1_Uncultured_marine mat
GQ442590.1_Uncultured_marine mat
GQ442592.1_Uncultured_marine mat
GQ442596.1_Uncultured_marine mat
GQ442597.1_Uncultured_marine mat
GQ442600.1_Uncultured_marine mat
GQ442601.1_Uncultured_marine mat
GQ442602.1_Uncultured_marine mat
GQ442604.1_Uncultured_marine mat
GQ442606.1_Uncultured_marine mat
GQ442612.1_Uncultured_marine mat
GQ464084.1_Uncultured_fungus
GQ464085.1_Uncultured_fungus
GQ464086.1_Uncultured_fungus
GQ464087.1_Uncultured_fungus
GQ464088.1_Uncultured_fungus

GQ464089.1_Uncultured_fungus
GQ464090.1_Uncultured_fungus
GQ464094.1_Uncultured_fungus
GQ464095.1_Uncultured_fungus
GQ464096.1_Uncultured_fungus
GQ464097.1_Uncultured_fungus
GQ464098.1_Uncultured_fungus
GQ475428.1_Uncultured_South China Sea
GQ475431.1_Uncultured_South China Sea
GQ475433.1_Uncultured_South China Sea
GQ475438.1_Uncultured_South China Sea
GQ475440.1_Uncultured_South China Sea
GQ475444.1_Uncultured_South China Sea
GQ475446.1_Uncultured_South China Sea
GQ475447.1_Uncultured_South China Sea
GQ475448.1_Uncultured_South China Sea
GQ475450.1_Uncultured_South China Sea
GQ475453.1_Uncultured_South China Sea
GQ475455.1_Uncultured_South China Sea
GQ475468.1_Uncultured_South China Sea
GQ475472.1_Uncultured_South China Sea
GQ475475.1_Uncultured_South China Sea
GQ475476.1_Uncultured_South China Sea
GQ475477.1_Uncultured_South China Sea
GQ475478.1_Uncultured_South China Sea
GQ475479.1_Uncultured_South China Sea
GQ499204.1_Uncultured_marine sediment
GQ499212.1_Uncultured_marine sediment
GQ499220.1_Uncultured_marine sediment
GQ499223.1_Uncultured_marine sediment
GQ499227.1_Uncultured_marine sediment
GQ499232.1_Uncultured_marine sediment
GQ499250.1_Uncultured_marine sediment
GQ499263.1_Uncultured_marine sediment
GU097325.1_Uncultured_soil
GU097326.1_Uncultured_soil
GU097328.1_Uncultured_soil
GU097329.1_Uncultured_soil
GU097330.1_Uncultured_soil
GU097331.1_Uncultured_soil
GU097334.1_Uncultured_soil
GU097336.1_Uncultured_soil
GU097337.1_Uncultured_soil
GU097338.1_Uncultured_soil

GU097339.1_Uncultured_soil
GU097341.1_Uncultured_soil
GU097342.1_Uncultured_soil
GU097343.1_Uncultured_soil
GU097345.1_Uncultured_soil
GU097348.1_Uncultured_soil
GU097350.1_Uncultured_soil
GU097353.1_Uncultured_soil
GU111789.1_Uncultured_soil
GU111792.1_Uncultured_soil
GU117591.1_Uncultured_soil
GU117594.1_Uncultured_soil
GU117595.1_Uncultured_soil
GU121497.1_Uncultured_soil
GU121504.1_Uncultured_soil
GU192468.1_Uncultured_marine mat
GU192469.1_Uncultured_marine mat
GU192470.1_Uncultured_marine mat
GU192471.1_Uncultured_marine mat
GU192475.1_Uncultured_marine mat
GU192476.1_Uncultured_marine mat
GU192487.1_Uncultured_marine mat
GU192489.1_Uncultured_marine mat
GU192490.1_Uncultured_marine mat
GU192494.1_Uncultured_marine mat
GU192504.1_Uncultured_marine mat
GU192508.1_Uncultured_marine mat
GU192512.1_Uncultured_marine mat
GU192518.1_Uncultured_marine mat
GU192519.1_Uncultured_marine mat
GU192523.1_Uncultured_marine mat
GU192536.1_Uncultured_marine mat
GU192538.1_Uncultured_marine mat
GU192539.1_Uncultured_marine mat
GU192541.1_Uncultured_marine mat
GU192547.1_Uncultured_marine mat
GU192553.1_Uncultured_marine mat
GU192556.1_Uncultured_marine mat
GU192557.1_Uncultured_marine mat
GU192559.1_Uncultured_marine mat
GU192562.1_Uncultured_marine mat
GU192565.1_Uncultured_marine mat
GU192566.1_Uncultured_marine mat
GU192568.1_Uncultured_marine mat

GU192570.1_Uncultured_marine mat
GU192574.1_Uncultured_marine mat
GU192577.1_Uncultured_marine mat
GU192581.1_Uncultured_marine mat
GU192587.1_Uncultured_marine mat
GU192590.1_Uncultured_marine mat
GU192591.1_Uncultured_marine mat
GU192593.1_Uncultured_marine mat
GU192598.1_Uncultured_marine mat
GU192601.1_Uncultured_marine mat
GU192602.1_Uncultured_marine mat
GU192603.1_Uncultured_marine mat
GU192604.1_Uncultured_marine mat
GU192605.1_Uncultured_marine mat
GU192606.1_Uncultured_marine mat
GU192608.1_Uncultured_marine mat
GU192609.1_Uncultured_marine mat
GU192610.1_Uncultured_marine mat
GU192615.1_Uncultured_marine mat
GU192616.1_Uncultured_marine mat
GU192617.1_Uncultured_marine mat
GU192618.1_Uncultured_marine mat
GU192627.1_Uncultured_marine mat
GU192640.1_Uncultured_marine mat
GU192641.1_Uncultured_marine mat
GU192642.1_Uncultured_marine mat
GU192643.1_Uncultured_marine mat
GU192644.1_Uncultured_marine mat
GU192645.1_Uncultured_marine mat
GU192646.1_Uncultured_marine mat
GU192647.1_Uncultured_marine mat
GU192648.1_Uncultured_marine mat
GU192649.1_Uncultured_marine mat
GU192650.1_Uncultured_marine mat
GU192651.1_Uncultured_marine mat
GU192652.1_Uncultured_marine mat
GU192653.1_Uncultured_marine mat
GU192654.1_Uncultured_marine mat
GU192655.1_Uncultured_marine mat
GU192656.1_Uncultured_marine mat
GU192658.1_Uncultured_marine mat
GU192659.1_Uncultured_marine mat
GU192660.1_Uncultured_marine mat
GU192661.1_Uncultured_marine mat

GU192663.1_Uncultured_marine mat
GU192667.1_Uncultured_marine mat
GU192668.1_Uncultured_marine mat
GU192669.1_Uncultured_marine mat
GU192670.1_Uncultured_marine mat
GU192672.1_Uncultured_marine mat
GU192673.1_Uncultured_marine mat
GU192676.1_Uncultured_marine mat
GU192677.1_Uncultured_marine mat
GU192678.1_Uncultured_marine mat
GU192679.1_Uncultured_marine mat
GU192681.1_Uncultured_marine mat
GU192683.1_Uncultured_marine mat
GU192684.1_Uncultured_marine mat
GU192685.1_Uncultured_marine mat
GU192686.1_Uncultured_marine mat
GU192687.1_Uncultured_marine mat
GU192688.1_Uncultured_marine mat
GU192689.1_Uncultured_marine mat
GU192690.1_Uncultured_marine mat
GU192691.1_Uncultured_marine mat
GU192692.1_Uncultured_marine mat
GU192693.1_Uncultured_marine mat
GU192694.1_Uncultured_marine mat
GU192695.1_Uncultured_marine mat
GU192696.1_Uncultured_marine mat
GU192697.1_Uncultured_marine mat
GU192698.1_Uncultured_marine mat
GU192701.1_Uncultured_marine mat
GU192702.1_Uncultured_marine mat
GU192703.1_Uncultured_marine mat
GU192704.1_Uncultured_marine mat
GU192707.1_Uncultured_marine mat
GU192708.1_Uncultured_marine mat
GU192709.1_Uncultured_marine mat
GU192710.1_Uncultured_marine mat
GU192711.1_Uncultured_marine mat
GU192712.1_Uncultured_marine mat
GU192714.1_Uncultured_marine mat
GU192715.1_Uncultured_marine mat
GU192716.1_Uncultured_marine mat
GU192717.1_Uncultured_marine mat
GU192718.1_Uncultured_marine mat
GU192719.1_Uncultured_marine mat

GU192720.1_Uncultured_marine mat
GU192721.1_Uncultured_marine mat
GU192722.1_Uncultured_marine mat
GU192723.1_Uncultured_marine mat
GU192724.1_Uncultured_marine mat
GU192725.1_Uncultured_marine mat
GU192726.1_Uncultured_marine mat
GU192727.1_Uncultured_marine mat
GU192728.1_Uncultured_marine mat
GU192729.1_Uncultured_marine mat
GU192730.1_Uncultured_marine mat
GU192731.1_Uncultured_marine mat
GU192734.1_Uncultured_marine mat
GU192735.1_Uncultured_marine mat
GU192736.1_Uncultured_marine mat
GU192737.1_Uncultured_marine mat
GU192739.1_Uncultured_marine mat
GU192740.1_Uncultured_marine mat
GU192741.1_Uncultured_marine mat
GU192742.1_Uncultured_marine mat
GU192743.1_Uncultured_marine mat
GU192746.1_Uncultured_marine mat
GU192747.1_Uncultured_marine mat
GU192749.1_Uncultured_marine mat
GU192752.1_Uncultured_marine mat
GU192753.1_Uncultured_marine mat
GU192755.1_Uncultured_marine mat
GU192760.1_Uncultured_marine mat
GU192761.1_Uncultured_marine mat
GU192763.1_Uncultured_marine mat
GU192764.1_Uncultured_marine mat
GU192765.1_Uncultured_marine mat
GU192766.1_Uncultured_marine mat
GU192767.1_Uncultured_marine mat
GU192768.1_Uncultured_marine mat
GU192769.1_Uncultured_marine mat
GU192770.1_Uncultured_marine mat
GU192771.1_Uncultured_marine mat
GU192772.1_Uncultured_marine mat
GU192774.1_Uncultured_marine mat
GU192775.1_Uncultured_marine mat
GU192777.1_Uncultured_marine mat
GU192778.1_Uncultured_marine mat
GU192779.1_Uncultured_marine mat

GU192780.1_Uncultured_marine mat
GU192782.1_Uncultured_marine mat
GU192783.1_Uncultured_marine mat
GU192786.1_Uncultured_marine mat
GU192787.1_Uncultured_marine mat
GU192788.1_Uncultured_marine mat
GU192789.1_Uncultured_marine mat
GU192792.1_Uncultured_marine mat
GU192794.1_Uncultured_marine mat
GU192795.1_Uncultured_marine mat
GU192796.1_Uncultured_marine mat
GU192797.1_Uncultured_marine mat
GU192798.1_Uncultured_marine mat
GU192799.1_Uncultured_marine mat
GU192800.1_Uncultured_marine mat
GU192803.1_Uncultured_marine mat
GU192804.1_Uncultured_marine mat
GU192806.1_Uncultured_marine mat
GU192809.1_Uncultured_marine mat
GU192810.1_Uncultured_marine mat
GU192811.1_Uncultured_marine mat
GU192812.1_Uncultured_marine mat
GU192815.1_Uncultured_marine mat
GU192816.1_Uncultured_marine mat
GU192817.1_Uncultured_marine mat
GU192818.1_Uncultured_marine mat
GU192820.1_Uncultured_marine mat
GU192821.1_Uncultured_marine mat
GU192823.1_Uncultured_marine mat
GU192824.1_Uncultured_marine mat
GU192825.1_Uncultured_marine mat
GU192826.1_Uncultured_marine mat
GU192832.1_Uncultured_marine mat
GU192833.1_Uncultured_marine mat
GU192834.1_Uncultured_marine mat
GU192835.1_Uncultured_marine mat
GU192837.1_Uncultured_marine mat
GU192838.1_Uncultured_marine mat
GU192839.1_Uncultured_marine mat
GU192840.1_Uncultured_marine mat
GU192841.1_Uncultured_marine mat
GU192842.1_Uncultured_marine mat
GU192843.1_Uncultured_marine mat
GU192844.1_Uncultured_marine mat

GU192845.1_Uncultured_marine mat
GU192846.1_Uncultured_marine mat
GU192847.1_Uncultured_marine mat
GU192848.1_Uncultured_marine mat
GU192849.1_Uncultured_marine mat
GU192850.1_Uncultured_marine mat
GU192852.1_Uncultured_marine mat
GU192855.1_Uncultured_marine mat
GU192859.1_Uncultured_marine mat
GU192860.1_Uncultured_marine mat
GU192862.1_Uncultured_marine mat
GU192864.1_Uncultured_marine mat
GU192868.1_Uncultured_marine mat
GU192869.1_Uncultured_marine mat
GU192870.1_Uncultured_marine mat
GU192872.1_Uncultured_marine mat
GU192876.1_Uncultured_marine mat
GU192877.1_Uncultured_marine mat
GU192879.1_Uncultured_marine mat
GU192881.1_Uncultured_marine mat
GU192882.1_Uncultured_marine mat
GU192883.1_Uncultured_marine mat
GU192884.1_Uncultured_marine mat
GU192886.1_Uncultured_marine mat
GU192887.1_Uncultured_marine mat
GU192888.1_Uncultured_marine mat
GU192889.1_Uncultured_marine mat
GU192890.1_Uncultured_marine mat
GU192891.1_Uncultured_marine mat
GU192892.1_Uncultured_marine mat
GU192893.1_Uncultured_marine mat
GU192894.1_Uncultured_marine mat
GU192895.1_Uncultured_marine mat
GU192898.1_Uncultured_marine mat
GU192899.1_Uncultured_marine mat
GU192900.1_Uncultured_marine mat
GU192901.1_Uncultured_marine mat
GU192903.1_Uncultured_marine mat
GU192904.1_Uncultured_marine mat
GU192905.1_Uncultured_marine mat
GU192907.1_Uncultured_marine mat
GU192908.1_Uncultured_marine mat
GU192911.1_Uncultured_marine mat
GU192912.1_Uncultured_marine mat

GU192913.1_Uncultured_marine mat
GU192915.1_Uncultured_marine mat
GU192916.1_Uncultured_marine mat
GU192917.1_Uncultured_marine mat
GU192918.1_Uncultured_marine mat
GU192919.1_Uncultured_marine mat
GU192920.1_Uncultured_marine mat
GU192921.1_Uncultured_marine mat
GU192923.1_Uncultured_marine mat
GU192925.1_Uncultured_marine mat
GU192926.1_Uncultured_marine mat
GU192927.1_Uncultured_marine mat
GU192928.1_Uncultured_marine mat
GU192929.1_Uncultured_marine mat
GU192931.1_Uncultured_marine mat
GU192933.1_Uncultured_marine mat
GU192934.1_Uncultured_marine mat
GU192935.1_Uncultured_marine mat
GU192937.1_Uncultured_marine mat
GU192939.1_Uncultured_marine mat
GU192941.1_Uncultured_marine mat
GU192942.1_Uncultured_marine mat
GU192943.1_Uncultured_marine mat
GU192944.1_Uncultured_marine mat
GU192945.1_Uncultured_marine mat
GU192947.1_Uncultured_marine mat
GU192948.1_Uncultured_marine mat
GU192949.1_Uncultured_marine mat
GU192950.1_Uncultured_marine mat
GU192951.1_Uncultured_marine mat
GU192953.1_Uncultured_marine mat
GU192954.1_Uncultured_marine mat
GU192955.1_Uncultured_marine mat
GU192956.1_Uncultured_marine mat
GU192957.1_Uncultured_marine mat
GU192959.1_Uncultured_marine mat
GU192961.1_Uncultured_marine mat
GU192962.1_Uncultured_marine mat
GU192963.1_Uncultured_marine mat
GU192966.1_Uncultured_marine mat
GU192968.1_Uncultured_marine mat
GU192969.1_Uncultured_marine mat
GU192972.1_Uncultured_marine mat
GU192973.1_Uncultured_marine mat

GU192974.1_Uncultured_marine mat
GU192975.1_Uncultured_marine mat
GU192978.1_Uncultured_marine mat
GU192979.1_Uncultured_marine mat
GU192980.1_Uncultured_marine mat
GU192981.1_Uncultured_marine mat
GU192984.1_Uncultured_marine mat
GU192987.1_Uncultured_marine mat
GU192998.1_Uncultured_marine mat
GU193000.1_Uncultured_marine mat
GU193004.1_Uncultured_marine mat
GU193005.1_Uncultured_marine mat
GU193011.1_Uncultured_marine mat
GU193013.1_Uncultured_marine mat
GU193014.1_Uncultured_marine mat
GU193017.1_Uncultured_marine mat
GU193020.1_Uncultured_marine mat
GU193022.1_Uncultured_marine mat
GU193023.1_Uncultured_marine mat
GU193024.1_Uncultured_marine mat
GU193028.1_Uncultured_marine mat
GU193033.1_Uncultured_marine mat
GU193039.1_Uncultured_marine mat
GU193040.1_Uncultured_marine mat
GU193042.1_Uncultured_marine mat
GU193043.1_Uncultured_marine mat
GU193044.1_Uncultured_marine mat
GU193045.1_Uncultured_marine mat
GU193049.1_Uncultured_marine mat
GU193050.1_Uncultured_marine mat
GU193051.1_Uncultured_marine mat
GU193052.1_Uncultured_marine mat
GU193053.1_Uncultured_marine mat
GU193054.1_Uncultured_marine mat
GU193055.1_Uncultured_marine mat
GU193056.1_Uncultured_marine mat
GU193057.1_Uncultured_marine mat
GU193058.1_Uncultured_marine mat
GU193064.1_Uncultured_marine mat
GU193066.1_Uncultured_marine mat
GU193069.1_Uncultured_marine mat
GU193070.1_Uncultured_marine mat
GU193071.1_Uncultured_marine mat
GU193072.1_Uncultured_marine mat

GU193073.1_Uncultured_marine mat
GU193074.1_Uncultured_marine mat
GU193075.1_Uncultured_marine mat
GU193077.1_Uncultured_marine mat
GU193083.1_Uncultured_marine mat
GU193084.1_Uncultured_marine mat
GU193085.1_Uncultured_marine mat
GU193086.1_Uncultured_marine mat
GU193087.1_Uncultured_marine mat
GU193088.1_Uncultured_marine mat
GU193093.1_Uncultured_marine mat
GU193098.1_Uncultured_marine mat
GU193100.1_Uncultured_marine mat
GU193101.1_Uncultured_marine mat
GU193103.1_Uncultured_marine mat
GU193104.1_Uncultured_marine mat
GU193105.1_Uncultured_marine mat
GU193107.1_Uncultured_marine mat
GU193109.1_Uncultured_marine mat
GU193116.1_Uncultured_marine mat
GU193121.1_Uncultured_marine mat
GU193122.1_Uncultured_marine mat
GU193123.1_Uncultured_marine mat
GU193125.1_Uncultured_marine mat
GU193126.1_Uncultured_marine mat
GU193127.1_Uncultured_marine mat
GU193132.1_Uncultured_marine mat
GU193133.1_Uncultured_marine mat
GU193134.1_Uncultured_marine mat
GU193135.1_Uncultured_marine mat
GU193136.1_Uncultured_marine mat
GU193138.1_Uncultured_marine mat
GU193139.1_Uncultured_marine mat
GU193143.1_Uncultured_marine mat
GU193144.1_Uncultured_marine mat
GU193145.1_Uncultured_marine mat
GU193147.1_Uncultured_marine mat
GU193148.1_Uncultured_marine mat
GU193154.1_Uncultured_marine mat
GU193156.1_Uncultured_marine mat
GU193160.1_Uncultured_marine mat
GU193169.1_Uncultured_marine mat
GU193170.1_Uncultured_marine mat
GU193177.1_Uncultured_marine mat

GU193185.1_Uncultured_marine mat
GU193193.1_Uncultured_marine mat
GU193196.1_Uncultured_marine mat
GU193197.1_Uncultured_marine mat
GU193204.1_Uncultured_marine mat
GU193211.1_Uncultured_marine mat
GU193220.1_Uncultured_marine mat
GU193244.1_Uncultured_marine mat
GU193246.1_Uncultured_marine mat
GU193251.1_Uncultured_marine mat
GU193257.1_Uncultured_marine mat
GU193263.1_Uncultured_marine mat
GU193272.1_Uncultured_marine mat
GU193278.1_Uncultured_marine mat
GU193301.1_Uncultured_marine mat
GU193338.1_Uncultured_marine mat
GU193372.1_Uncultured_marine mat
GU193396.1_Uncultured_marine mat
GU193427.1_Uncultured_marine mat
GU193438.1_Uncultured_marine mat
GU193439.1_Uncultured_marine mat
GU193440.1_Uncultured_marine mat
GU193444.1_Uncultured_marine mat
GU193446.1_Uncultured_marine mat
GU193448.1_Uncultured_marine mat
GU193450.1_Uncultured_marine mat
GU193458.1_Uncultured_marine mat
GU193460.1_Uncultured_marine mat
GU193464.1_Uncultured_marine mat
GU193467.1_Uncultured_marine mat
GU193477.1_Uncultured_marine mat
GU193479.1_Uncultured_marine mat
GU193482.1_Uncultured_marine mat
GU193495.1_Uncultured_marine mat
GU193504.1_Uncultured_marine mat
GU193505.1_Uncultured_marine mat
GU193507.1_Uncultured_marine mat
GU193508.1_Uncultured_marine mat
GU193509.1_Uncultured_marine mat
GU193516.1_Uncultured_marine mat
GU193517.1_Uncultured_marine mat
GU193521.1_Uncultured_marine mat
GU193522.1_Uncultured_marine mat
GU193524.1_Uncultured_marine mat

GU193543.1_Uncultured_marine mat
GU193556.1_Uncultured_marine mat
GU193559.1_Uncultured_marine mat
GU193565.1_Uncultured_marine mat
GU193567.1_Uncultured_marine mat
GU193596.1_Uncultured_marine mat
GU193608.1_Uncultured_marine mat
GU193609.1_Uncultured_marine mat
GU193610.1_Uncultured_marine mat
GU193611.1_Uncultured_marine mat
GU193613.1_Uncultured_marine mat
GU193614.1_Uncultured_marine mat
GU193615.1_Uncultured_marine mat
GU193616.1_Uncultured_marine mat
GU193617.1_Uncultured_marine mat
GU193618.1_Uncultured_marine mat
GU193619.1_Uncultured_marine mat
GU193620.1_Uncultured_marine mat
GU193621.1_Uncultured_marine mat
GU193622.1_Uncultured_marine mat
GU193623.1_Uncultured_marine mat
GU193624.1_Uncultured_marine mat
GU193625.1_Uncultured_marine mat
GU193626.1_Uncultured_marine mat
GU193627.1_Uncultured_marine mat
GU193628.1_Uncultured_marine mat
GU193629.1_Uncultured_marine mat
GU193630.1_Uncultured_marine mat
GU193632.1_Uncultured_marine mat
GU193633.1_Uncultured_marine mat
GU193634.1_Uncultured_marine mat
GU193635.1_Uncultured_marine mat
GU193637.1_Uncultured_marine mat
GU193638.1_Uncultured_marine mat
GU193639.1_Uncultured_marine mat
GU193640.1_Uncultured_marine mat
GU193641.1_Uncultured_marine mat
GU193642.1_Uncultured_marine mat
GU193644.1_Uncultured_marine mat
GU193645.1_Uncultured_marine mat
GU193646.1_Uncultured_marine mat
GU193647.1_Uncultured_marine mat
GU193648.1_Uncultured_marine mat
GU193649.1_Uncultured_marine mat

GU193650.1_Uncultured_marine mat
GU193651.1_Uncultured_marine mat
GU193653.1_Uncultured_marine mat
GU193654.1_Uncultured_marine mat
GU193655.1_Uncultured_marine mat
GU193656.1_Uncultured_marine mat
GU193657.1_Uncultured_marine mat
GU193658.1_Uncultured_marine mat
GU193659.1_Uncultured_marine mat
GU193660.1_Uncultured_marine mat
GU193662.1_Uncultured_marine mat
GU193663.1_Uncultured_marine mat
GU193664.1_Uncultured_marine mat
GU193665.1_Uncultured_marine mat
GU193666.1_Uncultured_marine mat
GU193667.1_Uncultured_marine mat
GU193668.1_Uncultured_marine mat
GU193669.1_Uncultured_marine mat
GU193670.1_Uncultured_marine mat
GU193671.1_Uncultured_marine mat
GU193674.1_Uncultured_marine mat
GU193675.1_Uncultured_marine mat
GU193676.1_Uncultured_marine mat
GU193677.1_Uncultured_marine mat
GU193679.1_Uncultured_marine mat
GU193680.1_Uncultured_marine mat
GU193683.1_Uncultured_marine mat
GU193684.1_Uncultured_marine mat
GU193687.1_Uncultured_marine mat
GU193688.1_Uncultured_marine mat
GU193689.1_Uncultured_marine mat
GU193690.1_Uncultured_marine mat
GU193691.1_Uncultured_marine mat
GU193692.1_Uncultured_marine mat
GU193694.1_Uncultured_marine mat
GU193695.1_Uncultured_marine mat
GU193697.1_Uncultured_marine mat
GU193698.1_Uncultured_marine mat
GU193699.1_Uncultured_marine mat
GU193700.1_Uncultured_marine mat
GU193701.1_Uncultured_marine mat
GU193702.1_Uncultured_marine mat
GU193703.1_Uncultured_marine mat
GU193764.1_Uncultured_marine mat

GU193773.1_Uncultured_marine mat
GU193776.1_Uncultured_marine mat
GU193787.1_Uncultured_marine mat
GU193788.1_Uncultured_marine mat
GU193792.1_Uncultured_marine mat
GU193796.1_Uncultured_marine mat
GU193802.1_Uncultured_marine mat
GU193803.1_Uncultured_marine mat
GU193812.1_Uncultured_marine mat
GU193813.1_Uncultured_marine mat
GU193814.1_Uncultured_marine mat
GU193815.1_Uncultured_marine mat
GU193817.1_Uncultured_marine mat
GU193821.1_Uncultured_marine mat
GU193825.1_Uncultured_marine mat
GU193829.1_Uncultured_marine mat
GU193834.1_Uncultured_marine mat
GU193837.1_Uncultured_marine mat
GU193851.1_Uncultured_marine mat
GU193856.1_Uncultured_marine mat
GU193858.1_Uncultured_marine mat
GU193861.1_Uncultured_marine mat
GU193864.1_Uncultured_marine mat
GU193865.1_Uncultured_marine mat
GU193866.1_Uncultured_marine mat
GU193867.1_Uncultured_marine mat
GU193869.1_Uncultured_marine mat
GU193870.1_Uncultured_marine mat
GU193871.1_Uncultured_marine mat
GU193872.1_Uncultured_marine mat
GU193873.1_Uncultured_marine mat
GU193874.1_Uncultured_marine mat
GU193875.1_Uncultured_marine mat
GU193877.1_Uncultured_marine mat
GU193878.1_Uncultured_marine mat
GU193881.1_Uncultured_marine mat
GU193882.1_Uncultured_marine mat
GU193883.1_Uncultured_marine mat
GU193884.1_Uncultured_marine mat
GU193885.1_Uncultured_marine mat
GU193888.1_Uncultured_marine mat
GU193890.1_Uncultured_marine mat
GU193891.1_Uncultured_marine mat
GU193892.1_Uncultured_marine mat

GU193893.1_Uncultured_marine mat
GU193897.1_Uncultured_marine mat
GU193898.1_Uncultured_marine mat
GU193901.1_Uncultured_marine mat
GU193903.1_Uncultured_marine mat
GU193904.1_Uncultured_marine mat
GU193908.1_Uncultured_marine mat
GU193910.1_Uncultured_marine mat
GU193914.1_Uncultured_marine mat
GU193916.1_Uncultured_marine mat
GU193918.1_Uncultured_marine mat
GU193919.1_Uncultured_marine mat
GU193920.1_Uncultured_marine mat
GU193921.1_Uncultured_marine mat
GU193925.1_Uncultured_marine mat
GU193926.1_Uncultured_marine mat
GU193927.1_Uncultured_marine mat
GU193929.1_Uncultured_marine mat
GU193930.1_Uncultured_marine mat
GU193931.1_Uncultured_marine mat
GU193932.1_Uncultured_marine mat
GU193933.1_Uncultured_marine mat
GU193936.1_Uncultured_marine mat
GU193938.1_Uncultured_marine mat
GU193939.1_Uncultured_marine mat
GU193940.1_Uncultured_marine mat
GU193942.1_Uncultured_marine mat
GU193943.1_Uncultured_marine mat
GU193944.1_Uncultured_marine mat
GU193945.1_Uncultured_marine mat
GU193946.1_Uncultured_marine mat
GU193947.1_Uncultured_marine mat
GU193948.1_Uncultured_marine mat
GU193954.1_Uncultured_marine mat
GU193955.1_Uncultured_marine mat
GU193957.1_Uncultured_marine mat
GU193960.1_Uncultured_marine mat
GU193972.1_Uncultured_marine mat
GU193975.1_Uncultured_marine mat
GU196851.1_Uncultured_marine dinoflagellate
GU196852.1_Uncultured_marine dinoflagellate
GU196853.1_Uncultured_marine dinoflagellate
GU196854.1_Uncultured_marine dinoflagellate
GU196855.1_Uncultured_marine dinoflagellate

GU196856.1_Uncultured_marine dinoflagellate
GU196857.1_Uncultured_marine dinoflagellate
GU196858.1_Uncultured_marine dinoflagellate
GU196861.1_Uncultured_marine dinoflagellate
GU196862.1_Uncultured_marine dinoflagellate
GU196865.1_Uncultured_marine dinoflagellate
GU196866.1_Uncultured_marine dinoflagellate
GU196867.1_Uncultured_marine dinoflagellate
GU196869.1_Uncultured_marine dinoflagellate
GU196870.1_Uncultured_marine dinoflagellate
GU196871.1_Uncultured_marine dinoflagellate
GU196872.1_Uncultured_marine dinoflagellate
GU196873.1_Uncultured_marine dinoflagellate
GU196880.1_Uncultured_marine dinoflagellate
GU196881.1_Uncultured_marine dinoflagellate
GU196882.1_Uncultured_marine dinoflagellate
GU196883.1_Uncultured_marine dinoflagellate
GU196884.1_Uncultured_marine dinoflagellate
GU196885.1_Uncultured_marine dinoflagellate
GU196913.1_Uncultured_marine dinoflagellate
GU196925.1_Uncultured_marine dinoflagellate
GU196926.1_Uncultured_marine dinoflagellate
GU196927.1_Uncultured_marine dinoflagellate
GU196928.1_Uncultured_marine dinoflagellate
GU196941.1_Uncultured_marine dinoflagellate
GU196942.1_Uncultured_marine dinoflagellate
GU196944.1_Uncultured_marine dinoflagellate
GU196945.1_Uncultured_marine dinoflagellate
GU196946.1_Uncultured_marine dinoflagellate
GU196952.1_Uncultured_marine dinoflagellate
GU196953.1_Uncultured_marine dinoflagellate
GU196966.1_Uncultured_marine dinoflagellate
GU196967.1_Uncultured_marine dinoflagellate
GU196968.1_Uncultured_marine dinoflagellate
GU196969.1_Uncultured_marine dinoflagellate
GU196970.1_Uncultured_marine dinoflagellate
GU232760.1_Uncultured_hydrothermal vent
GU362118.1_Uncultured_wasteland
GU367884.1_Uncultured_marine sediment
GU367888.1_Uncultured_marine sediment
GU367893.1_Uncultured_marine sediment
GU478938.1_Uncultured_soil
GU478945.1_Uncultured_soil
GU593984.1_Uncultured_freshwater

GU593987.1_Uncultured_freshwater
GU593988.1_Uncultured_freshwater
GU593990.1_Uncultured_freshwater
GU593992.1_Uncultured_freshwater
GU593996.1_Uncultured_freshwater
GU593997.1_Uncultured_freshwater
GU594000.1_Uncultured_freshwater
GU594001.1_Uncultured_freshwater
GU594002.1_Uncultured_freshwater
GU594003.1_Uncultured_freshwater
GU594004.1_Uncultured_freshwater
GU594010.1_Uncultured_freshwater
GU594011.1_Uncultured_freshwater
GU594014.1_Uncultured_freshwater
GU594016.1_Uncultured_freshwater
GU594017.1_Uncultured_freshwater
GU594018.1_Uncultured_freshwater
GU594020.1_Uncultured_freshwater
GU594021.1_Uncultured_freshwater
GU594022.1_Uncultured_freshwater
GU594023.1_Uncultured_freshwater
HE599414.1_Uncultured_marine sediment
HE599416.1_Uncultured_marine sediment
HE599428.1_Uncultured_marine sediment
HE599439.1_Uncultured_marine sediment
HE599442.1_Uncultured_marine sediment
HE599443.1_Uncultured_marine sediment
HE599444.1_Uncultured_marine sediment
HE599470.1_Uncultured_marine sediment
HE599471.1_Uncultured_marine sediment
HE599472.1_Uncultured_marine sediment
HE599477.1_Uncultured_marine sediment
HE599486.1_Uncultured_marine sediment
HE600410.1_Uncultured_moss
HE600475.1_Uncultured_moss
HE600656.1_Uncultured_moss
HE655432.1_Pseudomonas_rhizosphere
HE801219.1_Methylomonas
HE803322.1_Uncultured_culture
HE803323.1_Uncultured_culture
HE803325.1_Uncultured_culture
HE803329.1_Uncultured_culture
HE803330.1_Uncultured_culture
HF559482.1_Uncultured_wood

HF559483.1_Uncultured_wood
HF559498.1_Uncultured_wood
HF559499.1_Uncultured_wood
HF559505.1_Uncultured_wood
HF559531.1_Uncultured_wood
HF559535.1_Uncultured_wood
HF559538.1_Uncultured_wood
HF559548.1_Uncultured_wood
HF559551.1_Uncultured_wood
HF559579.1_Uncultured_wood
HF559580.1_Uncultured_wood
HF559592.1_Uncultured_wood
HF559596.1_Uncultured_wood
HF559605.1_Uncultured_wood
HF559607.1_Uncultured_wood
HF559614.1_Uncultured_wood
HF559615.1_Uncultured_wood
HF559618.1_Uncultured_wood
HF559621.1_Uncultured_wood
HF559626.1_Uncultured_wood
HF559631.1_Uncultured_wood
HF559634.1_Uncultured_wood
HF559637.1_Uncultured_wood
HF559639.1_Uncultured_wood
HF559640.1_Uncultured_wood
HF559642.1_Uncultured_wood
HF559646.1_Uncultured_wood
HF559648.1_Uncultured_wood
HF559700.1_Uncultured_wood
HF559704.1_Uncultured_wood
HF559834.1_Uncultured_wood
HF559851.1_Uncultured_wood
HF559873.1_Uncultured_wood
HF559877.1_Uncultured_wood
HF559939.1_Uncultured_wood
HF559953.1_Uncultured_wood
HF559954.1_Uncultured_wood
HF559962.1_Uncultured_wood
HF559963.1_Uncultured_wood
HF559964.1_Uncultured_wood
HF559969.1_Uncultured_wood
HF559970.1_Uncultured_wood
HF559975.1_Uncultured_wood
HF559978.1_Uncultured_wood

HF559979.1_Uncultured_wood
HF559983.1_Uncultured_wood
HF559985.1_Uncultured_wood
HF559986.1_Uncultured_wood
HF559994.1_Uncultured_wood
HF559995.1_Uncultured_wood
HF560020.1_Uncultured_wood
HF560029.1_Uncultured_wood
HF560060.1_Uncultured_wood
HF560064.1_Uncultured_wood
HF560067.1_Uncultured_wood
HF560068.1_Uncultured_wood
HF560073.1_Uncultured_wood
HF560079.1_Uncultured_wood
HF560091.1_Uncultured_wood
HF560093.1_Uncultured_wood
HF560096.1_Uncultured_wood
HF560109.1_Uncultured_wood
HF560112.1_Uncultured_wood
HF560250.1_Uncultured_wood
HF560278.1_Uncultured_wood
HF560351.1_Uncultured_wood
HF560394.1_Uncultured_wood
HF560461.1_Uncultured_wood
HF560482.1_Uncultured_wood
HF560510.1_Uncultured_wood
HF560523.1_Uncultured_wood
HF560538.1_Uncultured_wood
HF560541.1_Uncultured_wood
HF560550.1_Uncultured_wood
HF560557.1_Uncultured_wood
HF560560.1_Uncultured_wood
HF565530.1_Uncultured_biofilm
HF565531.1_Uncultured_marine plankton
HF565532.1_Uncultured_marine plankton
HF565533.1_Uncultured_marine plankton
HF565534.1_Uncultured_marine plankton
HF565535.1_Uncultured_marine plankton
HF565536.1_Uncultured_marine plankton
HF565537.1_Uncultured_marine plankton
HF565538.1_Uncultured_marine plankton
HF565539.1_Uncultured_marine plankton
HF565540.1_Uncultured_marine plankton
HF565541.1_Uncultured_marine plankton

HF565542.1_Uncultured_marine plankton
HF565543.1_Uncultured_marine plankton
HF565544.1_Uncultured_marine plankton
HF565545.1_Uncultured_marine plankton
HF565546.1_Uncultured_marine plankton
HF565547.1_Uncultured_marine plankton
HF565548.1_Uncultured_marine plankton
HF565549.1_Uncultured_marine plankton
HF565550.1_Uncultured_marine plankton
HF565551.1_Uncultured_marine plankton
HF565552.1_Uncultured_marine plankton
HF565553.1_Uncultured_marine plankton
HF565554.1_Uncultured_marine plankton
HF565555.1_Uncultured_marine plankton
HF565556.1_Uncultured_marine plankton
HF565557.1_Uncultured_marine plankton
HF565558.1_Uncultured_marine plankton
HF565559.1_Uncultured_marine plankton
HF565560.1_Uncultured_marine plankton
HF565561.1_Uncultured_marine plankton
HF565562.1_Uncultured_marine plankton
HF565563.1_Uncultured_marine plankton
HF565564.1_Uncultured_marine plankton
HF565565.1_Uncultured_marine plankton
HF565566.1_Uncultured_marine plankton
HF565568.1_Uncultured_marine sediment
HF954362.1_Methylomonas
HF954374.1_Methylomonas
HG422878.1_Uncultured_mire
HG422902.1_Uncultured_mire
HG422903.1_Uncultured_mire
HG422915.1_Uncultured_mire
HG423024.1_Uncultured_river
HG423025.1_Uncultured_river
HG423094.1_Uncultured_river
HM021145.1_Uncultured_marine sediment
HM042878.1_PCRreagent
HM042879.1_PCRreagent
HM042880.1_PCRreagent
HM042881.1_PCRreagent
HM042882.1_PCRreagent
HM042883.1_PCRreagent
HM042884.1_PCRreagent
HM042885.1_PCRreagent

HM042886.1_PCRreagent
HM042887.1_PCRreagent
HM042888.1_PCRreagent
HM042889.1_PCRreagent
HM042890.1_PCRreagent
HM042891.1_PCRreagent
HM042892.1_PCRreagent
HM042893.1_PCRreagent
HM063705.1_Uncultured_soil
HM063706.1_Uncultured_soil
HM063747.1_Uncultured_marine sediment
HM063750.1_Uncultured_marine sediment
HM063751.1_Uncultured_marine sediment
HM063752.1_Uncultured_marine sediment
HM063753.1_Uncultured_marine sediment
HM063754.1_Uncultured_marine sediment
HM063755.1_Uncultured_marine sediment
HM063756.1_Uncultured_marine sediment
HM063757.1_Uncultured_marine sediment
HM063759.1_Uncultured_marine sediment
HM063760.1_Uncultured_marine sediment
HM063761.1_Uncultured_marine sediment
HM063764.1_Uncultured_marine sediment
HM063765.1_Uncultured_marine sediment
HM063766.1_Uncultured_marine sediment
HM063768.1_Uncultured_marine sediment
HM063776.1_Uncultured_marine sediment
HM063778.1_Uncultured_marine sediment
HM063782.1_Uncultured_marine sediment
HM063784.1_Uncultured_marine sediment
HM063787.1_Uncultured_marine sediment
HM063788.1_Uncultured_marine sediment
HM063789.1_Uncultured_marine sediment
HM063793.1_Uncultured_marine sediment
HM063794.1_Uncultured_marine sediment
HM063803.1_Uncultured_marine sediment
HM063804.1_Uncultured_marine sediment
HM063818.1_Uncultured_marine sediment
HM063822.1_Uncultured_marine sediment
HM063825.1_Uncultured_marine sediment
HM063827.1_Uncultured_marine sediment
HM063828.1_Uncultured_marine sediment
HM063829.1_Uncultured_marine sediment
HM113546.1_Uncultured_hot spring

HM113547.1_Uncultured_hot spring
HM140729.1_Uncultured_terrestrial microbial mat
HM140733.1_Uncultured_terrestrial microbial mat
HM140742.1_Uncultured_terrestrial microbial mat
HM140743.1_Uncultured_terrestrial microbial mat
HM140744.1_Uncultured_terrestrial microbial mat
HM140752.1_Uncultured_terrestrial microbial mat
HM140761.1_Uncultured_terrestrial microbial mat
HM140763.1_Uncultured_terrestrial microbial mat
HM140765.1_Uncultured_terrestrial microbial mat
HM149325.1_Ectothiorhodospira
HM182107.1_Uncultured_rhizosphere
HM182108.1_Uncultured_rhizosphere
HM182109.1_Uncultured_rhizosphere
HM182111.1_Uncultured_rhizosphere
HM182147.1_Uncultured_rhizosphere
HM182148.1_Uncultured_rhizosphere
HM182149.1_Uncultured_rhizosphere
HM182152.1_Uncultured_rhizosphere
HM182153.1_Uncultured_rhizosphere
HM182154.1_Uncultured_rhizosphere
HM182158.1_Uncultured_rhizosphere
HM182165.1_Uncultured_rhizosphere
HM210309.1_Uncultured_marine
HM210311.1_Uncultured_marine
HM210324.1_Uncultured_marine
HM210325.1_Uncultured_marine
HM210326.1_Uncultured_marine
HM210327.1_Uncultured_marine
HM210334.1_Uncultured_marine
HM210338.1_Uncultured_marine
HM210342.1_Uncultured_marine
HM210343.1_Gamma2_marine
HM210345.1_Uncultured_marine
HM210358.1_Uncultured_marine
HM210359.1_Uncultured_marine
HM210360.1_Uncultured_marine
HM210361.1_Uncultured_marine
HM210362.1_Uncultured_marine
HM210363.1_Gamma4_marine
HM210364.1_Uncultured_marine
HM210365.1_Uncultured_marine
HM210366.1_Uncultured_marine
HM210369.1_Uncultured_marine

HM210370.1_Uncultured_marine
HM210371.1_Uncultured_marine
HM210372.1_Uncultured_marine
HM210373.1_Uncultured_marine
HM210374.1_Uncultured_marine
HM210375.1_Uncultured_marine
HM210376.1_Uncultured_marine
HM210377.1_Gamma1_marine
HM210378.1_Uncultured_marine
HM210379.1_Uncultured_marine
HM210381.1_Uncultured_marine
HM210384.1_Uncultured_marine
HM210385.1_Uncultured_marine
HM210386.1_Uncultured_marine
HM210388.1_Uncultured_marine
HM210390.1_Uncultured_marine
HM210393.1_Uncultured_marine
HM210394.1_Uncultured_marine
HM210395.1_Uncultured_marine
HM210396.1_Uncultured_marine
HM210397.1_Gamma3_marine
HM210403.1_Uncultured_marine
HM210404.1_Uncultured_marine
HM210405.1_Uncultured_marine
HM210406.1_Uncultured_marine
HM210407.1_Uncultured_marine
HM210408.1_Uncultured_marine
HM210409.1_Uncultured_marine
HM210410.1_Uncultured_marine
HM210411.1_Uncultured_marine
HM210412.1_Uncultured_marine
HM210413.1_Uncultured_marine
HM210414.1_Uncultured_marine
HM219676.1_Uncultured_marine mudflat
HM219684.1_Uncultured_marine mudflat
HM219695.1_Uncultured_marine mudflat
HM219701.1_Uncultured_marine mudflat
HM219710.1_Uncultured_marine mudflat
HM219711.1_Uncultured_marine mudflat
HM219713.1_Uncultured_marine mudflat
HM219715.1_Uncultured_marine mudflat
HM219744.1_Uncultured_marine mudflat
HM219748.1_Uncultured_marine mudflat
HM219749.1_Uncultured_marine mudflat

HM219751.1_Uncultured_marine mudflat
HM219757.1_Uncultured_marine mudflat
HM219758.1_Uncultured_marine mudflat
HM219776.1_Uncultured_marine mudflat
HM219777.1_Uncultured_marine mudflat
HM219791.1_Uncultured_marine mudflat
HM219795.1_Uncultured_marine mudflat
HM219796.1_Uncultured_marine mudflat
HM219804.1_Uncultured_marine mudflat
HM219809.1_Uncultured_marine mudflat
HM219820.1_Uncultured_marine mudflat
HM219823.1_Uncultured_marine mudflat
HM219837.1_Uncultured_marine mudflat
HM219838.1_Uncultured_marine mudflat
HM246699.1_Uncultured_soil
HM565843.1_Uncultured_wasteland
HM565858.1_Uncultured_wasteland
HM601483.1_Uncultured_marine
HM601484.1_Uncultured_marine
HM601485.1_Uncultured_marine
HM601487.1_Uncultured_marine
HM601488.1_Uncultured_marine
HM601490.1_Uncultured_marine
HM601493.1_Uncultured_marine
HM601497.1_Uncultured_marine
HM601500.1_Uncultured_marine
HM601505.1_Uncultured_marine
HM601507.1_Uncultured_marine
HM601508.1_Uncultured_marine
HM601510.1_Uncultured_marine
HM601511.1_Uncultured_marine
HM601512.1_Uncultured_marine
HM601513.1_Uncultured_marine
HM601516.1_Uncultured_marine
HM601518.1_Uncultured_marine
HM601520.1_Uncultured_marine
HM601521.1_Uncultured_marine
HM601522.1_Uncultured_marine
HM601524.1_Uncultured_marine
HM601527.1_Uncultured_marine
HM601528.1_Uncultured_marine
HM601529.1_Uncultured_marine
HM601532.1_Uncultured_marine
HM601533.1_Uncultured_marine

HM601534.1_Uncultured_marine
HM601535.1_Uncultured_marine
HM601536.1_Uncultured_marine
HM601537.1_Uncultured_marine
HM601539.1_Uncultured_marine
HM601540.1_Uncultured_marine
HM601541.1_Uncultured_marine
HM750262.1_Uncultured_rhizosphere
HM750263.1_Uncultured_rhizosphere
HM750265.1_Uncultured_rhizosphere
HM750266.1_Uncultured_rhizosphere
HM750267.1_Uncultured_rhizosphere
HM750268.1_Uncultured_rhizosphere
HM750269.1_Uncultured_rhizosphere
HM750270.1_Uncultured_rhizosphere
HM750271.1_Uncultured_rhizosphere
HM750275.1_Uncultured_rhizosphere
HM750277.1_Uncultured_rhizosphere
HM750278.1_Uncultured_rhizosphere
HM750279.1_Uncultured_rhizosphere
HM750280.1_Uncultured_rhizosphere
HM750281.1_Uncultured_rhizosphere
HM750284.1_Uncultured_rhizoplane
HM750289.1_Uncultured_rhizoplane
HM750291.1_Uncultured_rhizoplane
HM750293.1_Uncultured_rhizoplane
HM750294.1_Uncultured_rhizoplane
HM750295.1_Uncultured_rhizoplane
HM750308.1_Uncultured_rhizoplane
HM750316.1_Uncultured_rhizosphere
HM750319.1_Uncultured_rhizosphere
HM750324.1_Uncultured_rhizosphere
HM750332.1_Uncultured_rhizosphere
HM750333.1_Uncultured_rhizosphere
HM750334.1_Uncultured_rhizosphere
HM750335.1_Uncultured_rhizosphere
HM750336.1_Uncultured_rhizosphere
HM750337.1_Uncultured_rhizosphere
HM750338.1_Uncultured_rhizosphere
HM750343.1_Uncultured_rhizosphere
HM750349.1_Uncultured_rhizosphere
HM750362.1_Uncultured_rhizosphere
HM750364.1_Uncultured_rhizosphere
HM750366.1_Uncultured_rhizosphere

HM750368.1_Uncultured_rhizosphere
HM750369.1_Uncultured_rhizosphere
HM750370.1_Uncultured_rhizosphere
HM750373.1_Uncultured_rhizosphere
HM750374.1_Uncultured_rhizosphere
HM750375.1_Uncultured_rhizosphere
HM750380.1_Uncultured_rhizosphere
HM750383.1_Uncultured_rhizosphere
HM750387.1_Uncultured_rhizosphere
HM750388.1_Uncultured_rhizosphere
HM750398.1_Uncultured_rhizosphere
HM750399.1_Uncultured_rhizosphere
HM750400.1_Uncultured_rhizosphere
HM750401.1_Uncultured_rhizosphere
HM750402.1_Uncultured_rhizosphere
HM750404.1_Uncultured_rhizosphere
HM750406.1_Uncultured_rhizosphere
HM750412.1_Uncultured_rhizoplane
HM750415.1_Uncultured_rhizoplane
HM750417.1_Uncultured_rhizoplane
HM750427.1_Uncultured_rhizoplane
HM750429.1_Uncultured_rhizoplane
HM750432.1_Uncultured_rhizoplane
HM750434.1_Uncultured_rhizoplane
HM750436.1_Uncultured_rhizoplane
HM750437.1_Uncultured_rhizoplane
HM750438.1_Uncultured_rhizoplane
HM750439.1_Uncultured_rhizoplane
HM750441.1_Uncultured_rhizoplane
HM750442.1_Uncultured_rhizoplane
HM750443.1_Uncultured_rhizoplane
HM750445.1_Uncultured_rhizoplane
HM750446.1_Uncultured_rhizoplane
HM750456.1_Uncultured_rhizosphere
HM750461.1_Uncultured_rhizosphere
HM750469.1_Uncultured_rhizosphere
HM750502.1_Uncultured_rhizosphere
HM750509.1_Uncultured_rhizosphere
HM750512.1_Uncultured_rhizosphere
HM750515.1_Uncultured_rhizosphere
HM750516.1_Uncultured_rhizosphere
HM750520.1_Uncultured_rhizoplane
HM750523.1_Uncultured_rhizoplane
HM750526.1_Uncultured_rhizoplane

HM750532.1_Uncultured_rhizoplane
HM750540.1_Uncultured_rhizoplane
HM750545.1_Uncultured_rhizoplane
HM750547.1_Uncultured_rhizoplane
HM750550.1_Uncultured_rhizoplane
HM750625.1_Uncultured_rhizoplane
HM750626.1_Uncultured_rhizoplane
HM750629.1_Uncultured_rhizoplane
HM750632.1_Uncultured_rhizoplane
HM750643.1_Uncultured_rhizoplane
HM750644.1_Uncultured_rhizoplane
HM750645.1_Uncultured_rhizoplane
HM750647.1_Uncultured_rhizoplane
HM750648.1_Uncultured_rhizoplane
HM750654.1_Uncultured_rhizoplane
HM750660.1_Uncultured_rhizosphere
HM750661.1_Uncultured_rhizosphere
HM750662.1_Uncultured_rhizosphere
HM750663.1_Uncultured_rhizosphere
HM750664.1_Uncultured_rhizosphere
HM750699.1_Uncultured_rhizosphere
HM750703.1_Uncultured_rhizosphere
HM750705.1_Uncultured_rhizosphere
HM750709.1_Uncultured_rhizosphere
HM750710.1_Uncultured_rhizosphere
HM750711.1_Uncultured_rhizosphere
HM750712.1_Uncultured_rhizosphere
HM750713.1_Uncultured_rhizosphere
HM750716.1_Uncultured_rhizosphere
HM750717.1_Uncultured_rhizosphere
HM750721.1_Uncultured_rhizosphere
HM750731.1_Uncultured_rhizosphere
HM750732.1_Uncultured_rhizosphere
HM750737.1_Uncultured_rhizosphere
HM750738.1_Uncultured_rhizosphere
HM750739.1_Uncultured_rhizosphere
HM750741.1_Uncultured_rhizoplane
HM750742.1_Uncultured_rhizoplane
HM750743.1_Uncultured_rhizoplane
HM750744.1_Uncultured_rhizoplane
HM750745.1_Uncultured_rhizoplane
HM750746.1_Uncultured_rhizoplane
HM750747.1_Uncultured_rhizoplane
HM750748.1_Uncultured_rhizoplane

HM750749.1_Uncultured_rhizoplane
HM750750.1_Uncultured_rhizoplane
HM750753.1_Uncultured_rhizoplane
HM750754.1_Uncultured_rhizoplane
HM750755.1_Uncultured_rhizoplane
HM750756.1_Uncultured_rhizoplane
HM750758.1_Uncultured_rhizoplane
HM750760.1_Uncultured_rhizoplane
HM750764.1_Uncultured_rhizoplane
HM750765.1_Uncultured_rhizoplane
HM750772.1_Uncultured_rhizoplane
HM750773.1_Uncultured_rhizoplane
HM750786.1_Uncultured_rhizoplane
HM750790.1_Uncultured_rhizoplane
HM750798.1_Uncultured_rhizoplane
HM750799.1_Uncultured_rhizoplane
HM750805.1_Uncultured_rhizoplane
HM750806.1_Uncultured_rhizoplane
HM750809.1_Uncultured_rhizoplane
HM750810.1_Uncultured_rhizoplane
HM750811.1_Uncultured_rhizoplane
HM750813.1_Uncultured_rhizoplane
HM750814.1_Uncultured_rhizoplane
HM750816.1_Uncultured_rhizoplane
HM801226.1_Uncultured_marine
HM801245.1_Uncultured_marine
HM801250.1_Uncultured_marine
HM801253.1_Uncultured_marine
HM801254.1_Uncultured_marine
HM801257.1_Uncultured_marine
HM801259.1_Uncultured_marine
HM801261.1_Uncultured_marine
HM801264.1_Uncultured_marine
HM801265.1_Uncultured_marine
HM801267.1_Uncultured_marine
HM801272.1_Uncultured_marine
HM801277.1_Uncultured_marine
HM801335.1_Uncultured_marine
HM801336.1_Uncultured_marine
HM801344.1_Uncultured_marine
HM801346.1_Uncultured_marine
HM801349.1_Uncultured_marine
HM801351.1_Uncultured_marine
HM801354.1_Uncultured_marine

HM801356.1_Uncultured_marine
HM801360.1_Uncultured_marine
HM801361.1_Uncultured_marine
HM801364.1_Uncultured_marine
HM801365.1_Uncultured_marine
HM801367.1_Uncultured_marine
HM801369.1_Uncultured_marine
HM801371.1_Uncultured_marine
HM801372.1_Uncultured_marine
HM801381.1_Uncultured_marine
HM801382.1_Uncultured_marine
HM801397.1_Uncultured_marine
HM801411.1_Uncultured_marine
HM801415.1_Uncultured_marine
HM801416.1_Uncultured_marine
HM801417.1_Uncultured_marine
HM801420.1_Uncultured_marine
HM801421.1_Uncultured_marine
HM801423.1_Uncultured_marine
HM801424.1_Uncultured_marine
HM801426.1_Uncultured_marine
HM801430.1_Uncultured_marine
HM801434.1_Uncultured_marine
HM801437.1_Uncultured_marine
HM801438.1_Uncultured_marine
HM801439.1_Uncultured_marine
HM801440.1_Uncultured_marine
HM801443.1_Uncultured_marine
HM801444.1_Uncultured_marine
HM801445.1_Uncultured_marine
HM801446.1_Uncultured_marine
HM801450.1_Uncultured_marine
HM801451.1_Uncultured_marine
HM801452.1_Uncultured_marine
HM801454.1_Uncultured_marine
HM801455.1_Uncultured_marine
HM801457.1_Uncultured_marine
HM801459.1_Uncultured_marine
HM801460.1_Uncultured_marine
HM801461.1_Uncultured_marine
HM801462.1_Uncultured_marine
HM801464.1_Uncultured_marine
HM801471.1_Uncultured_marine
HM801515.1_Uncultured_marine

HM801516.1_Uncultured_marine
HM801518.1_Uncultured_marine
HM801519.1_Uncultured_marine
HM801520.1_Uncultured_marine
HM801521.1_Uncultured_marine
HM801522.1_Uncultured_marine
HM801523.1_Uncultured_marine
HM801524.1_Uncultured_marine
HM801525.1_Uncultured_marine
HM801526.1_Uncultured_marine
HM801527.1_Uncultured_marine
HM801530.1_Uncultured_marine
HM801532.1_Uncultured_marine
HM801533.1_Uncultured_marine
HM801534.1_Uncultured_marine
HM801536.1_Uncultured_marine
HM801537.1_Uncultured_marine
HM801539.1_Uncultured_marine
HM801540.1_Uncultured_marine
HM801541.1_Uncultured_marine
HM801543.1_Uncultured_marine
HM801544.1_Uncultured_marine
HM801545.1_Uncultured_marine
HM801547.1_Uncultured_marine
HM801548.1_Uncultured_marine
HM801549.1_Uncultured_marine
HM801550.1_Uncultured_marine
HM801551.1_Uncultured_marine
HM801553.1_Uncultured_marine
HM801554.1_Uncultured_marine
HM801555.1_Uncultured_marine
HM801560.1_Uncultured_marine
HM801561.1_Uncultured_marine
HM801562.1_Uncultured_marine
HM801563.1_Uncultured_marine
HM801564.1_Uncultured_marine
HM801566.1_Uncultured_marine
HM801567.1_Uncultured_marine
HM801568.1_Uncultured_marine
HM801569.1_Uncultured_marine
HM801570.1_Uncultured_marine
HM801571.1_Uncultured_marine
HM801572.1_Uncultured_marine
HM801573.1_Uncultured_marine

HM801574.1_Uncultured_marine
HM801575.1_Uncultured_marine
HM801576.1_Uncultured_marine
HM801577.1_Uncultured_marine
HM801578.1_Uncultured_marine
HM801579.1_Uncultured_marine
HM801580.1_Uncultured_marine
HM801581.1_Uncultured_marine
HM801582.1_Uncultured_marine
HM801583.1_Uncultured_marine
HM801584.1_Uncultured_marine
HM801585.1_Uncultured_marine
HM801586.1_Uncultured_marine
HM801587.1_Uncultured_marine
HM801610.1_Uncultured_marine
HM801623.1_Uncultured_marine
HM801708.1_Uncultured_marine
HM801737.1_Uncultured_marine
HM801738.1_Uncultured_marine
HM801739.1_Uncultured_marine
HM801740.1_Uncultured_marine
HM801741.1_Uncultured_marine
HM801744.1_Uncultured_marine
HM801751.1_Uncultured_marine
HM801752.1_Uncultured_marine
HM801755.1_Uncultured_marine
HM801757.1_Uncultured_marine
HM801762.1_Uncultured_marine
HM999098.1_Uncultured_marine
HM999105.1_Uncultured_marine
HM999106.1_Uncultured_marine
HM999117.1_Uncultured_marine
HM999123.1_Uncultured_marine
HM999141.1_Uncultured_marine
HM999148.1_Uncultured_marine
HM999196.1_Uncultured_marine
HM999197.1_Uncultured_marine
HM999199.1_Uncultured_marine
HM999200.1_Uncultured_marine
HM999201.1_Uncultured_marine
HM999203.1_Uncultured_marine
HM999204.1_Uncultured_marine
HM999205.1_Uncultured_marine
HM999207.1_Uncultured_marine

HM999210.1_Uncultured_marine
HM999211.1_Uncultured_marine
HM999212.1_Uncultured_marine
HM999213.1_Uncultured_marine
HM999215.1_Uncultured_marine
HM999216.1_Uncultured_marine
HM999219.1_Uncultured_marine
HM999220.1_Uncultured_marine
HM999222.1_Uncultured_marine
HM999352.1_Uncultured_marine
HM999396.1_Uncultured_marine
HM999432.1_Uncultured_marine
HM999434.1_Uncultured_marine
HM999437.1_Uncultured_marine
HM999440.1_Uncultured_marine
HM999441.1_Uncultured_marine
HM999442.1_Uncultured_marine
HM999443.1_Uncultured_marine
HM999447.1_Uncultured_marine
HM999448.1_Uncultured_marine
HM999450.1_Uncultured_marine
HM999452.1_Uncultured_marine
HM999461.1_Uncultured_marine
HM999462.1_Uncultured_marine
HM999463.1_Uncultured_marine
HM999464.1_Uncultured_marine
HM999468.1_Uncultured_marine
HM999469.1_Uncultured_marine
HM999471.1_Uncultured_marine
HM999506.1_Uncultured_marine
HM999509.1_Uncultured_marine
HM999511.1_Uncultured_marine
HM999515.1_Uncultured_marine
HM999516.1_Uncultured_marine
HM999520.1_Uncultured_marine
HM999528.1_Uncultured_marine
HM999549.1_Uncultured_marine
HM999551.1_Uncultured_marine
HM999554.1_Uncultured_marine
HM999629.1_Uncultured_marine
HQ130015.1_Uncultured_marine
HQ130026.1_Uncultured_river
HQ190136.1_Uncultured_soil
HQ190145.1_Uncultured_soil

HQ190156.1_Uncultured_soil
HQ190157.1_Uncultured_soil
HQ190160.1_Uncultured_soil
HQ204222.1_Klebsiella
HQ204225.1_Klebsiella
HQ204229.1_Klebsiella
HQ204230.1_Enterobacter
HQ204231.1_Klebsiella
HQ204232.1_Enterobacter
HQ204233.1_Enterobacter
HQ204235.1_Klebsiella
HQ204237.1_Klebsiella
HQ204243.1_Klebsiella
HQ204245.1_Enterobacter
HQ204248.1_Klebsiella
HQ204250.1_Klebsiella
HQ204254.1_Klebsiella
HQ204255.1_Enterobacter
HQ204257.1_Enterobacter
HQ223480.1_Uncultured_marine sediment
HQ223492.1_Uncultured_marine sediment
HQ223495.1_Uncultured_marine sediment
HQ223497.1_Uncultured_marine sediment
HQ223503.1_Uncultured_marine sediment
HQ223510.1_Uncultured_marine sediment
HQ223513.1_Uncultured_marine sediment
HQ223517.1_Uncultured_marine sediment
HQ223526.1_Uncultured_marine sediment
HQ223537.1_Uncultured_marine sediment
HQ223547.1_Uncultured_marine sediment
HQ223548.1_Uncultured_marine sediment
HQ223554.1_Uncultured_marine sediment
HQ223555.1_Uncultured_marine sediment
HQ223556.1_Uncultured_marine sediment
HQ223578.1_Uncultured_marine sediment
HQ223583.1_Uncultured_marine sediment
HQ223628.1_Uncultured_marine sediment
HQ223643.1_Uncultured_marine sediment
HQ223672.1_Uncultured_marine sediment
HQ223805.1_Uncultured_marine sediment
HQ223875.1_Uncultured_marine sediment
HQ223925.1_Uncultured_marine sediment
HQ223934.1_Uncultured_marine sediment
HQ223936.1_Uncultured_marine sediment

HQ223953.1_Uncultured_marine sediment
HQ224162.1_Uncultured_marine sediment
HQ224181.1_Uncultured_marine sediment
HQ224203.1_Uncultured_marine sediment
HQ224219.1_Uncultured_marine sediment
HQ224258.1_Uncultured_marine sediment
HQ224278.1_Uncultured_marine sediment
HQ224327.1_Uncultured_marine sediment
HQ224330.1_Uncultured_marine sediment
HQ224363.1_Uncultured_marine sediment
HQ224382.1_Uncultured_marine sediment
HQ224463.1_Uncultured_marine sediment
HQ335461.1_Uncultured_soil
HQ335467.1_Uncultured_soil
HQ335468.1_Uncultured_soil
HQ335469.1_Uncultured_soil
HQ335473.1_Uncultured_soil
HQ335474.1_Uncultured_soil
HQ335485.1_Uncultured_soil
HQ335487.1_Uncultured_soil
HQ335493.1_Uncultured_soil
HQ335494.1_Uncultured_soil
HQ335496.1_Uncultured_soil
HQ335498.1_Uncultured_soil
HQ335502.1_Uncultured_soil
HQ335513.1_Uncultured_soil
HQ335514.1_Uncultured_soil
HQ335518.1_Uncultured_soil
HQ335524.1_Uncultured_soil
HQ335533.1_Uncultured_soil
HQ335573.1_Uncultured_soil
HQ335579.1_Uncultured_soil
HQ335585.1_Uncultured_soil
HQ335617.1_Uncultured_soil
HQ335628.1_Uncultured_soil
HQ335629.1_Uncultured_soil
HQ335630.1_Uncultured_soil
HQ335638.1_Uncultured_soil
HQ335640.1_Uncultured_soil
HQ335641.1_Uncultured_soil
HQ335644.1_Uncultured_soil
HQ335648.1_Uncultured_soil
HQ335655.1_Uncultured_soil
HQ335659.1_Uncultured_soil

HQ335662.1_Uncultured_soil
HQ335664.1_Uncultured_soil
HQ335666.1_Uncultured_soil
HQ335672.1_Uncultured_soil
HQ335674.1_Uncultured_soil
HQ335675.1_Uncultured_soil
HQ335679.1_Uncultured_soil
HQ335680.1_Uncultured_soil
HQ335682.1_Uncultured_soil
HQ335683.1_Uncultured_soil
HQ335688.1_Uncultured_soil
HQ335691.1_Uncultured_soil
HQ335692.1_Uncultured_soil
HQ335694.1_Uncultured_soil
HQ335695.1_Uncultured_soil
HQ335702.1_Uncultured_soil
HQ335704.1_Uncultured_soil
HQ335718.1_Uncultured_soil
HQ335724.1_Uncultured_soil
HQ335731.1_Uncultured_soil
HQ335734.1_Uncultured_soil
HQ335736.1_Uncultured_soil
HQ335744.1_Uncultured_soil
HQ335752.1_Uncultured_soil
HQ335754.1_Uncultured_soil
HQ335755.1_Uncultured_soil
HQ335757.1_Uncultured_soil
HQ335759.1_Uncultured_soil
HQ335761.1_Uncultured_soil
HQ335766.1_Uncultured_soil
HQ335771.1_Uncultured_soil
HQ335776.1_Uncultured_soil
HQ335778.1_Uncultured_soil
HQ335779.1_Uncultured_soil
HQ335780.1_Uncultured_soil
HQ335782.1_Uncultured_soil
HQ335790.1_Uncultured_soil
HQ335793.1_Uncultured_soil
HQ335803.1_Uncultured_soil
HQ335809.1_Uncultured_soil
HQ335810.1_Uncultured_soil
HQ335811.1_Uncultured_soil
HQ335812.1_Uncultured_soil
HQ335818.1_Uncultured_soil

HQ335819.1_Uncultured_soil
HQ335828.1_Uncultured_soil
HQ335835.1_Uncultured_soil
HQ335839.1_Uncultured_soil
HQ335852.1_Uncultured_soil
HQ335854.1_Uncultured_soil
HQ335856.1_Uncultured_soil
HQ335863.1_Uncultured_soil
HQ335866.1_Uncultured_soil
HQ335876.1_Uncultured_soil
HQ335884.1_Uncultured_soil
HQ335887.1_Uncultured_soil
HQ335891.1_Uncultured_soil
HQ335893.1_Uncultured_soil
HQ335902.1_Uncultured_soil
HQ335907.1_Uncultured_soil
HQ335910.1_Uncultured_soil
HQ335920.1_Uncultured_soil
HQ335924.1_Uncultured_soil
HQ335930.1_Uncultured_soil
HQ335947.1_Uncultured_soil
HQ335967.1_Uncultured_soil
HQ335969.1_Uncultured_soil
HQ335987.1_Uncultured_soil
HQ335990.1_Uncultured_soil
HQ336009.1_Uncultured_soil
HQ336014.1_Uncultured_soil
HQ336019.1_Uncultured_soil
HQ336024.1_Uncultured_soil
HQ336039.1_Uncultured_soil
HQ336040.1_Uncultured_soil
HQ336041.1_Uncultured_soil
HQ404304.1_Klebsiella_root
HQ455836.1_Uncultured_South China Sea
HQ455840.1_Uncultured_South China Sea
HQ455843.1_Uncultured_South China Sea
HQ455845.1_Uncultured_South China Sea
HQ455846.1_Uncultured_South China Sea
HQ455847.1_Uncultured_South China Sea
HQ455848.1_Uncultured_South China Sea
HQ455851.1_Uncultured_South China Sea
HQ455852.1_Uncultured_South China Sea
HQ455857.1_Uncultured_South China Sea
HQ455861.1_Uncultured_South China Sea

HQ455862.1_Uncultured_South China Sea
HQ455863.1_Uncultured_South China Sea
HQ455866.1_Uncultured_South China Sea
HQ455867.1_Uncultured_South China Sea
HQ455869.1_Uncultured_South China Sea
HQ455870.1_Uncultured_South China Sea
HQ455871.1_Uncultured_South China Sea
HQ455872.1_Uncultured_South China Sea
HQ455873.1_Uncultured_South China Sea
HQ455874.1_Uncultured_South China Sea
HQ455875.1_Uncultured_South China Sea
HQ455877.1_Uncultured_South China Sea
HQ455878.1_Uncultured_South China Sea
HQ455879.1_Uncultured_South China Sea
HQ455880.1_Uncultured_South China Sea
HQ455881.1_Uncultured_South China Sea
HQ455882.1_Uncultured_South China Sea
HQ455883.1_Uncultured_South China Sea
HQ455884.1_Uncultured_South China Sea
HQ455885.1_Uncultured_South China Sea
HQ455888.1_Uncultured_South China Sea
HQ455889.1_Uncultured_South China Sea
HQ455890.1_Uncultured_South China Sea
HQ455891.1_Uncultured_South China Sea
HQ455892.1_Uncultured_South China Sea
HQ455893.1_Uncultured_South China Sea
HQ455896.1_Uncultured_South China Sea
HQ455897.1_Uncultured_South China Sea
HQ455898.1_Uncultured_South China Sea
HQ455900.1_Uncultured_South China Sea
HQ455901.1_Uncultured_South China Sea
HQ455902.1_Uncultured_South China Sea
HQ455903.1_Uncultured_South China Sea
HQ455904.1_Uncultured_South China Sea
HQ455905.1_Uncultured_South China Sea
HQ455906.1_Uncultured_South China Sea
HQ455907.1_Uncultured_South China Sea
HQ455908.1_Uncultured_South China Sea
HQ455909.1_Uncultured_South China Sea
HQ455910.1_Uncultured_South China Sea
HQ455913.1_Uncultured_South China Sea
HQ455914.1_Uncultured_South China Sea
HQ455915.1_Uncultured_South China Sea
HQ455917.1_Uncultured_South China Sea

HQ455918.1_Uncultured_South China Sea
HQ455919.1_Uncultured_South China Sea
HQ455920.1_Uncultured_South China Sea
HQ455921.1_Uncultured_South China Sea
HQ455923.1_Uncultured_South China Sea
HQ455929.1_Uncultured_South China Sea
HQ455930.1_Uncultured_South China Sea
HQ455934.1_Uncultured_South China Sea
HQ455940.1_Uncultured_South China Sea
HQ455942.1_Uncultured_South China Sea
HQ455943.1_Uncultured_South China Sea
HQ455944.1_Uncultured_South China Sea
HQ455945.1_Uncultured_South China Sea
HQ455946.1_Uncultured_South China Sea
HQ455951.1_Uncultured_South China Sea
HQ455954.1_Uncultured_South China Sea
HQ455956.1_Uncultured_South China Sea
HQ455957.1_Uncultured_South China Sea
HQ455958.1_Uncultured_South China Sea
HQ455959.1_Uncultured_South China Sea
HQ455960.1_Uncultured_South China Sea
HQ455961.1_Uncultured_South China Sea
HQ455962.1_Uncultured_South China Sea
HQ455964.1_Uncultured_South China Sea
HQ455966.1_Uncultured_South China Sea
HQ455967.1_Uncultured_South China Sea
HQ455968.1_Uncultured_South China Sea
HQ455970.1_Uncultured_South China Sea
HQ455974.1_Uncultured_South China Sea
HQ455977.1_Uncultured_South China Sea
HQ455978.1_Uncultured_South China Sea
HQ455979.1_Uncultured_South China Sea
HQ455980.1_Uncultured_South China Sea
HQ455984.1_Uncultured_South China Sea
HQ455988.1_Uncultured_South China Sea
HQ455989.1_Uncultured_South China Sea
HQ455990.1_Uncultured_South China Sea
HQ455991.1_Uncultured_South China Sea
HQ455992.1_Uncultured_South China Sea
HQ455994.1_Uncultured_South China Sea
HQ455995.1_Uncultured_South China Sea
HQ455996.1_Uncultured_South China Sea
HQ455998.1_Uncultured_South China Sea
HQ455999.1_Uncultured_South China Sea

HQ456001.1_Uncultured_South China Sea
HQ456004.1_Uncultured_South China Sea
HQ456005.1_Uncultured_South China Sea
HQ456006.1_Uncultured_South China Sea
HQ456007.1_Uncultured_South China Sea
HQ456010.1_Uncultured_South China Sea
HQ456012.1_Uncultured_South China Sea
HQ456013.1_Uncultured_South China Sea
HQ456016.1_Uncultured_South China Sea
HQ456017.1_Uncultured_South China Sea
HQ456019.1_Uncultured_South China Sea
HQ456025.1_Uncultured_South China Sea
HQ456026.1_Uncultured_South China Sea
HQ456028.1_Uncultured_South China Sea
HQ456029.1_Uncultured_South China Sea
HQ456030.1_Uncultured_South China Sea
HQ456031.1_Uncultured_South China Sea
HQ456032.1_Uncultured_South China Sea
HQ456033.1_Uncultured_South China Sea
HQ456034.1_Uncultured_South China Sea
HQ456035.1_Uncultured_South China Sea
HQ456037.1_Uncultured_South China Sea
HQ456038.1_Uncultured_South China Sea
HQ456040.1_Uncultured_South China Sea
HQ456041.1_Uncultured_South China Sea
HQ456042.1_Uncultured_South China Sea
HQ456043.1_Uncultured_South China Sea
HQ456044.1_Uncultured_South China Sea
HQ456045.1_Uncultured_South China Sea
HQ456046.1_Uncultured_South China Sea
HQ456047.1_Uncultured_South China Sea
HQ456049.1_Uncultured_South China Sea
HQ456051.1_Uncultured_South China Sea
HQ456053.1_Uncultured_South China Sea
HQ456057.1_Uncultured_South China Sea
HQ456059.1_Uncultured_South China Sea
HQ456060.1_Uncultured_South China Sea
HQ456068.1_Uncultured_South China Sea
HQ456069.1_Uncultured_South China Sea
HQ456071.1_Uncultured_South China Sea
HQ456073.1_Uncultured_South China Sea
HQ456075.1_Uncultured_South China Sea
HQ456079.1_Uncultured_South China Sea
HQ456088.1_Uncultured_South China Sea

HQ456093.1_Uncultured_South China Sea
HQ456095.1_Uncultured_South China Sea
HQ456096.1_Uncultured_South China Sea
HQ456098.1_Uncultured_South China Sea
HQ456099.1_Uncultured_South China Sea
HQ456101.1_Uncultured_South China Sea
HQ456102.1_Uncultured_South China Sea
HQ456103.1_Uncultured_South China Sea
HQ456104.1_Uncultured_South China Sea
HQ456105.1_Uncultured_South China Sea
HQ456106.1_Uncultured_South China Sea
HQ456107.1_Uncultured_South China Sea
HQ456108.1_Uncultured_South China Sea
HQ456109.1_Uncultured_South China Sea
HQ456110.1_Uncultured_South China Sea
HQ456111.1_Uncultured_South China Sea
HQ456112.1_Uncultured_South China Sea
HQ456114.1_Uncultured_South China Sea
HQ456115.1_Uncultured_South China Sea
HQ456116.1_Uncultured_South China Sea
HQ456117.1_Uncultured_South China Sea
HQ456120.1_Uncultured_South China Sea
HQ456121.1_Uncultured_South China Sea
HQ586273.1_Zhang_South China Sea
HQ586274.1_Uncultured_South China Sea
HQ586275.1_Uncultured_South China Sea
HQ586276.1_Uncultured_South China Sea
HQ586286.1_Uncultured_South China Sea
HQ586288.1_Uncultured_South China Sea
HQ586295.1_Uncultured_South China Sea
HQ586299.1_Uncultured_South China Sea
HQ586302.1_Uncultured_South China Sea
HQ586412.1_Uncultured_South China Sea
HQ586414.1_Uncultured_South China Sea
HQ586421.1_Uncultured_South China Sea
HQ586424.1_Uncultured_South China Sea
HQ586425.1_Uncultured_South China Sea
HQ586426.1_Uncultured_South China Sea
HQ586430.1_Uncultured_South China Sea
HQ586432.1_Uncultured_South China Sea
HQ586436.1_Uncultured_South China Sea
HQ586439.1_Uncultured_South China Sea
HQ586440.1_Uncultured_South China Sea
HQ586441.1_Uncultured_South China Sea

HQ586442.1_Uncultured_South China Sea
HQ586447.1_Uncultured_South China Sea
HQ586449.1_Uncultured_South China Sea
HQ586450.1_Uncultured_South China Sea
HQ586451.1_Uncultured_South China Sea
HQ586452.1_Uncultured_South China Sea
HQ586453.1_Uncultured_South China Sea
HQ586455.1_Uncultured_South China Sea
HQ586458.1_Uncultured_South China Sea
HQ586461.1_Uncultured_South China Sea
HQ586462.1_Uncultured_South China Sea
HQ586468.1_Uncultured_South China Sea
HQ586469.1_Uncultured_South China Sea
HQ586482.1_Uncultured_South China Sea
HQ586496.1_Uncultured_South China Sea
HQ586498.1_Uncultured_South China Sea
HQ586507.1_Uncultured_South China Sea
HQ586513.1_Uncultured_South China Sea
HQ586522.1_Uncultured_South China Sea
HQ586540.1_Uncultured_South China Sea
HQ586541.1_Uncultured_South China Sea
HQ586542.1_Uncultured_South China Sea
HQ586543.1_Uncultured_South China Sea
HQ586544.1_Uncultured_South China Sea
HQ586545.1_Uncultured_South China Sea
HQ586546.1_Uncultured_South China Sea
HQ586547.1_Uncultured_South China Sea
HQ586548.1_Uncultured_South China Sea
HQ586549.1_Uncultured_South China Sea
HQ586550.1_Uncultured_South China Sea
HQ586554.1_Uncultured_South China Sea
HQ586555.1_Uncultured_South China Sea
HQ586557.1_Uncultured_South China Sea
HQ586558.1_Uncultured_South China Sea
HQ586559.1_Uncultured_South China Sea
HQ586560.1_Uncultured_South China Sea
HQ586561.1_Uncultured_South China Sea
HQ586562.1_Uncultured_South China Sea
HQ586568.1_Uncultured_South China Sea
HQ586574.1_Uncultured_South China Sea
HQ586575.1_Uncultured_South China Sea
HQ586639.1_Uncultured_South China Sea
HQ586678.1_Uncultured_South China Sea
HQ605990.1_Uncultured_marine

HQ606011.1_Uncultured_marine
HQ606012.1_Uncultured_marine
HQ606013.1_Uncultured_marine
HQ606014.1_Uncultured_marine
HQ611353.1_Uncultured_marine
HQ611354.1_Uncultured_marine
HQ611355.1_Uncultured_marine
HQ611356.1_Uncultured_marine
HQ611357.1_Uncultured_marine
HQ611360.1_Uncultured_marine
HQ611363.1_Uncultured_marine
HQ611365.1_Uncultured_marine
HQ611366.1_Uncultured_marine
HQ611367.1_Uncultured_marine
HQ611369.1_Uncultured_marine
HQ611370.1_Uncultured_marine
HQ611374.1_Uncultured_marine
HQ611376.1_Uncultured_marine
HQ611377.1_Uncultured_marine
HQ611378.1_Uncultured_marine
HQ611379.1_Uncultured_marine
HQ611380.1_Uncultured_marine
HQ611381.1_Uncultured_marine
HQ611383.1_Uncultured_marine
HQ611385.1_Uncultured_marine
HQ611390.1_Uncultured_marine
HQ611392.1_Uncultured_marine
HQ611393.1_Uncultured_marine
HQ611395.1_Uncultured_marine
HQ611396.1_Uncultured_marine
HQ611398.1_Uncultured_marine
HQ611400.1_Uncultured_marine
HQ611404.1_Uncultured_marine
HQ611405.1_Uncultured_marine
HQ611409.1_Uncultured_marine
HQ611413.1_Uncultured_marine
HQ611414.1_Uncultured_marine
HQ611415.1_Uncultured_marine
HQ611417.1_Uncultured_marine
HQ611419.1_Uncultured_marine
HQ611420.1_Uncultured_marine
HQ611421.1_Uncultured_marine
HQ611422.1_Uncultured_marine
HQ611424.1_Uncultured_marine

HQ611426.1_Uncultured_marine
HQ611429.1_Uncultured_marine
HQ611430.1_Uncultured_marine
HQ611431.1_Uncultured_marine
HQ611432.1_Uncultured_marine
HQ611433.1_Uncultured_marine
HQ611435.1_Uncultured_marine
HQ611436.1_Uncultured_marine
HQ611437.1_Uncultured_marine
HQ611438.1_Uncultured_marine
HQ611439.1_Uncultured_marine
HQ611440.1_Uncultured_marine
HQ611442.1_Uncultured_marine
HQ611444.1_Uncultured_marine
HQ611448.1_Uncultured_marine
HQ611454.1_Uncultured_marine
HQ611456.1_Uncultured_marine
HQ611457.1_Uncultured_marine
HQ611460.1_Uncultured_marine
HQ611468.1_Uncultured_marine
HQ611469.1_Uncultured_marine
HQ611472.1_Uncultured_marine
HQ611473.1_Uncultured_marine
HQ611474.1_Uncultured_marine
HQ611480.1_Uncultured_marine
HQ611481.1_Uncultured_marine
HQ611482.1_Uncultured_marine
HQ611485.1_Uncultured_marine
HQ611488.1_Uncultured_marine
HQ611489.1_Uncultured_marine
HQ611490.1_Uncultured_marine
HQ611492.1_Uncultured_marine
HQ611493.1_Uncultured_marine
HQ611494.1_Uncultured_marine
HQ611496.1_Uncultured_marine
HQ611498.1_Uncultured_marine
HQ611499.1_Uncultured_marine
HQ611500.1_Uncultured_marine
HQ611501.1_Uncultured_marine
HQ611502.1_Uncultured_marine
HQ611503.1_Uncultured_marine
HQ611504.1_Uncultured_marine
HQ611505.1_Uncultured_marine
HQ611506.1_Uncultured_marine

HQ611507.1_Uncultured_marine
HQ611509.1_Uncultured_marine
HQ611510.1_Uncultured_marine
HQ611513.1_Uncultured_marine
HQ611515.1_Uncultured_marine
HQ611524.1_Uncultured_marine
HQ611530.1_Uncultured_marine
HQ611537.1_Uncultured_marine
HQ611538.1_Uncultured_marine
HQ611539.1_Uncultured_marine
HQ611543.1_Uncultured_marine
HQ611545.1_Uncultured_marine
HQ611547.1_Uncultured_marine
HQ611548.1_Uncultured_marine
HQ611550.1_Uncultured_marine
HQ611551.1_Uncultured_marine
HQ611553.1_Uncultured_marine
HQ611556.1_Uncultured_marine
HQ611557.1_Uncultured_marine
HQ611564.1_Uncultured_marine
HQ611565.1_Uncultured_marine
HQ611572.1_Uncultured_marine
HQ611575.1_Uncultured_marine
HQ611576.1_Uncultured_marine
HQ611578.1_Uncultured_marine
HQ611579.1_Uncultured_marine
HQ611581.1_Uncultured_marine
HQ611583.1_Uncultured_marine
HQ611585.1_Uncultured_marine
HQ611586.1_Uncultured_marine
HQ611588.1_Uncultured_marine
HQ611590.1_Uncultured_marine
HQ611593.1_Uncultured_marine
HQ611594.1_Uncultured_marine
HQ611600.1_Uncultured_marine
HQ611601.1_Uncultured_marine
HQ611603.1_Uncultured_marine
HQ611604.1_Uncultured_marine
HQ611610.1_Uncultured_marine
HQ611625.1_Uncultured_marine
HQ611627.1_Uncultured_marine
HQ611628.1_Uncultured_marine
HQ611629.1_Uncultured_marine
HQ611636.1_Uncultured_marine

HQ611637.1_Uncultured_marine
HQ611638.1_Uncultured_marine
HQ611641.1_Uncultured_marine
HQ611642.1_Uncultured_marine
HQ611643.1_Uncultured_marine
HQ611644.1_Uncultured_marine
HQ611646.1_Uncultured_marine
HQ611647.1_Uncultured_marine
HQ611649.1_Uncultured_marine
HQ611650.1_Uncultured_marine
HQ611651.1_Uncultured_marine
HQ611652.1_Uncultured_marine
HQ611655.1_Uncultured_marine
HQ611657.1_Uncultured_marine
HQ611662.1_Uncultured_marine
HQ611663.1_Uncultured_marine
HQ611667.1_Uncultured_marine
HQ611668.1_Uncultured_marine
HQ611669.1_Uncultured_marine
HQ611673.1_Uncultured_marine
HQ611675.1_Uncultured_marine
HQ611676.1_Uncultured_marine
HQ611677.1_Uncultured_marine
HQ611678.1_Uncultured_marine
HQ611680.1_Uncultured_marine
HQ611681.1_Uncultured_marine
HQ611683.1_Uncultured_marine
HQ611685.1_Uncultured_marine
HQ611686.1_Uncultured_marine
HQ611688.1_Uncultured_marine
HQ611691.1_Uncultured_marine
HQ611693.1_Uncultured_marine
HQ611695.1_Uncultured_marine
HQ611696.1_Uncultured_marine
HQ611700.1_Uncultured_marine
HQ611708.1_Uncultured_marine
HQ611709.1_Uncultured_marine
HQ611711.1_Uncultured_marine
HQ611712.1_Uncultured_marine
HQ611713.1_Uncultured_marine
HQ611717.1_Uncultured_marine
HQ611721.1_Uncultured_marine
HQ611722.1_Uncultured_marine
HQ611723.1_Uncultured_marine

HQ611725.1_Uncultured_marine
HQ611726.1_Uncultured_marine
HQ611728.1_Uncultured_marine
HQ611730.1_Uncultured_marine
HQ611732.1_Uncultured_marine
HQ611735.1_Uncultured_marine
HQ611737.1_Uncultured_marine
HQ611741.1_Uncultured_marine
HQ611745.1_Uncultured_marine
HQ611747.1_Uncultured_marine
HQ611749.1_Uncultured_marine
HQ611751.1_Uncultured_marine
HQ611753.1_Uncultured_marine
HQ611754.1_Uncultured_marine
HQ611756.1_Uncultured_marine
HQ611760.1_Uncultured_marine
HQ611762.1_Uncultured_marine
HQ611765.1_Uncultured_marine
HQ611767.1_Uncultured_marine
HQ611768.1_Uncultured_marine
HQ611769.1_Uncultured_marine
HQ611773.1_Uncultured_marine
HQ611775.1_Uncultured_marine
HQ611776.1_Uncultured_marine
HQ611777.1_Uncultured_marine
HQ611778.1_Uncultured_marine
HQ611779.1_Uncultured_marine
HQ611780.1_Uncultured_marine
HQ611781.1_Uncultured_marine
HQ611782.1_Uncultured_marine
HQ611785.1_Uncultured_marine
HQ611786.1_Uncultured_marine
HQ611789.1_Uncultured_marine
HQ611790.1_Uncultured_marine
HQ611791.1_Uncultured_marine
HQ611796.1_Uncultured_marine
HQ611799.1_Uncultured_marine
HQ611800.1_Uncultured_marine
HQ611801.1_Uncultured_marine
HQ611802.1_Uncultured_marine
HQ611804.1_Uncultured_marine
HQ611805.1_Uncultured_marine
HQ611810.1_Uncultured_marine
HQ611814.1_Uncultured_marine

HQ611816.1_Uncultured_marine
HQ611817.1_Uncultured_marine
HQ611821.1_Uncultured_marine
HQ611823.1_Uncultured_marine
HQ611824.1_Uncultured_marine
HQ611826.1_Uncultured_marine
HQ611827.1_Uncultured_marine
HQ611829.1_Uncultured_marine
HQ611831.1_Uncultured_marine
HQ611833.1_Uncultured_marine
HQ611835.1_Uncultured_marine
HQ611836.1_Uncultured_marine
HQ611837.1_Uncultured_marine
HQ611838.1_Uncultured_marine
HQ611839.1_Uncultured_marine
HQ611840.1_Uncultured_marine
HQ611844.1_Uncultured_marine
HQ611845.1_Uncultured_marine
HQ611849.1_Uncultured_marine
HQ611850.1_Uncultured_marine
HQ611851.1_Uncultured_marine
HQ611853.1_Uncultured_marine
HQ611855.1_Uncultured_marine
HQ611857.1_Uncultured_marine
HQ611861.1_Uncultured_marine
HQ611862.1_Uncultured_marine
HQ611863.1_Uncultured_marine
HQ611864.1_Uncultured_marine
HQ611865.1_Uncultured_marine
HQ611867.1_Uncultured_marine
HQ611869.1_Uncultured_marine
HQ611872.1_Uncultured_marine
HQ611874.1_Uncultured_marine
HQ611875.1_Uncultured_marine
HQ611878.1_Uncultured_marine
HQ611879.1_Uncultured_marine
HQ611882.1_Uncultured_marine
HQ611883.1_Uncultured_marine
HQ611884.1_Uncultured_marine
HQ611885.1_Uncultured_marine
HQ611887.1_Uncultured_marine
HQ611888.1_Uncultured_marine
HQ611900.1_Uncultured_marine
HQ611901.1_Uncultured_marine

HQ611909.1_Uncultured_marine
HQ611910.1_Uncultured_marine
HQ611911.1_Uncultured_marine
HQ611912.1_Uncultured_marine
HQ611914.1_Uncultured_marine
HQ611918.1_Uncultured_marine
HQ611926.1_Uncultured_marine
HQ611929.1_Uncultured_marine
HQ611931.1_Uncultured_marine
HQ611932.1_Uncultured_marine
HQ611942.1_Uncultured_marine
HQ611943.1_Uncultured_marine
HQ611946.1_Uncultured_marine
HQ611948.1_Uncultured_marine
HQ611953.1_Uncultured_marine
HQ611955.1_Uncultured_marine
HQ630792.1_Uncultured_Mediterranean
HQ630796.1_Uncultured_Mediterranean
HQ630797.1_Uncultured_Mediterranean
HQ630798.1_Uncultured_Mediterranean
HQ634480.1_Uncultured_marine
HQ634484.1_Uncultured_marine
HQ634488.1_Uncultured_marine
HQ634489.1_Uncultured_marine
HQ634490.1_Uncultured_marine
HQ634492.1_Uncultured_marine
HQ634493.1_Uncultured_marine
HQ634494.1_Uncultured_marine
HQ634498.1_Uncultured_marine
HQ634499.1_Uncultured_marine
HQ634501.1_Uncultured_marine
HQ634503.1_Uncultured_marine
HQ634510.1_Uncultured_marine
HQ634511.1_Uncultured_marine
HQ634512.1_Uncultured_marine
HQ634514.1_Uncultured_marine
HQ634515.1_Uncultured_marine
HQ634516.1_Uncultured_marine
HQ660814.1_Uncultured_marine
HQ660827.1_Uncultured_marine
HQ660828.1_Uncultured_marine
HQ660830.1_Uncultured_marine
HQ660831.1_Uncultured_marine
HQ660837.1_Uncultured_marine

HQ660840.1_Uncultured_marine
HQ660841.1_Uncultured_marine
HQ660847.1_Uncultured_marine
HQ660857.1_Uncultured_marine
HQ660860.1_Uncultured_marine
HQ660863.1_Uncultured_marine
HQ660871.1_Uncultured_marine
HQ660877.1_Uncultured_marine
HQ660889.1_Uncultured_marine
HQ660890.1_Uncultured_marine
HQ660894.1_Uncultured_marine
HQ660897.1_Uncultured_marine
HQ660901.1_Uncultured_marine
HQ660919.1_Uncultured_marine
HQ660921.1_Uncultured_marine
HQ660922.1_Uncultured_marine
HQ660930.1_Uncultured_marine
HQ660931.1_Uncultured_marine
HQ660934.1_Uncultured_marine
HQ660936.1_Uncultured_marine
HQ660938.1_Uncultured_marine
HQ691100.1_Uncultured_hot spring
HQ691101.1_Uncultured_hot spring
HQ691102.1_Uncultured_hot spring
HQ901775.1_Microbacterium
J01740.1_klebsiella
JF429940.1_Uncultured_marine
JF429957.1_Uncultured_marine
JF429958.1_Uncultured_marine
JF429965.1_Uncultured_marine
JF429967.1_Uncultured_marine
JF701923.1_Alteromonadales_cryopeg
JF826486.1_Uncultured_stromatolite
JF826487.1_Uncultured_stromatolite
JF896643.1_Uncultured_marine seagrass
JF896645.1_Uncultured_marine seagrass
JF896652.1_Uncultured_marine seagrass
JF896653.1_Uncultured_marine seagrass
JF896654.1_Uncultured_marine seagrass
JF896656.1_Uncultured_marine seagrass
JF896662.1_Uncultured_marine seagrass
JF896663.1_Uncultured_marine seagrass
JF896664.1_Uncultured_marine seagrass
JF896665.1_Uncultured_marine seagrass

JF896667.1_Uncultured_marine seagrass
JF896668.1_Uncultured_marine seagrass
JF896669.1_Uncultured_marine seagrass
JF896670.1_Uncultured_marine seagrass
JF896671.1_Uncultured_marine seagrass
JF896680.1_Uncultured_marine seagrass
JF896681.1_Uncultured_marine seagrass
JF896683.1_Uncultured_marine seagrass
JF896684.1_Uncultured_marine seagrass
JF896685.1_Uncultured_marine seagrass
JF896686.1_Uncultured_marine seagrass
JF896687.1_Uncultured_marine seagrass
JF896688.1_Uncultured_marine seagrass
JF896690.1_Uncultured_marine seagrass
JF896691.1_Uncultured_marine seagrass
JF896692.1_Uncultured_marine seagrass
JF896694.1_Uncultured_marine seagrass
JF896695.1_Uncultured_marine seagrass
JF896875.1_Uncultured_marine mat
JF896876.1_Uncultured_marine mat
JF896880.1_Uncultured_marine mat
JF896882.1_Uncultured_marine mat
JF896897.1_Uncultured_marine mat
JF896899.1_Uncultured_marine mat
JF896902.1_Uncultured_marine mat
JF896903.1_Uncultured_marine mat
JF896904.1_Uncultured_marine mat
JF896906.1_Uncultured_marine mat
JF896913.1_Uncultured_marine mat
JF896930.1_Uncultured_marine mat
JF896933.1_Uncultured_marine mat
JF896935.1_Uncultured_marine mat
JF896945.1_Uncultured_marine mat
JF896948.1_Uncultured_marine mat
JF896950.1_Uncultured_marine mat
JF896954.1_Uncultured_marine mat
JF896962.1_Uncultured_marine mat
JF896963.1_Uncultured_marine mat
JF896964.1_Uncultured_marine mat
JF896976.1_Uncultured_marine mat
JF896991.1_Uncultured_marine mat
JF896993.1_Uncultured_marine mat
JF896994.1_Uncultured_marine mat
JF896996.1_Uncultured_marine mat

JF896997.1_Uncultured_marine mat
JF897002.1_Uncultured_marine mat
JF897006.1_Uncultured_marine mat
JF897011.1_Uncultured_marine mat
JF897012.1_Uncultured_marine mat
JF897017.1_Uncultured_marine mat
JF897025.1_Uncultured_marine mat
JF897038.1_Uncultured_marine mat
JF897042.1_Uncultured_marine mat
JF897045.1_Uncultured_marine mat
JF897047.1_Uncultured_marine mat
JF897052.1_Uncultured_marine mat
JF897055.1_Uncultured_marine mat
JF897056.1_Uncultured_marine mat
JF897057.1_Uncultured_marine mat
JF897059.1_Uncultured_marine mat
JF897061.1_Uncultured_marine mat
JF897062.1_Uncultured_marine mat
JF897064.1_Uncultured_marine mat
JF897065.1_Uncultured_marine mat
JF897066.1_Uncultured_marine mat
JF897067.1_Uncultured_marine mat
JF897069.1_Uncultured_marine mat
JF897070.1_Uncultured_marine mat
JF897073.1_Uncultured_marine mat
JF897074.1_Uncultured_marine mat
JF897075.1_Uncultured_marine mat
JF897076.1_Uncultured_marine mat
JF897077.1_Uncultured_marine mat
JF897078.1_Uncultured_marine mat
JF897079.1_Uncultured_marine mat
JF897080.1_Uncultured_marine mat
JF897081.1_Uncultured_marine mat
JF897082.1_Uncultured_marine mat
JF897083.1_Uncultured_marine mat
JF897085.1_Uncultured_marine mat
JF897086.1_Uncultured_marine mat
JF897088.1_Uncultured_marine mat
JF897089.1_Uncultured_marine mat
JF897090.1_Uncultured_marine mat
JF897092.1_Uncultured_marine mat
JF897093.1_Uncultured_marine mat
JF897096.1_Uncultured_marine mat
JF897097.1_Uncultured_marine mat

JF897098.1_Uncultured_marine mat
JF897099.1_Uncultured_marine mat
JF897100.1_Uncultured_marine mat
JF897101.1_Uncultured_marine mat
JF897102.1_Uncultured_marine mat
JF897103.1_Uncultured_marine mat
JF897104.1_Uncultured_marine mat
JF897105.1_Uncultured_marine mat
JF897106.1_Uncultured_marine mat
JF897107.1_Uncultured_marine mat
JF897108.1_Uncultured_marine mat
JF897109.1_Uncultured_marine mat
JF897111.1_Uncultured_marine mat
JF897114.1_Uncultured_marine mat
JF897115.1_Uncultured_marine mat
JF897117.1_Uncultured_marine mat
JF897118.1_Uncultured_marine mat
JF897120.1_Uncultured_marine mat
JF897122.1_Uncultured_marine mat
JF897123.1_Uncultured_marine mat
JF897124.1_Uncultured_marine mat
JF897125.1_Uncultured_marine mat
JF897126.1_Uncultured_marine mat
JF897127.1_Uncultured_marine mat
JF897128.1_Uncultured_marine mat
JF897131.1_Uncultured_marine mat
JF897132.1_Uncultured_marine mat
JF897133.1_Uncultured_marine mat
JF897134.1_Uncultured_marine mat
JF897135.1_Uncultured_marine mat
JF897136.1_Uncultured_marine mat
JF897137.1_Uncultured_marine mat
JF897138.1_Uncultured_marine mat
JF897139.1_Uncultured_marine mat
JF897140.1_Uncultured_marine mat
JF897141.1_Uncultured_marine mat
JF897142.1_Uncultured_marine mat
JF897143.1_Uncultured_marine mat
JF897144.1_Uncultured_marine mat
JF897145.1_Uncultured_marine mat
JF897146.1_Uncultured_marine mat
JF897147.1_Uncultured_marine mat
JF897148.1_Uncultured_marine mat
JF897149.1_Uncultured_marine mat

JF897150.1_Uncultured_marine mat
JF897151.1_Uncultured_marine mat
JF897152.1_Uncultured_marine mat
JF897153.1_Uncultured_marine mat
JF897154.1_Uncultured_marine mat
JF897155.1_Uncultured_marine mat
JF897156.1_Uncultured_marine mat
JF897157.1_Uncultured_marine mat
JF897158.1_Uncultured_marine mat
JF897159.1_Uncultured_marine mat
JF897160.1_Uncultured_marine mat
JF897161.1_Uncultured_marine mat
JF897162.1_Uncultured_marine mat
JF897163.1_Uncultured_marine mat
JF897164.1_Uncultured_marine mat
JF897165.1_Uncultured_marine mat
JF897166.1_Uncultured_marine mat
JF897167.1_Uncultured_marine mat
JF897168.1_Uncultured_marine mat
JF897169.1_Uncultured_marine mat
JF897170.1_Uncultured_marine mat
JF897171.1_Uncultured_marine mat
JF897172.1_Uncultured_marine mat
JF897173.1_Uncultured_marine mat
JF897175.1_Uncultured_marine mat
JF897176.1_Uncultured_marine mat
JF897177.1_Uncultured_marine mat
JF897178.1_Uncultured_marine mat
JF897179.1_Uncultured_marine mat
JF897180.1_Uncultured_marine mat
JF897181.1_Uncultured_marine mat
JF897182.1_Uncultured_marine mat
JF897184.1_Uncultured_marine mat
JF897185.1_Uncultured_marine mat
JF897186.1_Uncultured_marine mat
JF897187.1_Uncultured_marine mat
JF897188.1_Uncultured_marine mat
JF897190.1_Uncultured_marine mat
JF897191.1_Uncultured_marine mat
JF897192.1_Uncultured_marine mat
JF897193.1_Uncultured_marine mat
JF897194.1_Uncultured_marine mat
JF897195.1_Uncultured_marine mat
JF897196.1_Uncultured_marine mat

JF897197.1_Uncultured_marine mat
JF897198.1_Uncultured_marine mat
JF897199.1_Uncultured_marine mat
JF897200.1_Uncultured_marine mat
JF897201.1_Uncultured_marine mat
JF897202.1_Uncultured_marine mat
JF897203.1_Uncultured_marine mat
JF897204.1_Uncultured_marine mat
JF897205.1_Uncultured_marine mat
JF897206.1_Uncultured_marine mat
JF897207.1_Uncultured_marine mat
JF897208.1_Uncultured_marine mat
JF897209.1_Uncultured_marine mat
JF897210.1_Uncultured_marine mat
JF897211.1_Uncultured_marine mat
JF897212.1_Uncultured_marine mat
JF897213.1_Uncultured_marine mat
JF897214.1_Uncultured_marine mat
JF897215.1_Uncultured_marine mat
JF897216.1_Uncultured_marine mat
JF897217.1_Uncultured_marine mat
JF897218.1_Uncultured_marine mat
JF897219.1_Uncultured_marine mat
JF897220.1_Uncultured_marine mat
JF897221.1_Uncultured_marine mat
JF897222.1_Uncultured_marine mat
JF897223.1_Uncultured_marine mat
JF897224.1_Uncultured_marine mat
JF897225.1_Uncultured_marine mat
JF897226.1_Uncultured_marine mat
JF897227.1_Uncultured_marine mat
JF897228.1_Uncultured_marine mat
JF897229.1_Uncultured_marine mat
JF897230.1_Uncultured_marine mat
JF897231.1_Uncultured_marine mat
JF897239.1_Uncultured_marine mat
JF897240.1_Uncultured_marine mat
JF897241.1_Uncultured_marine mat
JF897243.1_Uncultured_marine mat
JF897246.1_Uncultured_marine mat
JF897248.1_Uncultured_marine mat
JF897255.1_Uncultured_marine mat
JF897259.1_Uncultured_marine mat
JF897260.1_Uncultured_marine mat

JF897281.1_Uncultured_marine mat
JF897284.1_Uncultured_marine mat
JF897286.1_Uncultured_marine mat
JF897294.1_Uncultured_marine mat
JF897303.1_Uncultured_marine mat
JF897309.1_Uncultured_marine mat
JF897314.1_Uncultured_marine mat
JF897321.1_Uncultured_marine mat
JF897326.1_Uncultured_marine mat
JF897330.1_Uncultured_marine mat
JF897331.1_Uncultured_marine mat
JF897332.1_Uncultured_marine mat
JF897333.1_Uncultured_marine mat
JF897335.1_Uncultured_marine mat
JF897337.1_Uncultured_marine mat
JF897338.1_Uncultured_marine mat
JF897339.1_Uncultured_marine mat
JF897341.1_Uncultured_marine mat
JF897343.1_Uncultured_marine mat
JF897344.1_Uncultured_marine mat
JF897346.1_Uncultured_marine mat
JF897349.1_Uncultured_marine mat
JF897352.1_Uncultured_marine mat
JF897353.1_Uncultured_marine mat
JF897354.1_Uncultured_marine mat
JF897355.1_Uncultured_marine mat
JF897357.1_Uncultured_marine mat
JF897360.1_Uncultured_marine mat
JF897362.1_Uncultured_marine mat
JF897364.1_Uncultured_marine mat
JF897366.1_Uncultured_marine mat
JF897367.1_Uncultured_marine mat
JF897369.1_Uncultured_marine mat
JF897370.1_Uncultured_marine mat
JF897372.1_Uncultured_marine mat
JF897373.1_Uncultured_marine mat
JF897374.1_Uncultured_marine mat
JF897375.1_Uncultured_marine mat
JF897380.1_Uncultured_marine mat
JF897381.1_Uncultured_marine mat
JF897383.1_Uncultured_marine mat
JF897385.1_Uncultured_marine mat
JF897386.1_Uncultured_marine mat
JF897387.1_Uncultured_marine mat

JF897388.1_Uncultured_marine mat
JF897392.1_Uncultured_marine mat
JF897393.1_Uncultured_marine mat
JF897396.1_Uncultured_marine mat
JF897397.1_Uncultured_marine mat
JF897400.1_Uncultured_marine mat
JF897405.1_Uncultured_marine mat
JF897407.1_Uncultured_marine mat
JF897415.1_Uncultured_marine mat
JF897419.1_Uncultured_marine mat
JF897431.1_Uncultured_marine mat
JF897436.1_Uncultured_marine mat
JF897438.1_Uncultured_marine mat
JF897440.1_Uncultured_marine mat
JF897443.1_Uncultured_marine mat
JF897448.1_Uncultured_marine mat
JF897460.1_Uncultured_marine mat
JF897461.1_Uncultured_marine mat
JF897465.1_Uncultured_marine mat
JF897470.1_Uncultured_marine mat
JF897472.1_Uncultured_marine mat
JF897479.1_Uncultured_marine mat
JF897485.1_Uncultured_marine mat
JF897494.1_Uncultured_marine mat
JF897504.1_Uncultured_marine mat
JF897511.1_Uncultured_marine mat
JF897517.1_Uncultured_marine mat
JF897519.1_Uncultured_marine mat
JF897522.1_Uncultured_marine mat
JF897523.1_Uncultured_marine mat
JF897524.1_Uncultured_marine mat
JF897525.1_Uncultured_marine mat
JF897527.1_Uncultured_marine mat
JF897530.1_Uncultured_marine mat
JF897538.1_Uncultured_marine mat
JF897546.1_Uncultured_marine mat
JF897548.1_Uncultured_marine mat
JF897552.1_Uncultured_marine mat
JF897555.1_Uncultured_marine mat
JF897558.1_Uncultured_marine mat
JF897561.1_Uncultured_marine mat
JF897565.1_Uncultured_marine mat
JF897566.1_Uncultured_marine mat
JF897579.1_Uncultured_marine mat

JF897580.1_Uncultured_marine mat
JF897587.1_Uncultured_marine mat
JF897596.1_Uncultured_marine mat
JF897599.1_Uncultured_marine mat
JN093533.1_Uncultured_South China Sea
JN093539.1_Uncultured_South China Sea
JN093541.1_Uncultured_South China Sea
JN093547.1_Uncultured_South China Sea
JN093592.1_Uncultured_South China Sea
JN093600.1_Uncultured_South China Sea
JN093605.1_Uncultured_South China Sea
JN093607.1_Uncultured_South China Sea
JN093608.1_Uncultured_South China Sea
JN093610.1_Uncultured_South China Sea
JN093615.1_Uncultured_South China Sea
JN093644.1_Uncultured_South China Sea
JN093649.1_Uncultured_South China Sea
JN093661.1_Uncultured_South China Sea
JN093670.1_Uncultured_South China Sea
JN093696.1_Uncultured_South China Sea
JN093701.1_Uncultured_South China Sea
JN093739.1_Uncultured_South China Sea
JN093745.1_Uncultured_South China Sea
JN093765.1_Uncultured_South China Sea
JN093774.1_Uncultured_South China Sea
JN093779.1_Uncultured_South China Sea
JN093797.1_Uncultured_South China Sea
JN093818.1_Uncultured_South China Sea
JN093825.1_Uncultured_South China Sea
JN093878.1_Uncultured_South China Sea
JN093880.1_Uncultured_South China Sea
JN093882.1_Uncultured_South China Sea
JN093895.1_Uncultured_South China Sea
JN093898.1_Uncultured_South China Sea
JN093900.1_Uncultured_South China Sea
JN093910.1_Uncultured_South China Sea
JN093913.1_Uncultured_South China Sea
JN093917.1_Uncultured_South China Sea
JN093919.1_Uncultured_South China Sea
JN093923.1_Uncultured_South China Sea
JN093931.1_Uncultured_South China Sea
JN093933.1_Uncultured_South China Sea
JN093937.1_Uncultured_South China Sea
JN093939.1_Uncultured_South China Sea

JN093941.1_Uncultured_South China Sea
JN093950.1_Uncultured_South China Sea
JN093951.1_Uncultured_South China Sea
JN093953.1_Uncultured_South China Sea
JN093961.1_Uncultured_South China Sea
JN093968.1_Uncultured_South China Sea
JN094001.1_Uncultured_South China Sea
JN094003.1_Uncultured_South China Sea
JN094017.1_Uncultured_South China Sea
JN094029.1_Uncultured_South China Sea
JN094042.1_Uncultured_South China Sea
JN094044.1_Uncultured_South China Sea
JN094053.1_Uncultured_South China Sea
JN094065.1_Uncultured_South China Sea
JN094072.1_Uncultured_South China Sea
JN094126.1_Uncultured_South China Sea
JN094138.1_Uncultured_South China Sea
JN094146.1_Uncultured_South China Sea
JN094156.1_Uncultured_South China Sea
JN094200.1_Uncultured_South China Sea
JN094209.1_Uncultured_South China Sea
JN094223.1_Uncultured_South China Sea
JN094235.1_Uncultured_South China Sea
JN094238.1_Uncultured_South China Sea
JN094263.1_Uncultured_South China Sea
JN094279.1_Uncultured_South China Sea
JN094287.1_Uncultured_South China Sea
JN094289.1_Uncultured_South China Sea
JN094292.1_Uncultured_South China Sea
JN094296.1_Uncultured_South China Sea
JN094298.1_Uncultured_South China Sea
JN094304.1_Uncultured_South China Sea
JN094306.1_Uncultured_South China Sea
JN094308.1_Uncultured_South China Sea
JN094314.1_Uncultured_South China Sea
JN094317.1_Uncultured_South China Sea
JN094339.1_Uncultured_South China Sea
JN094347.1_Uncultured_South China Sea
JN094364.1_Uncultured_South China Sea
JN094369.1_Uncultured_South China Sea
JN094387.1_Uncultured_South China Sea
JN094398.1_Uncultured_South China Sea
JN094411.1_Uncultured_South China Sea
JN094433.1_Uncultured_South China Sea

JN094434.1_Uncultured_South China Sea
JN094450.1_Uncultured_South China Sea
JN094463.1_Uncultured_South China Sea
JN094468.1_Uncultured_South China Sea
JN094502.1_Uncultured_South China Sea
JN094529.1_Uncultured_South China Sea
JN094534.1_Uncultured_South China Sea
JN094544.1_Uncultured_South China Sea
JN094551.1_Uncultured_South China Sea
JN094553.1_Uncultured_South China Sea
JN094573.1_Uncultured_South China Sea
JN094578.1_Uncultured_South China Sea
JN094606.1_Uncultured_South China Sea
JN094619.1_Uncultured_South China Sea
JN094650.1_Uncultured_South China Sea
JN094651.1_Uncultured_South China Sea
JN094658.1_Uncultured_South China Sea
JN094666.1_Uncultured_South China Sea
JN094678.1_Uncultured_South China Sea
JN094680.1_Uncultured_South China Sea
JN094692.1_Uncultured_South China Sea
JN094706.1_Uncultured_South China Sea
JN094721.1_Uncultured_South China Sea
JN094727.1_Uncultured_South China Sea
JN094738.1_Uncultured_South China Sea
JN094747.1_Uncultured_South China Sea
JN094755.1_Uncultured_South China Sea
JN094764.1_Uncultured_South China Sea
JN094766.1_Uncultured_South China Sea
JN094787.1_Uncultured_South China Sea
JN094812.1_Uncultured_South China Sea
JN094857.1_Uncultured_South China Sea
JN094862.1_Uncultured_South China Sea
JN094877.1_Uncultured_South China Sea
JN094917.1_Uncultured_South China Sea
JN094920.1_Uncultured_South China Sea
JN094954.1_Uncultured_South China Sea
JN095008.1_Uncultured_South China Sea
JN095011.1_Uncultured_South China Sea
JN095022.1_Uncultured_South China Sea
JN095027.1_Uncultured_South China Sea
JN095035.1_Uncultured_South China Sea
JN095044.1_Uncultured_South China Sea
JN095050.1_Uncultured_South China Sea

JN095061.1_Uncultured_South China Sea
JN095080.1_Uncultured_South China Sea
JN095081.1_Uncultured_South China Sea
JN095094.1_Uncultured_South China Sea
JN095133.1_Uncultured_South China Sea
JN095163.1_Uncultured_South China Sea
JN095194.1_Uncultured_South China Sea
JN095236.1_Uncultured_South China Sea
JN095267.1_Uncultured_South China Sea
JN095312.1_Uncultured_South China Sea
JN095320.1_Uncultured_South China Sea
JN095332.1_Uncultured_South China Sea
JN095337.1_Uncultured_South China Sea
JN095356.1_Uncultured_South China Sea
JN095370.1_Uncultured_South China Sea
JN095392.1_Uncultured_South China Sea
JN095413.1_Uncultured_South China Sea
JN095414.1_Uncultured_South China Sea
JN095421.1_Uncultured_South China Sea
JN095422.1_Uncultured_South China Sea
JN095424.1_Uncultured_South China Sea
JN095435.1_Uncultured_South China Sea
JN095454.1_Uncultured_South China Sea
JN095469.1_Uncultured_South China Sea
JN095484.1_Uncultured_South China Sea
JN095496.1_Uncultured_South China Sea
JN095499.1_Uncultured_South China Sea
JN095501.1_Uncultured_South China Sea
JN095503.1_Uncultured_South China Sea
JN095504.1_Uncultured_South China Sea
JN095518.1_Uncultured_South China Sea
JN095550.1_Uncultured_South China Sea
JN095553.1_Uncultured_South China Sea
JN095572.1_Uncultured_South China Sea
JN095608.1_Uncultured_South China Sea
JN095609.1_Uncultured_South China Sea
JN095619.1_Uncultured_South China Sea
JN095626.1_Uncultured_South China Sea
JN095636.1_Uncultured_South China Sea
JN095641.1_Uncultured_South China Sea
JN095654.1_Uncultured_South China Sea
JN095657.1_Uncultured_South China Sea
JN095658.1_Uncultured_South China Sea
JN095662.1_Uncultured_South China Sea

JN095673.1_Uncultured_South China Sea
JN095674.1_Uncultured_South China Sea
JN095685.1_Uncultured_South China Sea
JN095686.1_Uncultured_South China Sea
JN095691.1_Uncultured_South China Sea
JN095706.1_Uncultured_South China Sea
JN095715.1_Uncultured_South China Sea
JN095719.1_Uncultured_South China Sea
JN095720.1_Uncultured_South China Sea
JN095721.1_Uncultured_South China Sea
JN095724.1_Uncultured_South China Sea
JN095726.1_Uncultured_South China Sea
JN095747.1_Uncultured_South China Sea
JN095755.1_Uncultured_South China Sea
JN095758.1_Uncultured_South China Sea
JN095764.1_Uncultured_South China Sea
JN095775.1_Uncultured_South China Sea
JN095776.1_Uncultured_South China Sea
JN095779.1_Uncultured_South China Sea
JN095784.1_Uncultured_South China Sea
JN095795.1_Uncultured_South China Sea
JN095800.1_Uncultured_South China Sea
JN095805.1_Uncultured_South China Sea
JN095813.1_Uncultured_South China Sea
JN095822.1_Uncultured_South China Sea
JN095843.1_Uncultured_South China Sea
JN095844.1_Uncultured_South China Sea
JN095846.1_Uncultured_South China Sea
JN095875.1_Uncultured_South China Sea
JN095929.1_Uncultured_South China Sea
JN095998.1_Uncultured_South China Sea
JN096001.1_Uncultured_South China Sea
JN096008.1_Uncultured_South China Sea
JN096037.1_Uncultured_South China Sea
JN096061.1_Uncultured_South China Sea
JN096079.1_Uncultured_South China Sea
JN096090.1_Uncultured_South China Sea
JN096108.1_Uncultured_South China Sea
JN096133.1_Uncultured_South China Sea
JN096134.1_Uncultured_South China Sea
JN096161.1_Uncultured_South China Sea
JN096163.1_Uncultured_South China Sea
JN096184.1_Uncultured_South China Sea
JN096189.1_Uncultured_South China Sea

JN096197.1_Uncultured_South China Sea
JN096216.1_Uncultured_South China Sea
JN096242.1_Uncultured_South China Sea
JN096245.1_Uncultured_South China Sea
JN096263.1_Uncultured_South China Sea
JN096341.1_Uncultured_South China Sea
JN096357.1_Uncultured_South China Sea
JN096388.1_Uncultured_South China Sea
JN096397.1_Uncultured_South China Sea
JN096413.1_Uncultured_South China Sea
JN096416.1_Uncultured_South China Sea
JN096421.1_Uncultured_South China Sea
JN096424.1_Uncultured_South China Sea
JN096426.1_Uncultured_South China Sea
JN096432.1_Uncultured_South China Sea
JN096461.1_Uncultured_South China Sea
JN096467.1_Uncultured_South China Sea
JN096510.1_Uncultured_South China Sea
JN096557.1_Uncultured_South China Sea
JN096570.1_Uncultured_South China Sea
JN096571.1_Uncultured_South China Sea
JN096575.1_Uncultured_South China Sea
JN096578.1_Uncultured_South China Sea
JN096583.1_Uncultured_South China Sea
JN096588.1_Uncultured_South China Sea
JN096592.1_Uncultured_South China Sea
JN096614.1_Uncultured_South China Sea
JN096656.1_Uncultured_South China Sea
JN096665.1_Uncultured_South China Sea
JN096666.1_Uncultured_South China Sea
JN096709.1_Uncultured_South China Sea
JN096711.1_Uncultured_South China Sea
JN096739.1_Uncultured_South China Sea
JN096740.1_Uncultured_South China Sea
JN096775.1_Uncultured_South China Sea
JN096779.1_Uncultured_South China Sea
JN096803.1_Uncultured_South China Sea
JN096805.1_Uncultured_South China Sea
JN096809.1_Uncultured_South China Sea
JN096826.1_Uncultured_South China Sea
JN096841.1_Uncultured_South China Sea
JN096877.1_Uncultured_South China Sea
JN096899.1_Uncultured_South China Sea
JN096908.1_Uncultured_South China Sea

JN096914.1_Uncultured_South China Sea
JN096922.1_Uncultured_South China Sea
JN096924.1_Uncultured_South China Sea
JN096935.1_Uncultured_South China Sea
JN096938.1_Uncultured_South China Sea
JN096952.1_Uncultured_South China Sea
JN096964.1_Uncultured_South China Sea
JN096966.1_Uncultured_South China Sea
JN096972.1_Uncultured_South China Sea
JN097006.1_Uncultured_South China Sea
JN097047.1_Uncultured_South China Sea
JN097052.1_Uncultured_South China Sea
JN097061.1_Uncultured_South China Sea
JN097069.1_Uncultured_South China Sea
JN097084.1_Uncultured_South China Sea
JN097116.1_Uncultured_South China Sea
JN097119.1_Uncultured_South China Sea
JN097122.1_Uncultured_South China Sea
JN097172.1_Uncultured_South China Sea
JN097192.1_Uncultured_South China Sea
JN097214.1_Uncultured_South China Sea
JN097225.1_Uncultured_South China Sea
JN097227.1_Uncultured_South China Sea
JN097234.1_Uncultured_South China Sea
JN097248.1_Uncultured_South China Sea
JN097264.1_Uncultured_South China Sea
JN097267.1_Uncultured_South China Sea
JN097327.1_Uncultured_South China Sea
JN097346.1_Uncultured_South China Sea
JN097347.1_Uncultured_South China Sea
JN097348.1_Uncultured_South China Sea
JN097349.1_Uncultured_South China Sea
JN097350.1_Uncultured_South China Sea
JN097351.1_Uncultured_South China Sea
JN097352.1_Uncultured_South China Sea
JN097353.1_Uncultured_South China Sea
JN097356.1_Uncultured_South China Sea
JN097358.1_Uncultured_South China Sea
JN097359.1_Uncultured_South China Sea
JN097360.1_Uncultured_South China Sea
JN097362.1_Uncultured_South China Sea
JN097363.1_Uncultured_South China Sea
JN097365.1_Uncultured_South China Sea
JN097366.1_Uncultured_South China Sea

JN097367.1_Uncultured_South China Sea
JN097368.1_Uncultured_South China Sea
JN097369.1_Uncultured_South China Sea
JN097370.1_Uncultured_South China Sea
JN097371.1_Uncultured_South China Sea
JN097373.1_Uncultured_South China Sea
JN097374.1_Uncultured_South China Sea
JN097376.1_Uncultured_South China Sea
JN097379.1_Uncultured_South China Sea
JN097380.1_Uncultured_South China Sea
JN097381.1_Uncultured_South China Sea
JN097382.1_Uncultured_South China Sea
JN097383.1_Uncultured_South China Sea
JN097384.1_Uncultured_South China Sea
JN097385.1_Uncultured_South China Sea
JN097386.1_Uncultured_South China Sea
JN097388.1_Uncultured_South China Sea
JN097391.1_Uncultured_South China Sea
JN097394.1_Uncultured_South China Sea
JN097395.1_Uncultured_South China Sea
JN097396.1_Uncultured_South China Sea
JN097397.1_Uncultured_South China Sea
JN097401.1_Uncultured_South China Sea
JN097413.1_Uncultured_South China Sea
JN097414.1_Uncultured_South China Sea
JN097417.1_Uncultured_South China Sea
JN097418.1_Uncultured_South China Sea
JN097420.1_Uncultured_South China Sea
JN097425.1_Uncultured_South China Sea
JN097427.1_Uncultured_South China Sea
JN097428.1_Uncultured_South China Sea
JN097434.1_Uncultured_South China Sea
JN097440.1_Uncultured_South China Sea
JN097441.1_Uncultured_South China Sea
JN097443.1_Uncultured_South China Sea
JN097446.1_Uncultured_South China Sea
JN097448.1_Uncultured_South China Sea
JN097449.1_Uncultured_South China Sea
JN097450.1_Uncultured_South China Sea
JN097452.1_Uncultured_South China Sea
JN122964.1_Uncultured_soil
JN122970.1_Uncultured_soil
JN122971.1_Uncultured_soil
JN122972.1_Uncultured_soil

JN122973.1_Uncultured_soil
JN122974.1_Uncultured_soil
JN122975.1_Uncultured_soil
JN122976.1_Uncultured_soil
JN162481.1_Uncultured_soil
JN162489.1_Uncultured_soil
JN162496.1_Uncultured_soil
JN386977.1_Methylococcaceae
JN600442.1_Pseudomonas_root
JN601397.1_Uncultured_marine
JN601406.1_Uncultured_mucus
JN601407.1_Uncultured_tissue
JN601408.1_Uncultured_tissue
JN601416.1_Uncultured_marine
JN601419.1_Uncultured_marine
JN638619.1_Uncultured_Black Sea
JN638620.1_Uncultured_Black Sea
JN638621.1_Uncultured_Black Sea
JN638622.1_Uncultured_Black Sea
JN638623.1_Uncultured_Black Sea
JN638624.1_Uncultured_Black Sea
JN638625.1_Uncultured_Black Sea
JN638626.1_Uncultured_Black Sea
JN638627.1_Uncultured_Black Sea
JN638628.1_Uncultured_Black Sea
JN638629.1_Uncultured_Black Sea
JN638630.1_Uncultured_Black Sea
JN638631.1_Uncultured_Black Sea
JN638632.1_Uncultured_Black Sea
JN638633.1_Uncultured_Black Sea
JN638634.1_Uncultured_Black Sea
JN638635.1_Uncultured_Black Sea
JN638636.1_Uncultured_Black Sea
JN638637.1_Uncultured_Black Sea
JN638638.1_Uncultured_Black Sea
JN638639.1_Uncultured_Black Sea
JN638640.1_Uncultured_Black Sea
JN638641.1_Uncultured_Black Sea
JN638642.1_Uncultured_Black Sea
JN638643.1_Uncultured_Black Sea
JN638644.1_Uncultured_Black Sea
JN638645.1_Uncultured_Black Sea
JN638646.1_Uncultured_Black Sea
JN638672.1_Uncultured_Black Sea

JN638673.1_Uncultured_Black Sea
JN638674.1_Uncultured_Black Sea
JN645307.1_Uncultured_marine sediment
JN645308.1_Uncultured_marine sediment
JN645309.1_Uncultured_marine sediment
JN645310.1_Uncultured_marine sediment
JN645311.1_Uncultured_marine sediment
JN645312.1_Uncultured_marine sediment
JN645313.1_Uncultured_marine sediment
JN645314.1_Uncultured_marine sediment
JN645315.1_Uncultured_marine sediment
JN645316.1_Uncultured_marine sediment
JN645317.1_Uncultured_marine sediment
JN645318.1_Uncultured_marine sediment
JN645319.1_Uncultured_marine sediment
JN645330.1_Uncultured_marine sediment
JN645331.1_Uncultured_marine sediment
JN645332.1_Uncultured_marine sediment
JN645333.1_Uncultured_marine sediment
JN645338.1_Uncultured_marine sediment
JN645340.1_Uncultured_marine sediment
JN645341.1_Uncultured_marine sediment
JN645343.1_Uncultured_marine sediment
JN645347.1_Uncultured_marine sediment
JN645348.1_Uncultured_marine sediment
JN645349.1_Uncultured_marine sediment
JN645351.1_Uncultured_marine sediment
JN645352.1_Uncultured_marine sediment
JN645353.1_Uncultured_marine sediment
JN645354.1_Uncultured_marine sediment
JN645355.1_Uncultured_marine sediment
JN645360.1_Uncultured_marine sediment
JN645366.1_Uncultured_marine sediment
JN645369.1_Uncultured_marine sediment
JN645370.1_Uncultured_marine sediment
JN645371.1_Uncultured_marine sediment
JN645390.1_Uncultured_marine sediment
JN645395.1_Uncultured_marine sediment
JN645398.1_Uncultured_marine sediment
JN645399.1_Uncultured_marine sediment
JN645409.1_Uncultured_marine sediment
JN645410.1_Uncultured_marine sediment
JN645411.1_Uncultured_marine sediment
JN645414.1_Uncultured_marine sediment

JN645415.1_Uncultured_marine sediment
JN645418.1_Uncultured_marine sediment
JN645428.1_Uncultured_marine sediment
JN645442.1_Uncultured_marine sediment
JN645443.1_Uncultured_marine sediment
JN645445.1_Uncultured_marine sediment
JN645448.1_Uncultured_marine sediment
JN645449.1_Uncultured_marine sediment
JN645450.1_Uncultured_marine sediment
JN645454.1_Uncultured_marine sediment
JN645455.1_Uncultured_marine sediment
JN645457.1_Uncultured_marine sediment
JN645458.1_Uncultured_marine sediment
JN645459.1_Uncultured_marine sediment
JN645471.1_Uncultured_marine sediment
JN645474.1_Uncultured_marine sediment
JN645475.1_Uncultured_marine sediment
JN645476.1_Uncultured_marine sediment
JN645480.1_Uncultured_marine sediment
JN645481.1_Uncultured_marine sediment
JN645490.1_Uncultured_marine sediment
JN648828.1_Uncultured_soil
JN648829.1_Uncultured_soil
JN648830.1_Uncultured_soil
JN648831.1_Uncultured_soil
JN648832.1_Uncultured_soil
JN648836.1_Uncultured_soil
JN648837.1_Uncultured_soil
JN648838.1_Uncultured_soil
JN648839.1_Uncultured_soil
JN648840.1_Uncultured_soil
JN648841.1_Uncultured_soil
JN648843.1_Uncultured_soil
JN648846.1_Uncultured_soil
JN648847.1_Uncultured_soil
JN648849.1_Uncultured_soil
JN648850.1_Uncultured_soil
JN648854.1_Uncultured_soil
JN648856.1_Uncultured_soil
JN648858.1_Uncultured_soil
JN648859.1_Uncultured_soil
JN648863.1_Uncultured_soil
JN648865.1_Uncultured_soil
JN648870.1_Uncultured_soil

JN648871.1_Uncultured_soil
JN648872.1_Uncultured_soil
JN648876.1_Uncultured_soil
JN648877.1_Uncultured_soil
JN648878.1_Uncultured_soil
JN648881.1_Uncultured_soil
JN648883.1_Uncultured_soil
JN648885.1_Uncultured_soil
JN648886.1_Uncultured_soil
JN648894.1_Uncultured_soil
JN648895.1_Uncultured_soil
JN648897.1_Uncultured_soil
JN648898.1_Uncultured_soil
JN648899.1_Uncultured_soil
JN698217.1_Klebsiella_root
JN698220.1_Enterobacter_root
JN987872.1_Uncultured_marine root
JQ012736.1_Uncultured_freshwater
JQ023572.1_Uncultured_coral
JQ023573.1_Uncultured_coral
JQ023583.1_Uncultured_coral
JQ023584.1_Uncultured_coral
JQ023585.1_Uncultured_coral
JQ023586.1_Uncultured_coral
JQ358635.1_Uncultured_marine
JQ358637.1_Uncultured_marine
JQ358638.1_Uncultured_marine
JQ358639.1_Uncultured_marine
JQ358640.1_Uncultured_marine
JQ358642.1_Uncultured_marine
JQ358644.1_Uncultured_marine
JQ358645.1_Uncultured_marine
JQ358647.1_Uncultured_marine
JQ358648.1_Uncultured_marine
JQ358650.1_Uncultured_marine
JQ358652.1_Uncultured_marine
JQ358653.1_Uncultured_marine
JQ358704.1_Uncultured_marine
JQ358705.1_Uncultured_marine
JQ358706.1_Uncultured_marine
JQ358707.1_Uncultured_marine
JQ412989.1_Uncultured_marine sediment
JQ912223.1_Uncultured_sponge
JQ912224.1_Uncultured_sponge

JQ912226.1_Uncultured_marine
JQ912227.1_Uncultured_marine
JQ912233.1_Uncultured_marine
JQ912238.1_Uncultured_marine
JX042297.1_Uncultured_rice
JX064466.1_Uncultured_marine
JX064468.1_Uncultured_marine
JX064472.1_Uncultured_marine
JX064473.1_Uncultured_marine
JX064474.1_Uncultured_marine
JX064475.1_Uncultured_marine
JX064476.1_Uncultured_marine
JX064477.1_Uncultured_marine
JX064478.1_Uncultured_marine
JX064479.1_Uncultured_marine
JX079563.1_Uncultured_soil
JX079602.1_Uncultured_soil
JX079609.1_Uncultured_soil
JX134258.1_Uncultured_cyanobacterial mat
JX134259.1_Uncultured_cyanobacterial mat
JX154687.1_Uncultured_soil
JX154701.1_Uncultured_soil
JX154728.1_Uncultured_soil
JX154731.1_Uncultured_soil
JX154737.1_Uncultured_soil
JX154738.1_Uncultured_soil
JX154765.1_Uncultured_soil
JX154768.1_Uncultured_soil
JX154775.1_Uncultured_soil
JX154784.1_Uncultured_soil
JX154793.1_Uncultured_soil
JX154811.1_Uncultured_soil
JX154815.1_Uncultured_soil
JX154830.1_Uncultured_soil
JX154833.1_Uncultured_soil
JX154834.1_Uncultured_soil
JX154838.1_Uncultured_soil
JX154844.1_Uncultured_soil
JX154849.1_Uncultured_soil
JX154851.1_Uncultured_soil
JX154852.1_Uncultured_soil
JX268263.1_Uncultured_soil
JX268267.1_Uncultured_soil
JX268277.1_Uncultured_soil

JX268295.1_Uncultured_soil
JX268304.1_Uncultured_soil
JX268305.1_Uncultured_soil
JX268390.1_Uncultured_soil
JX268401.1_Uncultured_soil
JX268422.1_Uncultured_soil
JX268424.1_Uncultured_soil
JX268425.1_Uncultured_soil
JX268430.1_Uncultured_soil
JX268431.1_Uncultured_soil
JX268433.1_Uncultured_soil
JX268435.1_Uncultured_soil
JX268436.1_Uncultured_soil
JX268437.1_Uncultured_soil
JX268442.1_Uncultured_soil
JX268444.1_Uncultured_soil
JX268455.1_Uncultured_soil
JX268456.1_Uncultured_soil
JX268483.1_Uncultured_soil
JX268487.1_Uncultured_soil
JX268503.1_Uncultured_soil
JX268504.1_Uncultured_soil
JX268508.1_Uncultured_soil
JX866603.1_Uncultured_soil
JX878659.1_Uncultured_soil
JX878660.1_Uncultured_soil
JX878661.1_Uncultured_soil
JX878662.1_Uncultured_soil
JX878663.1_Uncultured_soil
JX878664.1_Uncultured_soil
JX878665.1_Uncultured_soil
JX878666.1_Uncultured_soil
JX878667.1_Uncultured_soil
JX878669.1_Uncultured_soil
KC013041.1_Uncultured_marine
KC013042.1_Uncultured_marine
KC013044.1_Uncultured_marine
KC013045.1_Uncultured_marine
KC013046.1_Uncultured_marine
KC013047.1_Uncultured_marine
KC013048.1_Uncultured_marine
KC013049.1_Uncultured_marine
KC013050.1_Uncultured_marine
KC013051.1_Uncultured_marine

KC013052.1_Uncultured_marine
KC013053.1_Uncultured_marine
KC013054.1_Uncultured_marine
KC013055.1_Uncultured_marine
KC013056.1_Uncultured_marine
KC013057.1_Uncultured_marine
KC013058.1_Uncultured_marine
KC013059.1_Uncultured_marine
KC013060.1_Uncultured_marine
KC013061.1_Uncultured_marine
KC013062.1_Uncultured_marine
KC013063.1_Uncultured_marine
KC013064.1_Uncultured_marine
KC013065.1_Uncultured_marine
KC013066.1_Uncultured_marine
KC013067.1_Uncultured_marine
KC013068.1_Uncultured_marine
KC013069.1_Uncultured_marine
KC013070.1_Uncultured_marine
KC013071.1_Uncultured_marine
KC013072.1_Uncultured_marine
KC013073.1_Uncultured_marine
KC013074.1_Uncultured_marine
KC013075.1_Uncultured_marine
KC013076.1_Uncultured_marine
KC013077.1_Uncultured_marine
KC013078.1_Uncultured_marine
KC013079.1_Uncultured_marine
KC013080.1_Uncultured_marine
KC013081.1_Uncultured_marine
KC013082.1_Uncultured_marine
KC013083.1_Uncultured_marine
KC013084.1_Uncultured_marine
KC013085.1_Uncultured_marine
KC013086.1_Uncultured_marine
KC013087.1_Uncultured_marine
KC013088.1_Uncultured_marine
KC013089.1_Uncultured_marine
KC013090.1_Uncultured_marine
KC013091.1_Uncultured_marine
KC013092.1_Uncultured_marine
KC013093.1_Uncultured_marine
KC013094.1_Uncultured_marine
KC013095.1_Uncultured_marine

KC013096.1_Uncultured_marine
KC013097.1_Uncultured_marine
KC013098.1_Uncultured_marine
KC013099.1_Uncultured_marine
KC013100.1_Uncultured_marine
KC013101.1_Uncultured_marine
KC013102.1_Uncultured_marine
KC013103.1_Uncultured_marine
KC013104.1_Uncultured_marine
KC013105.1_Uncultured_marine
KC013106.1_Uncultured_marine
KC013107.1_Uncultured_marine
KC013108.1_Uncultured_marine
KC013109.1_Uncultured_marine
KC013110.1_Uncultured_marine
KC013111.1_Uncultured_marine
KC013112.1_Uncultured_marine
KC013113.1_Uncultured_marine
KC013114.1_Uncultured_marine
KC013115.1_Uncultured_marine
KC013116.1_Uncultured_marine
KC013117.1_Uncultured_marine
KC013118.1_Uncultured_marine
KC013119.1_Uncultured_marine
KC013123.1_Uncultured_marine
KC013124.1_Uncultured_marine
KC013125.1_Uncultured_marine
KC013126.1_Uncultured_marine
KC013127.1_Uncultured_marine
KC013129.1_Uncultured_marine
KC013130.1_Uncultured_marine
KC013131.1_Uncultured_marine
KC013132.1_Uncultured_marine
KC013133.1_Uncultured_marine
KC013134.1_Uncultured_marine
KC013135.1_Uncultured_marine
KC013136.1_Uncultured_marine
KC013137.1_Uncultured_marine
KC013138.1_Uncultured_marine
KC013139.1_Uncultured_marine
KC013140.1_Uncultured_marine
KC013141.1_Uncultured_marine
KC013143.1_Uncultured_marine
KC013144.1_Uncultured_marine

KC013145.1_Uncultured_marine
KC013146.1_Uncultured_marine
KC013147.1_Uncultured_marine
KC013148.1_Uncultured_marine
KC013149.1_Uncultured_marine
KC013151.1_Uncultured_marine
KC013152.1_Uncultured_marine
KC013153.1_Uncultured_marine
KC013154.1_Uncultured_marine
KC013155.1_Uncultured_marine
KC013156.1_Uncultured_marine
KC013157.1_Uncultured_marine
KC013158.1_Uncultured_marine
KC013159.1_Uncultured_marine
KC013160.1_Uncultured_marine
KC013161.1_Uncultured_marine
KC013163.1_Uncultured_marine
KC013164.1_Uncultured_marine
KC013165.1_Uncultured_marine
KC013166.1_Uncultured_marine
KC013167.1_Uncultured_marine
KC013168.1_Uncultured_marine
KC013171.1_Uncultured_marine
KC013172.1_Uncultured_marine
KC013173.1_Uncultured_marine
KC013174.1_Uncultured_marine
KC013175.1_Uncultured_marine
KC013176.1_Uncultured_marine
KC013177.1_Uncultured_marine
KC013201.1_Uncultured_marine
KC013220.1_Uncultured_marine
KC013223.1_Uncultured_marine
KC013224.1_Uncultured_marine
KC013225.1_Uncultured_marine
KC013226.1_Uncultured_marine
KC140352.1_Gamma_Baltic
KC140353.1_Gamma_Baltic
KC140354.1_Gamma_Baltic
KC140355.1_Gamma_Baltic
KC140357.1_Gamma_Baltic
KC140358.1_Gamma_Baltic
KC140359.1_Gamma_Baltic
KC140360.1_Gamma_Baltic
KC140362.1_Gamma_Baltic

KC140363.1_Gamma_Baltic
KC140366.1_Gamma_Baltic
KC140367.1_Gamma_Baltic
KC140391.1_Uncultured_Baltic
KC140392.1_Uncultured_Baltic
KC140393.1_Uncultured_Baltic
KC140394.1_Uncultured_Baltic
KC140395.1_Uncultured_Baltic
KC222033.1_Uncultured_Mediterranean
KC222034.1_Uncultured_Mediterranean
KC222035.1_Uncultured_Mediterranean
KC222036.1_Uncultured_Mediterranean
KC222037.1_Uncultured_Mediterranean
KC222041.1_Uncultured_Mediterranean
KC222042.1_Uncultured_Mediterranean
KC222043.1_Uncultured_Mediterranean
KC222044.1_Uncultured_Mediterranean
KC222045.1_Uncultured_Mediterranean
KC222046.1_Uncultured_Mediterranean
KC222047.1_Uncultured_Mediterranean
KC222048.1_Uncultured_Mediterranean
KC222049.1_Uncultured_Mediterranean
KC222050.1_Uncultured_Mediterranean
KC222051.1_Uncultured_Mediterranean
KC256769.1_Pseudanabaena_shore
KC256777.1_Pseudanabaena_shore
KC261265.1_Uncultured_peat
KC261266.1_Uncultured_peat
KC261276.1_Uncultured_peat
KC295666.1_Uncultured_hot spring
KC295671.1_Uncultured_hot spring
KC295686.1_Uncultured_hot spring
KC445680.1_Uncultured_soil
KC445682.1_Uncultured_soil
KC445684.1_Uncultured_soil
KC445695.1_Uncultured_soil
KC445699.1_Uncultured_soil
KC445703.1_Uncultured_soil
KC445714.1_Uncultured_soil
KC445719.1_Uncultured_soil
KC445727.1_Uncultured_soil
KC445729.1_Uncultured_soil
KC748139.1_Uncultured_coral
KC748143.1_Uncultured_coral

KC748154.1_Uncultured_coral
KC748160.1_Uncultured_coral
KC748166.1_Uncultured_coral
KC748181.1_Uncultured_coral
KC748186.1_Uncultured_coral
KC748189.1_Uncultured_coral
KC748190.1_Uncultured_coral
KC748192.1_Uncultured_coral
KC748193.1_Uncultured_coral
KC748201.1_Uncultured_coral
KC773962.1_Uncultured_soil
KC773976.1_Uncultured_soil
KC914246.1_Uncultured_stem
KC914255.1_Uncultured_stem
KC914263.1_Uncultured_stem
KC914270.1_Uncultured_root
KC914271.1_Uncultured_root
KC914274.1_Uncultured_root
KC914277.1_Uncultured_root
KC914278.1_Uncultured_root
KC989924.1_Enterobacter
KC992980.1_Okeania_marine
KC992983.1_Okeania_marine
KC992984.1_Okeania_marine
KC992985.1_Okeania_marine
KC992986.1_Okeania_marine
KC992987.1_Okeania_marine
KC992988.1_Okeania_marine
KC992989.1_Okeania_marine
KF016971.1_Uncultured_soil
KF025359.1_Uncultured_copepod
KF032126.1_Uncultured_soil
KF032133.1_Uncultured_soil
KF032212.1_Uncultured_soil
KF032230.1_Uncultured_soil
KF151373.1_Gamma_Baltic
KF151374.1_Gamma_Baltic
KF151375.1_Gamma_Baltic
KF151376.1_Gamma_Baltic
KF151377.1_Gamma_Baltic
KF151378.1_Gamma_Baltic
KF151379.1_Gamma_Baltic
KF151380.1_Gamma_Baltic
KF151381.1_Gamma_Baltic

KF151386.1_Gamma_Baltic
KF151388.1_Gamma_Baltic
KF151389.1_Gamma_Baltic Sea
KF151391.1_Gamma_Baltic Sea
KF151400.1_Gamma_Baltic Sea
KF151411.1_Gamma_Baltic Sea
KF151449.1_Uncultured_marine
KF151463.1_Uncultured_marine
KF151478.1_Uncultured_marine
KF151479.1_Uncultured_marine
KF151480.1_Uncultured_marine
KF151481.1_Uncultured_marine
KF151482.1_Uncultured_marine
KF151483.1_Uncultured_marine
KF151484.1_Uncultured_marine
KF151485.1_Uncultured_marine
KF151487.1_Uncultured_marine
KF151489.1_Uncultured_marine
KF151490.1_Uncultured_marine
KF151491.1_Uncultured_marine
KF151492.1_Uncultured_marine
KF151493.1_Uncultured_marine
KF151494.1_Uncultured_marine
KF151495.1_Uncultured_marine
KF151496.1_Uncultured_marine
KF151497.1_Uncultured_marine
KF151498.1_Uncultured_marine
KF151500.1_Uncultured_marine
KF151501.1_Uncultured_marine
KF151503.1_Uncultured_marine
KF151504.1_Uncultured_marine
KF151509.1_Uncultured_marine
KF151510.1_Uncultured_marine
KF151511.1_Uncultured_marine
KF151512.1_Uncultured_marine
KF151513.1_Uncultured_marine
KF151528.1_Uncultured_marine
KF151529.1_Uncultured_marine
KF151531.1_Uncultured_marine
KF151533.1_Uncultured_marine
KF151537.1_Uncultured_marine
KF151539.1_Uncultured_marine
KF151540.1_Uncultured_marine
KF151544.1_Uncultured_marine

KF151545.1_Uncultured_marine
KF151546.1_Uncultured_marine
KF151548.1_Uncultured_marine
KF151551.1_Uncultured_marine
KF151552.1_Uncultured_marine
KF151556.1_Uncultured_marine
KF151557.1_Uncultured_marine
KF151558.1_Uncultured_marine
KF151562.1_Uncultured_marine
KF151564.1_Uncultured_marine
KF151565.1_Uncultured_marine
KF151566.1_Uncultured_marine
KF151567.1_GammaETSP1_marine
KF151570.1_Uncultured_marine
KF151571.1_Uncultured_marine
KF151572.1_Uncultured_marine
KF151573.1_Uncultured_marine
KF151575.1_Uncultured_marine
KF151576.1_Uncultured_marine
KF151577.1_Uncultured_marine
KF151579.1_Uncultured_marine
KF151588.1_Uncultured_marine
KF151592.1_Uncultured_marine
KF151599.1_Uncultured_marine
KF151600.1_Uncultured_marine
KF151604.1_Uncultured_marine
KF151605.1_Uncultured_marine
KF151606.1_Uncultured_marine
KF151612.1_Uncultured_marine
KF151613.1_Uncultured_marine
KF151619.1_Uncultured_marine
KF151621.1_Uncultured_marine
KF151622.1_Uncultured_marine
KF151625.1_Uncultured_marine
KF151628.1_Uncultured_marine
KF151629.1_Uncultured_marine
KF151632.1_Uncultured_marine
KF151633.1_Uncultured_marine
KF151634.1_Uncultured_marine
KF151635.1_Uncultured_marine
KF151636.1_Uncultured_marine
KF151643.1_Uncultured_marine
KF151645.1_Uncultured_marine
KF151647.1_Uncultured_marine

KF151648.1_Uncultured_marine
KF151649.1_Uncultured_marine
KF151651.1_Uncultured_marine
KF151654.1_Uncultured_marine
KF151655.1_Uncultured_marine
KF151656.1_Uncultured_marine
KF151658.1_Uncultured_marine
KF151660.1_Uncultured_marine
KF151661.1_GammaETSP3_marine
KF151662.1_Uncultured_marine
KF151663.1_Uncultured_marine
KF151665.1_Uncultured_marine
KF151670.1_Uncultured_marine
KF151672.1_Uncultured_marine
KF151673.1_Uncultured_marine
KF151674.1_Uncultured_marine
KF151675.1_Uncultured_marine
KF151676.1_Uncultured_marine
KF151677.1_Uncultured_marine
KF151678.1_Uncultured_marine
KF151680.1_Uncultured_marine
KF151683.1_Uncultured_marine
KF151686.1_Uncultured_marine
KF151687.1_Uncultured_marine
KF151689.1_Uncultured_marine
KF151692.1_Uncultured_marine
KF151704.1_Uncultured_marine
KF151705.1_Uncultured_marine
KF151709.1_Uncultured_marine
KF151711.1_Uncultured_marine
KF151713.1_Uncultured_marine
KF151716.1_Uncultured_marine
KF151718.1_Uncultured_marine
KF151719.1_Uncultured_marine
KF151720.1_Uncultured_marine
KF151724.1_Uncultured_marine
KF151725.1_Uncultured_marine
KF151726.1_Uncultured_marine
KF151727.1_Uncultured_marine
KF151730.1_Uncultured_marine
KF151731.1_Uncultured_marine
KF151732.1_Uncultured_marine
KF151734.1_Uncultured_marine
KF151736.1_Uncultured_marine

KF151740.1_Uncultured_marine
KF151741.1_Uncultured_marine
KF151748.1_Uncultured_marine
KF151749.1_Uncultured_marine
KF151751.1_Uncultured_marine
KF151756.1_Uncultured_marine
KF151762.1_Uncultured_marine
KF151766.1_Uncultured_marine
KF151767.1_Uncultured_marine
KF151768.1_Uncultured_marine
KF151769.1_Uncultured_marine
KF151771.1_Uncultured_marine
KF151775.1_Uncultured_marine
KF151776.1_Uncultured_marine
KF151779.1_Uncultured_marine
KF151784.1_Uncultured_marine
KF151785.1_Uncultured_marine
KF151787.1_Uncultured_marine
KF151790.1_Uncultured_marine
KF151791.1_Uncultured_marine
KF151792.1_Uncultured_marine
KF151799.1_Uncultured_marine
KF151800.1_Uncultured_marine
KF151805.1_Uncultured_marine
KF151807.1_Uncultured_marine
KF151808.1_Uncultured_marine
KF151809.1_Uncultured_marine
KF151811.1_Uncultured_marine
KF151812.1_Uncultured_marine
KF151813.1_Uncultured_marine
KF151814.1_Uncultured_marine
KF151815.1_Uncultured_marine
KF151816.1_Uncultured_marine
KF151817.1_Uncultured_marine
KF151819.1_ETSP2_marine
KF151821.1_Uncultured_marine
KF151822.1_Uncultured_marine
KF151823.1_Uncultured_marine
KF151824.1_Uncultured_marine
KF151825.1_Uncultured_marine
KF151826.1_Uncultured_marine
KF151828.1_Uncultured_marine
KF151831.1_Uncultured_marine
KF151834.1_Uncultured_marine

KF151836.1_Uncultured_marine
KF151837.1_Uncultured_marine
KF151838.1_Uncultured_marine
KF151840.1_Uncultured_marine
KF151843.1_Uncultured_marine
KF285291.1_Uncultured_marine sediment
KF285295.1_Uncultured_marine sediment
KF285304.1_Uncultured_marine sediment
KF285311.1_Uncultured_marine sediment
KF285313.1_Uncultured_marine sediment
KF285314.1_Uncultured_marine sediment
KF285317.1_Uncultured_marine sediment
KF285319.1_Uncultured_marine sediment
KF285332.1_Uncultured_marine sediment
KF285333.1_Uncultured_marine sediment
KF285336.1_Uncultured_marine sediment
KF285337.1_Uncultured_marine sediment
KF285340.1_Uncultured_marine sediment
KF285342.1_Uncultured_marine sediment
KF285348.1_Uncultured_marine sediment
KF285351.1_Uncultured_marine sediment
KF285360.1_Uncultured_marine sediment
KF285364.1_Uncultured_marine sediment
KF285365.1_Uncultured_marine sediment
KF285370.1_Uncultured_marine sediment
KF285381.1_Uncultured_marine sediment
KF285382.1_Uncultured_marine sediment
KF285383.1_Uncultured_marine sediment
KF285386.1_Uncultured_marine sediment
KF285389.1_Uncultured_marine sediment
KF285397.1_Uncultured_marine sediment
KF307333.1_Uncultured_marine
KF307337.1_Uncultured_marine
KF307347.1_Uncultured_marine
KF444521.1_Uncultured_coral
KF444529.1_Uncultured_coral
KF444536.1_Uncultured_coral
KF444538.1_Uncultured_coral
KF515759.1_Uncultured_marine
KF515763.1_Uncultured_marine
KF515774.1_Uncultured_marine
KF515777.1_Uncultured_marine
KF515778.1_Uncultured_marine
KF515786.1_Uncultured_marine

KF515788.1_Uncultured_marine
KF515789.1_Uncultured_marine
KF515793.1_Uncultured_marine
KF515794.1_Uncultured_marine
KF515795.1_Uncultured_marine
KF515796.1_Uncultured_marine
KF515815.1_Uncultured_marine
KF515817.1_Uncultured_marine
KF515821.1_Uncultured_marine
KF515823.1_Uncultured_marine
KF515826.1_Uncultured_marine
KF515843.1_Uncultured_marine
KF541074.1_Uncultured_terrestrial grass
KF541075.1_Uncultured_terrestrial grass
KF541076.1_Uncultured_terrestrial grass
KF541077.1_Uncultured_terrestrial grass
KF546325.1_Uncultured_marine
KF546326.1_Uncultured_marine
KF546327.1_Uncultured_marine
KF546329.1_Uncultured_marine
KF546330.1_Uncultured_marine
KF546333.1_Uncultured_marine
KF546334.1_Uncultured_marine
KF546335.1_Uncultured_marine
KF546336.1_Uncultured_marine
KF546337.1_Uncultured_marine
KF546339.1_Uncultured_marine
KF546343.1_Uncultured_marine
KF546344.1_Uncultured_marine
KF546345.1_Uncultured_marine
KF546346.1_Uncultured_marine
KF546349.1_Uncultured_marine
KF546350.1_Uncultured_marine
KF546351.1_Uncultured_marine
KF546353.1_Uncultured_marine
KF546354.1_Uncultured_marine
KF546355.1_Uncultured_marine
KF546356.1_Uncultured_marine
KF546357.1_Uncultured_marine
KF546358.1_Uncultured_marine
KF546360.1_Uncultured_marine
KF546361.1_Uncultured_marine
KF546362.1_Uncultured_marine
KF546364.1_Uncultured_marine

KF546367.1_Uncultured_marine
KF546368.1_Uncultured_marine
KF546369.1_Uncultured_marine
KF546372.1_Uncultured_marine
KF546374.1_Uncultured_marine
KF546376.1_Uncultured_marine
KF546377.1_Uncultured_marine
KF546378.1_Uncultured_marine
KF546379.1_Uncultured_marine
KF546380.1_Uncultured_marine
KF546381.1_Uncultured_marine
KF546382.1_Uncultured_marine
KF546383.1_Uncultured_marine
KF546384.1_Uncultured_marine
KF546385.1_Uncultured_marine
KF546386.1_Uncultured_marine
KF546388.1_Uncultured_marine
KF546390.1_Uncultured_marine
KF546393.1_Uncultured_marine
KF546394.1_Uncultured_marine
KF546395.1_Uncultured_marine
KF546397.1_Uncultured_marine
KF546398.1_Uncultured_marine
KF546399.1_Uncultured_marine
KF546402.1_Uncultured_marine
KF546408.1_Uncultured_marine
KF546409.1_Uncultured_marine
KF546412.1_Uncultured_marine
KF546413.1_Uncultured_marine
KF546415.1_Uncultured_marine
KF546417.1_Uncultured_marine
KF546419.1_Uncultured_marine
KF546421.1_Uncultured_marine
KF546423.1_Uncultured_marine
KF546424.1_Uncultured_marine
KF546427.1_Uncultured_marine
KF546429.1_Uncultured_marine
KF546433.1_Uncultured_marine
KF546434.1_Uncultured_marine
KF546438.1_Uncultured_marine
KF546441.1_Uncultured_marine
KF546443.1_Uncultured_marine
KF546444.1_Uncultured_marine
KF546445.1_Uncultured_marine

KF546446.1_Uncultured_marine
KF546447.1_Uncultured_marine
KF546452.1_Uncultured_marine
KF546454.1_Uncultured_marine
KF546455.1_Uncultured_marine
KF546456.1_Uncultured_marine
KF546457.1_Uncultured_marine
KF546458.1_Uncultured_marine
KF546459.1_Uncultured_marine
KF546460.1_Uncultured_marine
KF546461.1_Uncultured_marine
KF546462.1_Uncultured_marine
KF546463.1_Uncultured_marine
KF546464.1_Uncultured_marine
KF546465.1_Uncultured_marine
KF546466.1_Uncultured_marine
KF546467.1_Uncultured_marine
KF546468.1_Uncultured_marine
KF546469.1_Uncultured_marine
KF546470.1_Uncultured_marine
KF546471.1_Uncultured_marine
KF619536.1_Uncultured_marine
KF619537.1_Uncultured_marine
KF740734.1_Uncultured_marine
KF740745.1_Uncultured_marine
KF740746.1_Uncultured_marine
KF740778.1_Uncultured_marine
KF800051.1_Thiorhodospira
KF800054.1_Rhodoblastus
KF800062.1_Thiocapsa
KF800064.1_Uncultured_saline lake sediment
KF800068.1_Uncultured_saline lake sediment
KF800070.1_Uncultured_saline lake sediment
KF800072.1_Uncultured_saline lake sediment
KF800074.1_Uncultured_saline lake
KF800080.1_Uncultured_saline lake sediment
KF800085.1_Uncultured_saline lake sediment
KF800088.1_Uncultured_saline lake sediment
KF813025.1_Uncultured_Yellow Sea
KF813026.1_Uncultured_Yellow Sea
KF813028.1_Uncultured_Yellow Sea
KF813031.1_Uncultured_Yellow Sea
KF813033.1_Uncultured_Yellow Sea
KF816262.1_Uncultured_freshwater biofilm

KF816272.1_Uncultured_freshwater biofilm
KF816286.1_Uncultured_freshwater biofilm
KF846584.1_Uncultured_soil
KF846586.1_Uncultured_soil
KF846595.1_Uncultured_soil
KF846597.1_Uncultured_soil
KF846599.1_Uncultured_soil
KF846600.1_Uncultured_soil
KF846607.1_Uncultured_soil
KF846608.1_Uncultured_soil
KF846610.1_Uncultured_soil
KF846611.1_Uncultured_soil
KF846619.1_Uncultured_soil
KF846622.1_Uncultured_soil
KF846624.1_Uncultured_soil
KF846626.1_Uncultured_soil
KF846635.1_Uncultured_soil
KF846638.1_Uncultured_soil
KF846641.1_Uncultured_soil
KF846642.1_Uncultured_soil
KF846644.1_Uncultured_soil
KF846650.1_Uncultured_soil
KF846659.1_Uncultured_soil
KF846660.1_Uncultured_soil
KF846666.1_Uncultured_soil
KF846670.1_Uncultured_soil
KF846671.1_Uncultured_soil
KF846672.1_Uncultured_soil
KF846673.1_Uncultured_soil
KF846676.1_Uncultured_soil
KF846677.1_Uncultured_soil
KF846678.1_Uncultured_soil
KF846684.1_Uncultured_soil
KF846686.1_Uncultured_soil
KF846687.1_Uncultured_soil
KF846689.1_Uncultured_soil
KF846690.1_Uncultured_soil
KF846691.1_Uncultured_soil
KF846697.1_Uncultured_soil
KF846699.1_Uncultured_soil
KF846702.1_Uncultured_soil
KF846705.1_Uncultured_soil
KF846706.1_Uncultured_soil
KF846708.1_Uncultured_soil

KF846716.1_Uncultured_soil
KF846717.1_Uncultured_soil
KF846718.1_Uncultured_soil
KF846722.1_Uncultured_soil
KF846725.1_Uncultured_soil
KF846727.1_Uncultured_soil
KF846731.1_Uncultured_soil
KF846732.1_Uncultured_soil
KF846735.1_Uncultured_soil
KF846736.1_Uncultured_soil
KF846738.1_Uncultured_soil
KF846739.1_Uncultured_soil
KF846741.1_Uncultured_soil
KF846743.1_Uncultured_soil
KF846744.1_Uncultured_soil
KF846746.1_Uncultured_soil
KF846750.1_Uncultured_soil
KF846755.1_Uncultured_soil
KF846761.1_Uncultured_soil
KF846762.1_Uncultured_soil
KF846766.1_Uncultured_soil
KF846768.1_Uncultured_soil
KF846770.1_Uncultured_soil
KF846771.1_Uncultured_soil
KF846773.1_Uncultured_soil
KF846777.1_Uncultured_soil
KF846782.1_Uncultured_soil
KF846785.1_Uncultured_soil
KF846788.1_Uncultured_soil
KF846792.1_Uncultured_soil
KF846799.1_Uncultured_soil
KF846806.1_Uncultured_soil
KF846811.1_Uncultured_soil
KF846812.1_Uncultured_soil
KF846813.1_Uncultured_soil
KF846816.1_Uncultured_soil
KF846821.1_Uncultured_soil
KF846822.1_Uncultured_soil
KF846824.1_Uncultured_soil
KF846825.1_Uncultured_soil
KF846833.1_Uncultured_soil
KF846838.1_Uncultured_soil
KF846842.1_Uncultured_soil
KF846844.1_Uncultured_soil

KF846846.1_Uncultured_soil
KF846850.1_Uncultured_soil
KF846856.1_Uncultured_soil
KF846859.1_Uncultured_soil
KF846860.1_Uncultured_soil
KF846863.1_Uncultured_soil
KF846866.1_Uncultured_soil
KF846868.1_Uncultured_soil
KF846869.1_Uncultured_soil
KF846880.1_Uncultured_soil
KF846881.1_Uncultured_soil
KF846882.1_Uncultured_soil
KF846884.1_Uncultured_soil
KF846887.1_Uncultured_soil
KF846889.1_Uncultured_soil
KF846890.1_Uncultured_soil
KF846894.1_Uncultured_soil
KF846897.1_Uncultured_soil
KF846899.1_Uncultured_soil
KF846903.1_Uncultured_soil
KF846921.1_Uncultured_soil
KF846927.1_Uncultured_soil
KF846941.1_Uncultured_soil
KF846943.1_Uncultured_soil
KF846949.1_Uncultured_soil
KF846952.1_Uncultured_soil
KF846955.1_Uncultured_soil
KF846960.1_Uncultured_soil
KF846965.1_Uncultured_soil
KF846973.1_Uncultured_soil
KF846978.1_Uncultured_soil
KF846981.1_Uncultured_soil
KF846985.1_Uncultured_soil
KF846994.1_Uncultured_soil
KF847019.1_Uncultured_soil
KF847028.1_Uncultured_soil
KF847040.1_Uncultured_soil
KF847046.1_Uncultured_soil
KF847048.1_Uncultured_soil
KF847059.1_Uncultured_soil
KF847060.1_Uncultured_soil
KF847065.1_Uncultured_soil
KF847071.1_Uncultured_soil
KF847074.1_Uncultured_soil

KF847080.1_Uncultured_soil
KF847081.1_Uncultured_soil
KF847086.1_Uncultured_soil
KF847089.1_Uncultured_soil
KF847091.1_Uncultured_soil
KF847094.1_Uncultured_soil
KF847095.1_Uncultured_soil
KF847105.1_Uncultured_soil
KF847106.1_Uncultured_soil
KF847111.1_Uncultured_soil
KF847112.1_Uncultured_soil
KF847115.1_Uncultured_soil
KF847116.1_Uncultured_soil
KF847117.1_Uncultured_soil
KF847138.1_Uncultured_soil
KF847140.1_Uncultured_soil
KF847142.1_Uncultured_soil
KF847145.1_Uncultured_soil
KF847147.1_Uncultured_soil
KF847151.1_Uncultured_soil
KF847152.1_Uncultured_soil
KF847153.1_Uncultured_soil
KF847160.1_Uncultured_soil
KF847163.1_Uncultured_soil
KF847166.1_Uncultured_soil
KF847171.1_Uncultured_soil
KF847187.1_Uncultured_soil
KF847188.1_Uncultured_soil
KF847204.1_Uncultured_soil
KF847206.1_Uncultured_soil
KF847208.1_Uncultured_soil
KF847210.1_Uncultured_soil
KF847216.1_Uncultured_soil
KF847221.1_Uncultured_soil
KF847222.1_Uncultured_soil
KF847225.1_Uncultured_soil
KF847227.1_Uncultured_soil
KF847235.1_Uncultured_soil
KF847236.1_Uncultured_soil
KF847238.1_Uncultured_soil
KF847239.1_Uncultured_soil
KF847241.1_Uncultured_soil
KF847243.1_Uncultured_soil
KF847244.1_Uncultured_soil

KF847251.1_Uncultured_soil
KF847297.1_Uncultured_soil
KF847299.1_Uncultured_soil
KF847304.1_Uncultured_soil
KF847316.1_Uncultured_soil
KF847333.1_Uncultured_soil
KF847408.1_Uncultured_soil
KF847423.1_Uncultured_soil
KF847447.1_Uncultured_soil
KF847452.1_Uncultured_soil
KF847508.1_Uncultured_soil
KF847515.1_Uncultured_soil
KF847531.1_Uncultured_soil
KF847550.1_Uncultured_soil
KF847551.1_Uncultured_soil
KF847558.1_Uncultured_soil
KF847564.1_Uncultured_soil
KF847619.1_Uncultured_soil
KF847641.1_Uncultured_soil
KF847681.1_Uncultured_soil
KF847798.1_Uncultured_soil
KF847812.1_Uncultured_soil
KF847816.1_Uncultured_soil
KF847818.1_Uncultured_soil
KF847824.1_Uncultured_soil
KF847834.1_Uncultured_soil
KF847858.1_Uncultured_soil
KF847861.1_Uncultured_soil
KF847864.1_Uncultured_soil
KF847876.1_Uncultured_soil
KF847878.1_Uncultured_soil
KF847879.1_Uncultured_soil
KF847888.1_Uncultured_soil
KF847897.1_Uncultured_soil
KF847905.1_Uncultured_soil
KF847908.1_Uncultured_soil
KF847912.1_Uncultured_soil
KF847932.1_Uncultured_soil
KF847954.1_Uncultured_soil
KF847965.1_Uncultured_soil
KF847997.1_Uncultured_soil
KF848025.1_Uncultured_soil
KF848027.1_Uncultured_soil
KF848041.1_Uncultured_soil

KF848042.1_Uncultured_soil
KF848059.1_Uncultured_soil
KF848070.1_Uncultured_soil
KF848074.1_Uncultured_soil
KF848083.1_Uncultured_soil
KF848086.1_Uncultured_soil
KF848104.1_Uncultured_soil
KF848107.1_Uncultured_soil
KF848110.1_Uncultured_soil
KF848117.1_Uncultured_soil
KF848118.1_Uncultured_soil
KF848122.1_Uncultured_soil
KF848125.1_Uncultured_soil
KF848132.1_Uncultured_soil
KF848141.1_Uncultured_soil
KF848161.1_Uncultured_soil
KF848170.1_Uncultured_soil
KF854483.1_Uncultured_marine
KF854503.1_Uncultured_marine
KF854505.1_Uncultured_marine
KF854516.1_Uncultured_marine
KF854518.1_Uncultured_marine
KF854520.1_Uncultured_marine
KF854521.1_Uncultured_marine
KF854523.1_Uncultured_marine
KF854524.1_Uncultured_marine
KF854528.1_Uncultured_marine
KF854533.1_Uncultured_marine
KF854547.1_Uncultured_marine
KF854548.1_Uncultured_marine
KF854553.1_Uncultured_marine
KF854558.1_Uncultured_marine
KF854560.1_Uncultured_marine
KF854561.1_Uncultured_marine
KF854562.1_Uncultured_marine
KF854571.1_Uncultured_marine
KF854575.1_Uncultured_marine
KF854576.1_Uncultured_marine
KF861045.1_Uncultured_soil
KF861046.1_Uncultured_soil
KF861049.1_Uncultured_soil
KF861050.1_Uncultured_soil
KF861051.1_Uncultured_soil
KF861054.1_Uncultured_soil

KF861055.1_Uncultured_soil
KF861056.1_Uncultured_soil
KF861060.1_Uncultured_soil
KF861061.1_Uncultured_soil
KF861062.1_Uncultured_soil
KF861063.1_Uncultured_soil
KF861064.1_Uncultured_soil
KF861066.1_Uncultured_soil
KF861067.1_Uncultured_soil
KF861071.1_Uncultured_soil
KF861077.1_Uncultured_soil
KF861078.1_Uncultured_soil
KF861081.1_Uncultured_soil
KF861083.1_Uncultured_soil
KF861085.1_Uncultured_soil
KF861086.1_Uncultured_soil
KF861088.1_Uncultured_soil
KF861090.1_Uncultured_soil
KF861095.1_Uncultured_soil
KF861097.1_Uncultured_soil
KF861104.1_Uncultured_soil
KF861106.1_Uncultured_soil
KF861108.1_Uncultured_soil
KF861114.1_Uncultured_soil
KF861115.1_Uncultured_soil
KF861117.1_Uncultured_soil
KF861121.1_Uncultured_soil
KF861125.1_Uncultured_soil
KF861126.1_Uncultured_soil
KF861127.1_Uncultured_soil
KF861131.1_Uncultured_soil
KF861133.1_Uncultured_soil
KF861134.1_Uncultured_soil
KF861136.1_Uncultured_soil
KF861137.1_Uncultured_soil
KF861139.1_Uncultured_soil
KF861143.1_Uncultured_soil
KF861146.1_Uncultured_soil
KF861147.1_Uncultured_soil
KF861148.1_Uncultured_soil
KF861150.1_Uncultured_soil
KF861152.1_Uncultured_soil
KF861153.1_Uncultured_soil
KF861158.1_Uncultured_soil

KF861159.1_Uncultured_soil
KF861160.1_Uncultured_soil
KF861161.1_Uncultured_soil
KF861166.1_Uncultured_soil
KF861170.1_Uncultured_soil
KF861175.1_Uncultured_soil
KF861180.1_Uncultured_soil
KF861181.1_Uncultured_soil
KF861187.1_Uncultured_soil
KF861188.1_Uncultured_soil
KF861190.1_Uncultured_soil
KF861194.1_Uncultured_soil
KF861195.1_Uncultured_soil
KF861196.1_Uncultured_soil
KF861197.1_Uncultured_soil
KF861198.1_Uncultured_soil
KF861200.1_Uncultured_soil
KF861204.1_Uncultured_soil
KF861212.1_Uncultured_soil
KF861219.1_Uncultured_soil
KF861222.1_Uncultured_soil
KF861223.1_Uncultured_soil
KF861224.1_Uncultured_soil
KF861232.1_Uncultured_soil
KF861236.1_Uncultured_soil
KF861238.1_Uncultured_soil
KF861240.1_Uncultured_soil
KF861243.1_Uncultured_soil
KF861244.1_Uncultured_soil
KF861248.1_Uncultured_soil
KF861255.1_Uncultured_soil
KF861261.1_Uncultured_soil
KF861288.1_Uncultured_soil
KF861289.1_Uncultured_soil
KF861295.1_Uncultured_soil
KF861297.1_Uncultured_soil
KF861300.1_Uncultured_soil
KF861308.1_Uncultured_soil
KF861314.1_Uncultured_soil
KF861324.1_Uncultured_soil
KF861335.1_Uncultured_soil
KF861338.1_Uncultured_soil
KF861347.1_Uncultured_soil
KF861371.1_Uncultured_soil

KF861380.1_Uncultured_soil
KF861403.1_Uncultured_soil
KF861408.1_Uncultured_soil
KF861486.1_Uncultured_soil
KF872848.1_Uncultured_root
KF872849.1_Uncultured_root
KF872854.1_Uncultured_root
KF872855.1_Uncultured_root
KF872857.1_Uncultured_root
KF872860.1_Uncultured_root
KF872862.1_Uncultured_root
KF872874.1_Uncultured_root
KF872881.1_Uncultured_root
KF872883.1_Uncultured_root
KF872887.1_Uncultured_root
KF872888.1_Uncultured_root
KF872889.1_Uncultured_root
KF872891.1_Uncultured_root
KF872894.1_Uncultured_root
KF872898.1_Uncultured_root
KF872903.1_Uncultured_root
KF872908.1_Uncultured_root
KF872909.1_Uncultured_root
KF872910.1_Uncultured_root
KF872911.1_Uncultured_root
KF872912.1_Uncultured_root
KF872923.1_Uncultured_root
KF872927.1_Uncultured_root
KF872941.1_Uncultured_root
KF872953.1_Uncultured_root
KF872956.1_Uncultured_root
KF872969.1_Uncultured_root
KF872971.1_Uncultured_root
KF872973.1_Uncultured_root
KF872982.1_Uncultured_root
KF872984.1_Uncultured_root
KF872990.1_Uncultured_root
KF872993.1_Uncultured_root
KF881088.1_Azotobacter_soil
KF881089.1_Azotobacter_soil
KF881090.1_Azotobacter_soil
KF881091.1_Azotobacter_soil
KF881092.1_Azotobacter_soil
KF881093.1_Azotobacter_soil

KF881094.1_Azotobacter_soil
KF881095.1_Azotobacter_soil
KF881096.1_Azotobacter_soil
KF881097.1_Azotobacter_soil
KF881098.1_Azotobacter_soil
KF881099.1_Azotobacter_soil
KF881100.1_Azotobacter_soil
KF881101.1_Azotobacter_soil
KF881102.1_Azotobacter_soil
KF881103.1_Azotobacter_soil
KF881104.1_Azotobacter_soil
KF881105.1_Azotobacter_soil
KF881106.1_Azotobacter_soil
KF881107.1_Azotobacter_soil
KF881108.1_Azotobacter_soil
KF881109.1_Azotobacter_soil
KF881110.1_Azotobacter_soil
KF881111.1_Azotobacter_soil
KF881112.1_Azotobacter_soil
KF881113.1_Azotobacter_soil
KF881114.1_Azotobacter_soil
KF881115.1_Azotobacter_soil
KF881116.1_Azotobacter_soil
KF881117.1_Azotobacter_soil
KF881118.1_Azotobacter_soil
KF881119.1_Azotobacter_soil
KF881120.1_Azotobacter_soil
KF881121.1_Azotobacter_soil
KF901484.1_Uncultured_borehole
KF901488.1_Uncultured_borehole
KF960581.1_Uncultured_marine
KF960582.1_Uncultured_marine
KF960590.1_Uncultured_marine
KF960592.1_Uncultured_marine
KF960599.1_Uncultured_marine
KF960600.1_Uncultured_marine
KF960605.1_Uncultured_marine
KF960606.1_Uncultured_marine
KF960619.1_Uncultured_marine
KF960627.1_Uncultured_marine
KF960630.1_Uncultured_marine
KJ021871.1_Marinobacterium_root
KJ021873.1_Marinobacterium
KJ170905.1_Uncultured_freshwater

KJ170907.1_Uncultured_freshwater
KJ460973.1_Uncultured_soil
KJ460975.1_Uncultured_soil
KJ460978.1_Uncultured_soil
KJ460979.1_Uncultured_soil
KJ460980.1_Uncultured_soil
KJ460981.1_Uncultured_soil
KJ460985.1_Uncultured_soil
KJ997934.1_Uncultured_wood
M11579.1_Azotobacter
M20568.1_A.vinelandii
M32371.1_A.vinelandii
M63690.1_Unknown_marine
M63691.1_Klebsiella
M73020.1_A.chroococcum
U23650.1_Vibrio
U26186.1_Unidentified_marine snow
U43437.1_Unidentified_marine cyanobacterial mat
U43438.1_Marine_marine cyanobacterial mat
U43439.1_Unidentified_marine cyanobacterial mat
U43442.1_Marine_marine cyanobacterial mat
U43443.1_Marine_marine cyanobacterial mat
U43444.1_Marine_marine cyanobacterial mat
U43445.1_Marine_marine cyanobacterial mat
V00631.1_Klebsiella
X03916.1_Azotobacter
X13303.1_Klebsiella
X13519.1_A.vinelandii
X51756.1_Azotobacter
