# Supplementary material for: Widespread Distribution and Expression of Gamma A (UMB), an Uncultured, Diazotrophic, γ-Proteobacterial nifH Phylotype
Source: PLoS One. 2015 Jun 23;10(6):e0128912. doi: 10.1371/journal.pone.0128912 (PMC4477881; doi:10.1371/journal.pone.0128912)
Supplement: S2 Table — The sample size of each cluster is given (n). The calculated mean, and standard error (SE), and range of values measured for each parameter are given. Means in bold font are statistically different (ANOVA, P < 0.01). (PDF) [file pone.0128912.s009.pdf]

S2 Table. Descriptive statistics summary of PCA clusters. The sample size of each cluster is given (n). The calculated mean, and standard error (SE), and range of values measured for each parameter are given. Means in bold font are statistically different (ANOVA, $P < 0.01$)

	N	DNA (*nifH* copies l^{-1})		cDNA (*nifH* copies l^{-1})		Depth (m)		Sal (psu)		Temp (°C)		NO3 (μM)		PO4 (μM)		SiO2 (μM)		O2 (μM)	
		Mean (SE)	Range	Mean (SE)	Range	Mean (SE)	Range	Mean (SE)	Range	Mean (SE)	Range	Mean (SE)	Range	Mean (SE)	Range	Mean (SE)	Range	Mean (SE)	Range
Cluster 1	196	**3x10^{3} (5x102)**	0-6x10^{4}	**8x10^{3} (2x103)**	0-2x10^{5}	26 (1.6)	1-100	**31 (0.4)**	22-37	**30 (0.4)**	18-36	**0.4 (0.07)**	0-6.7	**0.2 (0.01)**	0-1	**0.6 (0.04)**	0-3.1	**194 (1.6)**	92-29
Cluster 2	27	6x10^{2} (5x102)	0-1x10^{4}	30 (10)	3x10^{2}	66 (5.3)	40-150	**18 (0.4)**	15-23	**36 (0.06)**	36-37	17.5 (1.5)	0.4-28	1.1 (1.3)	0.02-1.7	**2.2 (0.4)**	0.4-7.5	100 (9.7)	31-213
Cluster 3	121	1x10^{2} (33)	0-2x10^{3}	**1x10^{2} (40)**	3x10^{3}	**177 (14)**	3-500	**36 (0.04)**	35-37	**16 (0.4)**	7-29	18 (0.95)	0.1-37	1.2 (0.06)	0.01-2.3	**7.1 (0.5)**	0.2-22	109 (4.5)	33-204
