# Supplementary material for: Widespread Distribution and Expression of Gamma A (UMB), an Uncultured, Diazotrophic, γ-Proteobacterial nifH Phylotype
Source: PLoS One. 2015 Jun 23;10(6):e0128912. doi: 10.1371/journal.pone.0128912 (PMC4477881; doi:10.1371/journal.pone.0128912)
Supplement: S3 Table — The number of samples where Gamma A abundances (nifH copies l-1) were detected per each depth range (m) are shown. The number of samples per depth range (n) is given and the detection frequency (%) was calculated. (PDF) [file pone.0128912.s010.pdf]

S3 Table. Detection Frequency of Gamma A in qPCR Analysis. The number samples where Gamma A abundances (*nifH* copies l^{-1}) were detected per each depth range (m) are shown. The number of samples per depth range (n) is given and the detection frequency (%) was calculated.

	<70 m		70-150 m		>150 m	
Abundance	DNA	cDNA	DNA	cDNA	DNA	cDNA
10^5	0	8	0	0	0	0
10^4	75	137	2	1	0	0
10^3	283	121	7	4	0	1
10^2	148	79	31	14	11	3
10^1	73	39	38	14	13	9
DNQ	3	0	1	0	0	0
n	755	527	153	93	60	50
Detected (%)	77	73	52	35	40	26
