# Supplementary material for: Widespread Distribution and Expression of Gamma A (UMB), an Uncultured, Diazotrophic, γ-Proteobacterial nifH Phylotype
Source: PLoS One. 2015 Jun 23;10(6):e0128912. doi: 10.1371/journal.pone.0128912 (PMC4477881; doi:10.1371/journal.pone.0128912)
Supplement: S4 Table — The number of DNA and cDNA samples collected in each latitudinal band (°N) is provided. (PDF) [file pone.0128912.s011.pdf]

S4 Table. Sampling frequency arranged by latitudinal bands. The number of DNA and cDNA samples collected in each latitudinal band (°N) is provided.

Lat (°N)	N	
	DNA	cDNA
>41	12	3
31-40	45	3
21-30	91	35
11-20	365	294
1-10	179	117
0	50	38
-1- -10	100	86
-11- -20	14	3
-21- -30	51	43
-31- -40	64	44
>-41	18	6
