# Supplementary material for: Widespread Distribution and Expression of Gamma A (UMB), an Uncultured, Diazotrophic, γ-Proteobacterial nifH Phylotype
Source: PLoS One. 2015 Jun 23;10(6):e0128912. doi: 10.1371/journal.pone.0128912 (PMC4477881; doi:10.1371/journal.pone.0128912)
Supplement: S5 Table — Representative sequences in Fig 3 are in bold font. (PDF) [file pone.0128912.s012.pdf]

S5 Table. Designation of sequences in the Marine 1 clade into OTUs at 100% similarity. Representative sequences in Figure 3 are in bold font.

Representative Sequence	Identical
AF059623.1_Zehr	KF740745.1_Uncultured
	AB679088.1_Uncultured
	AB679069.1_Uncultured
	AB679052.1_Uncultured
	AB679045.1_Uncultured
	AB679015.1_Uncultured
	AB678923.1_Uncultured
	AB678855.1_Uncultured
	AB678846.1_Uncultured
	AB678821.1_Uncultured
	HQ611393.1_Uncultured
	HQ456121.1_Uncultured
	HQ456019.1_Uncultured
	HQ455999.1_Uncultured
	HQ455989.1_Uncultured
	HQ455966.1_Uncultured
	HQ455930.1_Uncultured
	HQ455917.1_Uncultured
	HQ455902.1_Uncultured
	EU052409.1_Uncultured
	AY896329.1_Uncultured
**AY896371.1_GammaA**	AB679087.1_Uncultured
	AB679067.1_Uncultured
	AB679014.1_Uncultured
	AB678896.1_Uncultured
	AB678852.1_Uncultured
	HQ586455.1_Uncultured
	HQ611683.1_Uncultured
	HQ611681.1_Uncultured
	HQ456096.1_Uncultured
	HQ456045.1_Uncultured
	HQ456041.1_Uncultured
	HQ455978.1_Uncultured
	HQ455908.1_Uncultured
AB678941.1_Uncultured	AB678933.1_Uncultured
	HQ586414.1_Uncultured
	HQ586436.1_Uncultured
	HQ611578.1_Uncultured
	HQ611376.1_Uncultured
	HQ456095.1_Uncultured
	HQ455996.1_Uncultured
	HQ455995.1_Uncultured

	HQ455994.1_Uncultured
KF960627.1_Uncultured	KF960619.1_Uncultured
	KF960599.1_Uncultured
	KF960581.1_Uncultured
	HQ456044.1_Uncultured
	HQ456033.1_Uncultured
	HQ456031.1_Uncultured
	EU151863.1_Uncultured
	DQ831856.1_Uncultured
KF546458.1_Uncultured	AB678877.1_Uncultured
	KC013049.1_Uncultured
	HQ611357.1_Uncultured
	HQ611354.1_Uncultured
	AF016613.2_Unidentifi
	DQ118237.2_Uncultured
	HQ634484.1_Uncultured
HQ611867.1_Uncultured	EF568515.1_Uncultured
	EF568526.1_Uncultured
	EF568522.1_Uncultured
KF546455.1_Uncultured	HQ611396.1_Uncultured
	HQ611392.1_Uncultured
	HQ611398.1_Uncultured
HQ611490.1_Uncultured	HQ611488.1_Uncultured
	AY896369.1_Uncultured
AY706889.1_Church	AB678848.1_Uncultured
	AY706890.1_Uncultured
AB678807.1_Uncultured	EU052543.1_Uncultured
	EU052318.1_Uncultured
HQ611581.1_Uncultured	HQ611380.1_Uncultured
	AY896334.1_Uncultured
AY706892.1_Uncultured	AY706891.1_Uncultured
	DQ062535.1_Uncultured
KF546462.1_Uncultured	HQ611777.1_Uncultured
	HQ611405.1_Uncultured
KC013226.1_Uncultured	KC013220.1_Uncultured
	HQ456102.1_Uncultured
KF619537.1_Uncultured	HQ611669.1_Uncultured
KF960630.1_Uncultured	KC013050.1_Uncultured
AB727471.1_Uncultured	AB727483.1_Uncultured
KF740746.1_Uncultured	HQ586461.1_Uncultured
HQ611404.1_Uncultured	HQ456110.1_Uncultured
HQ611356.1_Uncultured	HQ611355.1_Uncultured
EF204556.1_Uncultured	HQ660814.1_Uncultured
HQ611864.1_Uncultured	HQ611851.1_Uncultured
HQ611865.1_Uncultured	HQ611855.1_Uncultured
**HQ611839.1_Uncultured**	HQ611831.1_Uncultured
HQ611646.1_Uncultured	HQ611637.1_Uncultured

**HM210397.1_Gamma3...**	HM210403.1_Uncultured
HQ611480.1_Uncultured	HQ611473.1_Uncultured
DQ062516.1_Uncultured	DQ118223.1_Uncultured
DQ062518.1_Uncultured	DQ062517.1_Uncultured
DQ118232.1_Uncultured	
AB678915.1_Uncultured	
AB678854.1_Uncultured	
AB678820.1_Uncultured	
HQ586421.1_Uncultured	
HQ611691.1_Uncultured	
HQ611651.1_Uncultured	
HQ611627.1_Uncultured	
HQ456108.1_Uncultured	
HQ456105.1_Uncultured	
HQ455964.1_Uncultured	
HQ455918.1_Uncultured	
HQ455883.1_Uncultured	
AY800138.1_Uncultured	
AY896370.1_Uncultured	
KF546469.1_Uncultured	
KF546466.1_Uncultured	
KF546465.1_Uncultured	
KF546454.1_Uncultured	
AB678960.1_Uncultured	
KC013173.1_Uncultured	
KC013054.1_Uncultured	
KC013051.1_Uncultured	
KC013047.1_Uncultured	
AB727478.1_Uncultured	
AB727470.1_Uncultured	
HQ586447.1_Uncultured	
HQ611955.1_Uncultured	
HQ611926.1_Uncultured	
HQ611878.1_Uncultured	
HQ611869.1_Uncultured	
HQ611840.1_Uncultured	
HQ611836.1_Uncultured	
HQ611680.1_Uncultured	
HQ611677.1_Uncultured	
HQ611668.1_Uncultured	
HQ611593.1_Uncultured	
HQ611553.1_Uncultured	
HQ611551.1_Uncultured	
HQ611547.1_Uncultured	
HQ611537.1_Uncultured	
HQ611515.1_Uncultured	
HQ611509.1_Uncultured	

HQ611485.1_Uncultured
HQ611482.1_Uncultured
HQ611481.1_Uncultured
HQ611472.1_Uncultured
HQ611468.1_Uncultured
HQ611413.1_Uncultured
HQ611365.1_Uncultured
HQ455968.1_Uncultured
HQ455967.1_Uncultured
HQ455890.1_Uncultured
EU052529.1_Uncultured
EU052412.1_Uncultured
EU693424.1_Uncultured
DQ269145.1_Uncultured
DQ481333.1_Uncultured
AY800134.1_Uncultured
DQ118229.1_Uncultured
AY896315.1_Uncultured
KF619536.1_Uncultured
KF960592.1_Uncultured
KF740734.1_Uncultured
AB679076.1_Uncultured
AB679026.1_Uncultured
AB678955.1_Uncultured
KC013059.1_Uncultured
AB727482.1_Uncultured
HQ586462.1_Uncultured
HQ586458.1_Uncultured
HQ586451.1_Uncultured
HQ586449.1_Uncultured
HQ586440.1_Uncultured
HQ586430.1_Uncultured
HQ586426.1_Uncultured
HQ586425.1_Uncultured
HQ586424.1_Uncultured
HQ611912.1_Uncultured
HQ611888.1_Uncultured
HQ611884.1_Uncultured
HQ611882.1_Uncultured
HQ611857.1_Uncultured
HQ611845.1_Uncultured
HQ611686.1_Uncultured
HQ611676.1_Uncultured
HQ611663.1_Uncultured
HQ611657.1_Uncultured
HQ611604.1_Uncultured
HQ611572.1_Uncultured

HQ611538.1_Uncultured
HQ611500.1_Uncultured
HQ611469.1_Uncultured
HQ611360.1_Uncultured
HQ456098.1_Uncultured
HQ456001.1_Uncultured
HQ455998.1_Uncultured
HQ455991.1_Uncultured
HQ455960.1_Uncultured
EU052666.1_Uncultured
EU052611.1_Uncultured
EU052555.1_Uncultured
EU052408.1_Uncultured
EU052343.1_Uncultured
EU052342.1_Uncultured
EU052319.1_Uncultured
EF568521.1_Uncultured
DQ825742.1_Uncultured
DQ481335.1_Uncultured
DQ481334.1_Uncultured
DQ481331.1_Uncultured
DQ481328.1_Uncultured
KF546457.1_Uncultured
HQ586441.1_Uncultured
HQ586439.1_Uncultured
HQ586432.1_Uncultured
HQ586412.1_Uncultured
HQ611424.1_Uncultured
HQ611409.1_Uncultured
DQ062537.1_Uncultured
DQ118236.2_Uncultured
DQ404435.1_Uncultured
DQ404436.1_Uncultured
DQ481327.1_Uncultured
HQ455891.1_Uncultured
HQ455990.1_Uncultured
HQ456099.1_Uncultured
HQ456115.1_Uncultured
HQ611369.1_Uncultured
HQ611378.1_Uncultured
HQ611415.1_Uncultured
KF546459.1_Uncultured
DQ404438.1_Uncultured
HQ456116.1_Uncultured
EU052329.1_Uncultured
KF546463.1_Uncultured
KC013052.1_Uncultured

HQ586452.1_Uncultured
HQ611430.1_Uncultured
HQ586450.1_Uncultured
HQ586442.1_Uncultured
HQ611353.1_Uncultured
HQ630797.1_Uncultured
HQ611918.1_Uncultured
HQ611887.1_Uncultured
HQ611883.1_Uncultured
HQ611667.1_Uncultured
HQ611600.1_Uncultured
HQ611504.1_Uncultured
HQ611499.1_Uncultured
HQ611498.1_Uncultured
HQ611489.1_Uncultured
HQ611493.1_Uncultured
HQ611436.1_Uncultured
EU052413.1_Moisander.
HQ611400.1_Uncultured
HQ611377.1_Uncultured
DQ481325.1_Uncultured
HQ611781.1_Uncultured
HQ611768.1_Uncultured
HQ611367.1_Uncultured
HQ456013.1_Uncultured
HQ456012.1_Uncultured
HQ455979.1_Uncultured
HQ455961.1_Uncultured
EU159551.1_Uncultured
DQ062532.1_Uncultured
DQ118238.2_Uncultured
HQ455962.1_Uncultured
EU159550.1_Uncultured
EU159549.1_Uncultured
GQ475478.1_Uncultured
EU052405.1_Uncultured
EU052380.1_Uncultured
EU052406.1_Uncultured
EU052327.1_Uncultured
EU159553.1_Uncultured
AY706894.1_Uncultured
AY706893.1_Uncultured
EU159548.1_Uncultured
EU159547.1_Uncultured
EU159546.1_Uncultured
DQ481330.1_Uncultured
DQ481323.1_Uncultured

AY800139.1_Uncultured
AY896330.1_Uncultured
KF546468.1_Uncultured
KF546456.1_Uncultured
HQ586469.1_Uncultured
HQ455909.1_Uncultured
EU052350.1_Uncultured
HQ630796.1_Uncultured
HQ630792.1_Uncultured
HQ586453.1_Uncultured
HQ611790.1_Uncultured
HQ611431.1_Uncultured
HQ611778.1_Uncultured
EU159552.1_Uncultured
DQ118230.1_Uncultured
HQ611810.1_Uncultured
HQ611754.1_Uncultured
HQ611643.1_Uncultured
HQ611426.1_Uncultured
HQ611625.1_Uncultured
DQ118235.1_Uncultured
KF151828.1_Uncultured
KF151834.1_Uncultured
KF151825.1_Uncultured
KF151824.1_Uncultured
KF151510.1_Uncultured
KF151501.1_Uncultured
KF151762.1_Uncultured
KF546464.1_Uncultured
KF546460.1_Uncultured
HM210395.1_Uncultured
HQ611824.1_Uncultured
HQ611817.1_Uncultured
HQ611802.1_Uncultured
HQ611769.1_Uncultured
HQ611737.1_Uncultured
HQ611713.1_Uncultured
HQ611712.1_Uncultured
HQ611711.1_Uncultured
HQ611708.1_Uncultured
HQ611641.1_Uncultured
HQ611603.1_Uncultured
HQ611414.1_Uncultured
HQ611395.1_Uncultured
HQ611390.1_Uncultured
HQ611385.1_Uncultured
KF546470.1_Uncultured

KF546461.1_Uncultured
HQ611773.1_Uncultured
HQ611730.1_Uncultured
HQ611717.1_Uncultured
DQ118233.1_Uncultured
KF151836.1_Uncultured
KF151813.1_Uncultured
KF151741.1_Uncultured
KF151485.1_Uncultured
KF151831.1_Uncultured
KF151823.1_Uncultured
KF151822.1_Uncultured
KF151821.1_Uncultured
KF151819.1_Uncultured
KF151816.1_Uncultured
KF151812.1_Uncultured
KF151811.1_Uncultured
KF151751.1_Uncultured
KF151749.1_Uncultured
KF151748.1_Uncultured
KF151736.1_Uncultured
KF151734.1_Uncultured
KF151513.1_Uncultured
KF151497.1_Uncultured
KF151490.1_Uncultured
KF151489.1_Uncultured
KF151487.1_Uncultured
KF151484.1_Uncultured
KF151826.1_Uncultured
KF151740.1_Uncultured
KF151504.1_Uncultured
KF151512.1_Uncultured
KF151500.1_Uncultured
EF204562.1_Uncultured
KF151814.1_Uncultured
KF151494.1_Uncultured
KF151493.1_Uncultured
KF151492.1_Uncultured
KF151815.1_Uncultured
KF151491.1_Uncultured
KF151817.1_Uncultured
KF151731.1_Uncultured
KF151725.1_Uncultured
KF151483.1_Uncultured
KF151730.1_Uncultured
KF151726.1_Uncultured
KF151724.1_Uncultured

KF151511.1_Uncultured
KF151509.1_Uncultured
KF151503.1_Uncultured
KF151498.1_Uncultured
KF151732.1_Uncultured
KF151727.1_Uncultured
HQ611853.1_Uncultured
HQ611931.1_Uncultured
HQ611835.1_Uncultured
HQ611644.1_Uncultured
HQ611833.1_Uncultured
HQ611785.1_Uncultured
HQ611780.1_Uncultured
HQ611590.1_Uncultured
HQ611782.1_Uncultured
HQ611747.1_Uncultured
HQ611762.1_Uncultured
HQ611741.1_Uncultured
HQ611474.1_Uncultured
HQ611564.1_Uncultured
KC013175.1_Uncultured
KC013172.1_Uncultured
HQ456117.1_Uncultured
HQ456107.1_Uncultured
HQ456106.1_Uncultured
KC013223.1_Uncultured
HM210386.1_Uncultured
HM210394.1_Uncultured
HQ611909.1_Uncultured
HQ611765.1_Uncultured
HM210396.1_Uncultured
HM210393.1_Uncultured
HQ611910.1_Uncultured
HM210388.1_Uncultured
HQ456037.1_Uncultured
EF204563.1_Uncultured
AY896456.1_Uncultured
DQ481326.1_Uncultured
DQ062533.1_Uncultured
DQ118222.1_Uncultured
DQ118218.1_Uncultured
KF960582.1_Uncultured
DQ062525.1_Uncultured
DQ062524.1_Uncultured
DQ118220.1_Uncultured
AY800137.1_Uncultured
AY800136.1_Uncultured

KF546467.1_Uncultured
HQ634488.1_Uncultured
AY800142.1_Uncultured
HQ611932.1_Uncultured
**AB679086.1_Uncultured**
DQ481329.1_Uncultured
HQ456047.1_Uncultured
HQ456043.1_Uncultured
DQ404424.1_Uncultured
DQ062526.1_Uncultured
DQ404413.1_Uncultured
HQ586468.1_Uncultured
DQ404427.1_Uncultured
DQ404410.1_Uncultured
DQ825739.1_Uncultured
DQ404418.1_Uncultured
HQ634498.1_Uncultured
AY800135.1_Uncultured
AY800140.1_Uncultured
AY896311.1_Uncultured
AY896314.1_Uncultured
KF546471.1_Uncultured
HQ630798.1_Uncultured
AY896304.1_Uncultured
AY896306.1_Uncultured
AY896372.1_Uncultured
DQ404439.1_Uncultured
